# Iridium-Catalyzed Double Convergent 1,3-Rearrangement/Hydrogenation of Allylic Alcohols

**DOI:** 10.1021/jacs.2c11289

**Published:** 2022-12-19

**Authors:** Jianping Yang, Luca Massaro, Weigao Hu, Bram B. C. Peters, Norman Birke, Chayamon Chantana, Thishana Singh, Pher G. Andersson

**Affiliations:** † Department of Organic Chemistry, Arrhenius Laboratory, Stockholm University, 106 91 Stockholm, Sweden; ‡ School of Chemistry and Physics, University of Kwazulu-Natal, Private Bag X54001, Durban 4000, South Africa

## Abstract

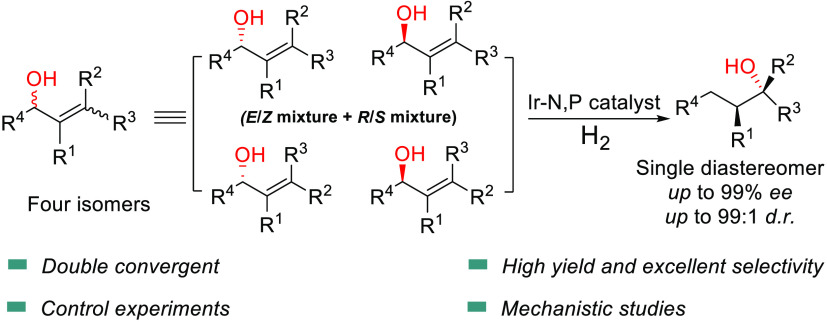

Enantioconvergent catalysis has the potential to convert different isomers of a starting material to a single highly enantioenriched product. Here we report a novel enantioselective double convergent 1,3-rearrangement/hydrogenation of allylic alcohols using an Ir-N,P catalyst. A variety of allylic alcohols, each consisting of a 1:1:1:1 mixture of four isomers, were converted to the corresponding tertiary alcohols with two contiguous stereogenic centers, in up to 99% *ee* and 99:1 *d.r*. DFT calculations, and control experiments suggest that the 1,3-rearrangement is the crucial stereodetermining element of the reaction.

## Introduction

**Scheme 1:**
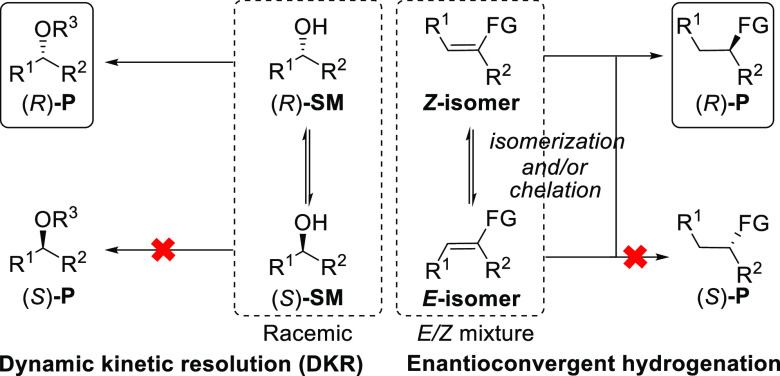
Enantioconvergent Catalysis

Stereoconvergent catalysis is a green and sustainable approach that converts racemic or isomeric mixtures of starting materials to a single enantioenriched product. So far, enantioconvergent catalytic processes are involved in various dynamic kinetic resolution (DKR) reactions as well as in a few recently reported enantioconvergent hydrogenations (Scheme 1).^1^ For the DKR processes, a racemic starting material can be converted to enantiomerically enriched products with up to 100% yield and has been well investigated in the past half-century.^2^ For enantioconvergent hydrogenations, the *E* and *Z* olefins, which normally generate opposite enantiomers,^1^ are hydrogenated into the same enantiomerically enriched product independent of the double-bond geometry. Till date, no method exists that can convert multiple isomers (more than two isomers) to a single enantiopure product.

**Figure 1:**
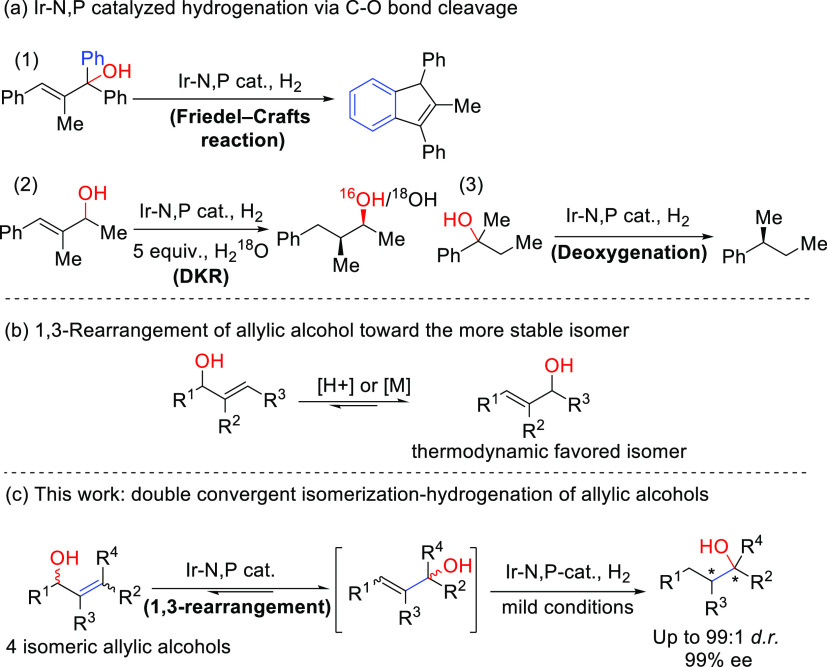
Development of enantioconvergent asymmetric synthesis of tertiary alcohols containing two contiguous stereogenic centers. (a) Ir-N,P catalyzed hydrogenation *via* C–O bond cleavage: (1) *via* Friedel−Crafts; (2) *via* DKR; and (3) *via* deoxygenation. (b) 1,3-Rearrangement of allylic alcohol. (c) This work.

Enantioenriched alcohols, especially tertiary alcohols and their derivatives, provide valuable feedstock for the synthesis of bioactive natural products and pharmaceuticals.^3^ Consequently, their preparation has attracted great attention from synthetic chemists, and the development of efficient preparative methods has become a high priority goal in organic synthesis. In this regard, asymmetric hydrogenation of allylic alcohols is a direct and efficient way to prepare chiral alcohols. Over the past few years, several works have been reported on the Ir-catalyzed hydrogenation of di-/tri-substituted primary/secondary allylic alcohols with excellent enantioselectivity.^4^ However, quaternary alcohols are for obvious reasons not directly accessible via asymmetric hydrogenation. Iridium-hydride complexes are considered as potential Lewis acids and/or Brønsted acids in certain cases.^5^ In 2010, Burgess reported that many N,P-based Ir-complexes have a Brønsted acidity similar to acetic acid.^6^ In earlier investigations, the Ir-hydride species was found to be acidic, and in some cases, it has the capability to cleave the allylic alcohol C–O bond (Figure 1a).^7^ Exploiting this acidic feature, an Ir-N,P-catalyzed hydrogenative DKR process was discovered and the mechanistic studies showed that the acid-assisted isomerization was the crucial element of the reaction (Figure 1a (2))^4^ This reactivity pattern was also extended later to the deoxygenation of racemic alcohols to produce chiral alkanes (Figure 1a (3))^8^

**Figure 2:**
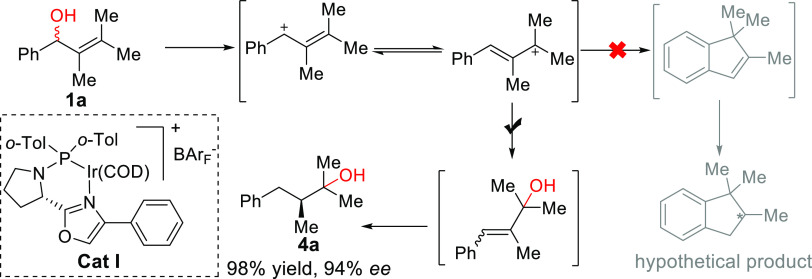
Initial idea and studies.

**Figure 3:**
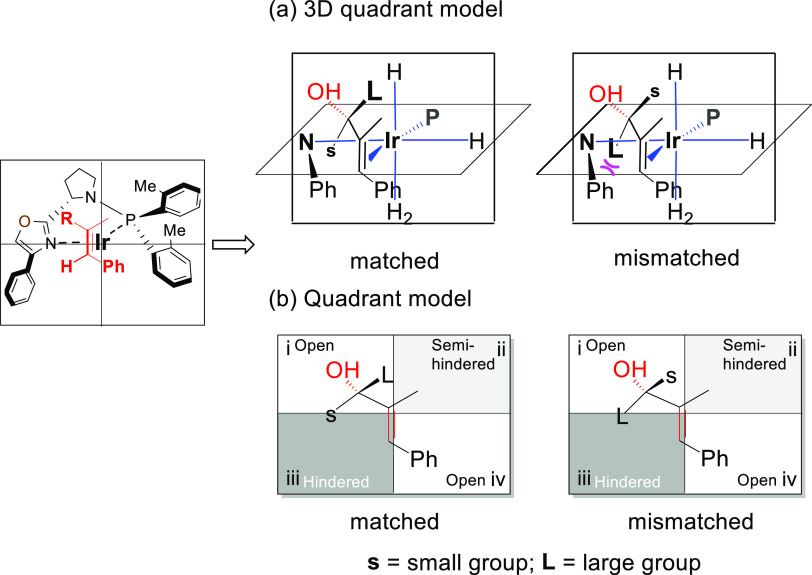
Origin of enantioselectivity. (a) 3D quadrant model. (b) Quadrant model.

The regioisomers of allylic alcohols have different thermodynamic stabilities due to the diverse extent of conjugation, substitution, and steric hindrance (Figure 1b). It has been reported that in the presence of Brønsted acids or transition metal complexes, allylic alcohols could be transformed to more stable isomers *via* 1,3-rearrangement.^9^ However, the reported methods are usually limited to di- or tri-substituted allylic alcohols, and to obtain geometrically pure products (*E* or *Z*), geometrically pure starting material is always required.

Considering the acidic nature of the Ir-N,P catalyst, the possible 1,3-rearrangement of allylic alcohols and the efficiency of enantioconvergent catalysis led us to investigate a possible double convergent 1,3-rearrangement/hydrogenation of allylic alcohols (Figure 1c). As shown in Figure 1c, a racemic, 1:1 *E*/*Z* mixture of a tetrasubstituted allylic alcohol was synthesized and subjected to hydrogenation. As the direct hydrogenation of a tetrasubstituted olefin is slow, an isomerization toward the trisubstituted allylic alcohol intermediate took place, followed by hydrogenation to an enantiopure tertiary alcohol. After optimization of the reaction, an impressive double stereo- and enantioconvergent catalytic process was observed, in which all four isomeric allylic alcohols were converted into a single enantiomer in a single step.

## Results and Discussion

### Initial Idea and Studies

In 2016, we reported on the efficient asymmetric hydrogenation of various allylic alcohols.^7^ During that study, we sometimes observed carbocation formation in the allylic position. For instance, the attempted hydrogenation of a diphenyl-substituted allylic tertiary alcohol (Figure 1a (1)) led to a Friedel–Crafts product instead of the expected hydrogenation product.

These results compelled us to prepare a new substrate **1a**, which was thought to be reduced to the saturated 1*H*-indane *via* a trisubstituted 1*H*-indene intermediate (Figure 2, top). Interestingly, when the hydrogenation of **1a** was attempted, only the tertiary alcohol **4a** was obtained with 80% yield and 94% *ee* when **Cat I** was used (Figure 2, bottom). In this case, the hydroxyl group was shifted *via* 1,3-rearrangement to form a trisubstituted allylic alcohol, which then underwent asymmetric hydrogenation in a highly enantioselective manner.

### Optimization of the Reaction Conditions

The stereoselective transformation of **1a** into **4a** suggested that the 1,3-rearrangement/hydrogenation cascade held potential to become a useful reaction. To further evaluate the efficiency of this methodology, an isopropyl group was introduced, giving rise to the asymmetric olefin **2a**. In the first attempts, a 1:1 *E*/*Z* mixture of racemic **2a** was hydrogenated using **Cat I** in CH_2_Cl_2_ under 10 bar of H_2_. Initially, only 20% of the product **5a** was observed together with a large number of byproducts obtained from deoxygenation, dimerization (ether formation), and their respective hydrogenated products. Screening of different solvents (Table 1, entries 1–4) revealed that PhCl resulted in a more selective reaction, giving **5a** in 80% yield (Table 1, entry 4) and excellent *d.r.* and *ee* (97/3 *d.r.*, 98% *ee*). Further optimization showed that PhF was the best solvent in terms of reactivity, enantioselectivity, and diastereoselectivity (87% of product, 97/3 *d.r.*, 98% *ee*, Table 1, entry 5). Subsequently, a catalyst screening established complex **Cat II** to be the most efficient and stereoselective catalyst for this reaction (Table 1, entry 6). To conclude, the optimal conditions for good yield, good enantioselectivity, and stereoselectivity are 1.0 mol % of **Cat II**, 10 bar of H_2_ pressure, and PhF as the solvent (entry 6). Notably, in this case, a 1:1 *E*/*Z* mixture of the racemic alcohol, which consists of four different isomers in total, is converted to a single diastereomer, enabling an efficient double convergent 1,3-rearrangement/hydrogenation reaction.

**Table 1:**
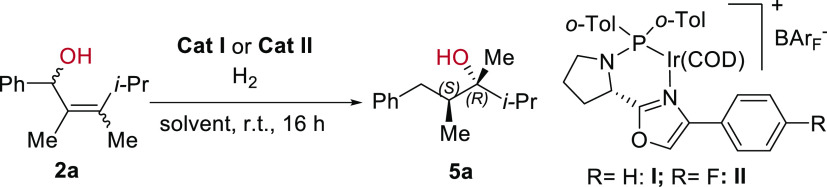
Optimization of Reaction Conditions^a^

entry	pressure (bar)	catalyst	solvent	**5a** (%)	*d.r.*	*ee* (%)
1	10	**Cat I**	CH_2_Cl_2_	20		
2	10	**Cat I**	PhCH_3_	30		
3	10	**Cat I**	PhCF_3_	76	97/3	95
4	10	**Cat I**	PhCl	80	97/3	98
5	10	**Cat I**	PhF	87	97/3	98
6	10	**Cat II**	PhF	91	98/2	99

a Reaction conditions: 0.05 mmol of substrate (1:1–2:1 ratio of the *E*/*Z* mixture), 1 mol % of catalyst, 5 bar of H_2_, 1 mL of solvent. **2a** was detected by ^1^H-NMR spectroscopy with mesitylene as an internal standard. Enantiomeric excesses were determined by chiral SFC or GC/MS.

### Substrate Scope

With the optimized conditions in hand, the substrate scope of the asymmetric 1,3-rearrangement/hydrogenation of allylic alcohols was examined. First, we evaluated the hydrogenation of various 2,3-dimethyl-1-phenylbut-2-en-1-ol derivatives (Table 2a). Substrates bearing different substituents on the phenyl ring were all successfully hydrogenated to give the desired tertiary alcohols **4a-4h**. Both electron-donating and electron-withdrawing substituents gave good to excellent isolated yields (84–98%) and excellent enantioselectivity (90–99% *ee*). Interestingly, the ortho-methyl-substituted substrate **33g** gave 99% *ee* (ortho substitution of styrenic derivatives normally results in lower enantioselectivities in asymmetric hydrogenations). The naphthyl group (**4h)** was also tolerated (86% yield, 90% *ee*). Further investigation was focused on more challenging substrates having unsymmetrical olefins that exist in *E/Z* mixtures and result in two contiguous stereogenic centers.

**Table 2:**
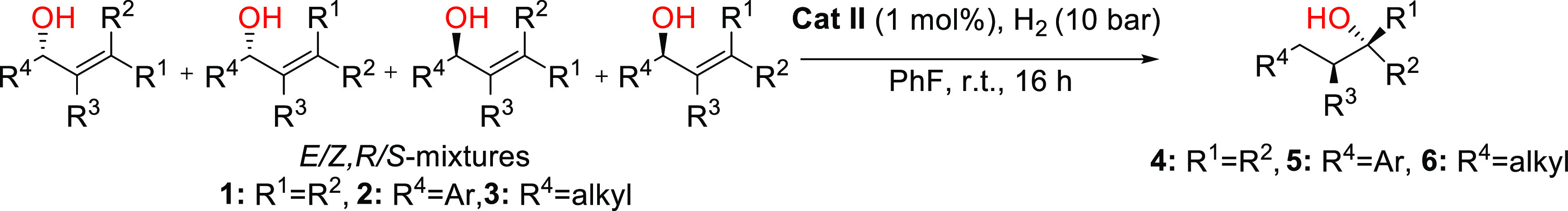

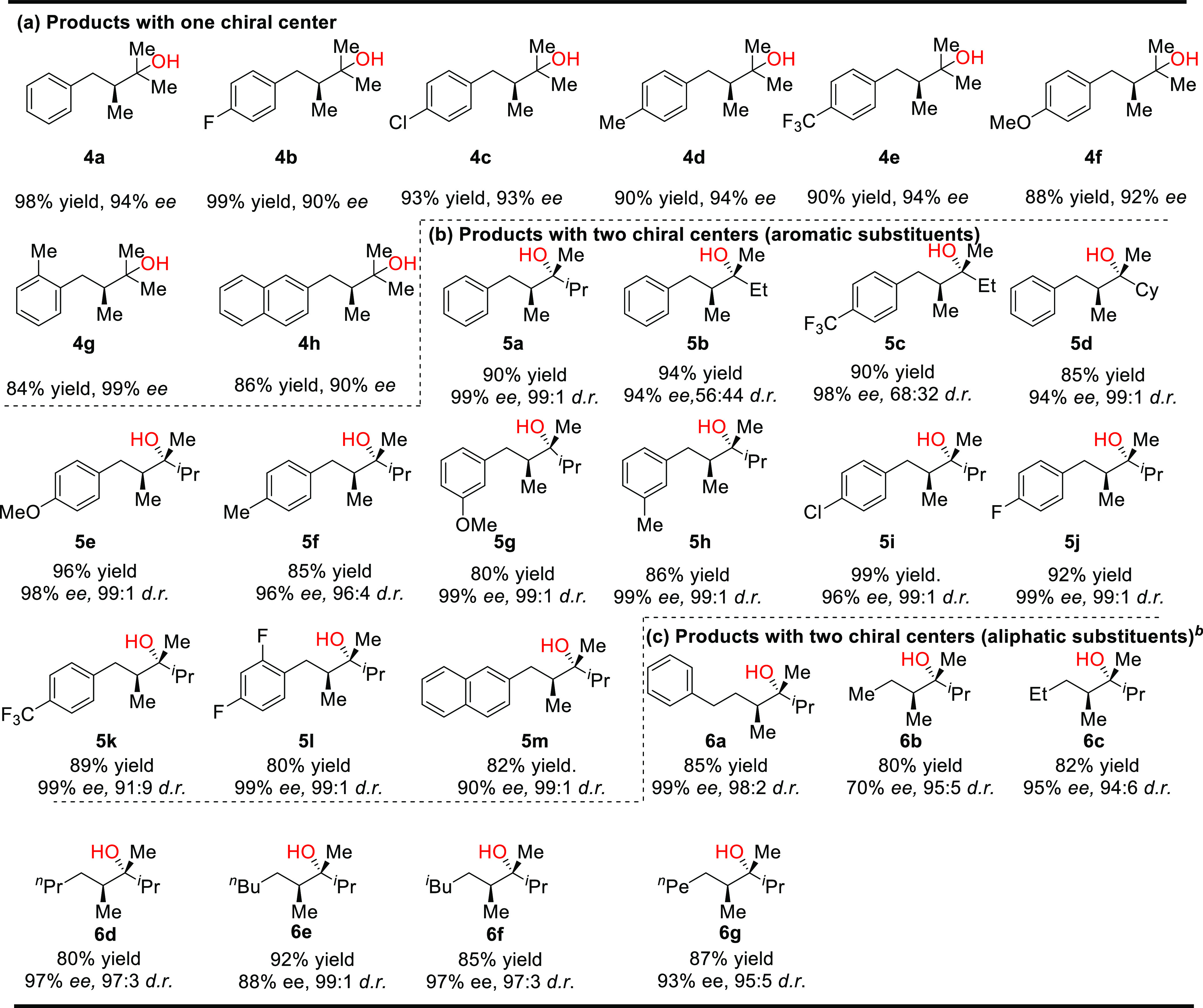
Substrates Scope^a^,^b^

a Reaction conditions: 0.05 mmol of substrate (1:1 ratio of *E*/*Z* mixture), 1 mol % of catalyst, 1 mL of solvent. Enantiomeric excesses were determined by chiral SFC or GC/MS.

b 1 bar of H_2_.

Substrate (*E*/*Z*)-**2a** was synthesized by simply adding Grignard reagents to *E*/*Z* mixtures of the corresponding aldehydes (∼1:1 ratio). The resulting product mixtures are very difficult to separate by column chromatography. However, when subjecting these 1:1:1:1 mixtures of isomers directly to the 1,3-rearrangement/hydrogenation protocol, all isomers were readily converted to a single, optically pure tertiary alcohol in excellent isolated yield, enantioselectivity, and stereoselectivity. First, substrates bearing different alkyl groups on the double bond, ranging from ethyl, isopropyl, and cyclohexyl groups were evaluated. All of these were hydrogenated efficiently (**5a**-**d**) in good yield (85–91%) and enantioselectivities (90–99% *ee*). Substrates with a less bulky substituent (Et, **5b-c**) gave lower diastereoselectivity. The ^*i*^Pr group resulted in the highest selectivity and afforded 90% of isolated yield with 99% *ee* and 98.5:1.5 *d.r.* Substrates bearing different functional groups on the phenyl ring were also investigated. Electron-donating or electron-withdrawing substituents on the aromatic ring were tolerated and gave good to excellent isolated yields (80–96%), excellent enantioselectivity (90–99% *ee*), and outstanding stereoselectivity (96/4–99/1 *d.r.*) (Table 2, **5e**-**l**). The substrate with the naphthyl group (**5m**) resulted in similar stereoselectivity.

Usually, the 1,3-rearrangement of allylic alcohols requires an aryl group next to the C-O bond to give stable conjugated allylic alcohols.^10^ Moreover, the hydrogenation of allylic alcohols usually requires substrates bearing at least one aryl group on the γ-carbon in order to give high enantioselectivities. Gratifyingly, this protocol was found to be independent of the aliphatic or aromatic nature of the substituents, and various aliphatic substrates **3** were efficiently and selectively hydrogenated under mild conditions. Interestingly, the H_2_ pressure could be reduced to 1 bar in these cases. The benzyl-substituted allylic alcohol **3a** delivered the desired product in 85% isolated yield with 98% *ee* and 98:2 *d.r.* Next, substrates having various alkyl groups from short to long aliphatic chains gave satisfactory results (89–99% *ee*, 94:6–99:1 *d.r*., Table 2, **6b**-**g**). Generally, substrates bearing longer chains afford higher enantioselectivities.

**Scheme 2:**
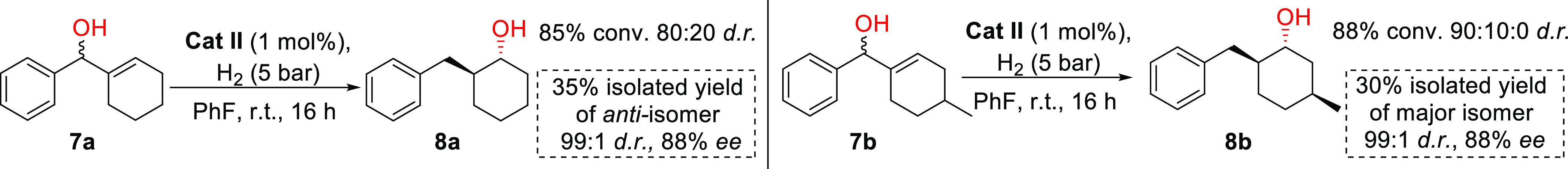
Hydrogenation of Trisubstituted Allylic Alcohols *via* 1,3-Rearrangement Reaction conditions: 0.05 mmol of substrate, 1 mol % of catalyst, 1 mL of solvent. Yields are isolated yields of the major diastereomer. Enantiomeric excesses were determined by chiral SFC or GC/MS.

A variety of tetrasubstituted allylic alcohols were successfully converted to enantioenriched tertiary alcohols *via* 1,3-rearrangement/asymmetric hydrogenation. We then proceeded to investigate the hydrogenation of some trisubstituted allylic alcohols (Scheme 2). These are usually directly hydrogenated to the product; therefore, a competition between 1,3-rearrangement and direct hydrogenation will occur during the process. Interestingly, cyclohex-1-en-1-yl(phenyl)methanol **7a** was converted to the desired secondary alcohol with 88% *ee*. The major *anti*-diastereomer (**8a**) was purified by column chromatography and was isolated in 35% yield. Another interesting result was observed when a methyl group was introduced on the cyclohexenyl ring (**7b**). In this case, the chirality was transferred from the remote carbon and resulted in 88% *ee* and 30% isolated yield of the major diastereomer. These results show that some trisubstituted olefins are also tolerated in the 1,3-rearrangement/hydrogenation although competitive direct hydrogenation byproducts are also generated.

**Scheme 3:**
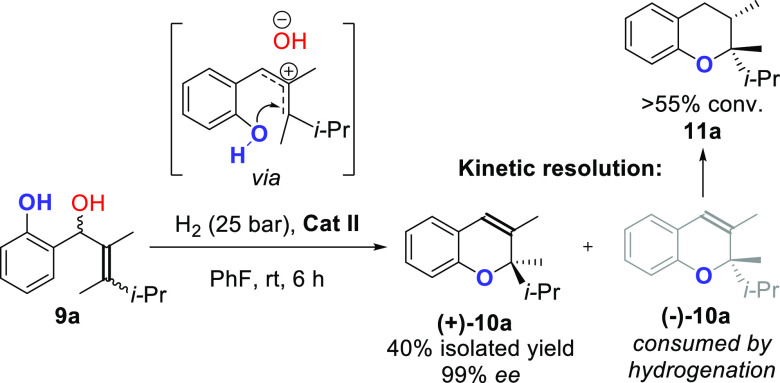
Synthesis and Kinetic Resolution of Chiral *2H*-Chromene *via* Cascade 1,3-Rearrangement and Hydrogenation

Finally, we investigated if the presence of an internal nucleophile could intercept the allylic carbocation formed in the reaction. The transformation was performed on substrate **9**, having an additional hydroxyl group on the ortho position of the ring, which afforded 2*H*-chromene **10** as the product. Under optimized conditions, the intramolecular cyclization followed by asymmetric hydrogenation resulted in a very efficient kinetic resolution, and the remaining 2-chromene could be isolated in 40% yield with 99% *ee* (Scheme 3).

## Mechanistic Studies

### 1,3-Rearrangement

**Scheme 4:**
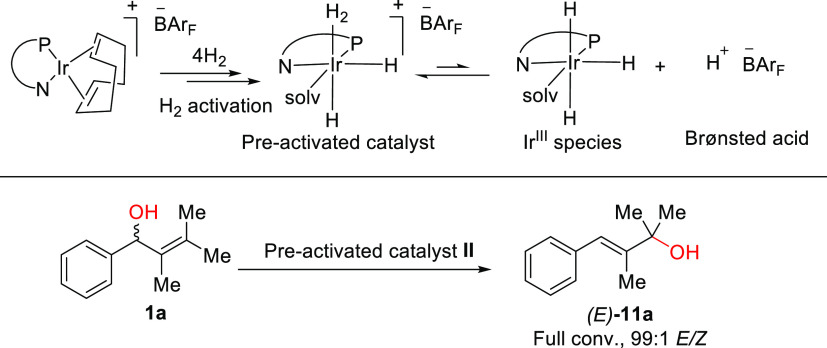
Synthesis of the 1,3-rearrangement intermediate using the pre-activated catalyst The pre-activated catalyst was prepared by treating the catalyst with hydrogen for 10 min and then degassed.

To further understand the stereochemical outcome of the 1,3-rearrangement, substrate **1a** was treated with the pre-activated catalyst **II**. This H_2_ activated catalyst is likely to behave similar (in the 1,3-rearrangement) as the active catalyst under hydrogenation conditions and generate iridium hydrides, [(N,P)*Ir(H)_2_(solv)_2_]BAr_F_, resulting in the liberation of HBAr_F_. The Ir-hydrides and formed Brønsted acid could be responsible for the 1,3-rearrangement besause Brønsted acids, transition metal complexes, and even neutral H_2_O^11^ have been reported to catalyze the 1,3-rearrangement. When **1a** was subjected to the pre-activated catalyst, a fast rearrangement took place and the racemic (*E*)-2,3-dimethyl-4-phenylbut-3-en-2-ol (***E*****-11a**) was formed in a 99:1 *E*/*Z* ratio (Scheme 4).

**Scheme 5:**
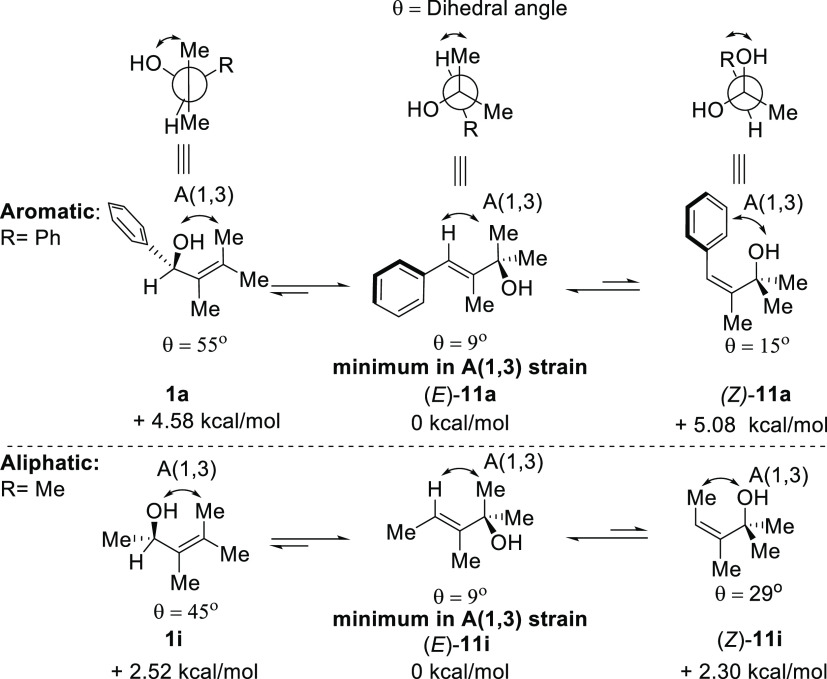
Calculated Energies for the Ground State Energies of Allylic Alcohols

To further understand this transformation, DFT calculations for an aryl substituted allylic alcohol and an alkyl substituted allylic alcohol were conducted (Scheme 5). The minimized conformations of the starting materials and possible rearrangement products are shown in Scheme 5. When the smallest substituent in the allylic position is nearly eclipsed with the double bond and the vinylic hydrogen, the 1,3-strain (A (1,3)) was minimized (θ = 9°). The *E*-isomer for the 1,3-rearrangement product was the most stable configuration (*E*-**11a**, *E*-**11i**). The other possible aromatic allylic alcohol conformations had energies of >4.58 kcal·mol^–1^, and the energies of the aliphatic example were >2.30 kcal·mol^–1^.

### Control Experiments

To better understand the features of the 1,3-rearrangement of the allylic alcohol, several control experiments were conducted. First, substrate **2a** was added to complex **Cat II** in the absence of hydrogen. Under these conditions, the Ir-N,P complex can be regarded as a Lewis acid; however, no product was observed in this case (Table 3, entry 1). Next, the experiment was carried out using pre-activated **Cat II** together with 10 mol % K_2_CO_3_ to remove HBAr_F_ (Table 3, entry 2). No conversion was observed, which suggests that the neutral iridium trihydride species alone does not catalyze the 1,3-rearrangement. Finally, when substrate **2a** was treated with 10 mol % Brønsted acid (HBAr_F._2Et_2_O), a complex mixture of products was obtained (Table 3, entry 3), which excluded the possibility of the acid alone catalyzing the 1,3-rearrangement.

**Table 3:**
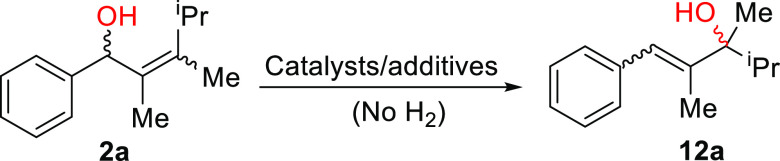
Control Experiments^a^

entry	catalyst	additive	conversion
1	precatalyst (1 mol %)		N.R
2	preactivated catalyst II (1 mol %)	K_2_CO_3_ (10 mol %)	N.R.
3	HBAr_F._2Et_2_O (10 mol %)		complex mixture

a N.R = no reaction.

Proposed Pathway

### via

In 2021, we reported on an efficient kinetic resolution protocol for a variety of trisubstituted allylic alcohols Ir-N,P-catalyzed asymmetric hydrogenation. In that case, an interaction between the hydroxyl group and iridium hydride was proposed to control the stereochemical outcome of the reaction. DFT calculation showed that a hydrogen bond between the alcohol and the iridium center is responsible for the enantioselectivity.

When the larger group on the carbinol is pointing toward the ligand backbone, the steric interactions disfavor the hydrogenation (Figure 3, mismatched enantiomer). Contrarily, there is a match for the other enantiomer, where the hydrogen bonding results in the smaller substituent pointing toward the catalyst (Figure 3, matched). This difference in steric clash resulted in an energy difference of 3.0 kcal·mol^–1^ for the two transition states.^4d^

**Scheme 6:**
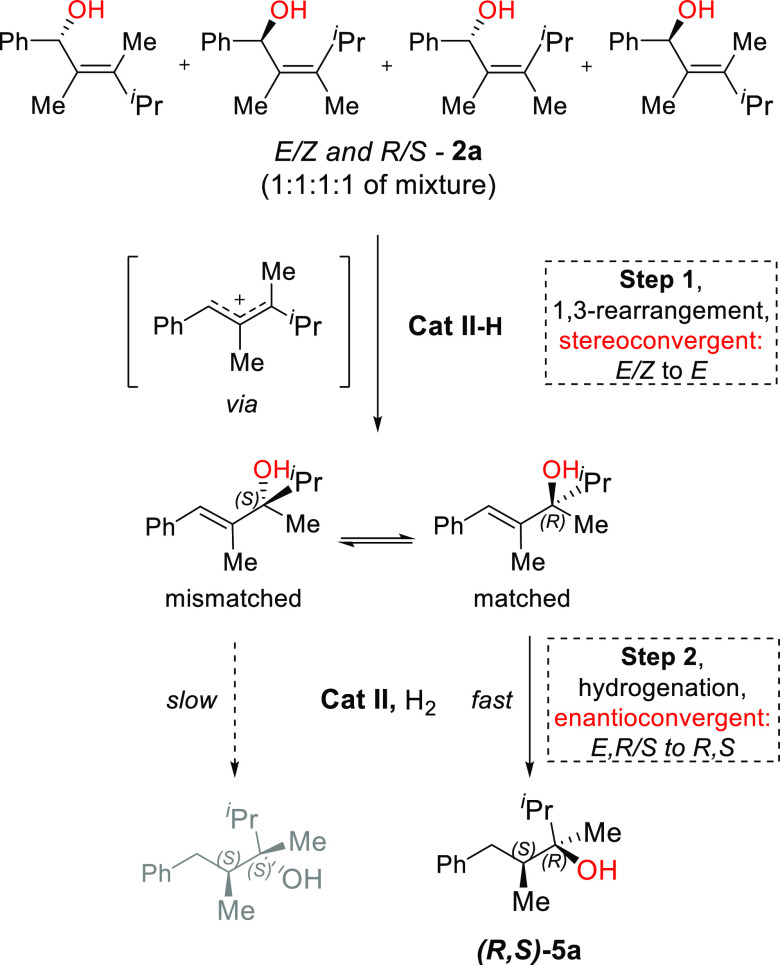
Proposed Mechanism for the 1,3-Rearrangement/Hydrogenation

Based on these control experiments, we propose that the OH group is cleaved with the assistance of the acidic Ir-hydride species. This results in 1,3-rearrangement taking place and forming the thermodynamically more stable isomer (Scheme 6, step 1). As mentioned above, the bulkier group ^*i*^Pr at the carbinol should point away from the ligand backbone (Figure 3, matched; Scheme 6, matched). Consequently, via a convergent hydrogenation, the racemic mixture is converted to a single stereoisomer with purity up to 99% *ee* and 99:1 *d.r.* (Scheme 6, Step 2).

## Conclusions

To conclude, we have developed an efficient catalytic system for a doubly convergent 1,3-rearrangement/hydrogenation of allylic alcohols. A variety of allylic alcohols consisting of a 1:1:1:1 mixture of four isomers were converted to the corresponding tertiary alcohols with up to 99% *ee* and 99:1 *d.r*. In addition, DFT calculations and control experiments agreed with the outcome of the 1,3-rearrangement. Finally, a rationale explaining the origin of selectivity in this enantioconvergent hydrogenation was also proposed.

## Supporting Information Available

Experimental procedures, characterization data of new compounds, separation of chiral products, NMR spectra of new compounds and computational details (PDF)
