# Supplementary material for: Iridium-Catalyzed Double Convergent 1,3-Rearrangement/Hydrogenation of Allylic Alcohols
Source: J Am Chem Soc. 2022 Dec 19;145(1):626–33. doi: 10.1021/jacs.2c11289 (PMC9837846; doi:10.1021/jacs.2c11289)
Supplement: Supplementary file 1 — ja2c11289_si_001.pdf [file ja2c11289_si_001.pdf]

**Iridium-catalyzed Double Convergent 1,3-Rearrangement/Hydrogenation of Allylic Alcohols**

**Jianping Yang,[a] Luca Massaro,[a] Weigao Hu,[a] Bram B. C. Peters,[a] Norman Birke,[a] Chayamon Chantana,[a] Thishana Singh,[b] and Pher G. Andersson[* a,b]**

[a]Department of Organic Chemistry, Stockholm University, Svante Arrhenius väg 16C, SE-10691 Stockholm, Sweden.

[b]School of Chemistry and Physics, University of Kwazulu-Natal, Private Bag X54001, Durban, 4000, South Africa.

*Corresponding author. Email: pher.andersson@su.se.

## Contents

*1. General methods* ........................ *S1*

*2. General procedure for the synthesis of starting materials* ........................ *S2*

2.1. General procedure for the synthesis of allylic alcohol ........................ *S2*

*3. General procedure for isomerization-hydrogenation of allylic alcohol* ..... *S11*

*4. Assignment of the absolute configurations of hydrogenated products.* ..... *S24*

*5. NMR spectra* ........................ *S25*

*6. Chromatograms* ........................ *S90*

*7. Computational details* ........................ *S98*

*8. References* ........................ *S105*

## 1. General methods

Unless otherwise noted, all reactions were conducted under dry nitrogen or argon atmosphere using magnetic stirring. $CH_2Cl_2$ was freshly distilled from calcium hydride under nitrogen; THF was freshly distilled from Na and benzophenone under argon; All other solvents and reagents were purchased from commercial suppliers and used without further purification. Chromatographic separations were performed on Kiesel gel 60 H silica gel (particle size: 0.063-0.100 mm). Thin layer chromatography (TLC) was performed on aluminium plates coated with Kieselgel 60 (0.20 mm, UV254) and visualized under ultraviolet light (ν = 254 nm), or by staining with ethanolic phosphomolybdic acid and heating. $^1$H NMR spectra were

recorded at 400 MHz in Chloroform-*d* at 25°C and referenced internally to the residual $CHCl_3$ peak (7.26 ppm). $^{13}C$ NMR spectra were recorded at 101 MHz in Chloroform-*d* at 25 °C and referenced to the central peak of Chloroform-*d* (77.16 ppm). $^{19}F$ NMR spectra were recorded at 377 MHz in Chloroform-*d* at 25 °C. $^{31}P$ NMR spectra were recorded at 202 MHz in Chloroform-*d* at 25 °C. Chemical shifts are reported in ppm (δ scale). Optical rotations were recorded on a thermostatic polarimeter using a sodium lamp (589 nm) and a 1.0 dm cell. Enantiomeric excesses and diastereomeric ratios were determined using SFC or GC (30 m columns, Helium gas carrier at 1 mL $min^{-1}$, constant pressure) with a MS detector. Racemic compounds were used for comparison. HRMS was performed on a Bruker microTOF with an ESI source.

## 2. General procedure for the synthesis of starting materials

### 2.1. General procedure for the synthesis of allylic alcohol

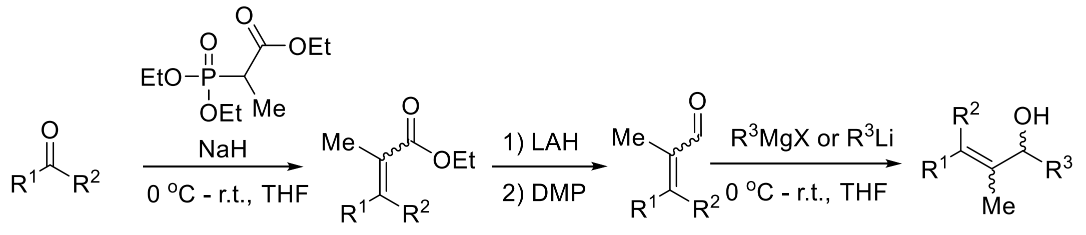


Scheme S1. Multi-steps synthesis of allylic alcohols.

Triethyl-2-phosphonopropionate (60 mmol, 1.2 equiv.) was slowly added to a stirring suspension of NaH (60% in mineral oil, 60 mmol, 1.2 equiv.) in 200 ml THF at 0 °C. After 30 min, the respective ketone (50 mmol, 1.0 equiv.) was added dropwise and the mixture was allowed to stir at room temperature or reflux over-night. Thereafter, the mixture was quenched with 200 ml $NH_4Cl$ and extracted with 100 ml $Et_2O$ (3 times). The combined organic phases were washed with brine, dried over $Na_2SO_4$ and evaporated in vacuo to give the crude product. The crude product was purified by column chromatography (pentane/$Et_2O$, 20:1) to obtain the product as an E/Z mixture.

The E/Z unsaturated ester (20 mmol, 1.0 equiv.) was dissolved in 200 ml dry THF and $LiAlH_4$ (30 mmol, 1.14 g, 1.5 equiv.) was added portionwise at 0 °C over 1 h. Then the mixture was stirred at room temperature until the starting material was consumed completely. At 0 °C, 1.14 mL $H_2O$ was added to the mixture, then 1.14 mL 10% NaOH followed by 3.42 mL $H_2O$ was added to quench the reaction. The mixture was filtered through celite. The filtrate was concentrated under reduced pressure to obtain the crude alcohol, which was used in further steps without additional purification.

The E/Z allylic alcohol (10 mmol, 1.0 equiv.) was dissolved in 50 ml dry DCM, and DMP (12 mmol, 1.2 equiv.) was added in the solution. After complete consumption of the starting material, the mixture was filtered and the filtrate was concentrated under vacuo. The crude was purified by distillation or column chromatography (pentane/$Et_2O$, 40:1) to obtain the E/Z unsaturated aldehyde.

The E/Z unsaturated aldehyde (1 mmol, 1.0 equiv.) was dissolved in 5 ml dry THF, then Grignard reagent or RLi (1.5 mmol, 1.5 equiv.) was added in the solution. After complete consumption of the starting material, the mixture was quenched with 5 ml $NH_4Cl$ and extracted with 5 ml $Et_2O$ (3 times). The combined organic phases were washed with brine, dried over $Na_2SO_4$ and evaporated in vacuum to give the crude product. The crude product was purified by column chromatography (pentane/$Et_2O$, 10:1-5:1) to obtain the E/Z mixture products.

**Characterization of new compounds:**

All the allylic alcohols were prepared starting from 1 mmol of the corresponding aldehydes.

**2,3-dimethyl-1-phenylbut-2-en-1-ol (1a)**

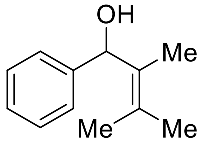


Colorless oil,149.6 mg (85% yield). $^1$H NMR (400 MHz, Chloroform-*d*) δ 7.37 – 7.30 (m, 4H), 7.26 – 7.20 (m, 1H), 5.86 (d, *J* = 2.97 Hz, 1H), 1.90 (q, *J* = 1.45 Hz, 3H), 1.74 (q, *J* = 1.02 Hz, 3H), 1.50 (p, *J* = 1.24 Hz, 3H). $^{13}$C NMR (101 MHz, Chloroform-*d*) δ 143.3, 129.6, 128.8, 128.3, 126.9, 125.6, 72.3, 21.3, 20.4, 12.4. HRMS-ESI: Found $[M+Na]^+$ = 199.1095; $C_{12}H_{16}ONa$ requires 199.1093.

**1-(4-fluorophenyl)-2,3-dimethylbut-2-en-1-ol (1b)**

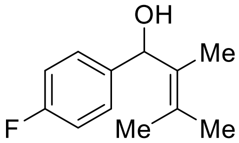


Colorless oil, 159.1 mg (82% yield). $^1$H NMR (400 MHz, Chloroform-*d*) δ 7.30 (dd, *J* = 8.42, 5.51 Hz, 2H), 7.00 (t, *J* = 8.70 Hz, 2H), 5.82 (s, 1H), 1.88 (d, *J* = 2.11 Hz, 3H), 1.73 (s, 3H), 1.48 (s, 3H). $^{13}$C NMR (101 MHz, Chloroform-*d*) δ 161.9 (d, *J* = 244.18 Hz), 138.9 (d, *J* = 2.92 Hz), 129.5, 129.0, 127.2 (d, *J* = 7.96 Hz), 115.0 (d, *J* = 21.23 Hz), 71.8, 21.3, 20.3, 12.3. $^{19}$F NMR (377 MHz, Chloroform-*d*) δ -116.6. **HRMS-ESI**: Found $[M+Na]^+$ = 217.0978; $C_{12}H_{15}FONa$ requires 217.0999.

**1-(4-chlorophenyl)-2,3-dimethylbut-2-en-1-ol (1c)**

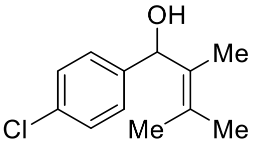


White solid, 159.6 mg (76% yield). $^1$H NMR (400 MHz, Chloroform-*d*) δ 7.35 – 7.28 (m, 4H), 5.84 (s, 1H), 1.92 (s, 3H), 1.76 (s, 3H), 1.49 (s, 3H). $^{13}$C NMR (101 MHz, Chloroform-*d*) δ 141.7, 132.5, 129.3, 129.3, 128.4, 127.1, 71.8, 21.3, 20.4, 12.3. **HRMS-ESI**: Found $[M+Na]^+$ = 233.0705; $C_{12}H_{15}ClONa$ requires 233.0704.

**2,3-dimethyl-1-(4-(trifluoromethyl)phenyl)but-2-en-1-ol (1d)**

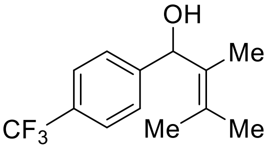


Colorless oil, 197.6 mg (81% yield). $^{1}$H NMR (400 MHz, Chloroform-*d*) δ 7.66 – 7.57 (m, 2H), 7.52 – 7.36 (m, 2H), 5.92 (s, 1H), 1.95 (q, *J* = 1.47 Hz, 3H), 1.77 (d, *J* = 1.10 Hz, 3H), 1.49 (q, *J* = 1.27 Hz, 3H). $^{13}$C NMR (101 MHz, Chloroform-*d*) δ 147.3, 129.8, 129.1 (q, *J* = 32.01 Hz), 126.0, 125.2 (q, *J* = 3.83 Hz), 124.4 (q, *J* = 268.66 Hz), 115.6, 71.9, 21.3, 20.4, 12.3. $^{19}$F NMR (377 MHz, Chloroform-*d*) δ -62.3. **HRMS-ESI**: Found [M+Na]$^+$ = 267.0956; $C_{13}H_{15}F_3ONa$ requires 267.0967.

**2,3-dimethyl-1-(p-tolyl)but-2-en-1-ol (1e)**

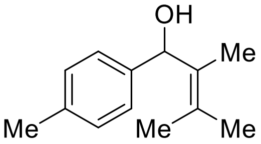


Colorless oil, 136.2 mg (72% yield). $^{1}$H NMR (400 MHz, Chloroform-*d*) δ 7.25 – 7.21 (m, 2H), 7.16 – 7.12 (m, 2H), 5.83 (s, 1H), 2.34 (s, 3H), 1.89 (q, *J* = 1.41 Hz, 3H), 1.73 (s, 3H), 1.51 (q, *J* = 1.47 Hz, 3H). $^{13}$C NMR (101 MHz, Chloroform-*d*) δ 140.2, 136.5, 136.3, 128.9, 128.8, 125.4, 72.1, 30.9, 21.2, 20.3, 12.2. **HRMS-ESI**: Found [M+Na]$^+$ = 213.1251; $C_{13}H_{18}ONa$ requires 213.1250.

**1-(4-methoxyphenyl)-2,3-dimethylbut-2-en-1-ol (1f)**

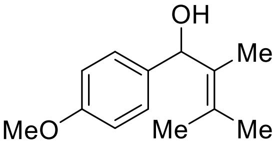


Colorless oil, 164.8 mg (80% yield). $^{1}$H NMR (400 MHz, Chloroform-*d*) δ 7.26 (d, *J* = 8.48 Hz, 2H), 6.87 (d, *J* = 8.70 Hz, 2H), 5.81 (s, 1H), 3.80 (s, 3H), 1.87 (s, 3H), 1.73 (s, 3H), 1.51 (s, 3H). $^{13}$C NMR (101 MHz, Chloroform-d) δ 158.5, 135.3, 129.5, 128.3, 126.7, 113.5, 71.9, 55.3, 21.2, 20.2, 12.2. **HRMS-ESI**: Found [M+Na]$^+$ = 229.1197; $C_{13}H_{18}O_2Na$ requires 229.1199.

**2,3-dimethyl-1-(o-tolyl)but-2-en-1-ol (1g)**

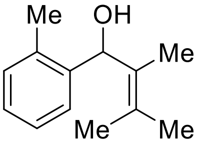


White solid, 148.2 mg (77% yield).$^{1}$H NMR (400 MHz, Chloroform-*d*) δ 7.63 (dd, *J* = 7.72, 1.46 Hz, 1H), 7.26 – 7.12 (m, 2H), 7.12 – 7.06 (m, 1H), 5.83 (s, 1H), 2.17 (s, 3H), 1.93 (q, *J* = 1.47 Hz, 3H), 1.72 (d, *J* = 1.17 Hz, 3H), 1.41 (p, *J* = 1.17 Hz, 3H). $^{13}$C NMR (101 MHz, Chloroform-*d*) δ 141.1, 134.7, 130.0, 129.8, 128.0, 126.7, 125.7, 125.7, 70.2, 21.2, 20.1, 19.1, 12.9. **HRMS-ESI**: Found [M+Na]$^+$ = 213.1250; $C_{13}H_{18}ONa$ requires 213.1250.

**2,3-dimethyl-1-(naphthalen-2-yl)but-2-en-1-ol (1h)**

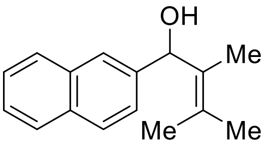


Yellow oil, 165.0 mg (73% yield). $^{1}$H NMR (400 MHz, Chloroform-*d*) δ 7.96 – 7.75 (m, 4H), 7.59 – 7.43 (m, 2H), 7.42 – 7.35 (m, 1H), 6.04 (s, 1H), 2.00 (s, 3H), 1.79 (s, 3H), 1.54 (s, 3H). $^{13}$C NMR (101

MHz, Chloroform-*d*) δ 140.7, 133.3, 132.5, 129.4, 129.1, 128.0, 127.8, 127.6, 126.0, 125.5, 124.2, 123.8, 72.3, 21.2, 20.4, 12.3. **HRMS-ESI**: Found $[M+Na]^+$ =249.1252; $C_{16}H_{18}ONa$ requires 249.1250.

***(E/Z)*-2,3,4-trimethyl-1-phenylpent-2-en-1-ol (2a)**

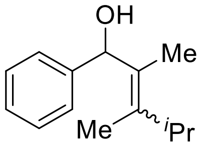


Colorless oil, 167.3 mg (82% yield). $^1$H NMR (400 MHz, Chloroform-*d, Z*-isomer) δ 7.37 – 7.28 (m, 4H), 7.26 – 7.18 (m, 1H), 5.95 (s, 1H), 3.20 (hept, *J* = 6.79 Hz, 1H), 1.61 (q, *J* = 1.00 Hz, 3H), 1.48 (q, *J* = 0.99 Hz, 3H), 1.06 (d, *J* = 3.61 Hz, 3H), 1.04 (d, *J* = 3.61 Hz, 3H). $^1$H NMR (400 MHz, Chloroform-*d, E*-isomer) δ 7.40 – 7.28 (m, 4H), 7.26 – 7.20 (m, 1H), 5.83 (s, 1H), 2.88 (hept, *J* = 6.85 Hz, 1H), 1.76 (q, *J* = 1.41 Hz, 3H), 1.51 (q, *J* = 1.38 Hz, 3H), 1.01 (d, *J* = 6.91 Hz, 3H), 0.99 (d, *J* = 6.89 Hz, 3H). $^{13}$C NMR (101 MHz, Chloroform-*d, Z*-isomer) δ 143.3, 138.6, 128.3, 128.3, 126.8, 125.8, 71.4, 29.7, 21.4, 21.3, 13.1, 13.0. $^{13}$C NMR (101 MHz, Chloroform-*d, E*-isomer) δ 143.2, 137.8, 128.3, 128.3, 126.8, 125.7, 72.5, 30.5, 20.5, 20.2, 12.0, 11.7. **HRMS-ESI**: Found $[M+Na]^+$ = 227.1409; $C_{14}H_{20}ONa$ requires 227.1406.

***(E/Z)*-2,3-dimethyl-1-phenylpent-2-en-1-ol (2b)**

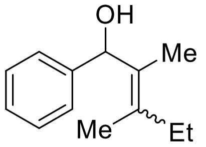


Colorless oil, 166.0 mg (86% yield). $^1$H NMR (400 MHz, Chloroform-*d, E/Z* Mixture) δ 7.39 – 7.29 (m, 8H), 7.26 – 7.20 (m, 2H), 5.84 (s, 1H), 5.83 (s, 1H), 2.39 – 2.19 (m, 2H), 2.10 (qd, *J* = 7.55, 2.92 Hz, 2H), 1.88 (q, *J* = 1.39 Hz, 3H), 1.73 (q, *J* = 1.00 Hz, 3H), 1.51 (q, *J* = 1.48 Hz, 3H), 1.48 (q, *J* = 0.94 Hz, 3H), 1.08 (t, *J* = 7.54 Hz, 3H), 1.01 (t, *J* = 7.58 Hz, 3H). $^{13}$C NMR (101 MHz, Chloroform-*d*, *E/Z* Mixture) δ 143.2, 143.2, 135.1, 134.5, 129.1, 129.1, 128.3, 128.3, 126.8, 126.8, 125.8, 125.6, 72.4, 72.0, 28.1, 27.2, 18.7, 17.9, 13.9, 12.6, 12.3, 11.7. **HRMS-ESI**: Found $[M+Na]^+$ = 213.1249; $C_{13}H_{18}ONa$ requires 213.1250.

***(E/Z)*-2,3-dimethyl-1-(4-(trifluoromethyl)phenyl)pent-2-en-1-ol (2c)**

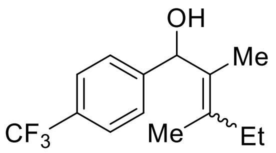


Colorless oil, 219.3 mg (85% yield). $^1$H NMR (400 MHz, Chloroform-*d, Z*-isomer) δ 7.58 (d, *J* = 8.15 Hz, 2H), 7.49 – 7.47 (m, 2H), 5.87 (s, 1H), 2.40 – 2.20 (m, 2H), 1.74 (q, *J* = 0.97 Hz, 3H), 1.43 (q, *J* = 0.92 Hz, 3H), 1.10 (t, *J* = 7.54 Hz, 3H). $^1$H NMR (400 MHz, Chloroform-*d, E*-isomer) δ 7.58 (d, *J* = 8.15 Hz, 2H), 7.46 – 7.44 (m, 2H), 5.85 (s, 1H), 2.19 – 2.01 (m, 2H), 1.90 (q, *J* = 1.42 Hz, 3H), 1.46 (q, *J* = 1.46 Hz, 3H), 1.01 (t, *J* = 7.54 Hz, 3H). $^{13}$C NMR (101 MHz, Chloroform-*d, Z*-isomer) δ 147.2, 136.2, 129.1 (q, *J* = 32.60 Hz), 128.5, 126.1, 125.2 (q, *J* = 1.51 Hz), 124.5 (q, *J* = 271.80 Hz), 71.6, 27.2, 18.8, 13.9, 12.3. $^{13}$C NMR (101 MHz, Chloroform-*d, E*-isomer) δ 147.2, 135.5, 129.1 (q, *J* = 32.60 Hz), 128.7, 126.0, 125.2 (q, *J* = 1.51 Hz), 124.5 (q, *J* = 271.80 Hz), 72.0, 28.1, 18.0, 12.6, 11.7. $^{19}$F

NMR (377 MHz, Chloroform-*d*) δ -62.3, -62.2. **HRMS-ESI**: Found $[M+Na]^+$ = 281.1131; $C_{14}H_{17}F_3ONa$ requires 281.1124.

**(*E/Z*)-1-(4-methoxyphenyl)-2,3,4-trimethylpent-2-en-1-ol (2e)**

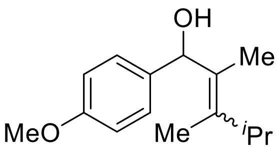


Colorless oil, 180.2 mg (77% yield). $^1$H NMR (400 MHz, Chloroform-*d*, *Z*-isomer) δ 7.28 – 7.22 (m, 2H), 6.92 – 6.83 (m, 2H), 5.90 (d, *J* = 3.61 Hz, 1H), 3.80 (s, 3H), 3.16 (hept, *J* = 6.81 Hz, 1H), 1.61 (q, *J* = 1.01 Hz, 3H), 1.49 (q, *J* = 1.03 Hz, 3H), 1.04 (d, *J* = 6.78 Hz, 3H), 1.02 (d, *J* = 6.96 Hz, 3H). $^1$H NMR (400 MHz, Chloroform-*d, E*-isomer) δ 7.29 – 7.19 (m, 2H), 6.92 – 6.83 (m, 2H), 5.77 (d, *J* = 3.83 Hz, 1H), 3.80 (s, 3H), 2.92 – 2.82 (m, 1H), 1.72 (q, *J* = 1.38 Hz, 3H), 1.52 (q, *J* = 1.38 Hz, 3H), 1.00 (d, *J* = 7.00 Hz, 3H), 0.98 (d, *J* = 6.85 Hz, 3H). $^{13}$C NMR (101 MHz, Chloroform-*d*, *E/Z* Mixture) δ 158.6, 158.5, 138.2, 137.4, 135.5, 135.3, 128.5, 128.3, 127.0, 126.8, 113.7, 113.7, 72.2, 71.0, 55.4 (2C), 30.5, 29.6, 21.4, 21.3, 20.5, 20.2, 13.1, 13.0, 11.9, 11.7. **HRMS-ESI**: Found $[M+Na]^+$ = 257.1512; $C_{15}H_{22}O_2Na$ requires 257.1512.

**(*E/Z*)-2,3,4-trimethyl-1-(p-tolyl)pent-2-en-1-ol (2f)**

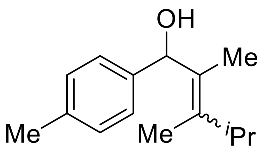


Colorless oil, 152.6 mg (70% yield). $^1$H NMR (400 MHz, Chloroform-*d*, *Z*-isomer) δ 7.22 (d, *J* = 3.34 Hz, 2H), 7.14 (d, *J* = 7.87 Hz, 2H), 5.91 (d, *J* = 3.44 Hz, 1H), 3.25 – 3.10 (m, 1H), 2.34 (s, 3H), 1.61 (q, *J* = 1.04 Hz, 3H), 1.52 (q, *J* = 1.43 Hz, 3H), 1.04 (t, *J* = 6.75 Hz, 6H). $^1$H NMR (400 MHz, Chloroform-*d*, *E*-isomer) δ 7.24 (d, *J* = 3.36 Hz, 2H), 7.14 (d, *J* = 7.87 Hz, 2H), 5.79 (d, *J* = 3.65 Hz, 1H), 2.95 – 2.80 (m, 1H), 2.34 (s, 3H), 1.74 (q, *J* = 1.38 Hz, 3H), 1.48 (q, *J* = 1.07 Hz, 3H), 1.00 (d, *J* = 6.88 Hz, 3H), 0.98 (d, *J* = 6.86 Hz, 3H). $^{13}$C NMR (101 MHz, Chloroform-*d*, *E/Z* mixture) δ 140.3, 140.2, 138.3, 137.5, 136.4, 136.4, 129.0, 129.0, 128.4, 128.3, 125.7, 125.6, 72.4, 71.3, 30.5, 29.6, 22.7, 21.4, 21.3, 21.2, 20.5, 20.2, 13.1, 13.0, 12.0, 11.7. **HRMS-ESI**: Found $[M+Na]^+$ = 241.1561; $C_{15}H_{22}ONa$ requires 241.1563.

**(*E/Z*)-1-(3-methoxyphenyl)-2,3,4-trimethylpent-2-en-1-ol (2g)**

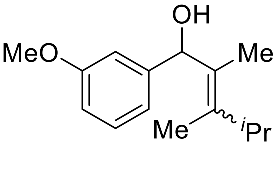


Colorless oil, 159.1 mg (68% yield). $^1$H NMR (400 MHz, Chloroform-*d*, *Z*-isomer) δ 7.26 – 6.39 (m, 4H), 5.92 (s, 1H), 3.81 (s, 3H), 3.18 (hept, *J* = 6.80 Hz, 1H), 1.61 (q, *J* = 1.10 Hz, 3H), 1.48 (d, *J* = 1.06 Hz, 3H), 1.05 (d, *J* = 6.64 Hz, 6H). $^1$H NMR (400 MHz, Chloroform-*d, E*-isomer) δ 7.26 – 6.39 (m, 4H), 5.79 (s, 1H), 3.81 (s, 3H), 2.88 (hept, *J* = 6.91 Hz, 1H), 1.75 (q, *J* = 1.38 Hz, 3H), 1.52 (q, *J* = 1.45 Hz, 3H), 1.01 (d, *J* = 6.88 Hz, 3H), 0.99 (d, *J* = 6.82 Hz, 3H). $^{13}$C NMR (101 MHz, Chloroform-*d, E*-isomer) δ 159.8, 145.1,

138.6, 129.3, 128.3, 118.2, 112.2, 111.6, 71.3, 55.3, 29.7, 21.4, 21.3, 13.1, 13.0. $^{13}$C NMR (101 MHz, Chloroform-*d*, *Z*-isomer) δ 159.8, 145.0, 137.8, 129.3, 128.2, 118.0, 112.2, 111.4, 72.4, 55.3, 30.5, 20.5, 20.2, 12.0, 11.7. **HRMS-ESI**: Found [M+Na]$^{+}$ = 257.1512; $C_{15}H_{22}O_2Na$ requires 257.1512.

***(E/Z)*-2,3,4-trimethyl-1-(m-tolyl)pent-2-en-1-ol (2h)**

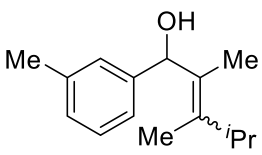


Colorless oil, 143.9 mg (66% yield). $^{1}$H NMR (400 MHz, Chloroform-*d*, *Z*-isomer) δ 7.25 – 7.03 (m, 4H), 5.92 (s, 1H), 3.20 (hept, *J* = 6.79 Hz, 1H), 2.36 (s, 3H), 1.62 (s, 3H), 1.49 (s, 3H), 1.07 (d, *J* = 2.54 Hz, 3H), 1.05 (d, *J* = 2.55 Hz, 3H). $^{1}$H NMR (400 MHz, Chloroform-*d*, *E*-isomer) δ 7.27 – 6.95 (m, 4H), 5.80 (s, 1H), 2.89 (hept, *J* = 6.92 Hz, 1H), 2.34 (s, 3H), 1.76 (q, *J* = 1.45 Hz, 3H), 1.53 (d, *J* = 1.47 Hz, 3H), 1.01 (d, *J* = 6.96 Hz, 3H), 0.99 (d, *J* = 7.01 Hz, 3H). $^{13}$C NMR (101 MHz, Chloroform-*d*, *E/Z* mixture) δ 143.3, 143.2, 138.4, 137.9, 137.9, 137.6, 128.4, 128.3, 128.2, 128.2, 127.6, 127.5, 126.5, 126.4, 122.9, 122.7, 72.5, 71.4, 30.5, 29.7, 21.7, 21.7, 21.4, 21.3, 20.5, 20.2, 13.1, 13.0, 12.0, 11.8. **HRMS-ESI**: Found [M+Na]$^{+}$ = 241.1559; $C_{15}H_{22}ONa$ requires 241.1568.

***(E/Z)*-1-(4-chlorophenyl)-2,3,4-trimethylpent-2-en-1-ol (2i)**

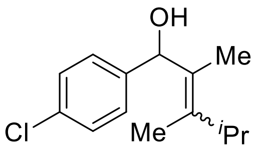


White solid, 185.6 mg (78% yield). $^{1}$H NMR (400 MHz, Chloroform-*d*, *Z*-isomer) δ 7.32 – 7.24 (m, 4H), 5.90 (s, 1H), 3.15 (hept, *J* = 6.81 Hz, 1H), 1.61 (q, *J* = 0.99 Hz, 3H), 1.44 (p, *J* = 1.16 Hz, 3H), 1.05 (d, *J* = 2.94 Hz, 3H), 1.04 (d, *J* = 2.96 Hz, 3H). $^{1}$H NMR (400 MHz, Chloroform-*d*, *E*-isomer) δ 7.32 – 7.24 (m, 4H), 5.78 (s, 1H), 2.87 (hept, *J* = 6.86 Hz, 1H), 1.74 (q, *J* = 1.37 Hz, 3H), 1.48 (q, *J* = 1.37 Hz, 3H), 1.00 (d, *J* = 6.85 Hz, 3H), 0.98 (d, *J* = 6.88 Hz, 3H). $^{13}$C NMR (101 MHz, Chloroform-*d*, *Z*-isomer) δ 141.8, 139.1, 132.5, 128.4, 128.0, 127.3, 70.8, 29.7, 21.4, 21.3, 13.0, 13.0. $^{13}$C NMR (101 MHz, Chloroform-*d*, *E*-isomer) δ 141.7, 138.2, 132.5, 128.4, 128.0, 127.1, 71.9, 30.5, 20.5, 20.2, 12.0, 11.6. **HRMS-ESI**: Found [M+Na]$^{+}$ = 261.1012; $C_{14}H_{19}ClONa$ requires 2661.1017.

***(E/Z)*-(1-(4-fluorophenyl)-2,3,4-trimethylpent-2-en-1-ol (2j)**

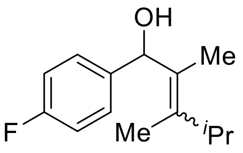


Colorless oil, 162.1 mg (73% yield). $^{1}$H NMR (400 MHz, Chloroform-*d*, *Z*-isomer) δ 7.34 – 7.27 (m, 2H), 7.03 – 6.97 (m, 2H), 5.91 (s, 1H), 3.22 – 3.09 (m, 1H), 1.61 (s, 3H), 1.45 (s, 3H), 1.05 (d, *J* = 5.92 Hz, 3H), 1.03 (d, *J* = 5.13 Hz, 3H). $^{1}$H NMR (400 MHz, Chloroform-*d*, *E*-isomer) δ 7.34 – 7.27 (m, 2H), 7.03 – 6.97 (m, 2H), 5.79 (s, 1H), 2.91 – 2.83 (m, 1H), 1.74 (q, *J* = 1.44 Hz, 3H), 1.49 (t, *J* = 1.45 Hz, 3H), 1.00 (d, *J* = 7.13 Hz, 3H), 0.98 (d, *J* = 7.56 Hz, 3H). $^{13}$C NMR (101 MHz, Chloroform-*d*, *E/Z* mixture) δ 163.1 (d, *J* = 2.22

Hz), 160.7 (d, *J* = 2.18 Hz), 138.9 (d, *J* = 2.74 Hz), 138.8, 138.8, 138.0 (d, *J* = 1.10 Hz), 128.2, 128.1, 127.4 (d, *J* = 7.84 Hz), 127.3 (d, *J* = 7.90 Hz), 115.1 (d, *J* = 2.46 Hz), 114.9 (d, *J* = 2.49 Hz), 72.0, 70.8, 30.5, 29.7, 21.4, 21.3, 20.5, 20.2, 13.0, 13.0, 12.0, 11.6. $^{19}$F NMR (377 MHz, Chloroform-d) δ -116.7, -116.7. **HRMS-ESI**: Found [M+Na]$^+$ = 245.1319; $C_{14}H_{19}FONa$ requires 245.1312.

***(E/Z)*-2,3,4-trimethyl-1-(4-(trifluoromethyl)phenyl)pent-2-en-1-ol (2k)**

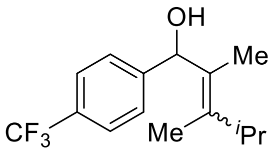


Colorless oil, 206.7 mg (76% yield). $^1$H NMR (400 MHz, Chloroform-*d*, *Z*-isomer) δ 7.62 – 7.54 (m, 2H), 7.44 (dt, *J* = 3.88, 0.80 Hz, 2H), 5.86 (s, 1H), 2.95 – 2.80 (m, 1H), 1.62 (q, *J* = 0.99 Hz, 3H), 1.43 (q, *J* = 0.98 Hz, 3H), 1.01 (d, *J* = 6.92 Hz, 3H), 0.99 (d, *J* = 6.88 Hz, 3H). $^1$H NMR (400 MHz, Chloroform-*d*, *E*-isomer) δ 7.62 – 7.54 (m, 2H), 7.46 (dt, *J* = 3.87, 0.84 Hz, 2H), 5.98 (s, 1H), 3.26 – 3.11 (m, 1H), 1.78 (q, *J* = 1.39 Hz, 3H), 1.47 (q, *J* = 1.39 Hz, 3H), 1.07 (d, *J* = 1.60 Hz, 3H), 1.06 (d, *J* = 1.53 Hz, 3H), $^{13}$C NMR (101 MHz, Chloroform-*d*, *E/Z* mixture) δ 147.1(2C), 139.5, 138.6, 128.9 (q, *J* = 32.01 Hz, 2C), 127.7, 127.6, 126.0, 125.8, 125.1 (q, *J* = 2.05 Hz, 2C), 124.1 (q, *J* = 271.46 Hz, 2C), 71.9, 70.8, 30.4, 29.7, 21.3, 21.2, 20.3, 20.0, 12.9, 12.9, 12.0, 11.5. $^{19}$F NMR (377 MHz, Chloroform-*d*) δ -62.3, -62.3. **HRMS-ESI**: Found [M+Na]$^+$ = 295.1285; $C_{15}H_{19}F_3ONa$ requires 295.1280.

***(E/Z)*-1-(2,4-difluorophenyl)-2,3,4-trimethylpent-2-en-1-ol (2l)**

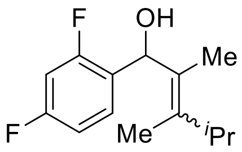


Colorless oil, 165.6 mg (69% yield). $^1$H NMR (400 MHz, Chloroform-*d*, *Z*-isomer) δ 7.54 (tddd, *J* = 8.68, 6.63, 3.91, 0.86 Hz, 1H), 6.92 – 6.82 (m, 1H), 6.73 (ddd, *J* = 10.90, 8.76, 2.52 Hz, 1H), 6.10 (s, 1H), 3.21 (hept, *J* = 6.83 Hz, 1H), 1.58 (q, *J* = 0.99 Hz, 3H), 1.49 (q, *J* = 1.00 Hz, 3H), 1.03 (d, *J* = 6.82 Hz, 3H), 0.99 (d, *J* = 6.82 Hz, 3H). $^1$H NMR (400 MHz, Chloroform-*d*, *E*-isomer) δ 7.54 (tddd, *J* = 8.68, 6.63, 3.91, 0.86 Hz, 1H), 6.92 – 6.82 (m, 1H), 6.73 (ddd, *J* = 10.90, 8.76, 2.52 Hz, 1H), 5.98 (s, 1H), 2.83 (hept, *J* = 6.84 Hz, 1H), 1.74 – 1.72 (m, 3H), 1.52 (q, *J* = 1.40 Hz, 3H), 0.99 (d, *J* = 6.82 Hz, 3H), 0.93 (d, *J* = 6.86 Hz, 3H). $^{13}$C NMR (101 MHz, Chloroform-*d*, *E/Z* mixture) δ 163.5 – 163.1 (m,2C), 161.4 – 160.6 (m, 2C), 158.8 - 158.5 (m, 2C), 139.5, 138.8, 128.8 (dd, *J* = 9.49, 5.88 Hz), 128.6 (dd, *J* = 9.39, 6.05 Hz), 126.5 - 126.1 (m, 2C), 110.9 (t, *J* = 3.48 Hz), 110.7 (t, *J* = 3.50 Hz), 103.6 (t, *J* = 25.50 Hz, 2C), 67.6 (d, *J* = 2.15 Hz), 66.3 (d, *J* = 2.21 Hz), 30.6, 29.7, 21.5, 20.7 (d, *J* = 1.62 Hz), 20.3 (d, *J* = 4.43 Hz), 13.1 (d, *J* = 2.66 Hz), 11.7 (d, *J* = 2.93 Hz), 11.6. $^{19}$F NMR (377 MHz, Chloroform-*d*, *E/Z* mixture) δ -112.9 (dd, *J* = 49.62, 7.26 Hz), -113.4 (dd, *J* = 41.37, 7.18 Hz). **HRMS-ESI**: Found [M+Na]$^+$ = 263.1220; $C_{14}H_{18}F_2ONa$ requires 263.1218.

**(E/Z)-2,3,4-trimethyl-1-(naphthalen-2-yl)pent-2-en-1-ol (2m)**

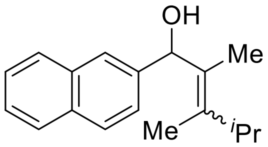


Yellow oil, 167.6 mg (66% yield). $^1$H NMR (400 MHz, Chloroform-*d*, *Z*-isomer) δ 7.92 – 7.71 (m, 4H), 7.46 (tt, *J* = 8.76, 6.06 Hz, 2H), 7.39 – 7.30 (m, 1H), 6.11 (s, 1H), 3.28 (hept, *J* = 6.81 Hz, 1H), 1.65 (s, 3H), 1.50 (s, 3H), 1.10 (t, *J* = 6.35 Hz, 6H). $^1$H NMR (400 MHz, Chloroform-*d*, *E*-isomer) δ 7.92 – 7.71 (m, 4H), 7.46 (tt, *J* = 8.76, 6.06 Hz, 2H), 7.39 – 7.30 (m, 1H), 5.99 (s, 1H), 2.91 (hept, *J* = 6.85 Hz, 1H), 1.83 (q, *J* = 1.45 Hz, 3H), 1.54 (q, *J* = 1.46 Hz, 3H), 1.03 (t, *J* = 6.75 Hz, 6H). $^{13}$C NMR (101 MHz, Chloroform-*d*, *E/Z* mixture) δ 140.8, 140.7, 139.0, 138.1, 133.5, 132.6, 132.6, 129.9, 128.2, 128.2, 127.9, 127.9, 127.7, 127.7, 126.6, 126.5, 126.1, 126.1, 125.7, 125.7, 124.5, 124.3, 124.1, 124.0, 72.6, 71.5, 30.5, 29.8, 21.5, 21.4, 20.5, 20.2, 13.2, 13.1, 12.1, 11.8. **HRMS-ESI**: Found [M+Na]$^+$ = 277.1562; $C_{18}H_{22}ONa$ requires 277.1563.

**(E/Z)- 3,4,5-trimethyl-1-phenylhex-3-en-2-ol (3a)**

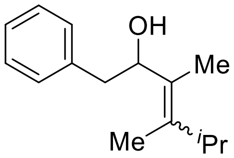


Colorless oil, 174.4 mg (80% yield). $^1$H NMR (400 MHz, Chloroform-*d*, *Z*-isomer) δ 7.33 – 7.26 (m, 2H), 7.23 – 7.15 (m, 3H), 4.94 (t, *J* = 6.51 Hz, 1H), 2.95 – 2.67 (m, 3H), 1.71 (s, 3H), 1.51 (s, 3H), 0.92 (d, *J* = 6.78 Hz, 3H), 0.66 (d, *J* = 6.78 Hz, 3H). $^1$H NMR (400 MHz, Chloroform-*d*, *E*-isomer) δ 7.33 – 7.26 (m, 2H), 7.23 – 7.15 (m, 3H), 4.81 (t, *J* = 7.48 Hz, 1H), 2.95 – 2.67 (m, 3H), 1.73 (q, *J* = 1.48 Hz, 3H), 1.33 (q, *J* = 1.41 Hz, 3H), 0.92 (d, *J* = 6.78 Hz, 3H), 0.83 (d, *J* = 6.81 Hz, 3H). $^{13}$C NMR (101 MHz, Chloroform-*d*, *Z*-isomer) δ 138.8, 137.5, 129.5, 128.6, 127.6, 126.5, 71.7, 42.4, 29.3, 21.1, 20.8, 12.9, 12.6. $^{13}$C NMR (101 MHz, Chloroform-*d*, *E*-isomer) δ 138.7, 136.8, 129.6, 128.4, 127.1, 126.4, 72.7, 41.9, 30.4, 20.3, 20.1, 11.3, 11.2. **HRMS-ESI**: Found [M+Na]$^+$ = 241.1561; $C_{15}H_{22}ONa$ requires 241.1563.

**(E/Z)- 3,4,5-trimethylhex-3-en-2-ol (E/Z)-4,5,6-trimethylhept-4-en-3-ol (3b)**

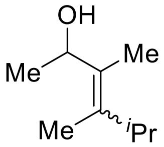


Colorless oil, 92.3 mg (65% yield). $^1$H NMR (400 MHz, Chloroform-*d*, *Z*-isomer) δ 4.97 (q, *J* = 6.47 Hz, 1H), 2.96 (hept, *J* = 6.90 Hz, 1H), 1.62 (d, *J* = 1.20 Hz, 3H), 1.53 (d, *J* = 1.24 Hz, 3H), 1.21 (d, *J* = 5.88, 3H), 0.97 (d, *J* = 6.84 Hz, 3H), 0.93 (d, *J* = 6.64 Hz, 3H). $^1$H NMR (400 MHz, Chloroform-*d*, *E*-isomer) δ 4.84 (q, *J* = 6.45 Hz, 1H), 2.82 (hept, *J* = 6.85 Hz, 1H), 1.65 (q, *J* = 1.41 Hz, 3H), 1.56 (q, *J* = 1.43 Hz, 3H), 1.20 (d, *J* = 5.32 Hz, 3H), 0.94 (d, *J* = 6.33 Hz, 3H), 0.92 (d, *J* = 6.83 Hz, 3H). $^{13}$C NMR (101 MHz, Chloroform-*d*, *Z*-isomer) δ 136.0, 129.8, 66.2, 29.1, 21.8, 21.3, 21.2, 12.9, 12.0. $^{13}$C NMR (101 MHz, Chloroform-*d*, *E*-isomer) δ 135.1, 129.4, 67.5, 30.3, 21.1, 20.4, 20.2, 11.3, 10.7. **HRMS-ESI**: Found [M+Na]$^+$ = 165.1267; $C_9H_{18}ONa$ requires 165.1250.

***(E/Z)*-4,5,6-trimethylhept-4-en-3-ol (3c)**

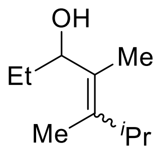

Colorless oil, 110.8 mg (71% yield).$^{1}$H NMR (400 MHz, Chloroform-*d*, *Z*-isomer) δ 4.65 (t, *J* = 7.14 Hz, 1H), 2.98 (hept, *J* = 6.80 Hz, 1H), 1.69 – 1.63 (m, 2H), 1.59 (q, *J* = 1.04 Hz, 3H), 1.55 (q, *J* = 1.01 Hz, 3H), 0.96 (t, *J* = 6.82 Hz, 6H), 0.87 – 0.84 (m, 3H). $^{1}$H NMR (400 MHz, Chloroform-*d*, *E*-isomer) δ 4.55 (t, *J* = 7.17 Hz, 1H), 2.85 (hept, *J* = 6.92 Hz, 1H), 1.62 (q, *J* = 1.39 Hz, 3H), 1.57 (q, *J* = 1.41 Hz, 3H), 1.52 – 1.43 (m, 2H), 0.94 (d, *J* = 0.94 Hz, 3H), 0.92 (d, *J* = 0.85 Hz, 3H), 0.82 (d, *J* = 4.58 Hz, 3H). $^{13}$C NMR (101 MHz, Chloroform-*d*, *Z*-isomer) δ 137.5, 128.4, 71.8, 29.1, 28.2, 21.3, 20.1, 12.9, 11.6, 10.3. $^{13}$C NMR (101 MHz, Chloroform-*d*, *E*-isomer) δ 136.8, 127.9, 72.9, 30.5, 28.0, 21.3, 20.6, 12.2, 10.8, 10.6. **HRMS-ESI**: Found $[M+Na]^+$ = 179.1421; $C_{10}H_{20}ONa$ requires 179.1406.

***(E/Z)*- 5,6,7-trimethyloct-5-en-4-ol (3d)**

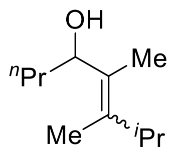

Colorless oil, 105.4 mg (62% yield).$^{1}$H NMR (400 MHz, Chloroform-*d*, *Z*-isomer) δ 4.74 (t, *J* = 6.86 Hz, 1H), 2.98 (hept, *J* = 6.84 Hz, 1H), 1.59 (s, 3H), 1.54 (s, 3H), 1.47 – 1.18 (m, 4H), 0.98 – 0.89 (m, 9H). $^{1}$H NMR (400 MHz, Chloroform-*d*, *E*-isomer) δ 4.63 (t, *J* = 6.97 Hz, 1H), 2.84 (hept, *J* = 6.84 Hz, 1H), 1.62 (q, *J* = 1.50 Hz, 3H), 1.56 (q, *J* = 1.40 Hz, 3H), 1.47 – 1.18 (m, 4H), 0.98 – 0.89 (m, 9H). $^{13}$C NMR (101 MHz, Chloroform-*d*, *Z*-isomer) δ 137.1, 128.8, 70.1, 37.6, 29.1, 21.3, 21.3, 19.4, 14.3, 12.9, 12.3. $^{13}$C NMR (101 MHz, Chloroform-*d*, *E*-isomer) δ 136.3, 128.3, 71.2, 37.4, 30.4, 20.6, 20.2, 19.2, 14.3, 11.5, 10.9. **HRMS-ESI**: Found $[M+Na]^+$ = 193.1570; $C_{11}H_{22}ONa$ requires 193.1563.

***(E/Z)*-2,3,4-trimethylnon-3-en-5-ol (3e)**

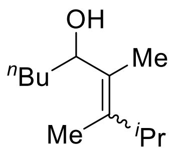

Colorless oil, 138.0 mg (75% yield). $^{1}$H NMR (400 MHz, Chloroform-*d*, *Z*-isomer) δ 4.72 (t, *J* = 7.02 Hz, 1H), 2.98 (hept, *J* = 6.80 Hz, 1H), 1.59 (q, *J* = 1.04 Hz, 3H), 1.54 (q, *J* = 1.05 Hz, 3H), 1.50 – 1.12 (m, 6H), 1.00 – 0.85 (m, 9H). $^{1}$H NMR (400 MHz, Chloroform-*d*, *E*-isomer) δ 4.62 (t, *J* = 7.04 Hz, 1H), 2.84 (hept, *J* = 6.87 Hz, 1H), 1.62 (q, *J* = 1.45 Hz, 3H), 1.56 (q, *J* = 1.38 Hz, 3H), 1.50 – 1.12 (m, 6H), 1.00 – 0.85 (m, 9H). $^{13}$C NMR (101 MHz, Chloroform-*d*, *Z*-isomer) δ 137.2, 128.8, 70.3, 35.1, 29.1, 28.4, 22.9, 21.3, 21.3, 14.2, 12.9, 12.3. $^{13}$C NMR (101 MHz, Chloroform-*d*, *E*-isomer) δ 136.4, 128.4, 71.5, 34.8, 30.4, 28.2, 22.9, 20.6, 20.2, 14.2, 11.5, 10.9. **HRMS-ESI**: Found $[M+Na]^+$ = 207.1713; $C_{12}H_{24}ONa$ requires 207.1719.

***(E/Z)*-2,5,6,7-tetramethyloct-5-en-4-ol (3f)**

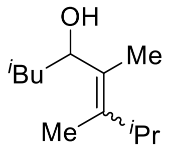

Colorless oil, 119.6 mg (65% yield). $^{1}H$ NMR (400 MHz, Chloroform-*d*, *Z*-isomer) δ 4.83 (dd, *J* = 7.78, 5.87 Hz, 1H), 2.99 (hept, *J* = 6.82 Hz, 1H), 1.60 (s, 3H), 1.54 (s, 3H), 1.25 (dq, *J* = 13.36, 6.71 Hz, 2H), 1.03 – 0.83 (m, 14H). $^{1}H$ NMR (400 MHz, Chloroform-*d*, *E*-isomer) δ 4.70 (dd, *J* = 7.82, 6.07 Hz, 1H), 2.83 (hept, *J* = 6.96 Hz, 1H), 1.62 (s, 3H), 1.57 (s, 3H), 1.25 (dq, *J* = 13.36, 6.71 Hz, 2H), 1.03 – 0.83 (m, 14H). $^{13}C$ NMR (101 MHz, Chloroform-*d*, *Z*-isomer) δ 136.8, 129.1, 68.4, 44.7, 29.2, 25.0, 23.5, 22.7, 21.3, 20.5, 12.4, 11.4. $^{13}C$ NMR (101 MHz, Chloroform-*d*, *E*-isomer) δ 135.9, 128.7, 69.7, 44.2, 30.4, 25.0, 23.5, 22.7, 21.2, 20.2, 12.9, 11.1. **HRMS-ESI**: Found $[M+Na]^+$ = 207.1715; $C_{12}H_{24}ONa$ requires 207.1719.

***(E/Z)*-2,3,4-trimethyldec-3-en-5-ol (3g)**

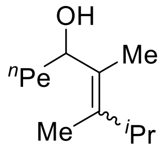

Colorless oil, 132.7 mg (67% yield). $^{1}H$ NMR (400 MHz, Chloroform-*d*, *Z*-isomer) δ 4.74 (t, *J* = 6.99 Hz, 1H), 2.99 (hept, *J* = 6.81 Hz, 1H), 1.86 – 1.69 (m, 1H), 1.61 (s, 3H), 1.56 (s, 3H), 1.51 – 1.39 (m, 2H), 1.37 – 1.26 (m, 5H), 1.05 – 0.86 (m, 9H). $^{1}H$ NMR (400 MHz, Chloroform-*d*, *Z*-isomer) δ 4.64 (t, *J* = 7.05 Hz, 1H), 2.86 (hept, *J* = 6.94 Hz, 1H), 1.86 – 1.69 (m, 1H), 1.64 (s, 3H), 1.58 (s, 3H), 1.51 – 1.39 (m, 2H), 1.37 – 1.26 (m, 5H), 1.05 – 0.86 (m, 9H). $^{13}C$ NMR (101 MHz, Chloroform-*d*, *Z*-isomer) δ 137.2, 128.7, 70.3, 35.3, 32.0, 29.1, 25.9, 22.8, 21.3, 21.3, 14.2, 12.9, 12.3. $^{13}C$ NMR (101 MHz, Chloroform-*d*, *E*-isomer) δ 136.4, 128.3, 71.5, 37.6, 35.1, 32.0, 30.4, 25.6, 20.6, 20.1, 14.2, 11.5, 10.9. **HRMS-ESI**: Found $[M+Na]^+$ = 221.1871; $C_{13}H_{26}ONa$ requires 221.1876.

## 3. General procedure for isomerization-hydrogenation of allylic alcohol

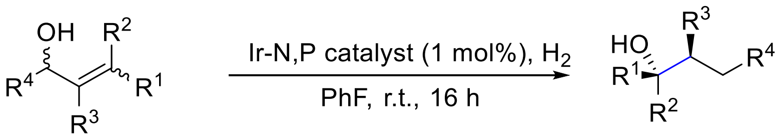


Scheme S2. Asymmetric hydrogenation using iridium catalysts.

A vial was charged with the substrate (0.15 mmol) and the Ir-complex (1 mol%). Dry PhF (3.0 mL) was added (so that the concentration of the substrate was 0.05 M) and the vial was placed in a high-pressure hydrogenation apparatus. The reactor was purged three times with Ar gas, then purged three times with $H_2$ gas and finally filled with $H_2$ gas (1 or 5 bar). The reaction was stirred at room temperature for 16 hours before the $H_2$ pressure was released and the solvent was removed *in vacuo*. The crude product was filtered through on a short plug of silica (1:1 pentane/diethyl ether). Conversion was determined by $^{1}H$ NMR spectroscopy and *ee* and/or *d.r.* values were determined by SFC or GCMS using a chiral stationary phase. Racemic

samples were prepared following the same procedure and employing the corresponding racemic iridium catalysts.

**Characterization of hydrogenated products**

**(*S*)-2,3-dimethyl-4-phenylbutan-2-ol (4a)**

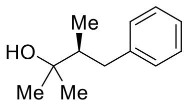


Colorless oil, 26.2 mg (98% yield). $^1$H NMR (400 MHz, Chloroform-*d*) δ 7.32 – 7.26 (m, 2H), 7.20 (dd, *J* = 10.5, 4.6 Hz, 3H), 3.09 (dd, *J* = 13.1, 3.0 Hz, 1H), 2.15 (dd, *J* = 13.1, 11.2 Hz, 1H), 1.81 – 1.71 (m, 1H), 1.27 (s, 3H), 1.27 (s, 3H), 0.82 (d, *J* = 6.8 Hz, 3H). $^{13}$C NMR (101 MHz, Chloroform-*d*) δ 141.9, 129.1, 128.2, 125.7, 73.3, 46.6, 38.0, 27.7, 26.1, 14.1. **HRMS-ESI**: Found [M+Na]$^+$ = 201.1251; $C_{12}H_{18}ONa$ requires 201.1250.

**(*S*)-4-(4-fluorophenyl)-2,3-dimethylbutan-2-ol (4b)**

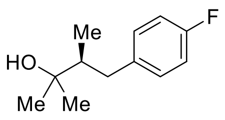


Colorless oil, 29.1 mg (99% yield).$^1$H NMR (400 MHz, Chloroform-*d*) δ 7.17 – 7.10 (m, 2H), 7.03 – 6.94 (m, 2H), 3.08 (dd, *J* = 13.2, 2.8 Hz, 1H), 2.14 (dd, *J* = 13.2, 11.2 Hz, 1H), 1.77 – 1.67 (m, 1H), 1.36 (s, 1H), 1.29 (s, 3H), 1.27 (s, 3H), 0.82 (d, *J* = 6.8 Hz, 3H). $^{13}$C NMR (101 MHz, Chloroform-*d*) δ 161.2 (d, *J* = 243.16 Hz), 137.4 (d, *J* = 3.21 Hz), 130.3 (d, *J* = 7.71 Hz), 114.9 (d, *J* = 21.04 Hz), 73.2, 46.7, 37.1, 27.8, 26.0, 14.0. $^{19}$F NMR (377 MHz, Chloroform-*d*) δ -118.0. **HRMS-ESI**: Found [M+Na]$^+$ = 219.1157; $C_{12}H_{17}ONa$ requires 219.1156.

**(*S*)-4-(4-chlorophenyl)-2,3-dimethylbutan-2-ol (4c)**

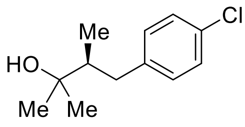


White solid, 29.6 mg (93% yield). $^1$H NMR (400 MHz, Chloroform-*d*) δ 7.28 (d, *J* = 1.8 Hz, 1H), 7.27 – 7.25 (m, 1H), 7.15 – 7.10 (m, 2H), 3.10 (s, 1H), 2.14 (s, 1H), 1.77 – 1.67 (m, 1H), 1.44 (s, 1H), 1.29 (d, *J* = 1.6 Hz, 3H), 1.27 (d, *J* = 1.6 Hz, 3H), 0.81 (dd, *J* = 6.8, 1.4 Hz, 3H. $^{13}$C NMR (101 MHz, Chloroform-*d*) δ 140.4, 131.4, 130.4, 128.3, 73.21, 46.6, 37.3, 27.9, 26.0, 14.1. **HRMS-ESI**: Found [M+Na]$^+$ = 235.0862; $C_{12}H_{17}ClONa$ requires 235.0860.

**(*S*)-2,3-dimethyl-4-(4-(trifluoromethyl)phenyl)butan-2-ol (4e)**

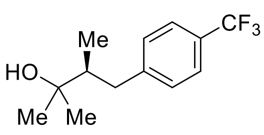


Colorless oil, 33.2 mg (90% yield). $^1$H NMR (400 MHz, Chloroform-*d*) δ 7.53 (d, *J* = 8.0 Hz, 2H), 7.28 (d, *J* = 8.1 Hz, 2H), 3.16 (dd, *J* = 13.1, 2.4 Hz, 1H), 2.21 (dd, *J* = 13.1, 11.3 Hz, 1H), 1.80 – 1.70 (m, 1H), 1.56 (s, 1H), 1.28 (s, 3H), 1.26 (s, 3H), 0.80 (d, *J* = 6.8 Hz, 3H. $^{13}$C NMR (101 MHz, Chloroform-*d*) δ 146.2, 129.4, 128.1 (d, *J* = 32.32 Hz), 125.1 (q, *J* = 3.79 Hz),

124.4 (q, *J* = 271.70 Hz), 73.2, 46.5, 37.8, 28.0, 25.9, 14.1. $^{19}$F NMR (377 MHz, Chloroform-*d*) δ -62.3. **HRMS-ESI**: Found [M+Na]$^+$ = 269.1126; $C_{13}H_{17}F_3ONa$ requires 269.1124.

**(*S*)-2,3-dimethyl-4-(p-tolyl)butan-2-ol (4d)**

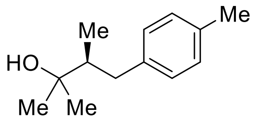


Colorless oil, 26.0 mg (90% yield). $^1$H NMR (400 MHz, Chloroform-*d*) δ 7.10 (d, *J* = 8.1 Hz, 2H), 7.08 – 7.04 (m, 2H), 3.04 (dd, *J* = 13.2, 3.0 Hz, 1H), 2.32 (s, 3H), 2.11 (dd, *J* = 13.1, 11.1 Hz, 1H), 1.78 – 1.67 (m, 1H), 1.36 (s, 1H), 1.26 (d, *J* = 1.6 Hz, 6H), 0.81 (d, *J* = 6.8 Hz, 3H. $^{13}$C NMR (101 MHz, Chloroform-*d*) δ 138.7, 135.1, 129.0, 128.9, 73.3, 46.7, 37.6, 27.7, 26.1, 21.0, 14.2. **HRMS-ESI**: Found [M+Na]$^+$ = 215.1406; $C_{13}H_{20}ONa$ requires 215.1406.

**(*S*)-4-(4-methoxyphenyl)-2,3-dimethylbutan-2-ol (4f)**

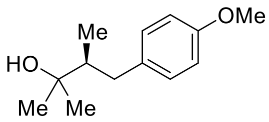


Colorless oil, 27.5 mg (88% yield). $^1$H NMR (400 MHz, Chloroform-*d*) δ 7.09 (d, *J* = 8.5 Hz, 2H), 6.85 – 6.81 (m, 2H), 3.79 (s, 3H), 3.01 (dd, *J* = 13.3, 3.0 Hz, 1H), 2.09 (dd, *J* = 13.2, 11.1 Hz, 1H), 1.73 – 1.67 (m, 1H), 1.25 (d, *J* = 2.1 Hz, 6H), 0.80 (d, *J* = 6.8 Hz, 3H). $^{13}$C NMR (101 MHz, Chloroform-*d*) δ 157.7, 133.9, 130.0, 113.7, 73.4, 55.2, 46.7, 37.1, 27.7, 26.1, 14.1. **HRMS-ESI**: Found [M+Na]$^+$ = 231.1352; $C_{13}H_{20}O_2Na$ requires 231.1356.

**(*S*)-2,3-dimethyl-4-(o-tolyl)butan-2-ol (4g)**

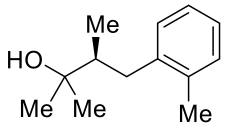


Colorless oil, 24.2 mg (84% yield). $^1$H NMR (400 MHz, Chloroform-*d*) δ 7.16 – 7.08 (m, 4H), 3.07 (dd, *J* = 13.2, 2.8 Hz, 1H), 2.34 (s, 3H), 2.21 (dd, *J* = 13.2, 11.4 Hz, 1H), 1.80 – 1.70 (m, 1H), 1.36 (s, 1H), 1.29 (d, *J* = 1.4 Hz, 6H), 0.83 (d, *J* = 6.8 Hz, 3H). $^{13}$C NMR (101 MHz, Chloroform-*d*) δ 139.8, 136.3, 130.3, 130.2, 125.8, 125.6, 73.5, 44.9, 35.1, 27.6, 26.0, 19.6, 14.0. **HRMS-ESI**: Found [M+Na]$^+$ = 215.1403; $C_{13}H_{20}ONa$ requires 215.1406.

**(*S*)-2,3-dimethyl-4-(naphthalen-2-yl)butan-2-ol (4h)**

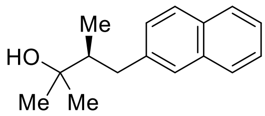


Colorless oil, 29.4 mg (86% yield). $^1$H NMR (400 MHz, Chloroform-*d*) δ 7.83 – 7.80 (m, 1H), 7.78 (d, *J* = 8.1 Hz, 2H), 7.61 (s, 1H), 7.48 – 7.40 (m, 2H), 7.33 (dd, *J* = 8.4, 1.7 Hz, 1H), 3.25 (dd, *J* = 13.1, 3.0 Hz, 1H), 2.32 (dd, *J* = 13.1, 11.2 Hz, 1H), 1.92 – 1.82 (m, 1H), 1.39 (s, 1H), 1.30 (s, 6H), 0.84 (d, *J* = 6.8 Hz, 3H. $^{13}$C NMR (101 MHz, Chloroform-*d*) δ 139.4, 133.5, 132.0, 127.8, 127.8, 127.6, 127.4, 127.3, 125.9, 125.1, 73.4, 46.5, 38.2, 27.8, 26.1, 14.2. **HRMS-ESI**: Found [M+Na]$^+$ = 251.1404; $C_{16}H_{20}ONa$ requires 251.1406.

**(*2S,3R*)-2,3,4-trimethyl-1-phenylpentan-3-ol (5a)**

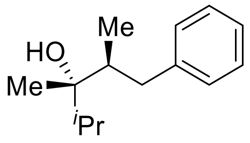

Colorless oil, 27.8 mg (90% yield). $^1$H NMR (400 MHz, Chloroform-*d*) δ 7.31 – 7.26 (m, 2H), 7.20 (dd, *J* = 5.8, 1.6 Hz, 1H), 7.17 (dd, *J* = 5.0, 3.2 Hz, 2H), 2.97 (dd, *J* = 13.2, 2.8 Hz, 1H), 2.17 (dd, *J* = 13.2, 11.3 Hz, 1H), 2.01 – 1.91 (m, 2H), 1.15 (s, 3H), 1.02 (d, *J* = 6.8 Hz, 3H), 0.94 (d, *J* = 6.8 Hz, 3H), 0.81 (d, *J* = 6.7 Hz, 3H). $^{13}$C NMR (101 MHz, Chloroform-*d*) δ 141.9, 129.1, 128.2, 125.7, 76.1, 41.9, 37.9, 34.2, 19.4, 17.6, 16.6, 12.7. **HRMS-ESI**: Found [M+Na]$^+$ = 229.1564; $C_{14}H_{22}ONa$ requires 229.1563.

**(*2S,3R*)-2,3-dimethyl-1-(4-(trifluoromethyl)phenyl)pentan-3-ol (5b)**

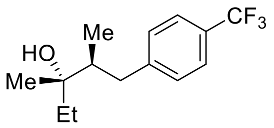

Colorless oil, 35.1 mg (90% yield). $^1$H NMR (400 MHz, Chloroform-*d,* major diastereoisomer) δ 7.55 – 7.48 (m, 2H), 7.32 – 7.26 (m, 2H), 3.05 (dd, *J* = 12.71, 2.56 Hz, 1H), 2.23 (dd, *J* = 13.34, 2.65 Hz, 1H), 1.88 – 1.72 (m, 1H), 1.60 (dq, *J* = 8.95, 7.48 Hz, 2H), 1.21 (s, 3H), 0.98 (t, *J* = 7.45 Hz, 3H), 0.80 (d, *J* = 6.83 Hz, 3H). $^1$H NMR (400 MHz, Chloroform-*d,* minor diastereoisomer) δ 7.55 – 7.48 (m, 2H), 7.32 – 7.26 (m, 2H), 3.19 (dd, *J* = 12.96, 2.83 Hz, 1H), 2.23 (dd, *J* = 13.34, 2.65 Hz, 1H), 1.88 – 1.72 (m, 1H), 1.60 (dq, *J* = 8.95, 7.48 Hz, 2H), 1.17 (s, 3H), 0.95 (t, *J* = 7.44 Hz, 3H), 0.76 (d, *J* = 6.82 Hz, 3H).$^{13}$C NMR (101 MHz, Chloroform-*d*) δ 146.1, 129.4, 128.3 (q, *J* = 32.25 Hz), 125.1 (q, *J* = 3.64 Hz), 123.0 (q, *J* = 273.10 Hz), 74.7, 44.3, 37.7, 31.6, 23.9, 13.3, 7.8. $^{13}$C NMR (101 MHz, Chloroform-*d*) δ 146.5, 129.4, 128.3 (q, *J* = 32.25 Hz), 125.1 (q, *J* = 3.64 Hz), 123.0 (q, *J* = 273.10 Hz), 74.8, 44.0, 37.3, 32.9, 22.7, 13.9, 7.5. $^{19}$F NMR (377 MHz, Chloroform-*d*) δ -62.3, -62.3. **HRMS-ESI**: Found [M+Na]$^+$ = 283.1295; $C_{14}H_{19}F_3ONa$ requires 283.1280.

**(*2R,3S*)-2-cyclohexyl-3-methyl-4-phenylbutan-2-ol (5d)**

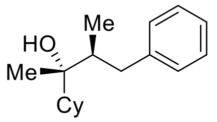

Colorless oil, 31.4 mg (85% yield). $^1$H NMR (400 MHz, Chloroform-*d*) δ 7.32 – 7.24 (m, 2H), 7.21 – 7.12 (m, 3H), 2.96 (dd, *J* = 13.31, 2.90 Hz, 1H), 2.19 (dd, *J* = 13.31, 11.10 Hz, 1H), 2.02 – 1.42 (m, 6H), 1.31 – 1.00 (m, 9H), 0.79 (d, *J* = 6.78 Hz, 3H).$^{13}$C NMR (101 MHz, Chloroform-*d*) δ 142.0, 129.1, 128.2, 125.7, 76.0, 44.9, 41.4, 37.7, 27.7, 26.9, 26.8, 26.7, 26.4, 20.1, 12.7. **HRMS-ESI**: Found [M+Na]$^+$ = 269.1881; $C_{17}H_{26}ONa$ requires 269.1881.

**(*2S,3R*)-1-(4-methoxyphenyl)-2,3,4-trimethylpentan-3-ol (5e)**

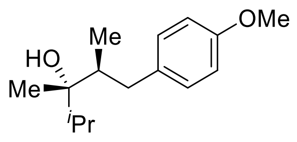

Colorless oil, 34.0 mg (96% yield). $^{1}$H NMR (400 MHz, Chloroform-*d*) δ 7.08 (d, *J* = 8.4 Hz, 2H), 6.85 – 6.81 (m, 2H), 3.79 (s, 3H), 2.90 (dd, *J* = 13.3, 2.9 Hz, 1H), 2.11 (dd, *J* = 13.3, 11.3 Hz, 1H), 1.96 (p, *J* = 6.7 Hz, 1H), 1.91 – 1.83 (m, 1H), 1.14 (s, 3H), 1.01 (d, *J* = 6.8 Hz, 3H), 0.93 (d, *J* = 6.8 Hz, 3H), 0.82 – 0.78 (m, 3H). $^{13}$C NMR (101 MHz, Chloroform-*d*) δ 157.7, 133.8, 129.9, 113.6, 76.1, 55.2, 42.0, 36.9, 34.2, 19.3, 17.6, 16.5, 12.6. **HRMS-ESI**: Found [M+Na]$^{+}$ = 259.1671; $C_{15}H_{24}O_{2}Na$ requires 259.1669.

**(*2S,3R*)-2,3,4-trimethyl-1-(p-tolyl)pentan-3-ol (5f)**

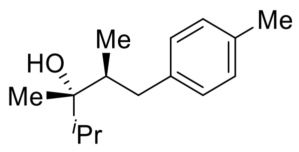

Colorless oil, 28.1 mg (85% yield). $^{1}$H NMR (400 MHz, Chloroform-*d*) δ 7.09 (d, *J* = 8.0 Hz, 2H), 7.05 (d, *J* = 8.1 Hz, 2H), 2.92 (dd, *J* = 13.3, 2.9 Hz, 1H), 2.32 (s, 3H), 2.12 (dd, *J* = 13.2, 11.2 Hz, 1H), 1.96 (dt, *J* = 12.7, 6.4 Hz, 1H), 1.93 – 1.84 (m, 1H), 1.14 (s, 3H), 1.01 (d, *J* = 6.8 Hz, 3H), 0.93 (d, *J* = 6.8 Hz, 3H), 0.80 (d, *J* = 6.7 Hz, 3H). $^{13}$C NMR (101 MHz, Chloroform-*d*) δ 138.7, 135.1, 129.0, 128.9, 76.2, 42.0, 37.4, 34.2, 21.0, 19.4, 17.6, 16.6, 12.7. **HRMS-ESI**: Found [M+Na]$^{+}$ = 243.1719; $C_{15}H_{24}ONa$ requires 243.1719.

**(*2S,3R*)-1-(3-methoxyphenyl)-2,3,4-trimethylpentan-3-ol (5g)**

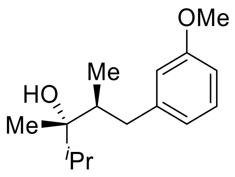

Colorless oil, 28.3 mg (80% yield). $^{1}$H NMR (400 MHz, Chloroform-*d*) δ 7.20 (t, *J* = 7.8 Hz, 1H), 6.78 – 6.71 (m, 3H), 3.80 (s, 3H), 2.94 (dd, *J* = 13.2, 3.0 Hz, 1H), 2.14 (dd, *J* = 13.2, 11.3 Hz, 1H), 2.00 – 1.89 (m, 2H), 1.14 (s, 3H), 1.01 (d, *J* = 6.8 Hz, 3H), 0.93 (d, *J* = 6.8 Hz, 3H), 0.83 – 0.79 (m, 3H). $^{13}$C NMR (101 MHz, Chloroform-*d*) δ 159.5, 143.6, 129.1, 121.6, 115.1, 110.7, 76.1, 55.1, 41.8, 37.9, 34.2, 19.4, 17.6, 16.6, 12.7. **HRMS-ESI**: Found [M+Na]$^{+}$ = 259.1667; $C_{15}H_{24}O_{2}Na$ requires 259.1669.

**(*2S,3R*)-2,3,4-trimethyl-1-(m-tolyl)pentan-3-ol (5h)**

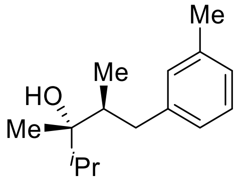

Colorless oil, 28.4 mg (86% yield). $^{1}$H NMR (400 MHz, Chloroform-*d*) δ 7.17 (t, *J* = 7.6 Hz, 1H), 6.98 (dd, *J* = 14.8, 7.8 Hz, 3H), 2.93 (dd, *J* = 13.2, 2.7 Hz, 1H), 2.33 (s, 3H), 2.12 (dd, *J* = 13.1, 11.3 Hz, 1H), 2.01 – 1.87 (m, 2H), 1.15 (s, 3H), 1.01 (d, *J* = 6.8 Hz, 3H), 0.93 (d, *J* = 6.8 Hz, 3H), 0.81 (d, *J* = 6.6 Hz, 3H). $^{13}$C NMR (101 MHz, Chloroform-*d*) δ 141.8, 137.8, 129.9, 128.1, 126.4, 126.2, 76.2, 41.9, 37.8, 34.2, 21.4, 19.4, 17.6, 16.6, 12.7. **HRMS-ESI**: Found [M+Na]$^{+}$ = 243.1721; $C_{15}H_{24}ONa$ requires 243.1719.

**(*2S,3R*)-1-(4-chlorophenyl)-2,3,4-trimethylpentan-3-ol (5i)**

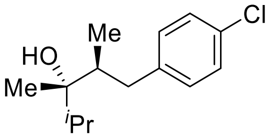


White solid, 35.6 mg (99% yield). $^{1}$H NMR (400 MHz, Chloroform-*d*) δ 7.27 – 7.26 (m, 1H), 7.24 (d, *J* = 2.0 Hz, 1H), 7.11 – 7.07 (m, 2H), 2.93 (dd, *J* = 13.3, 3.0 Hz, 1H), 2.15 (dd, *J* = 13.3, 11.3 Hz, 1H), 1.99 – 1.86 (m, 2H), 1.14 (s, 3H), 1.08 (s, 1H), 1.01 (d, *J* = 6.8 Hz, 3H), 0.93 (d, *J* = 6.8 Hz, 3H), 0.79 (dd, *J* = 6.7, 0.6 Hz, 3H). $^{13}$C NMR (101 MHz, Chloroform-*d*) δ 140.3, 131.4, 130.4, 128.3, 76.0, 41.9, 37.2, 34.2, 19.4, 17.6, 16.5, 12.6; **HRMS-ESI**: Found [M+Na]$^{+}$ = 263.1173; $C_{14}H_{21}ClONa$ requires 263.1173.

**(*2S,3R*)-1-(4-fluorophenyl)-2,3,4-trimethylpentan-3-ol (5j)**

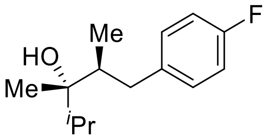


Colorless oil, 30.9 mg (92% yield). $^{1}$H NMR (400 MHz, Chloroform-*d*) δ 7.11 (d, *J* = 8.4 Hz, 2H), 6.96 (d, *J* = 8.0 Hz, 2H), 2.93 (dd, *J* = 13.4, 2.6 Hz, 1H), 2.15 (dd, *J* = 13.2, 11.4 Hz, 1H), 1.95 (dt, *J* = 13.6, 6.8 Hz, 1H), 1.88 (tdd, *J* = 9.6, 6.7, 3.0 Hz, 1H), 1.14 (s, 3H), 1.01 (d, *J* = 6.8 Hz, 3H), 0.93 (d, *J* = 6.8 Hz, 3H), 0.79 (d, *J* = 6.7 Hz, 3H). $^{13}$C NMR (101 MHz, Chloroform-*d*) δ 161.2 (d, *J* = 243.25 Hz), 137.4 (d, *J* = 3.24 Hz), 130.3 (d, *J* = 7.73 Hz), 114.9 (d, *J* = 21.06 Hz), 76.0, 42.0, 37.0, 34.2, 19.4, 17.6, 16.5, 12.6. $^{19}$F NMR (377 MHz, Chloroform-*d*) δ -118.0. **HRMS-ESI**: Found [M+Na]$^{+}$ = 247.1468; $C_{14}H_{21}FONa$ requires 247.1469.

**(*2S,3R*)-2,3,4-trimethyl-1-(4-(trifluoromethyl)phenyl)pentan-3-ol (5k)**

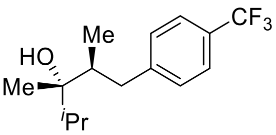


Colorless oil, 36.6 mg (89% yield). $^{1}$H NMR (400 MHz, Chloroform-*d*) δ 7.53 (d, *J* = 7.9 Hz, 2H), 7.27 (d, *J* = 8.9 Hz, 2H), 3.01 (dd, *J* = 13.1, 2.3 Hz, 1H), 2.26 (dd, *J* = 13.3, 11.4 Hz, 1H), 2.00 – 1.90 (m, 2H), 1.16 (s, 3H), 1.02 (d, *J* = 6.8 Hz, 3H), 0.94 (d, *J* = 6.8 Hz, 3H), 0.79 (d, *J* = 6.7 Hz, 3H). $^{13}$C NMR (101 MHz, Chloroform-*d*) δ 146.1, 129.4, 128.1 (q, *J* = 32.38 Hz), 125.1 (q, *J* = 3.81 Hz), 124.4 (q, *J* = 271.95 Hz), 76.0, 41.8, 37.7, 34.3, 19.4, 17.6, 16.5, 12.6. $^{19}$F NMR (377 MHz, Chloroform-*d*) δ -62.3. **HRMS-ESI**: Found [M+Na]$^{+}$ = 297.1438; $C_{15}H_{21}F_3ONa$ requires 297.1437.

**(*2S,3R*)-1-(2,4-difluorophenyl)-2,3,4-trimethylpentan-3-ol (5l)**

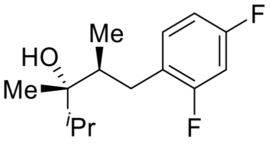


Colorless oil, 29.0 mg (80% yield). $^{1}$H NMR (400 MHz, Chloroform-*d*) δ 7.10 (td, *J* = 8.5, 6.8 Hz, 1H), 6.82 – 6.78 (m, 1H), 6.78 – 6.74 (m, 1H), 2.94 – 2.87 (m, 1H), 2.24 (ddd, *J* = 13.2, 11.5, 1.1 Hz, 1H),

1.99 – 1.88 (m, 2H), 1.15 (s, 3H), 1.00 (d, *J* = 6.8 Hz, 3H), 0.92 (d, *J* = 6.8 Hz, 3H), 0.79 (dd, *J* = 6.8, 0.8 Hz, 3H). $^{13}$C NMR (101 MHz, Chloroform-*d*) δ 162.4 (dd, *J* = 22.10, 12.00 Hz), 160.0 (dd, *J* = 23.71, 12.00 Hz), 131.9 (dd, *J* = 9.33, 6.83 Hz), 124.4 (dd, *J* = 16.00, 3.76 Hz), 110.7 (dd, *J* = 20.73, 3.71 Hz), 103.6 (dd, *J* = 24.80, 1.58 Hz), 76.1, 40.5, 34.3, 30.6, 19.2, 17.6, 16.4, 12.5. $^{19}$F NMR (377 MHz, Chloroform-*d*) δ -113.6 (d, *J* = 6.30 Hz), -113.9 (d, *J* = 6.92 Hz). **HRMS-ESI**: Found [M+Na]$^+$ = 265.1376; $C_{14}H_{20}F_2ONa$ requires 265.1374.

**(*2S,3R*)-2,3,4-trimethyl-1-(naphthalen-2-yl)pentan-3-ol (5m)**

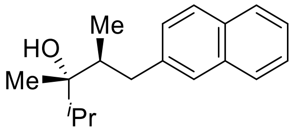


Colorless oil, 31.5 mg (82% yield). $^1$H NMR (400 MHz, Chloroform-*d*) δ 7.82 – 7.76 (m, 3H), 7.60 (s, 1H), 7.48 – 7.40 (m, 2H), 7.32 (dd, *J* = 8.3, 1.8 Hz, 1H), 3.13 (dd, *J* = 13.2, 3.0 Hz, 1H), 2.35 (dd, *J* = 13.2, 11.3 Hz, 1H), 2.09 – 1.97 (m, 2H), 1.20 (s, 3H), 1.05 (d, *J* = 6.8 Hz, 3H), 0.96 (d, *J* = 6.8 Hz, 3H), 0.83 (d, *J* = 6.7 Hz, 3H). $^{13}$C NMR (101 MHz, Chloroform-*d*) δ 139.4, 133.5, 131.9, 127.8, 127.7, 127.6, 127.4, 127.3, 125.9, 125.1, 76.2, 41.8, 38.0, 34.3, 19.4, 17.6, 16.6, 12.7. **HRMS-ESI**: Found [M+Na]$^+$ = 279.1719; $C_{18}H_{24}ONa$ requires 279.1719.

**(*3R,4S*)-2,3,4-trimethyl-6-phenylhexan-3-ol (6a)**

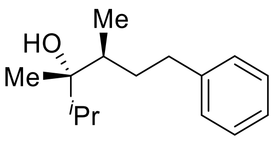


Colorless oil, 28.1 mg (85% yield). $^1$H NMR (400 MHz, Chloroform-*d*) δ 7.31 – 7.26 (m, 2H), 7.21 – 7.15 (m, 3H), 2.80 (ddd, *J* = 13.55, 10.43, 4.98 Hz, 1H), 2.48 (ddd, *J* = 13.59, 10.07, 6.73 Hz, 1H), 1.92 – 1.73 (m, 2H), 1.65 (dqd, *J* = 10.67, 6.74, 2.49 Hz, 1H), 1.38 – 1.23 (m, 1H), 1.02 (d, *J* = 6.82 Hz, 3H), 1.01 (s, 3H), 0.87 (d, *J* = 6.83 Hz, 3H), 0.82 (d, *J* = 6.78 Hz, 3H). $^{13}$C NMR (101 MHz, Chloroform-*d*) δ 142.7, 128.4, 128.3, 125.7, 76.2, 39.2, 34.5, 33.9, 33.7, 19.3, 17.5, 16.3, 13.2. **HRMS-ESI**: Found [M+Na]$^+$ = 243.1719; $C_{15}H_{24}ONa$ requires 243.1719.

**(*3R,4S*)-2,3,4-trimethylhexan-3-ol (6b)**

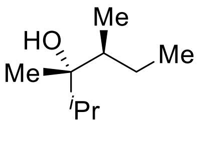


Colorless oil, 14.5 mg (67% yield). $^1$H NMR (400 MHz, Chloroform-d) δ 1.91 – 1.81 (m, 1H), 1.56 – 1.44 (m, 1H), 1.30 – 1.16 (m, 2H), 1.01 (s, 3H), 0.98 – 0.85 (m, 12H). $^{13}$C NMR (101 MHz, Chloroform-*d*) δ 76.5, 41.7, 34.1, 24.2, 19.4, 17.7, 16.6, 13.0, 12.7. **HRMS-ESI**: Found [M+Na]$^+$ = 167.1414; $C_9H_{20}ONa$ requires 167.1406.

**(*3R,4S*)-2,3,4-trimethylheptan-3-ol (6c)**

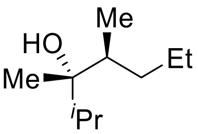

Colorless oil, 19.4 mg (82% yield). $^{1}$H NMR (400 MHz, Chloroform-*d*) δ 1.87 (p, *J* = 6.8 Hz, 1H), 1.61 (ddd, *J* = 10.6, 6.7, 2.4 Hz, 1H), 1.55 – 1.44 (m, 2H), 1.43 – 1.37 (m, 1H), 1.23 – 1.17 (m, 1H), 1.01 (s, 3H), 0.93 – 0.87 (m, 12H). $^{13}$C NMR (101 MHz, Chloroform-*d*) δ 76.3, 39.3, 33.9, 33.7, 21.2, 19.2, 17.5, 16.4, 14.4, 13.1. **HRMS-ESI**: Found [M+Na]$^{+}$ = 181.1569; $C_{10}H_{22}ONa$ requires 181.1563.

**(*3R,4S*)-2,3,4-trimethyloctan-3-ol (6d)**

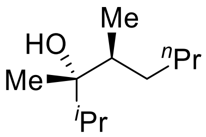

Colorless oil, 20.6 mg (80% yield). $^{1}$H NMR (400 MHz, Chloroform-*d*) δ 1.87 (p, *J* = 6.8 Hz, 1H), 1.66 – 1.52 (m, 2H), 1.51 – 1.41 (m, 2H), 1.39 – 1.29 (m, 2H), 1.25 – 1.12 (m, 2H), 1.01 (s, 3H), 0.92 (s, 2H), 0.91 – 0.89 (m, 6H), 0.89 – 0.88 (m, 4H). $^{13}$C NMR (101 MHz, Chloroform-*d*) δ 76.3, 39.6, 33.9, 31.2, 30.5, 23.0, 19.2, 17.5, 16.5, 14.2, 13.2. **HRMS-ESI**: Found [M+Na]$^{+}$ = 195.1713; $C_{11}H_{24}ONa$ requires 195.1719.

**(*3R,4S*) -2,3,4-trimethylnonan-3-ol (6e)**

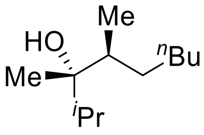

Colorless oil, 25.7 mg (92% yield).$^{1}$H NMR (400 MHz, Chloroform-*d*) δ 1.90 – 1.82 (m, 1H), 1.66 – 1.54 (m, 2H), 1.48 – 1.42 (m, 2H), 1.33 – 1.26 (m, 4H), 1.23 – 1.14 (m, 2H), 1.00 (d, *J* = 2.7 Hz, 3H), 0.92 (s, 1H), 0.90 (d, *J* = 2.2 Hz, 5H), 0.89 – 0.86 (m, 6H). $^{13}$C NMR (101 MHz, Chloroform-*d*) δ 76.3, 39.6, 33.9, 32.2, 31.4, 27.9, 22.7, 19.2, 17.5, 16.5, 14.1, 13.2. **HRMS-ESI**: Found [M+Na]$^{+}$ = 209.1872; $C_{12}H_{26}ONa$ requires 209.1876.

**(*3R,4S*)-2,3,4,7-tetramethyloctan-3-ol (6f)**

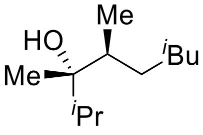

Colorless oil, 23.7 mg (85% yield). $^{1}$H NMR (400 MHz, Chloroform-*d*) δ 1.87 (p, *J* = 6.82 Hz, 1H), 1.58 – 1.43 (m, 4H), 1.34 – 1.24 (m, 1H), 1.15 – 1.03 (m, 1H), 1.01 (s, 3H), 0.92 – 0.86 (m, 15H).$^{13}$C NMR (101 MHz, Chloroform-*d*) δ 76.4, 40.0, 37.7, 34.0, 29.2, 28.4, 23.1, 22.4, 19.3, 17.5, 16.5, 13.2. **HRMS-ESI**: Found [M+Na]$^{+}$ = 209.1873; $C_{12}H_{26}ONa$ requires 209.1876.

**(*3R,4S*)-2,3,4-trimethyldecan-3-ol (6g)**

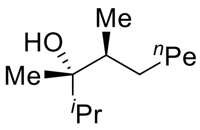

Colorless oil, 26.1 mg (87% yield). $^{1}$H NMR (400 MHz, Chloroform-*d*) δ 1.91 – 1.79 (m, 1H), 1.51 – 1.36 (m, 3H), 1.35 – 1.20 (m, 8H), 1.00 (s, 3H), 0.95 – 0.82 (m, 12H). $^{13}$C NMR (101 MHz, Chloroform-*d*) δ 76.5, 39.7, 34.1, 32.1, 31.6, 29.8, 28.4, 22.8, 19.4, 17.7, 16.6, 14.3, 13.3. **HRMS-ESI**: Found [M+Na]$^{+}$ = 223.2040; $C_{13}H_{28}ONa$ requires 223.2032.

**(*1R,2S*)-2-benzylcyclohexan-1-ol (8a)**

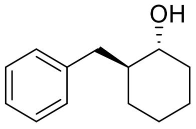


Colorless oil, 10.0 mg (35% yield). $^1$H NMR (400 MHz, Chloroform-*d*) δ 7.33 – 7.26 (m, 2H), 7.22 – 7.16 (m, 3H), 3.30 (td, *J* = 9.71, 4.41 Hz, 1H), 3.17 (dd, *J* = 13.33, 4.01 Hz, 1H), 2.36 (dd, *J* = 13.31, 9.14 Hz, 1H), 2.02 – 1.95 (m, 1H), 1.75 – 1.50 (m, 4H), 1.41 – 1.17 (m, 3H), 1.16 – 1.02 (m, 1H), 0.98 – 0.84 (m, 1H). $^{13}$C NMR (101 MHz, Chloroform-*d*) δ 140.8, 129.4, 128.2, 125.8, 74.6, 47.1, 39.0, 35.8, 30.0, 25.4, 24.9. **HRMS-ESI**: Found [M+Na]$^+$ = 213.1262; $C_{13}H_{18}ONa$ requires 213.1250.

**(*1R,2S*)-2-benzyl-5-methylcyclohexan-1-ol (8b)**

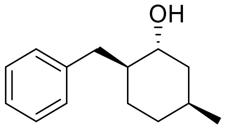


Colorless oil, 9.2 mg (30% yield).$^1$H NMR (400 MHz, Chloroform-*d*) δ 7.35 – 7.29 (m, 2H), 7.25 – 7.17 (m, 3H), 3.70 (td, *J* = 6.82, 3.77 Hz, 1H), 2.95 (dd, *J* = 13.49, 5.43 Hz, 1H), 2.49 (dd, *J* = 13.46, 8.97 Hz, 1H), 1.98 (tt, *J* = 7.24, 3.34 Hz, 1H), 1.73 – 1.39 (m, 7H), 0.97 (d, *J* = 6.98 Hz, 3H). $^{13}$C NMR (101 MHz, Chloroform-*d*) δ 141.0, 129.3, 128.4, 126.0, 70.6, 45.1, 39.5, 38.3, 30.2, 27.6, 24.3, 20.5. **HRMS-ESI**: Found [M+Na]$^+$ = 227.1414; $C_{14}H_{20}ONa$ requires 227.1406.

**(*1R,2S*)-2-benzyl-5-methylcyclohexan-1-ol (10a)**

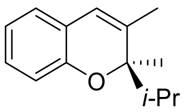


Colorless oil, 12.1 mg (40% yield). $^1$H NMR (400 MHz, Chloroform-*d*) δ 7.0 (td, J = 7.7, 1.7 Hz, 1H), 6.9 (dd, J = 7.4, 1.7 Hz, 1H), 6.8 – 6.7 (m, 2H), 6.1 (p, J = 1.4 Hz, 1H), 2.1 (hept, J = 6.8 Hz, 1H), 1.9 (d, J = 1.5 Hz, 3H), 1.4 (s, 3H), 1.0 (dd, J = 23.6, 6.8 Hz, 6H).. $^{13}$C NMR (101 MHz, Chloroform-*d*) δ 152.8, 137.3, 128.0, 125.2, 122.3, 120.3, 120.2, 115.3, 83.3, 34.6, 21.9, 19.8, 17.5, 16.3. **HRMS-ESI**: Found [M+Na]$^+$ = 225.1247; $C_{14}H_{18}ONa$ requires 225.1255.

**Catalyst II**

**Prepared according to literature procedure.[1]**

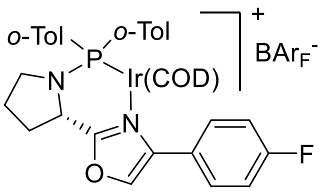


Orange solid, 55.2 mg (30% yield). $^1$H NMR (400 MHz, Chloroform-*d*) δ 7.74 – 7.68 (m, 8H), 7.51 (s, 4H), 7.49 – 7.45 (m, 2H), 7.45 – 7.40 (m, 1H), 7.36 – 7.30 (m, 1H), 7.25 – 7.16 (m, 3H), 7.07 (t, *J* = 7.64 Hz, 1H), 6.92 (t, *J* = 8.46 Hz, 2H), 6.87 – 6.82 (m, 2H), 6.61 (ddd, *J* = 9.34, 7.92, 1.32 Hz, 1H), 5.01 (d, *J* = 7.44 Hz, 1H), 4.73 (d, *J* = 1.70 Hz, 1H), 4.73 – 4.62 (m, 1H), 3.61 (s, 1H), 3.60 – 3.52 (m, 1H), 3.24 (s, 3H), 3.08 (s, 1H), 2.75 (p, *J* = 7.33 Hz, 1H), 2.37 – 2.26 (m, 2H), 2.23

(s, 3H), 2.20 – 2.12 (m, 1H), 2.05 – 1.98 (m, 1H), 1.92 – 1.83 (m, 2H), 1.83 – 1.77 (m, 2H), 1.73 – 1.67 (m, 2H), 1.11 – 1.03 (m, 1H), 1.02 – 0.93 (m, 1H). $^{13}$C NMR (101 MHz, Chloroform-*d*) δ 172.2, 162.6, 162.1, 161.6, 161.1, 156.7, 141.9 (d, *J* = 14.87 Hz), 139.6 (d, *J* = 15.68 Hz), 135.0, 132.6, 131.5, 129.4 – 128.5 (m), 127.7 (d, *J* = 7.38 Hz), 126.7 (d, *J* = 9.21 Hz), 126.0, 125.2, 124.6, 123.3, 120.6, 117.6, 116.8, 116.3, 116.1, 90.8 (d, *J* = 7.77 Hz), 78.4 (d, *J* = 18.51 Hz), 75.7, 67.1 (d, *J* = 9.62 Hz), 65.0, 60.2 (d, *J* = 4.29 Hz), 42.5 (d, *J* = 5.63 Hz), 39.0 – 38.8 (m), 36.7 (d, *J* = 4.60 Hz), 34.7, 30.1 – 29.7 (m), 29.2, 28.0, 25.1 – 24.1 (m), 21.8 (d, *J* = 8.42 Hz). $^{19}$F NMR (377 MHz, Chloroform-*d*) δ -62.4, -108.6. $^{31}$P NMR (162 MHz, Chloroform-*d*) δ 49.4. **HRMS-EI**: Found [M - $BAr_F$]$^+$ = 744.8919, [$C_{35}H_{38}IrN_2OP$]$^+$ requires: 744.8906.

**Separation methods and optical rotations**

| Entry | Product | Separation method | Optical rotation | *ee*/ % | *d.r.* |
|---|---|---|---|---|---|
| 1 | 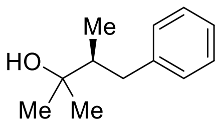 | **GC-MS,** column Chiraldex β-DM, 50 ºC to 175°C at 1°C/min. $t_R$ = 62.2 min (major), $t_R$ = 63.8 min (minor). | $[\alpha]_D^{29}$ = 38.0 (c = 0.1, $CHCl_3$) | 94 | -- |
| 2 | 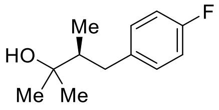 | **GC-MS,** column Chiraldex β-DM, 50 ºC to 175°C at 1°C/min. $t_R$ = 64.7 min (major), $t_R$ = 66.5 min (minor). | $[\alpha]_D^{29}$ = 36.0 (c = 0.1, $CHCl_3$) | 90 | -- |
| 3 | 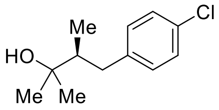 | **SFC**, IF column, 5% MeOH, 2 mL/min, $t_R$ = 6.3 min (minor)/6.5 min (major) | $[\alpha]_D^{29}$ = 20.0 (c = 0.1, $CHCl_3$) | 92 | -- |
| 4 | 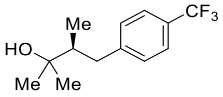 | **SFC**, IF column, 5% MeOH, 2 mL/min, $t_R$ = 11.2 min (major), $t_R$ = 12.4 (minor) | $[\alpha]_D^{29}$ = 42.0 (c = 0.1, $CHCl_3$) | 94 | -- |
| 5 | 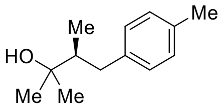 | **GC-MS,** column Chiraldex β-DM, 50 ºC to 175°C at 1°C/min. $t_R$ = 69.0 min (major), $t_R$ = 70.7 min (minor). | $[\alpha]_D^{29}$ = 46.0 (c = 0.1, $CHCl_3$) | 94 | -- |

| | | | | | |
|---|---|---|---|---|---|
| 6 | 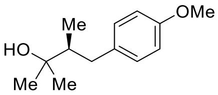 | **SFC**, IF column, 5% MeOH, 2 mL/min, $t_R$ = 7.3 min (minor)/7.5 min (major) | $[\alpha]_D^{29}$ = 25.0 (c = 0.1, $CHCl_3$) | 92 | -- |
| 7 | 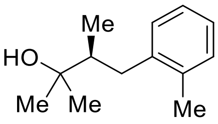 | **GC-MS,** column Chiraldex β-DM, 50 °C to 175°C at 1°C/min. $t_R$ = 65.7 min (major), $t_R$ = 68.5 min (minor). | $[\alpha]_D^{29}$ = 87.0 (c = 0.1, $CHCl_3$) | 99 | -- |
| 8 | 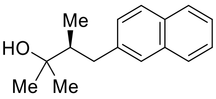 | **SFC**, IF column, 5% MeOH, 2 mL/min, $t_R$ = 11.1 min (minor)/12.0 (major). | $[\alpha]_D^{29}$ = 21.0 (c = 0.1, $CHCl_3$) | 89 | -- |
| 9 | 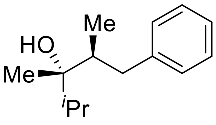 | **GC-MS,** column Chiraldex β-DM, 50 °C to 175°C at 1°C/min. $t_R$ = 12.4 (minor), $t_R$ = 77.8 min (major)/ | $[\alpha]_D^{29}$ = 37.0 (c = 0.1, $CHCl_3$) | 99 | 99:1 |
| 10 | 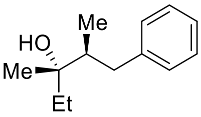 | **SFC**, ID column, 5% MeOH, 2 mL/min, $t_R$ = 17.1 min (minor)/16.6 (major). | –– | 94 | 56:44 |
| 11 | 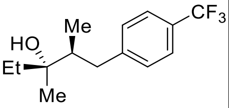 | **GC-MS,** column Chiraldex β-DM, 50 °C to 175°C at 1°C/min. $t_R$ = 66.3 min (minor), $t_R$ = 67.8 min (major), $t_R$ = 76.5 min (minor). | $[\alpha]_D^{29}$ = 9.0 (c = 0.1, $CHCl_3$) | 98 | 68:32 |
| 12 | 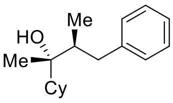 | **SFC**, ID column, 5% MeOH, 2 mL/min, $t_R$ = 18.2 min (major)/19.1 (minor). | $[\alpha]_D^{29}$ = 32.0 (c = 0.1, $CHCl_3$) | 94 | 99:1 |
| 13 | 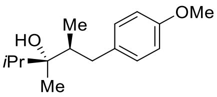 | **SFC**, IC column, 5% MeOH, 2 mL/min, $t_R$ = 17.1 min (major)/18.0 (minor). | $[\alpha]_D^{29}$ = 49.0 (c = 0.1, $CHCl_3$) | 98 | 99:1 |
| 14 | 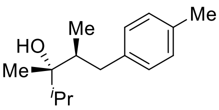 | **GC-MS,** column Chiraldex β-DM, 50 °C to 175°C at 1°C/min. $t_R$ = 43.0 min (minor), $t_R$ = 50.8 min (minor), $t_R$ = 51.9 min (major). | $[\alpha]_D^{29}$ = 27.0 (c = 0.1, $CHCl_3$) | 96 | 96:4 |

| | | | | | |
|---|---|---|---|---|---|
| 15 | 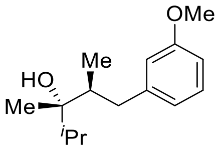 | **GC-MS,** column Chiraldex β-DM, iso 120°C 100min. $t_R$ = 54.0 min (minor), $t_R$ = 62.8 min (major). | $[\alpha]_D^{29}$ = 38.0 (c = 0.1, $CHCl_3$) | 99 | 99:1 |
| 16 | 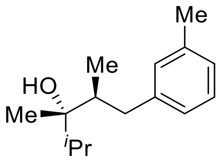 | **SFC**, OJH column, 5% MeOH, 2 mL/min, $t_R$ = 10.0 min (major). | $[\alpha]_D^{29}$ = 38.0 (c = 0.1, $CHCl_3$) | 99 | 99:1 |
| 17 | 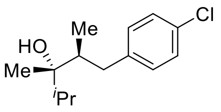 | **SFC**, OJH column, 5% MeOH, 2 mL/min, $t_R$ = 7.6 min (major). | $[\alpha]_D^{29}$ = 27.0 (c = 0.1, $CHCl_3$) | 99 | 99:1 |
| 18 | 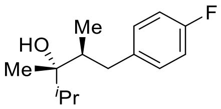 | **SFC**, IF column, 5% MeOH, 2 mL/min, $t_R$ = 8.3 min (major). | $[\alpha]_D^{29}$ = 12.0 (c = 0.1, $CHCl_3$) | 99 | 99:1 |
| 19 | 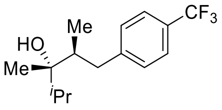 | **SFC**, OJH column, 5% MeOH, 2 mL/min, $t_R$ = 3.6 min (major). | $[\alpha]_D^{29}$ = 33.0 (c = 0.1, $CHCl_3$) | 99 | 99:1 |
| 20 | 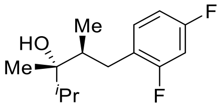 | **GC-MS,** column Chiraldex β-DM, 50 $^oC$ to 175°C at 1°C/min. $t_R$ = 49.0 min (major). | $[\alpha]_D^{29}$ = 18.0 (c = 0.1, $CHCl_3$) | 99 | 99:1 |
| 21 | 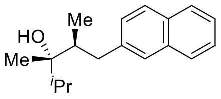 | **SFC**, OJH column, 5% MeOH, 2 mL/min, $t_R$ = 11.1 min (major)/11.6 (minor). | $[\alpha]_D^{29}$ = 17.0 (c = 0.1, $CHCl_3$) | 90 | 99:1 |
| 22 | 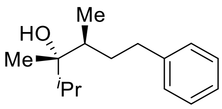 | **GC-MS,** column Chiraldex β-DM, 50 $^oC$ to 175°C at 1°C/min. $t_R$ = 83.2 min (minor), $t_R$ = 84.6 min (minor), $t_R$ = 85.2 min (major). | $[\alpha]_D^{29}$ = 32.0 (c = 0.1, $CHCl_3$) | 98 | 98:2 |
| 23 | 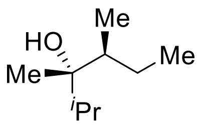 | **GC-MS,** column Chiraldex β-DM, iso 60 $^oC$ 50min. $t_R$ = 12.4 min (minor), $t_R$ = 14.4 min (major), $t_R$ = 15.7 min (minor). | $[\alpha]_D^{29}$ = 15.0 (c = 0.1, $CHCl_3$) | 87 | 98:2 |

| | | | | | |
|---|---|---|---|---|---|
| 24 | 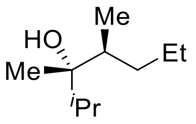 | **GC-MS,** column Chiraldex β-DM, 50 ºC to 175°C at 1°C/min. $t_R$ = 23.2 min (minor), $t_R$ = 25.9 min (minor), $t_R$ = 26.2 min (major). | $[\alpha]_D^{29}$ = 26.0 (c = 0.1, $CHCl_3$) | 95 | 94:6 |
| 25 | 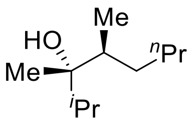 | **GC-MS,** column Chiraldex β-DM, 50 ºC to 175°C at 1°C/min. $t_R$ = 32.1 min (minor), $t_R$ = 34.5 min (minor), $t_R$ = 35 min (major). | $[\alpha]_D^{29}$ = 19.0 (c = 0.1, $CHCl_3$) | 99 | 97:3 |
| 26 | 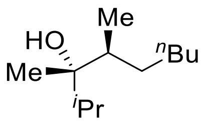 | **GC-MS,** column Chiraldex β-DM, 50 ºC to 175°C at 1°C/min. min (minor), $t_R$ = 45.0 min (minor), $t_R$ = 45.5 min (major). | $[\alpha]_D^{29}$ = 29.0 (c = 0.1, $CHCl_3$) | 87 | 90:10 |
| 27 | 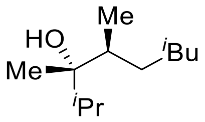 | **GC-MS,** column Chiraldex β-DM, 50 ºC to 175°C at 1°C/min. $t_R$ = 32.2 min (minor), $t_R$ = 32.2 min (minor), $t_R$ = 34.5 min (minor), $t_R$ = 35.0 min (major). | $[\alpha]_D^{29}$ = 12.0 (c = 0.1, $CHCl_3$) | 97 | 97:3 |
| 28 | 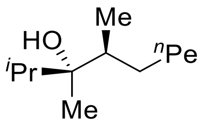 | **GC-MS,** column Chiraldex β-DM, 50 ºC to 175°C at 1°C/min. $t_R$ = 32.2 min (minor), $t_R$ = 32.2 min (minor), $t_R$ = 34.5 min (minor), $t_R$ = 35.0 min (major). | $[\alpha]_D^{29}$ = 26.0 (c = 0.1, $CHCl_3$) | 93 | 95:5 |
| 29 | 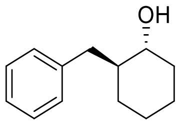 | **SFC**, IA column, 5% MeOH, 2 mL/min, $t_R$ = 23.7 min (major)/24.2 min (minor). | $[\alpha]_D^{29}$ = -29.5 (c = 0.1, $CHCl_3$) | 88 | 80:20 |
| 30 | 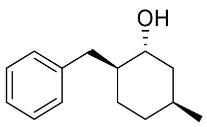 | **SFC**, IA column, 10% MeOH, 0.8 mL/min, $t_R$ = 3.40 min (major)/3.71 min (minor). | $[\alpha]_D^{29}$ = -19.0 (c = 0.1, $CHCl_3$) | 88 | 90:10 :0 |
| 31 | 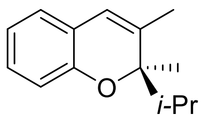 | **SFC,** ADH column, 5% MeOH, 2mL/min, $t_R$ = 6.05 min (major)/6.41 min (minor). | $[\alpha]_D^{29}$ = -22.0 (c = 0.1, $CHCl_3$) | 99% | - |

## 4. Assignment of the absolute configurations of hydrogenated products.

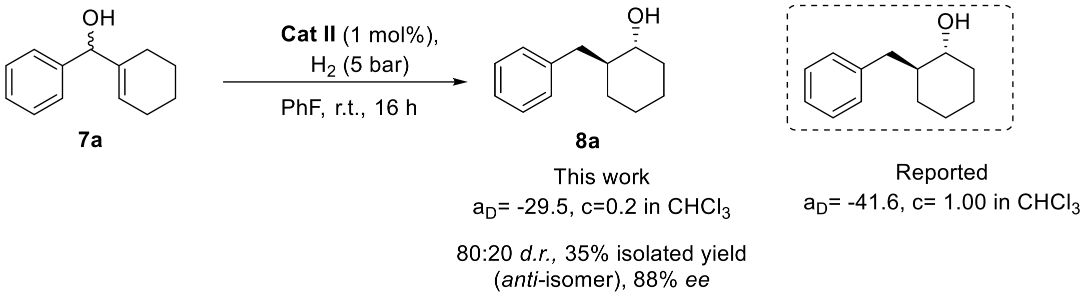


Scheme S3. Absolute configuration of compound **8a**.

Substrate **7a** was hydrogenated to obtain **8a** (Scheme S3, 35% yield, 88% *ee*). The correct diastereoisomer was assigned via $^{1}$H NNR. The optical rotations were $[\alpha]^{25}_{D}$= -29.5 (c = 0.1$CHCl_3$). This was confirmed to be the (*S*,*R*)-configuration for the product **8a** by comparison with the optical rotation data with literature.[2]

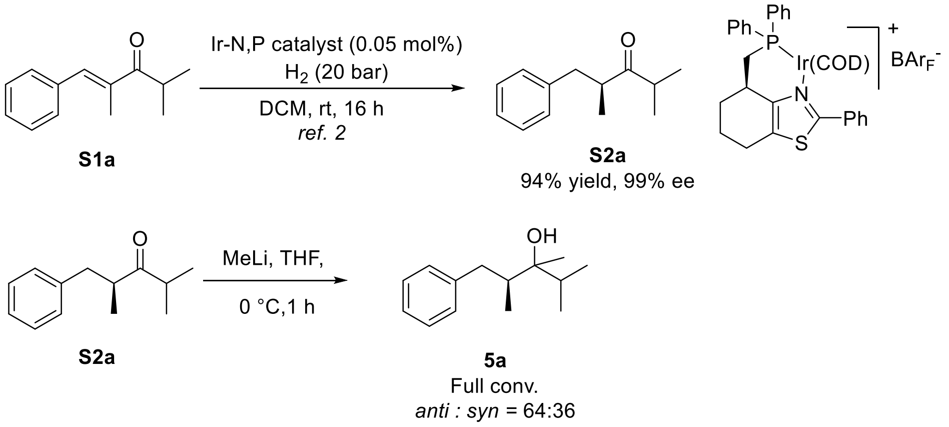


Scheme S4. Independent synthesis of **5a**.

Substrate **S1a** was hydrogenated following the reported procedure affording the enantioenriched ketone **S2a.**[3] After a short filtration on silica, **S2a** was dissolved in THF at 0 °C followed by a slow addition of 5 equiv. of MeLi. The reaction was quenched with $H_2O$ after 1 h and the crude was analyzed by $^{1}$H NMR. Product **5a** was obtained quantitatively in a 64:36 diastereomeric ratio. To assign the *anti* and *syn* configuration of the alcohol the Felkin-Ahn model was used to predict from which face of the chiral ketone the attack of the nucleophile would occurr (Scheme S5).[4]

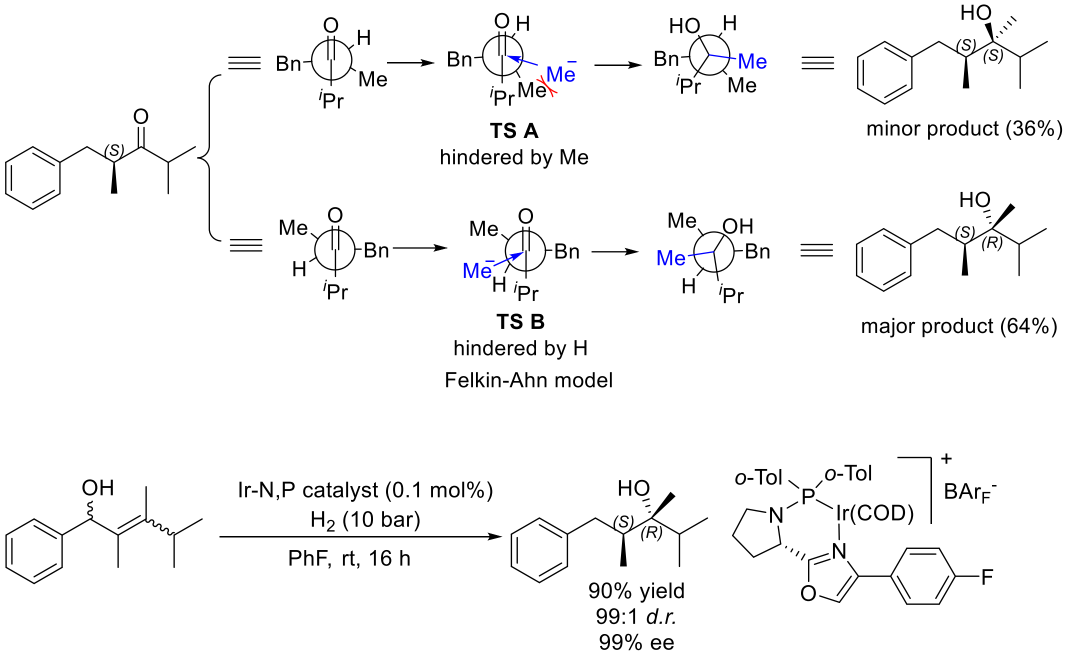


Scheme S5. Prediction of the absolute configuration using Felkin-Ahn model.

The obtained enantiomer matched the same peak in the chiral GC-MS chromatogram of compound **5a**.

## 5. NMR spectra

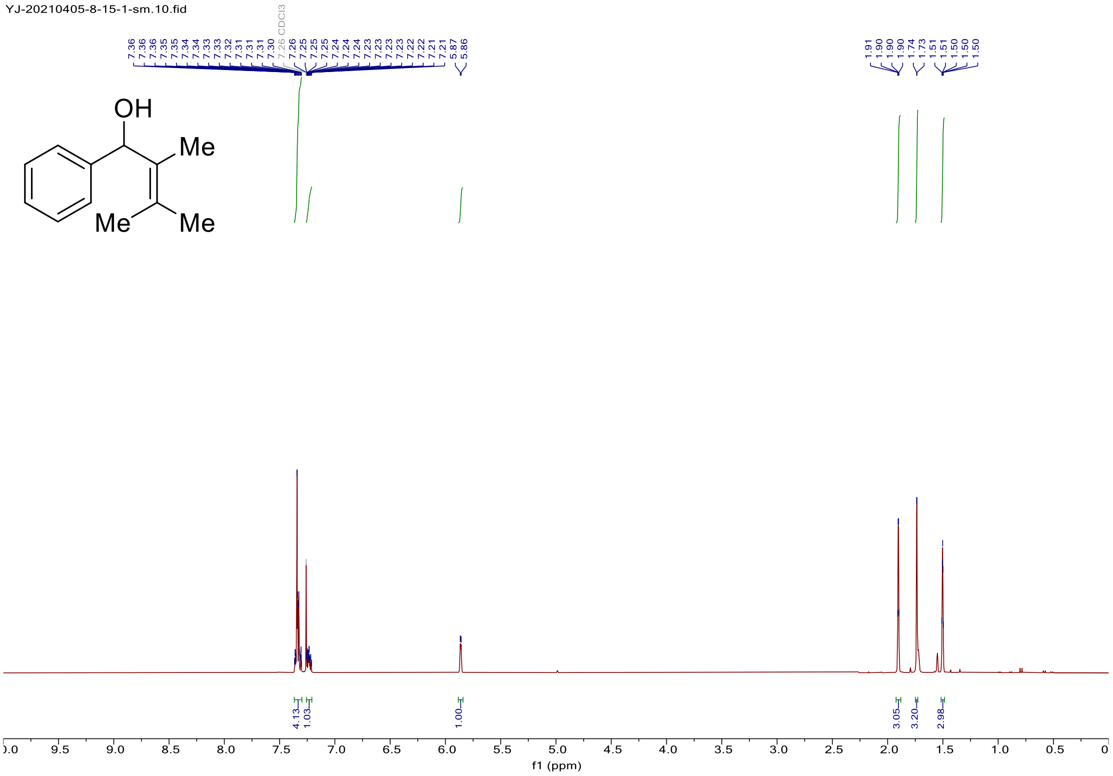

YJ-20210405-8-15-1-sm.11.fid

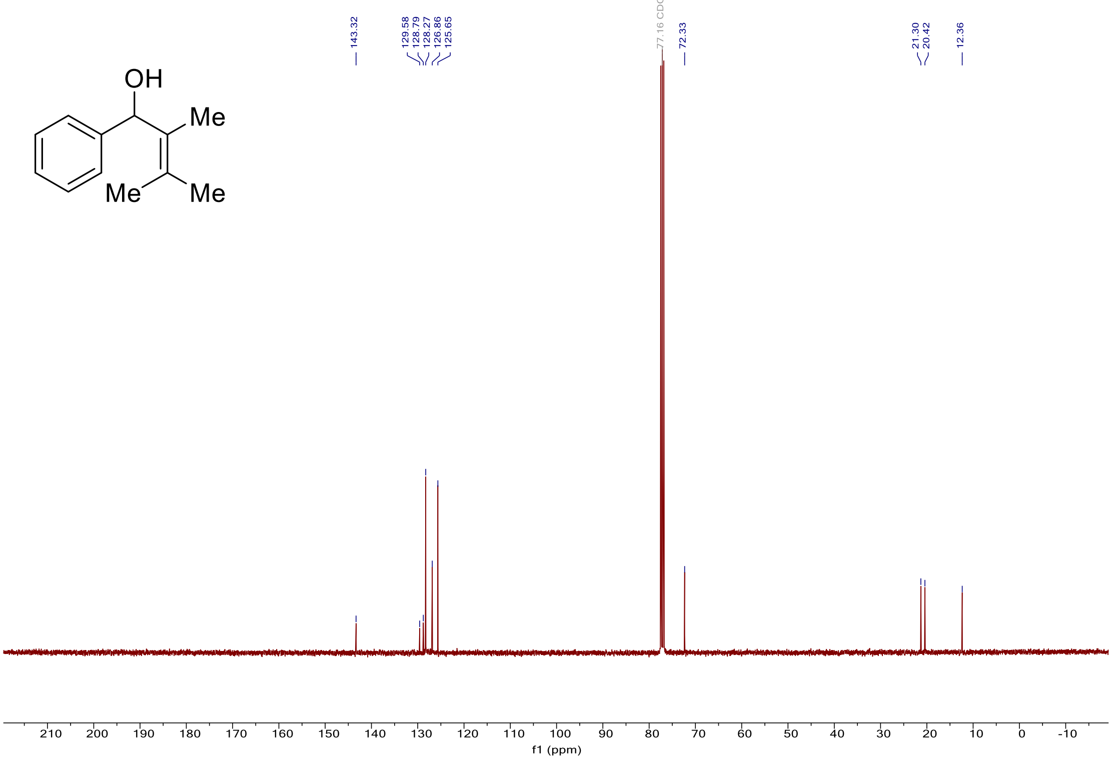


yanji-20211124-8-15-2-SM.1.fid

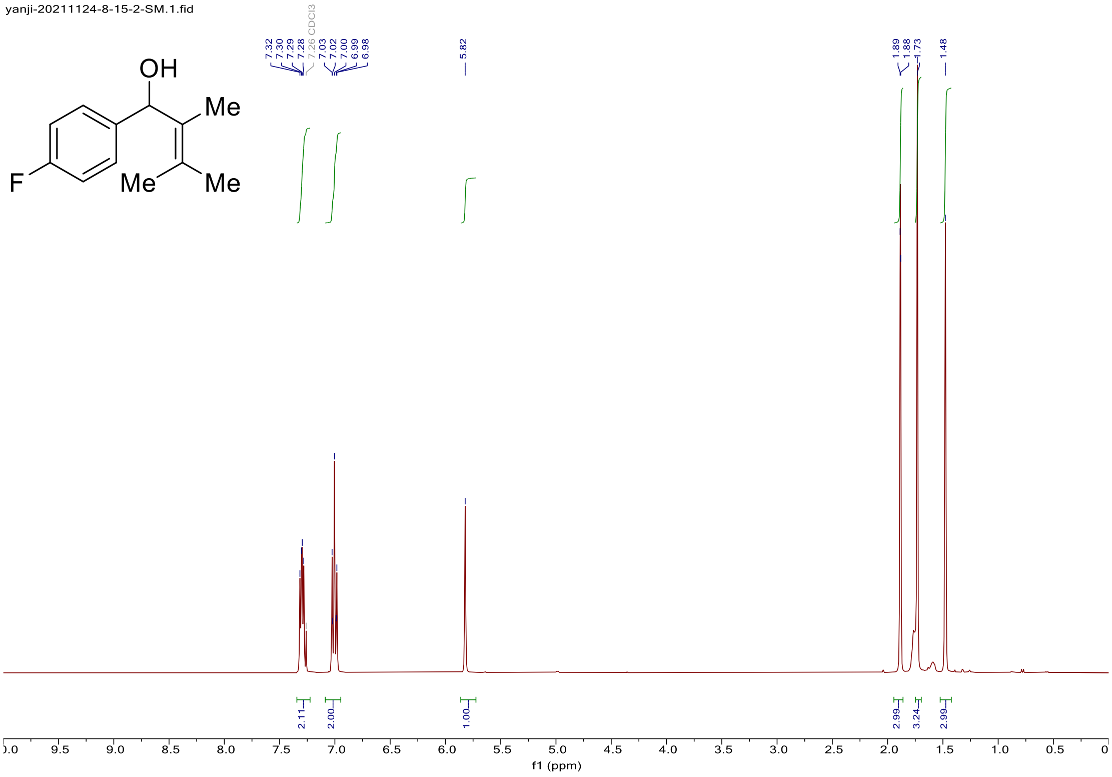

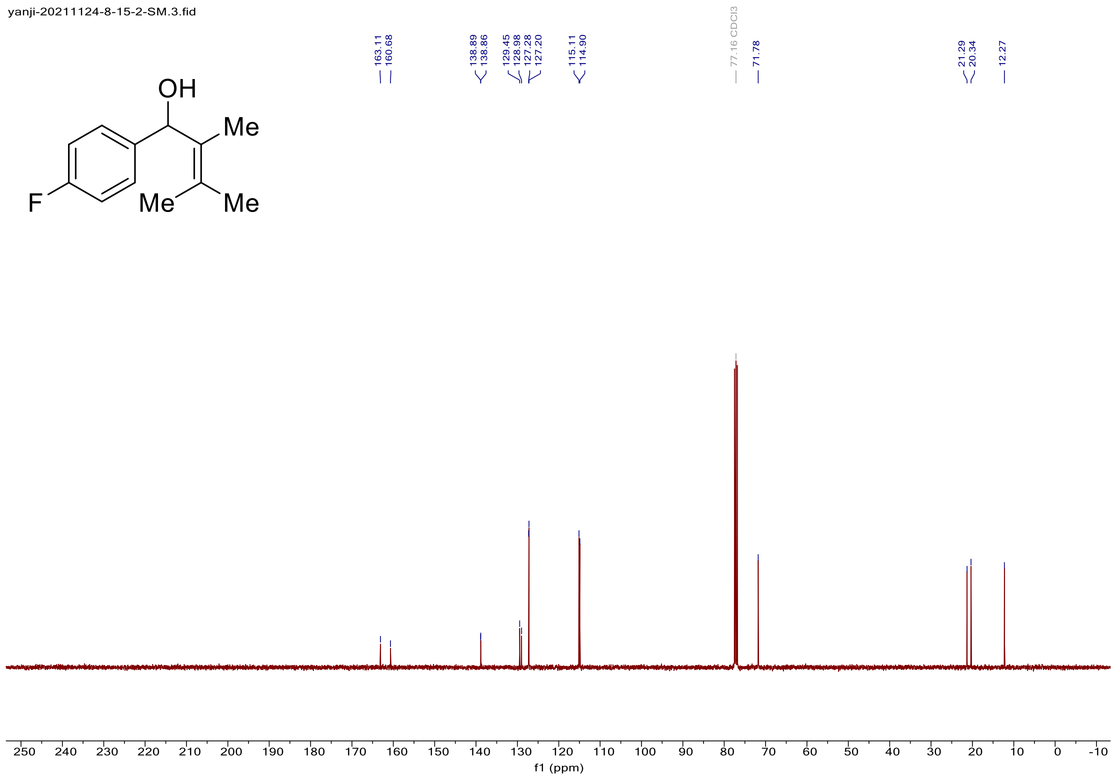
yanji-20211124-8-15-2-SM.3.fid
163.11
160.68
138.89
138.86
129.45
128.98
127.28
127.20
115.11
114.90
77.16 CDCl3
71.78
21.29
20.34
12.27
OH
Me
F
Me
Me
f1 (ppm)

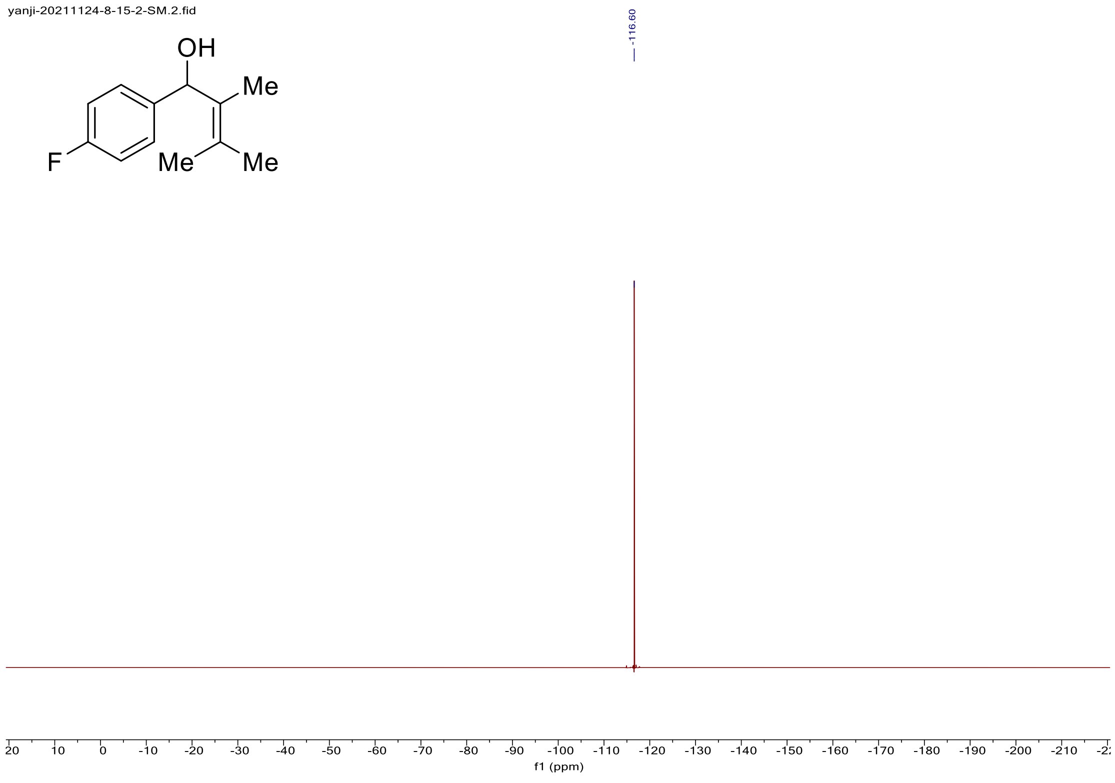
yanji-20211124-8-15-2-SM.2.fid
-116.60
OH
Me
F
Me
Me
f1 (ppm)

yanji-20220108-8-15-6-SM-2.1.fid

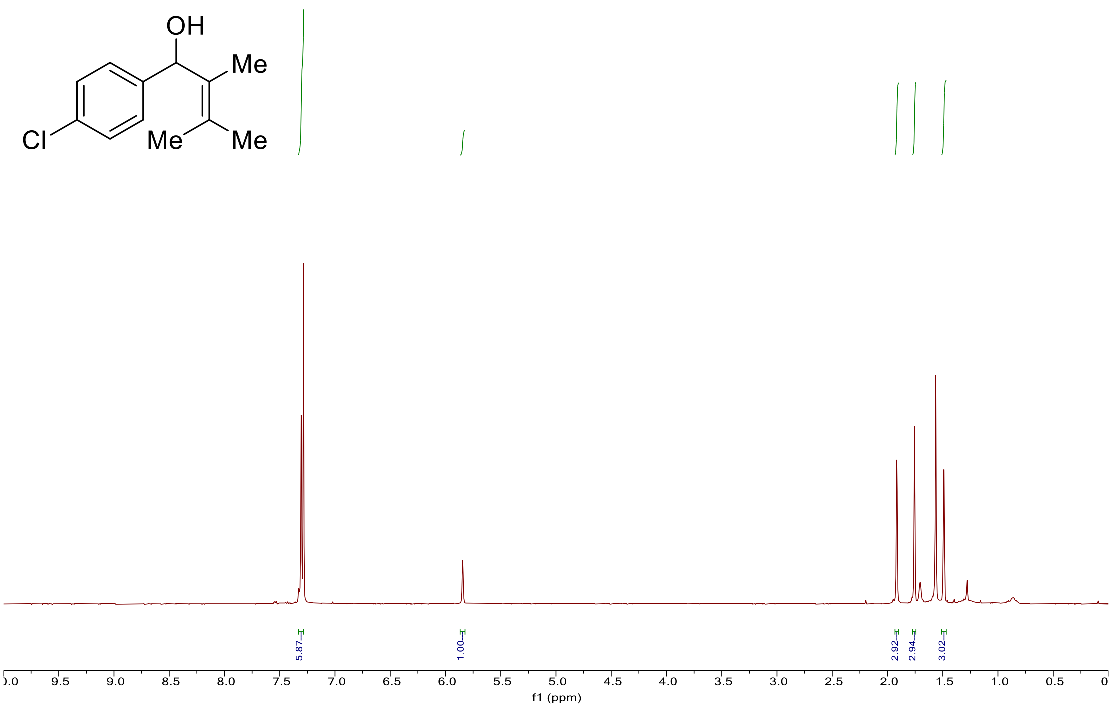


yanji-20220108-8-15-6-SM-2.2.fid

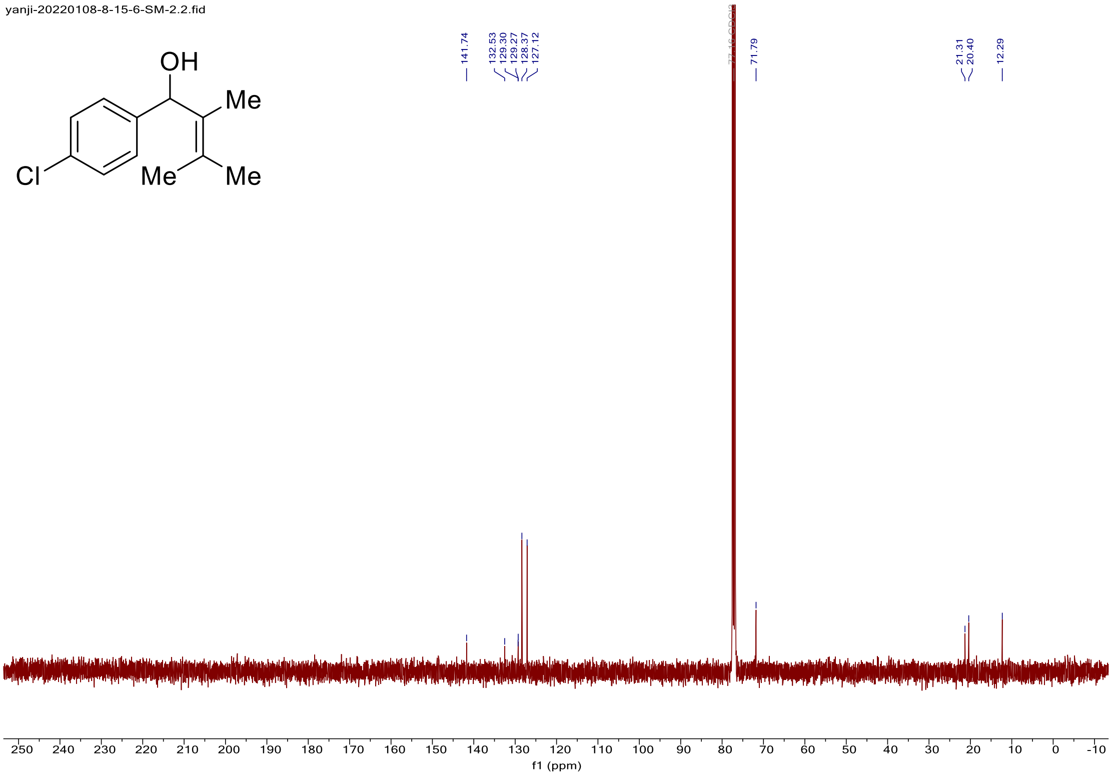

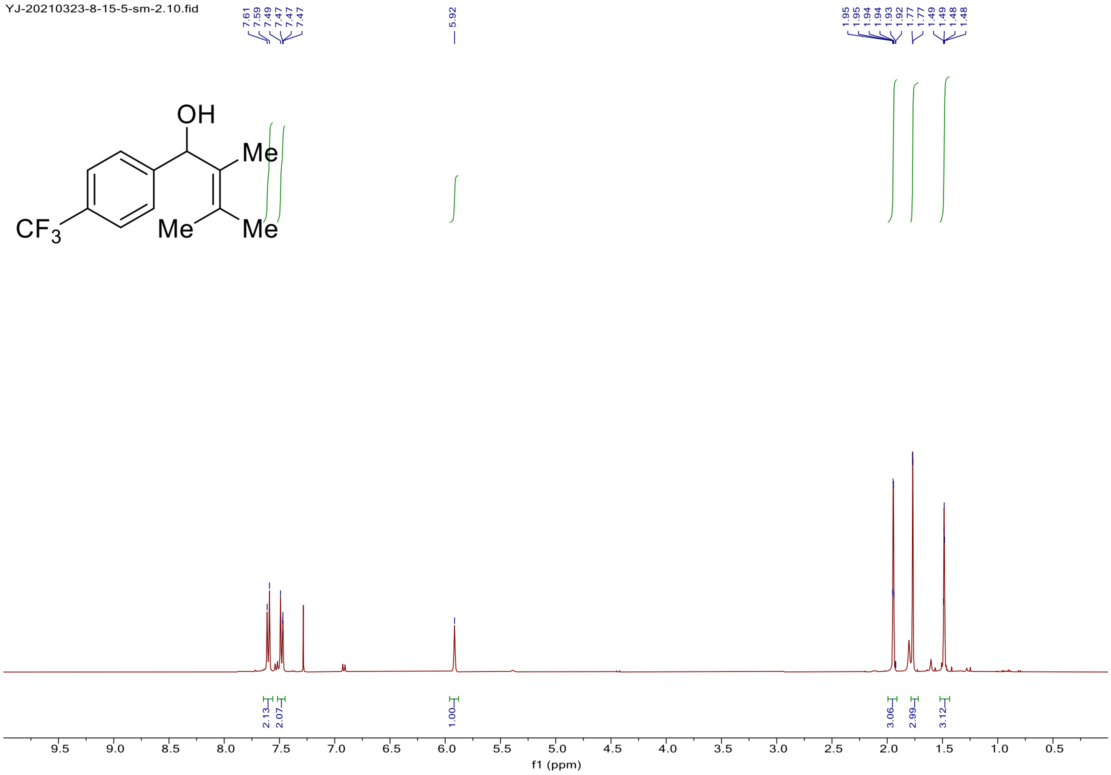
YJ-20210323-8-15-5-sm-2.10.fid
7.61
7.59
7.49
7.47
7.47
7.47
5.92
1.95
1.95
1.94
1.94
1.93
1.92
1.77
1.77
1.49
1.48
1.48
1.48
OH
Me
Me
Me
$CF_3$
2.13
2.07
1.00
3.06
2.99
3.12
f1 (ppm)

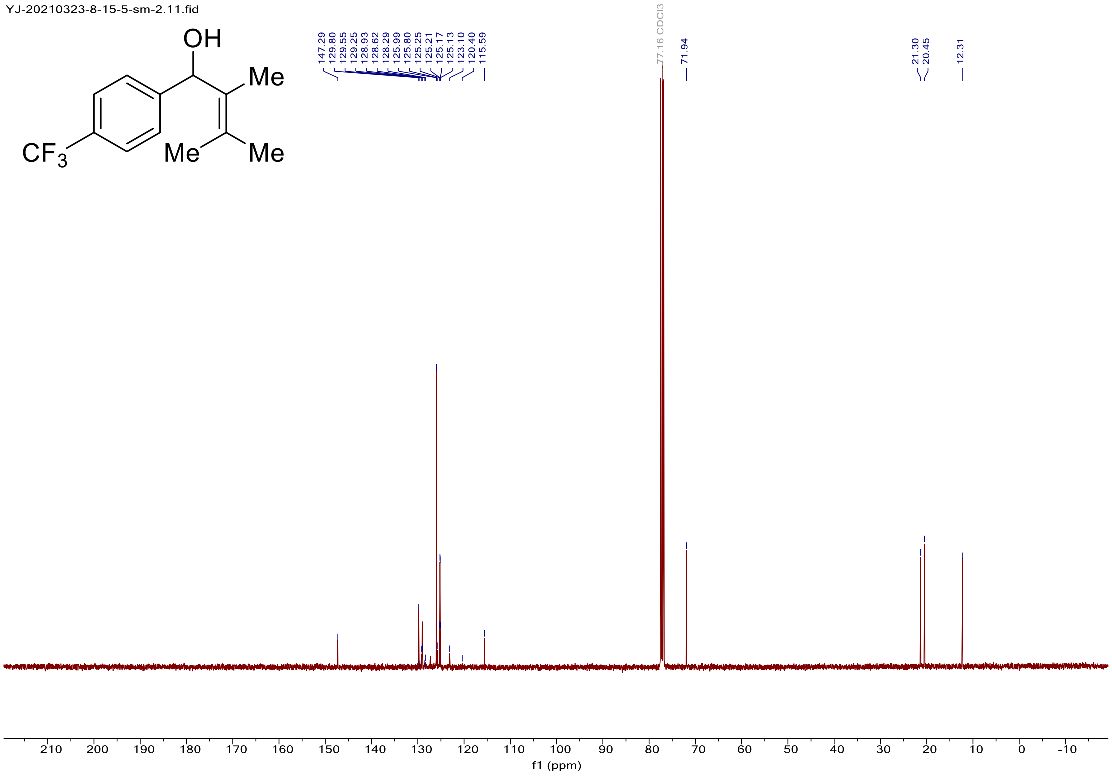
YJ-20210323-8-15-5-sm-2.11.fid
147.29
129.80
129.55
129.25
128.93
128.62
128.29
125.99
125.80
125.25
125.21
125.17
125.13
123.10
120.40
115.59
77.16 CDCl3
71.94
21.30
20.45
12.31
OH
Me
Me
Me
$CF_3$
f1 (ppm)

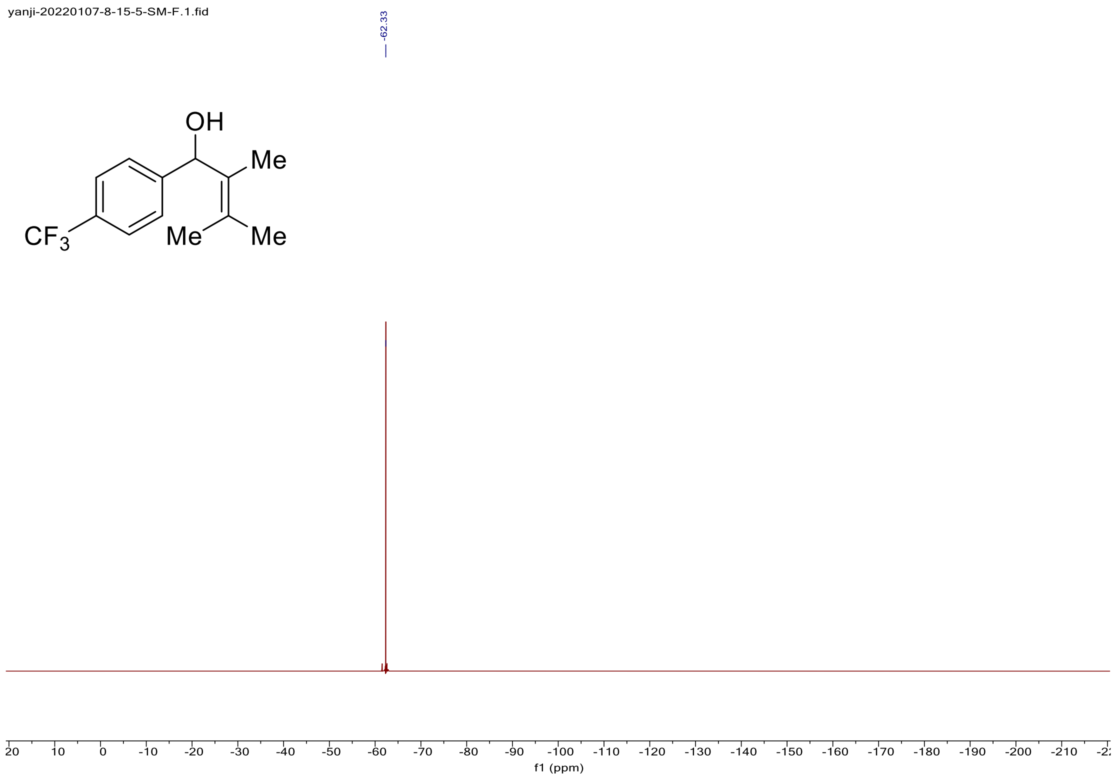
yanji-20220107-8-15-5-SM-F.1.fid
-62.33
OH
Me
CF3
Me
Me
20 10 0 -10 -20 -30 -40 -50 -60 -70 -80 -90 -100 -110 -120 -130 -140 -150 -160 -170 -180 -190 -200 -210
f1 (ppm)

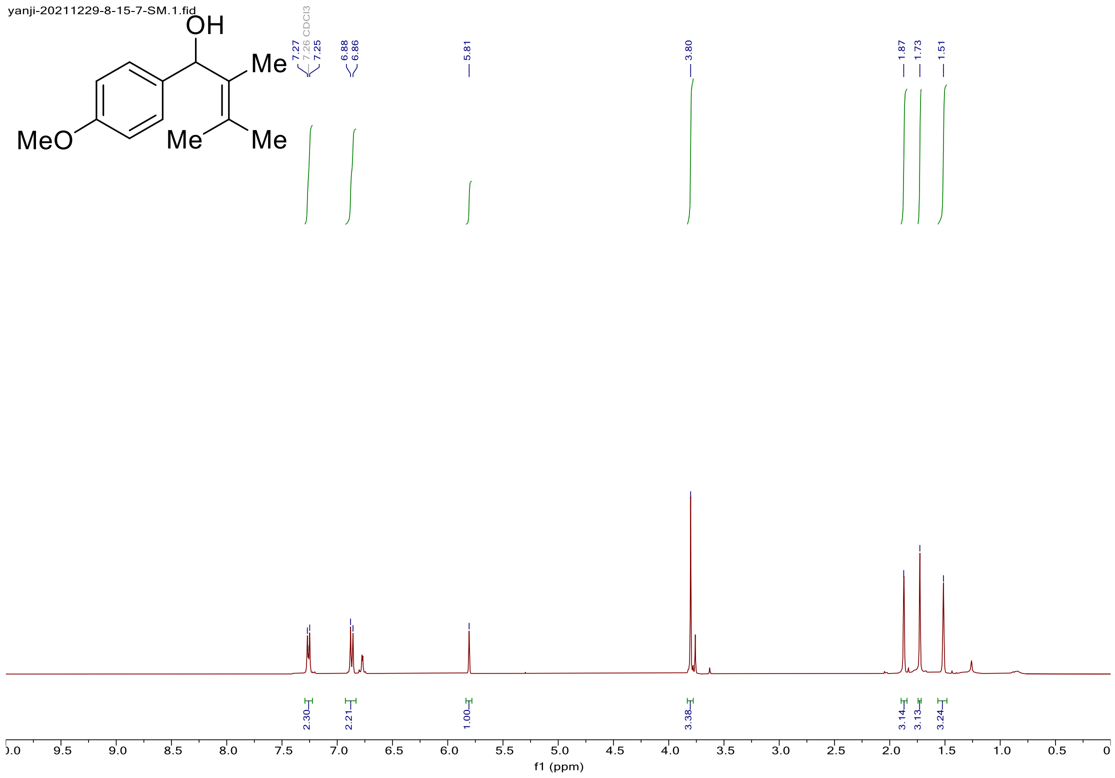
yanji-20211229-8-15-7-SM.1.fid
OH
Me
MeO
Me
Me
7.27
7.26 CDCl3
7.25
6.88
6.86
5.81
3.80
1.87
1.73
1.51
2.30
2.21
1.00
3.38
3.14
3.13
3.24
9.5 9.0 8.5 8.0 7.5 7.0 6.5 6.0 5.5 5.0 4.5 4.0 3.5 3.0 2.5 2.0 1.5 1.0 0.5 0
f1 (ppm)

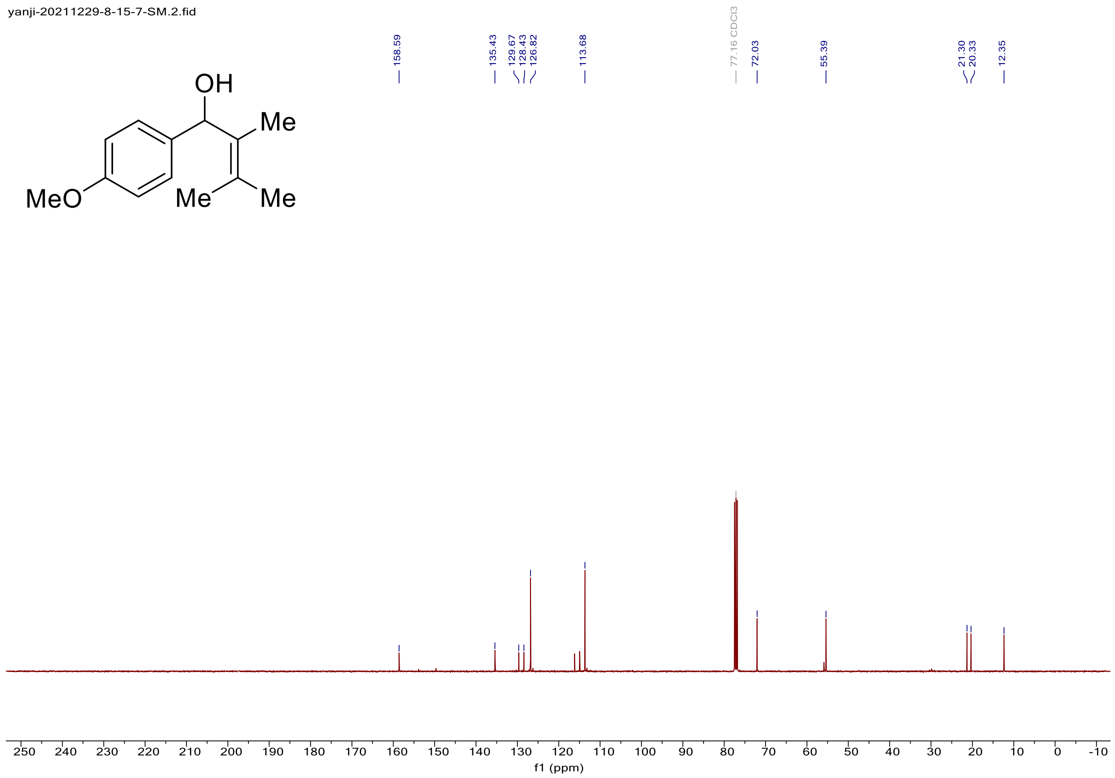
yanji-20211229-8-15-7-SM.2.fid
OH
Me
MeO
Me
Me
158.59
135.43
129.67
128.43
126.82
113.68
77.16 CDCl3
72.03
55.39
21.30
20.33
12.35
250 240 230 220 210 200 190 180 170 160 150 140 130 120 110 100 90 80 70 60 50 40 30 20 10 0 -10
f1 (ppm)

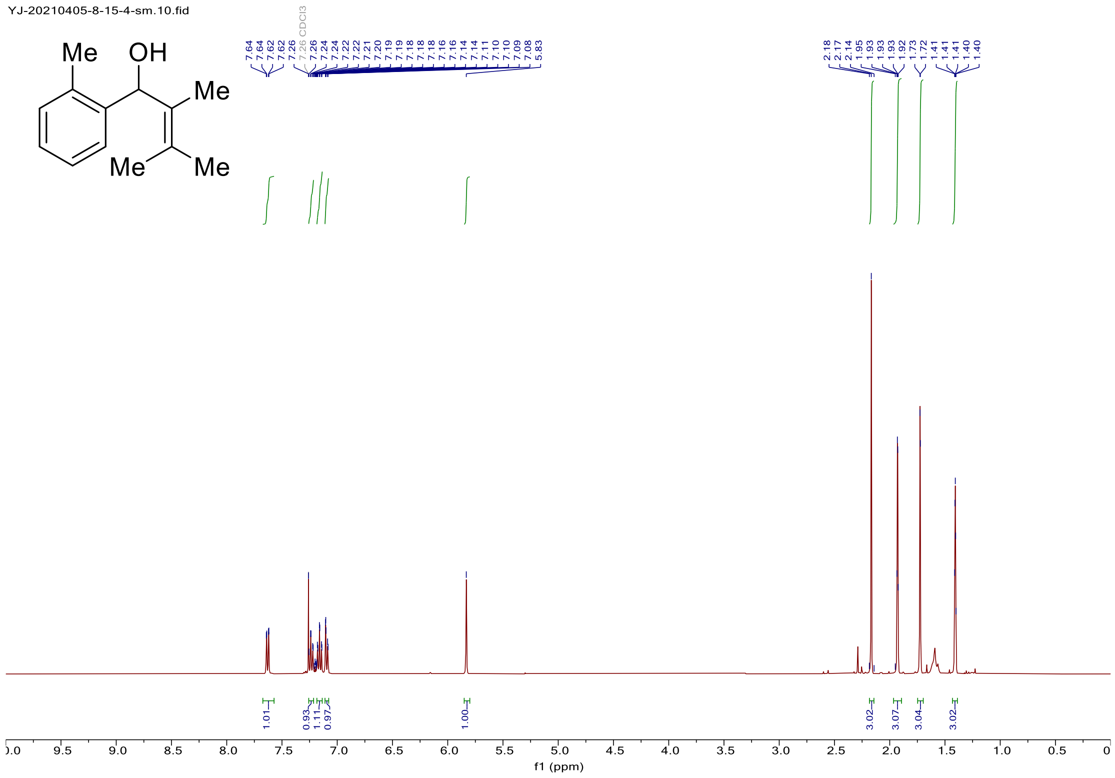
YJ-20210405-8-15-4-sm.10.fid
Me
OH
Me
Me
Me
7.64 7.64 7.62 7.62 7.26 7.26 CDCl3 7.26 7.24 7.24 7.22 7.22 7.21 7.20 7.19 7.19 7.18 7.18 7.18 7.16 7.16 7.14 7.14 7.11 7.10 7.10 7.09 7.08 5.83
2.18 2.17 2.14 1.95 1.93 1.93 1.93 1.92 1.73 1.72 1.41 1.41 1.41 1.40 1.40
1.01
0.93
1.11
0.97
1.00
3.02
3.07
3.04
3.02
f1 (ppm)

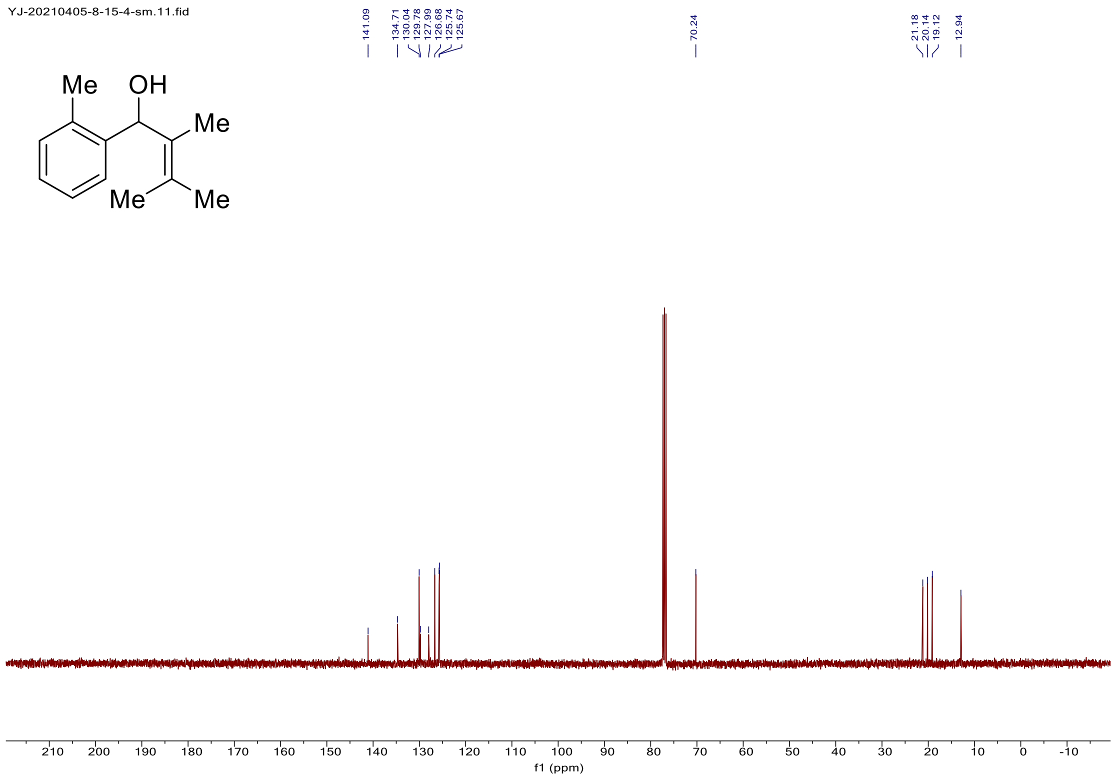

YJ-20210405-8-15-4-sm.11.fid
141.09
134.71
130.04
129.78
127.99
126.68
125.74
125.67
70.24
21.18
20.14
19.12
12.94
Me
OH
Me
Me
Me
210 200 190 180 170 160 150 140 130 120 110 100 90 80 70 60 50 40 30 20 10 0 -10
f1 (ppm)


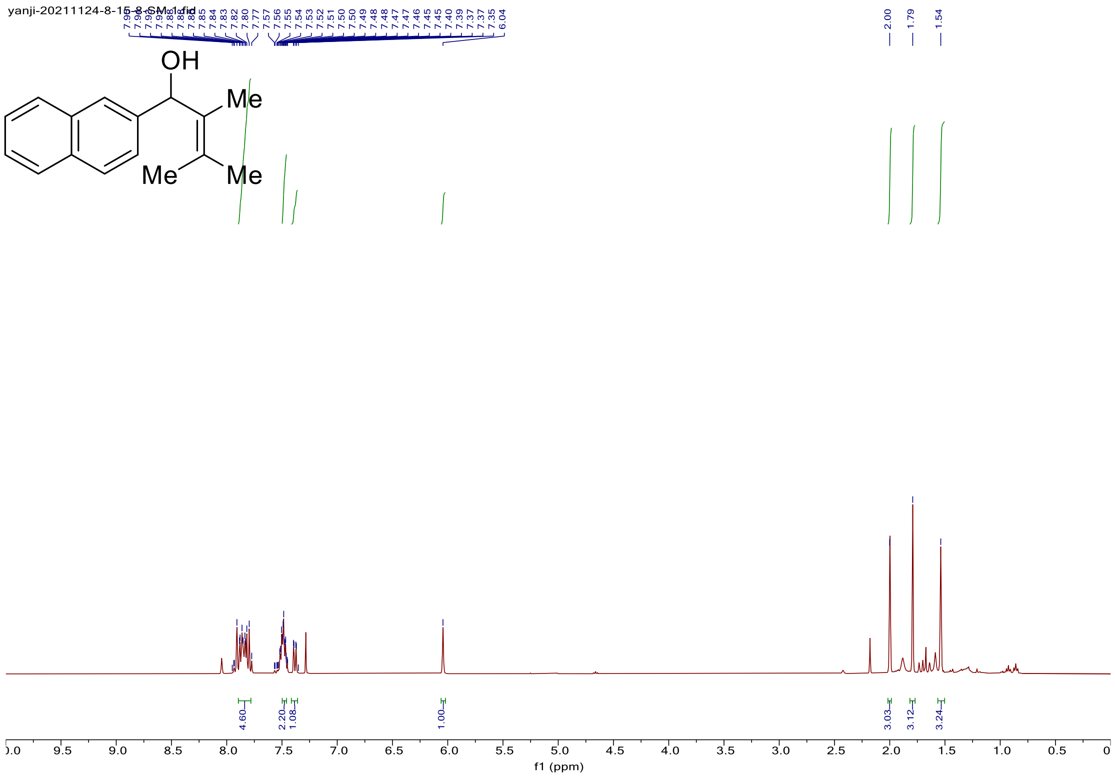

yanji-20211124-8-15-8-SM.1.fid
7.96 7.94 7.93 7.91 7.88 7.86 7.86 7.85 7.84 7.83 7.82 7.80 7.77 7.57 7.56 7.55 7.54 7.53 7.52 7.51 7.50 7.50 7.49 7.48 7.48 7.47 7.47 7.46 7.45 7.45 7.40 7.39 7.37 7.37 7.35 6.04
2.00
1.79
1.54
OH
Me
Me
Me
4.60
2.20
1.08
1.00
3.03
3.12
3.24
10.0 9.5 9.0 8.5 8.0 7.5 7.0 6.5 6.0 5.5 5.0 4.5 4.0 3.5 3.0 2.5 2.0 1.5 1.0 0.5 0
f1 (ppm)

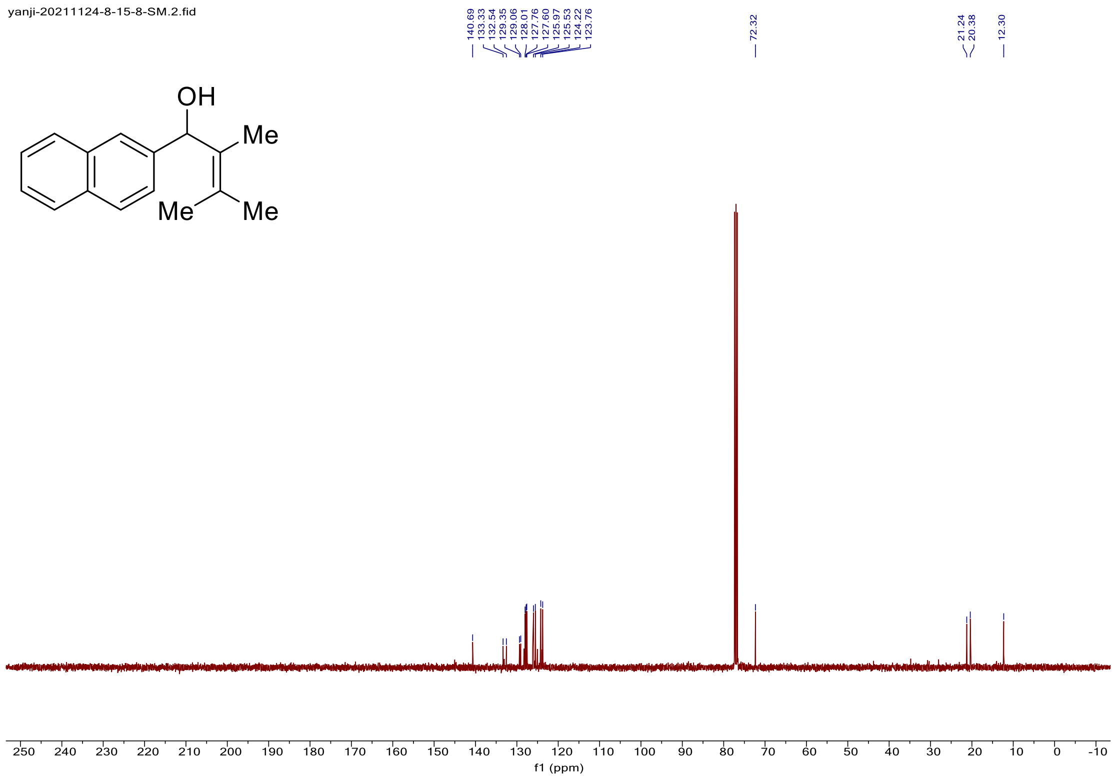
yanji-20211124-8-15-8-SM.2.fid
140.69
133.33
132.54
129.35
129.06
128.01
127.76
127.60
125.97
125.53
124.22
123.76
72.32
21.24
20.38
12.30
OH
Me
Me
Me
f1 (ppm)

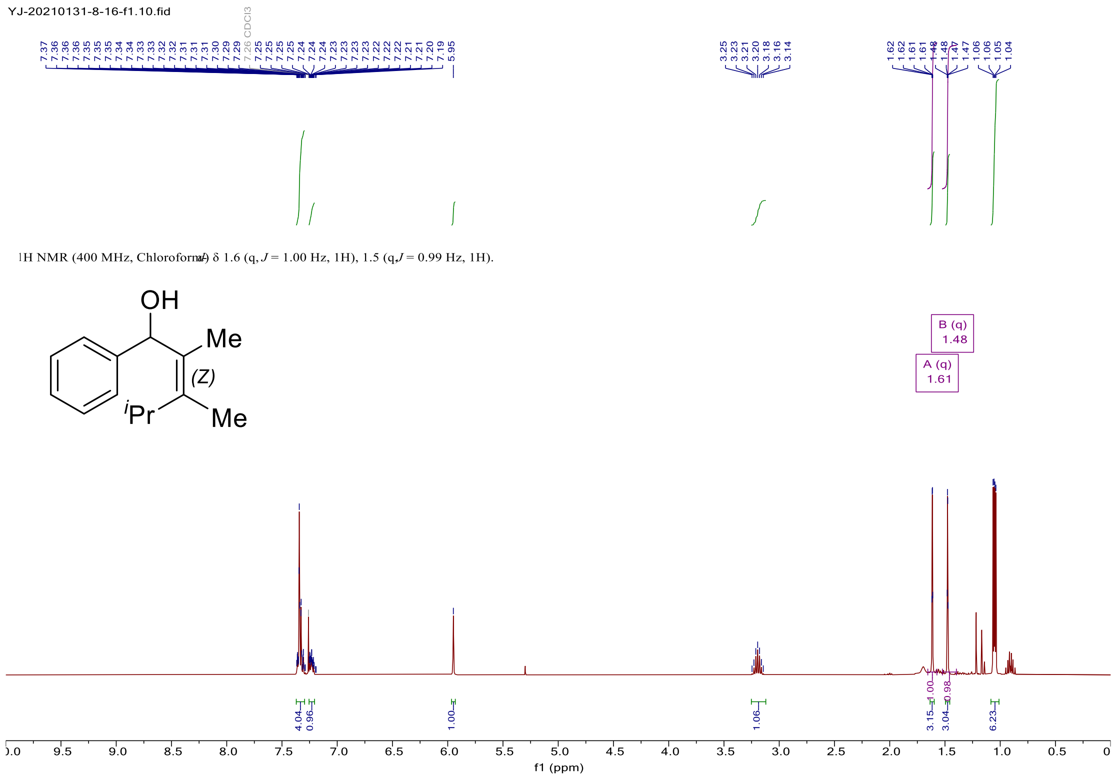
YJ-20210131-8-16-f1.10.fid
7.26 CDCl3
7.37 7.36 7.36 7.35 7.35 7.35 7.34 7.33 7.33 7.32 7.32 7.31 7.31 7.30 7.29 7.29 7.25 7.25 7.25 7.24 7.24 7.24 7.23 7.23 7.23 7.22 7.22 7.22 7.21 7.20 7.19
5.95
3.25 3.23 3.21 3.20 3.18 3.16 3.14
1.62 1.62 1.61 1.48 1.48 1.47 1.47 1.06 1.05 1.04
1H NMR (400 MHz, Chloroform-d) δ 1.6 (q, J = 1.00 Hz, 1H), 1.5 (q, J = 0.99 Hz, 1H).
OH
Me
(Z)
iPr
Me
B (q)
1.48
A (q)
1.61
4.04
0.96
1.00
1.06
3.15
1.00
3.04
0.98
6.23
f1 (ppm)

YJ-20210131-8-16-f1.12.fid

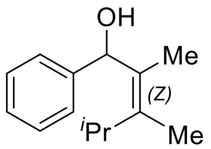


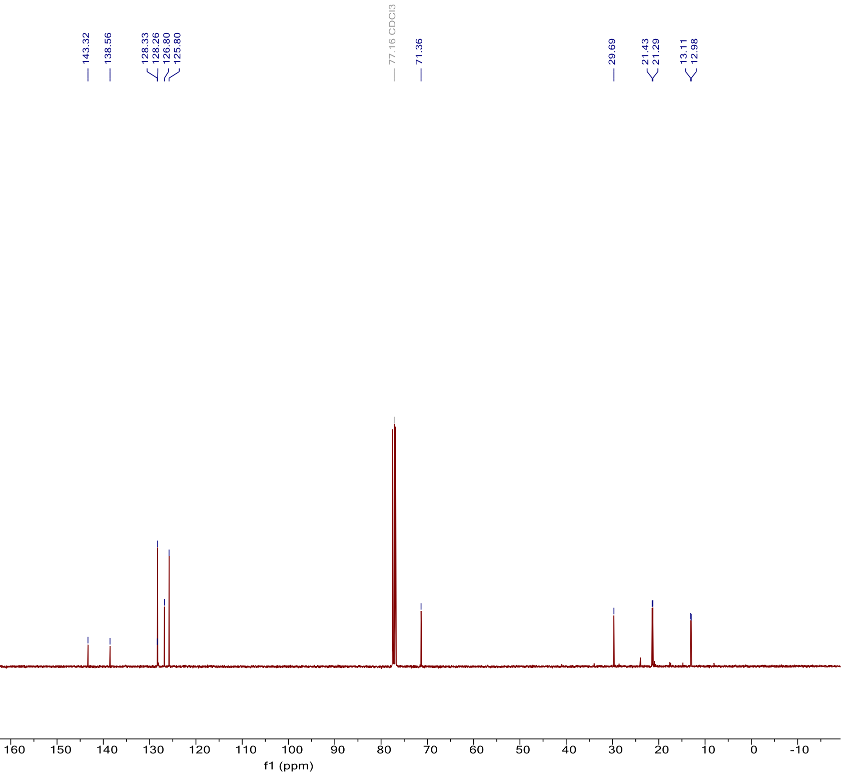

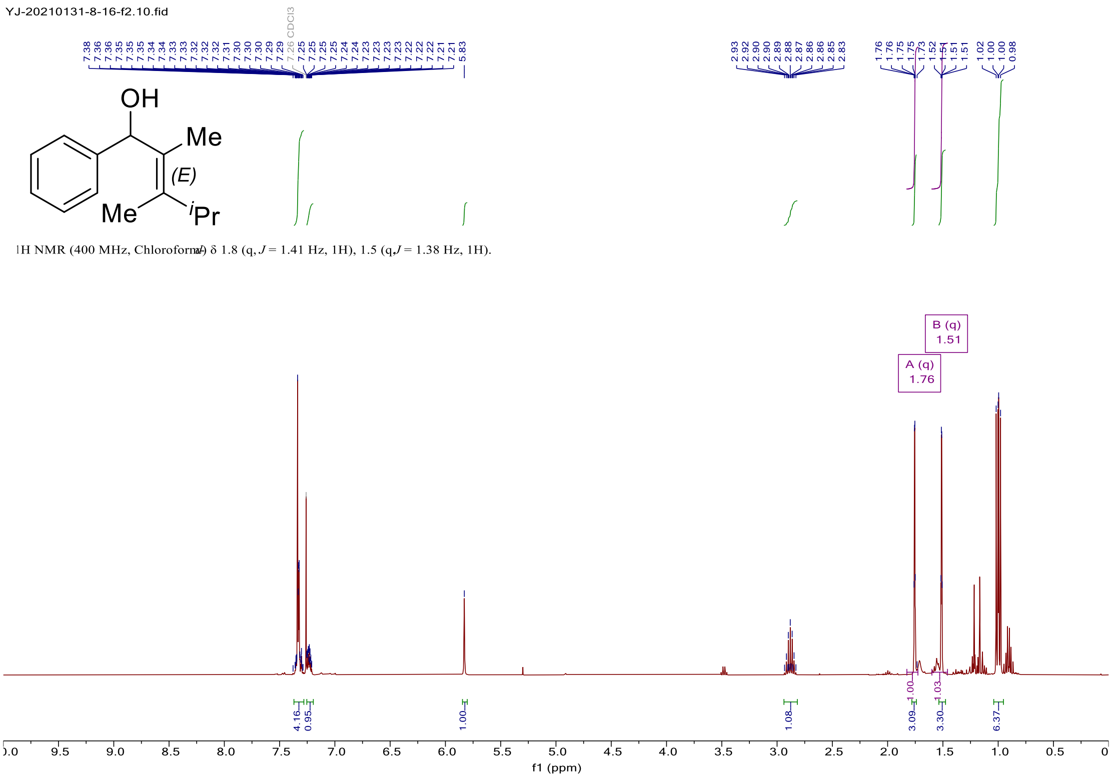
YJ-20210131-8-16-f2.10.fid
OH
Me
(E)
Me
iPr
1H NMR (400 MHz, Chloroform-d) δ 1.8 (q, J = 1.41 Hz, 1H), 1.5 (q, J = 1.38 Hz, 1H).
B (q) 1.51
A (q) 1.76
f1 (ppm)

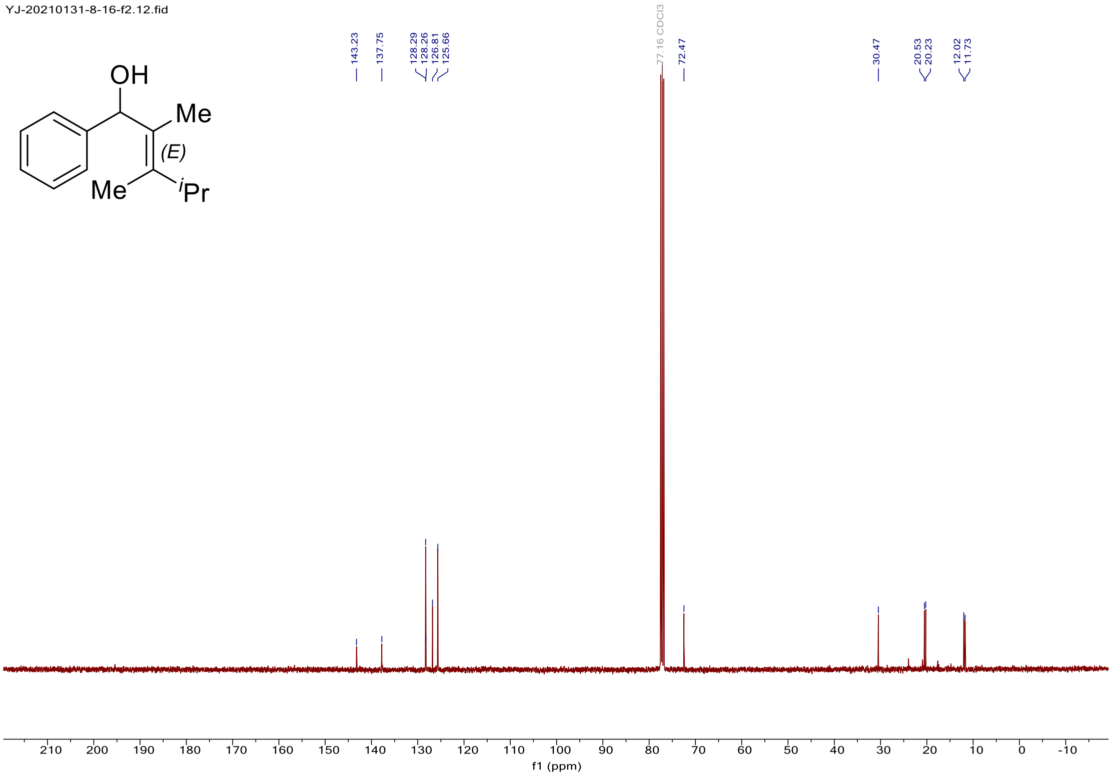
YJ-20210131-8-16-f2.12.fid
OH
Me
(E)
Me
iPr
143.23
137.75
128.29
128.26
126.81
125.66
77.16 CDCl3
72.47
30.47
20.53
20.23
12.02
11.73
f1 (ppm)

YJ-20211229-8-15-20-SM.10.fid

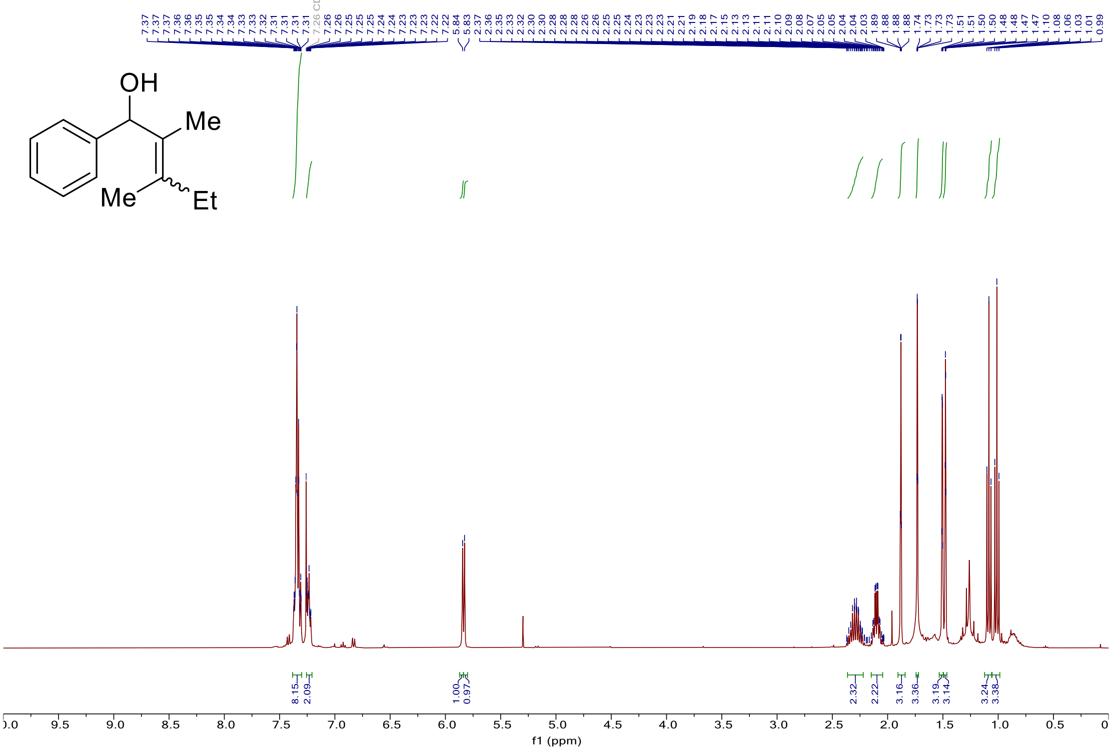

YJ-20211229-8-15-20-SM.11.fid

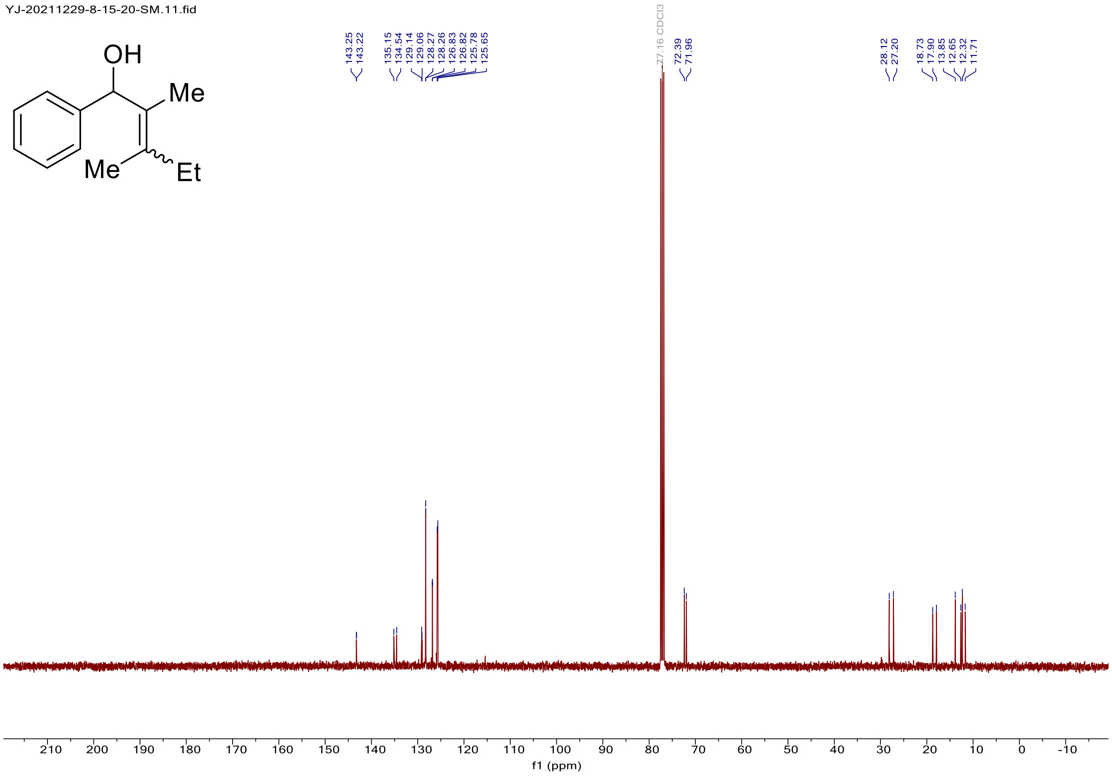

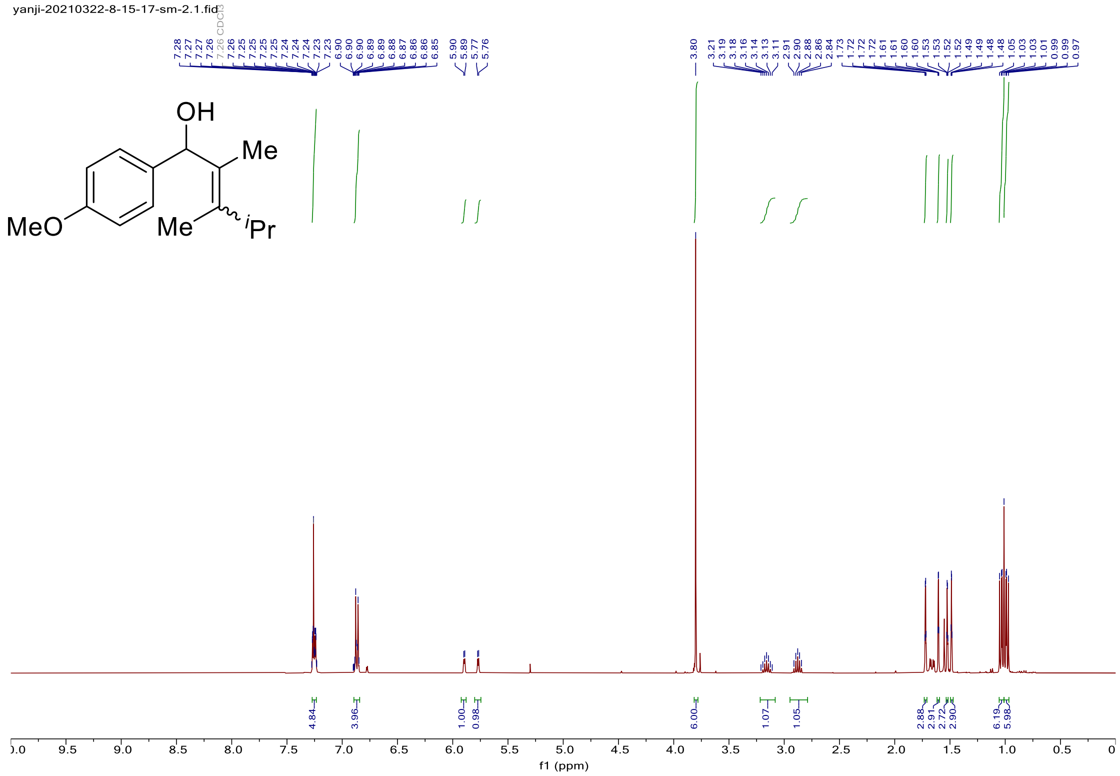
yanji-20210322-8-15-17-sm-2.1.fid
OH
Me
MeO
Me
iPr
7.28 7.27 7.27 7.26 7.26 CDCl3 7.26 7.25 7.25 7.25 7.25 7.24 7.24 7.24 7.23 7.23 6.90 6.90 6.89 6.89 6.88 6.87 6.86 6.86 6.85 5.90 5.89 5.77 5.76
3.80 3.21 3.19 3.18 3.16 3.14 3.13 3.11 2.91 2.90 2.88 2.86 2.84 1.73 1.72 1.72 1.61 1.61 1.60 1.60 1.53 1.53 1.52 1.52 1.49 1.48 1.48 1.05 1.03 1.03 1.01 0.99 0.99 0.97
4.84 3.96 1.00 0.98 6.00 1.07 1.05 2.88 2.91 2.72 2.90 6.19 5.98
f1 (ppm)

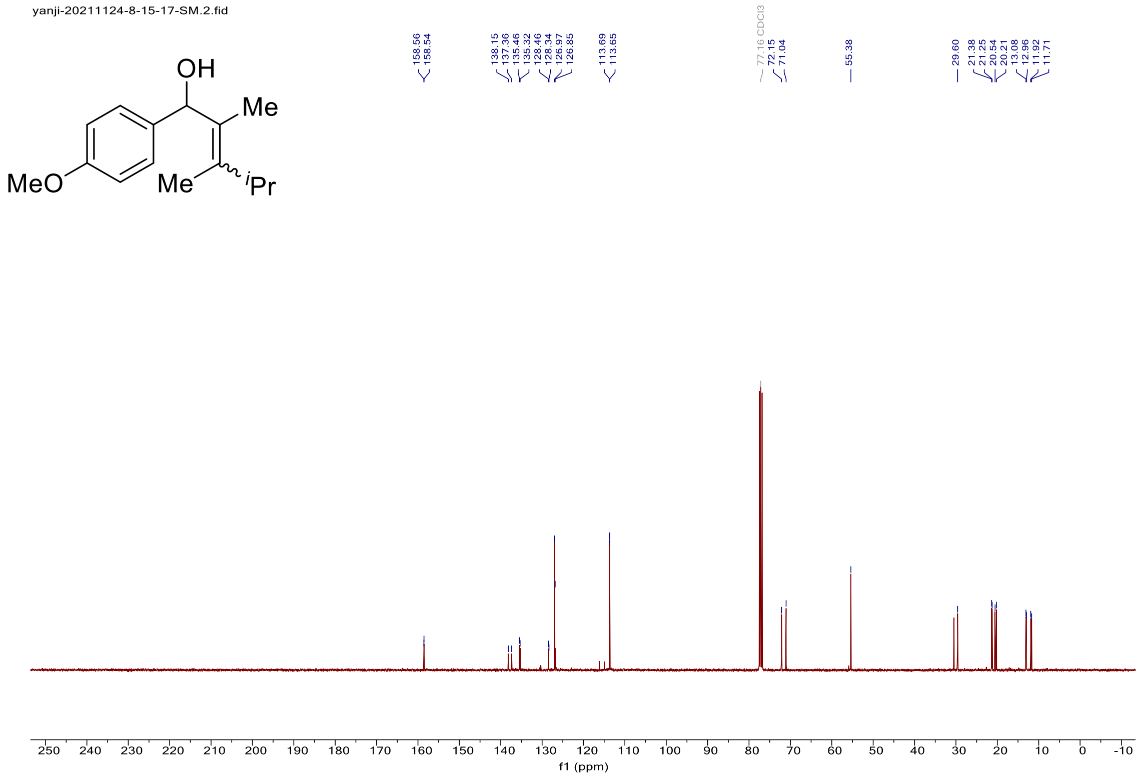
yanji-20211124-8-15-17-SM.2.fid
OH
Me
MeO
Me
iPr
158.56 158.54 138.15 137.36 135.46 135.32 128.46 128.34 126.97 126.85 113.69 113.65
77.16 CDCl3 72.15 71.04 55.38 29.60 21.38 21.25 20.54 20.21 13.08 12.96 11.92 11.71
f1 (ppm)

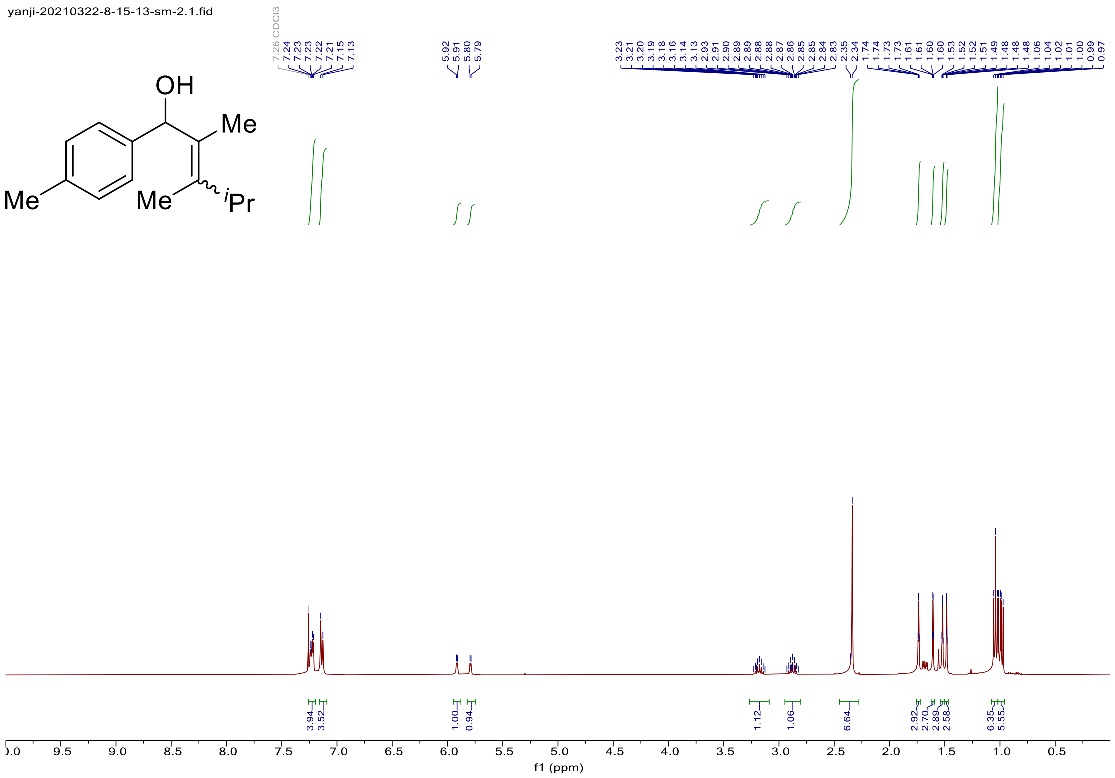
yanji-20210322-8-15-13-sm-2.1.fid
OH
Me
Me
Me
iPr
7.26 CDCl3
7.24
7.23
7.23
7.22
7.21
7.15
7.13
5.92
5.91
5.80
5.79
3.94
3.52
1.00
0.94
1.12
1.06
6.64
2.92
2.70
2.89
2.58
6.35
5.55
f1 (ppm)

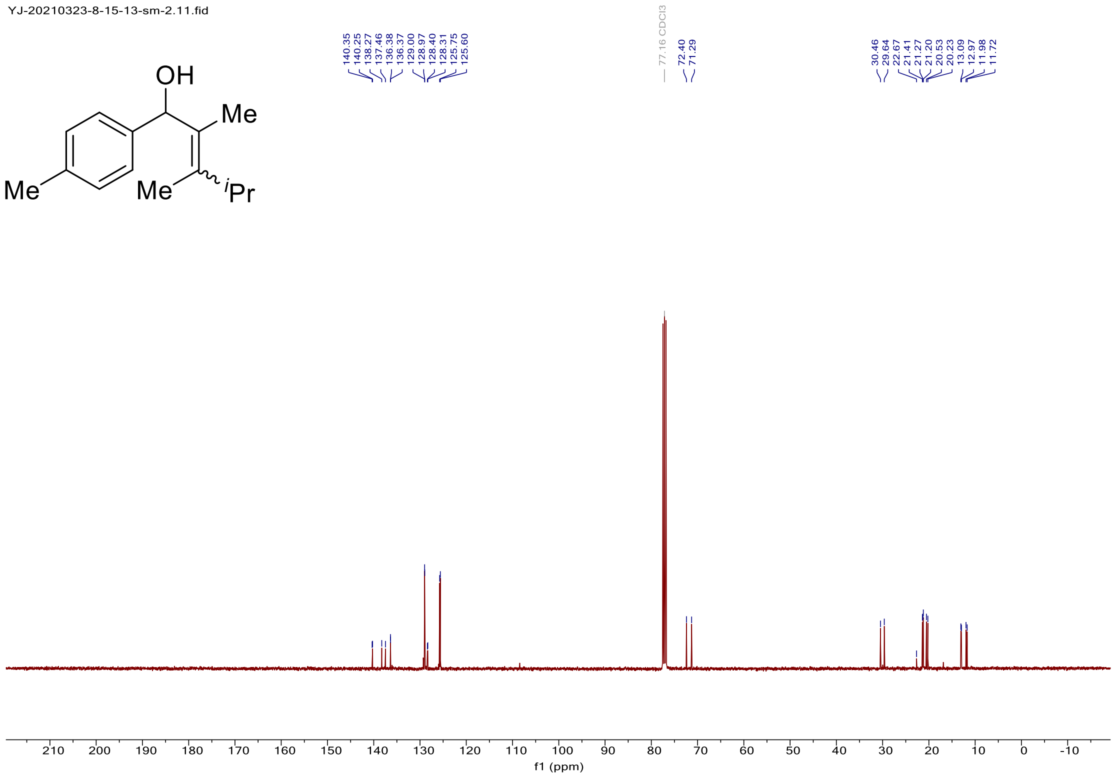
YJ-20210323-8-15-13-sm-2.11.fid
OH
Me
Me
Me
iPr
140.35
140.25
138.27
137.46
136.38
136.37
129.00
128.97
128.40
128.31
125.75
125.60
77.16 CDCl3
72.40
71.29
30.46
29.64
22.67
21.41
21.27
21.20
20.53
20.23
13.09
12.97
11.98
11.72
f1 (ppm)

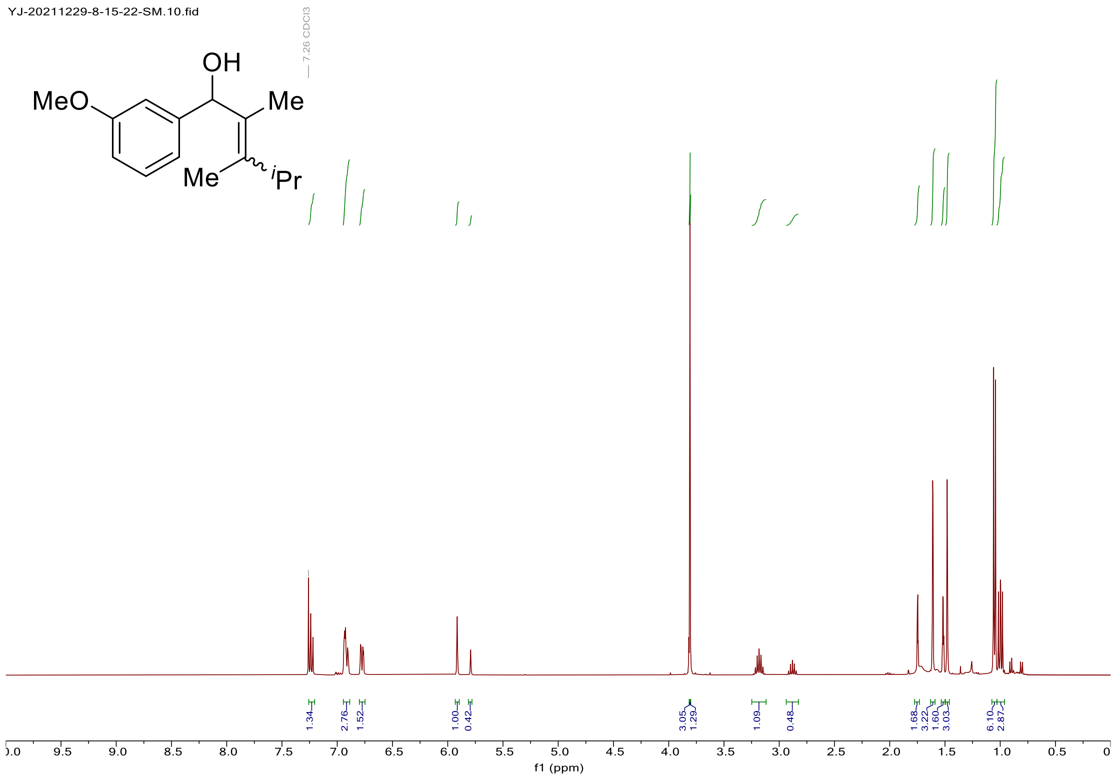
YJ-20211229-8-15-22-SM.10.fid
OH
MeO
Me
Me
iPr
7.26 CDCl3
1.34
2.76
1.52
1.00
0.42
3.05
1.29
1.09
0.48
1.68
3.22
1.60
3.03
6.10
2.87
f1 (ppm)

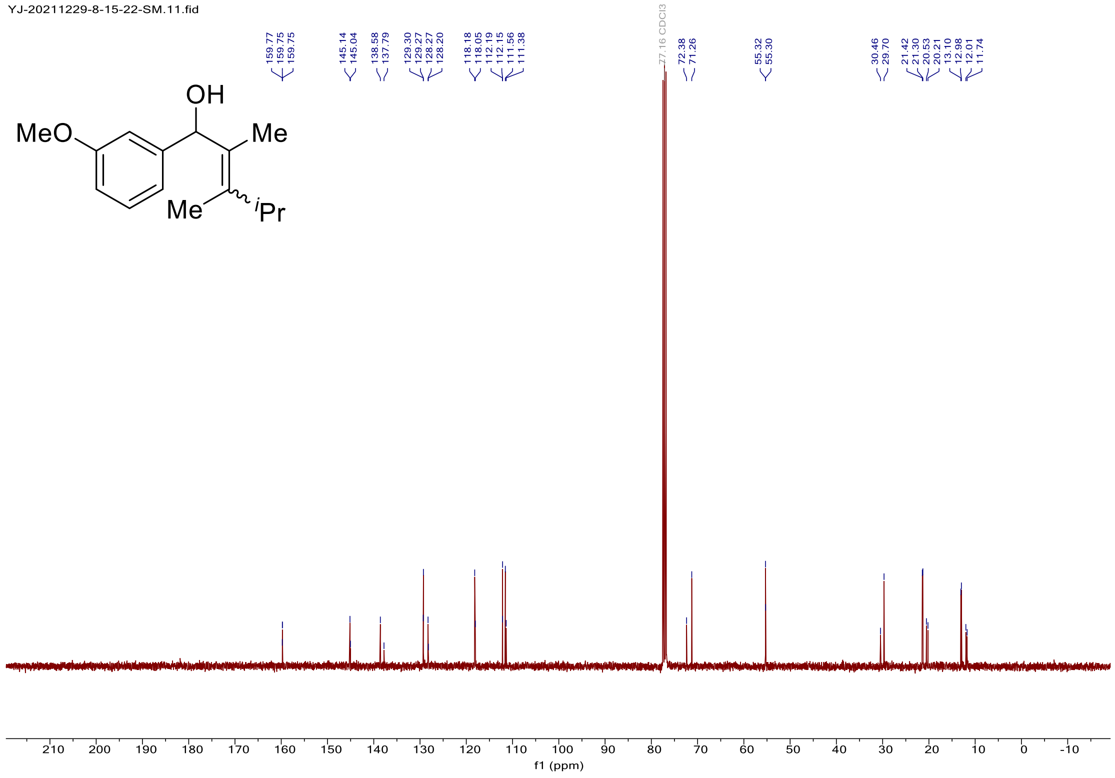
YJ-20211229-8-15-22-SM.11.fid
159.77
159.75
159.75
145.14
145.04
138.58
137.79
129.30
129.27
128.27
128.20
118.18
118.05
112.19
112.15
111.56
111.38
77.16 CDCl3
72.38
71.26
55.32
55.30
30.46
29.70
21.42
21.30
20.53
20.21
13.10
12.98
12.01
11.74
OH
MeO
Me
Me
iPr
f1 (ppm)

yanji-20210331-8-15-23-sm-2.1.fid

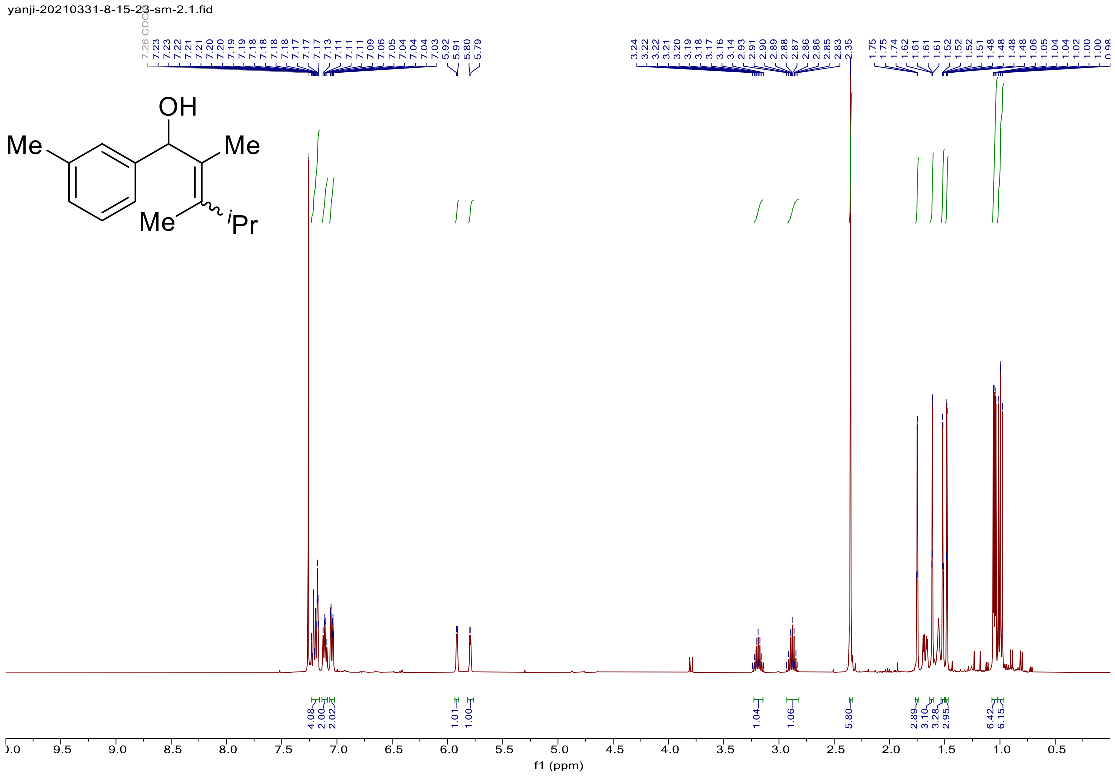


yanji-20220107-8-15-23-SM-2.2.fid

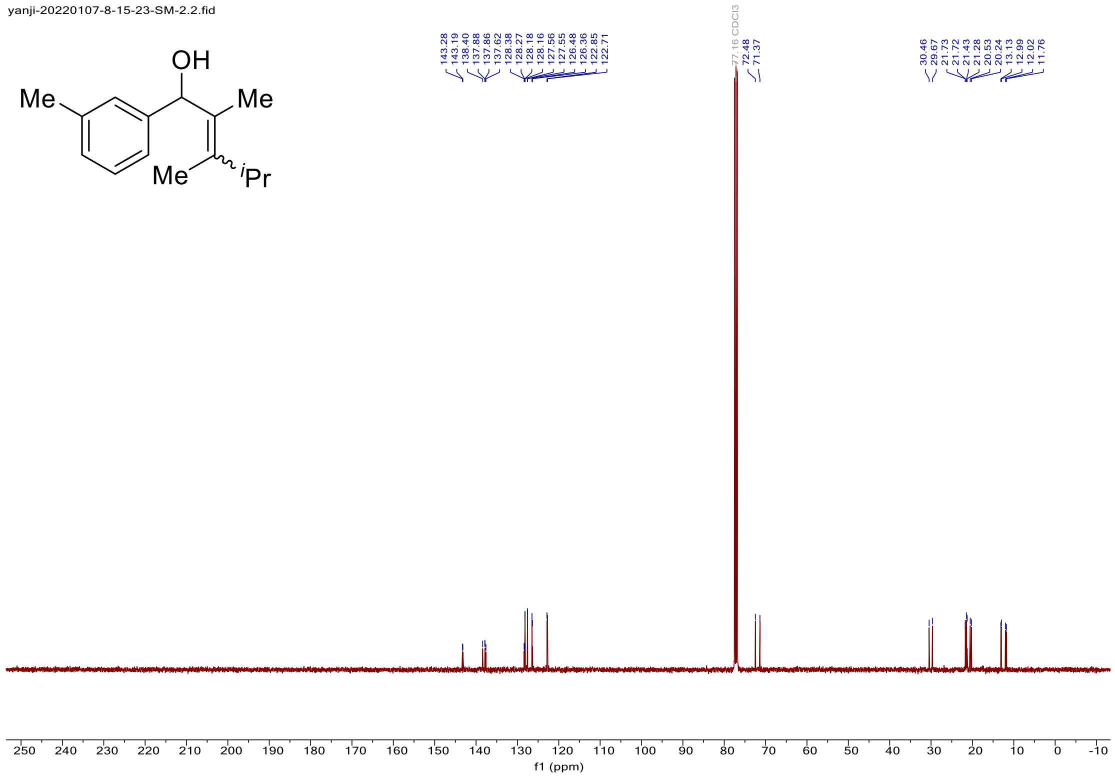

YJ-20210318-8-15-16-sm.10.fid

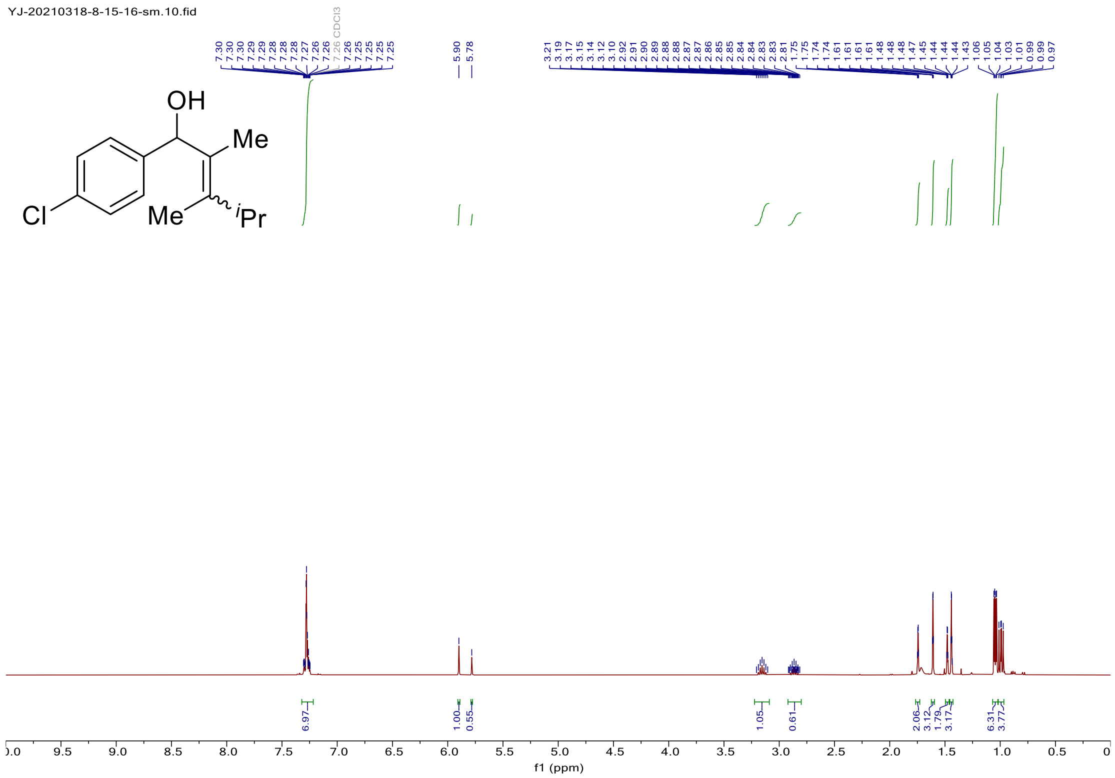


YJ-20210318-8-15-16-sm.12.fid

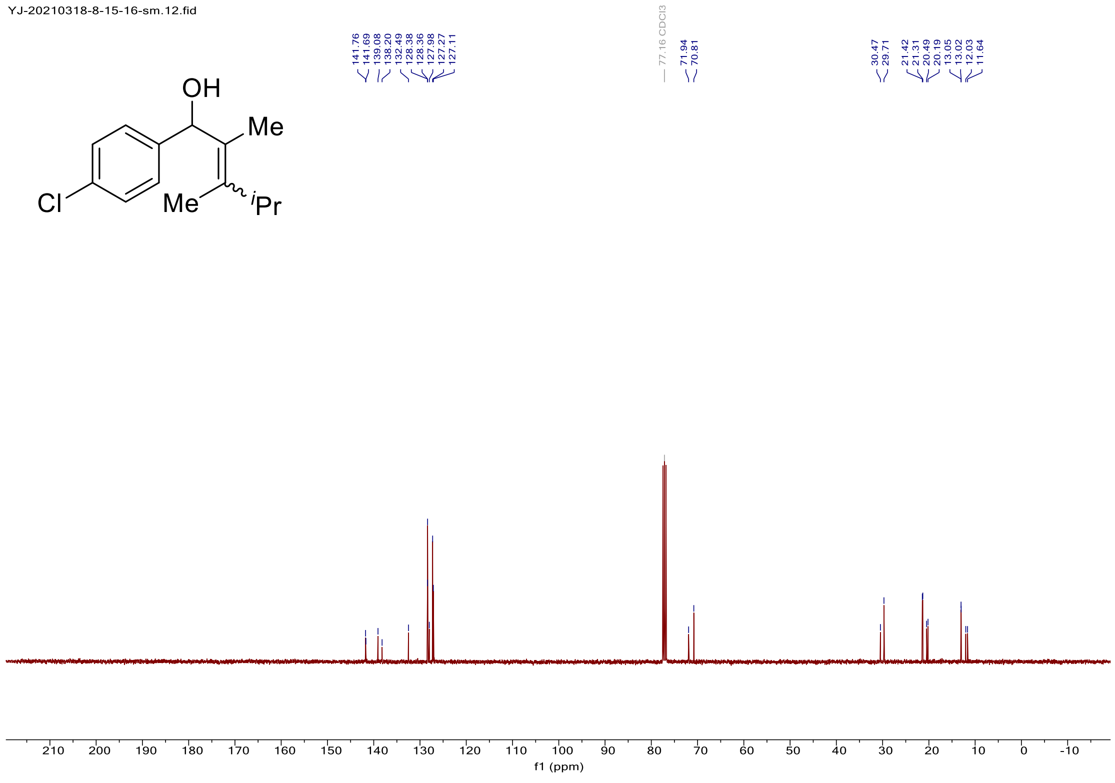

yanji-20211229-8-15-12-SM.1.fid

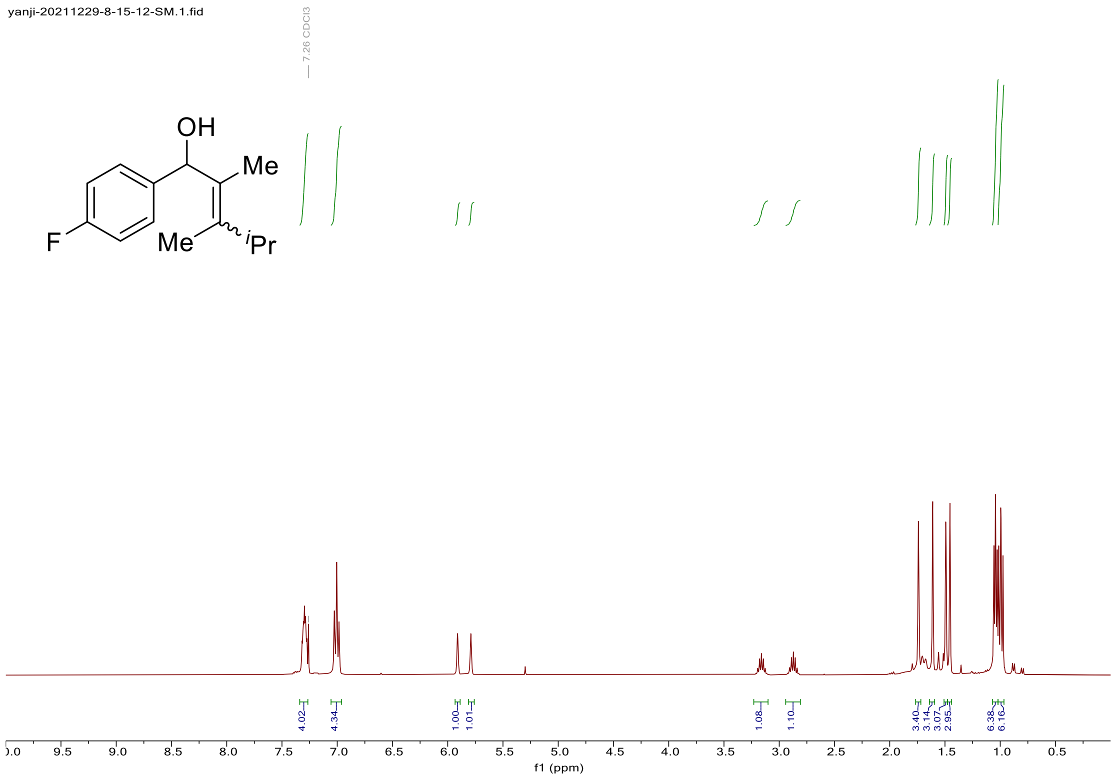

yanji-20211229-8-15-12-SM.3.fid

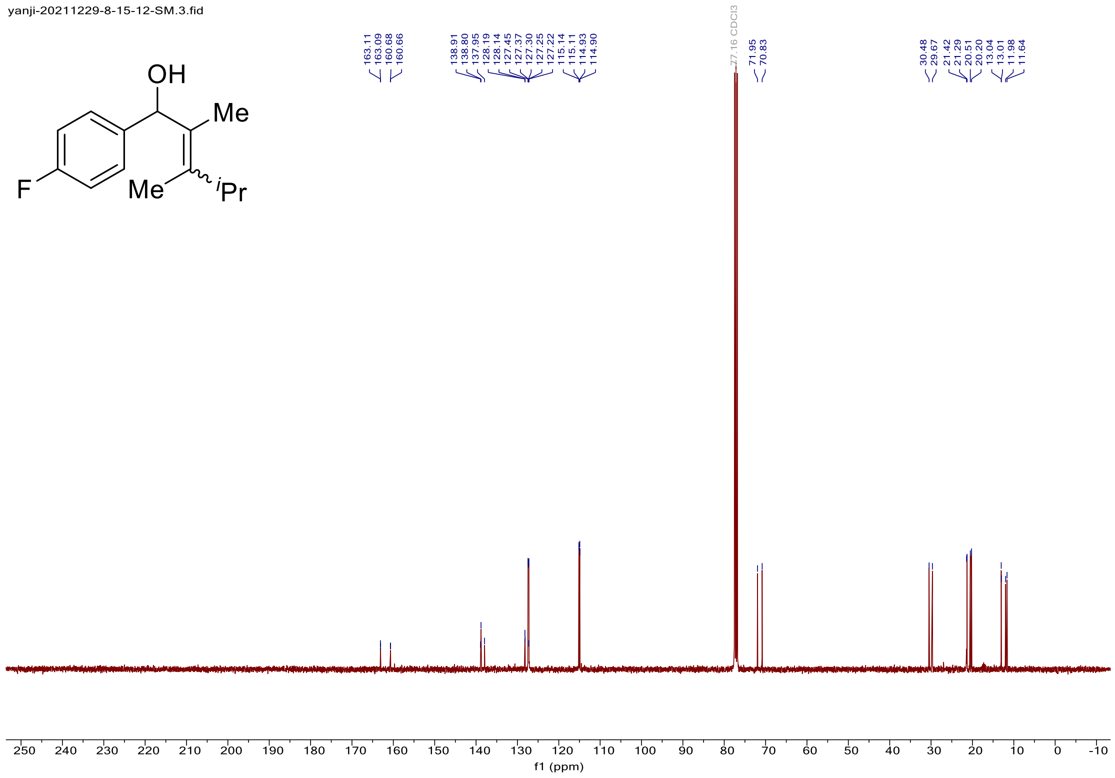

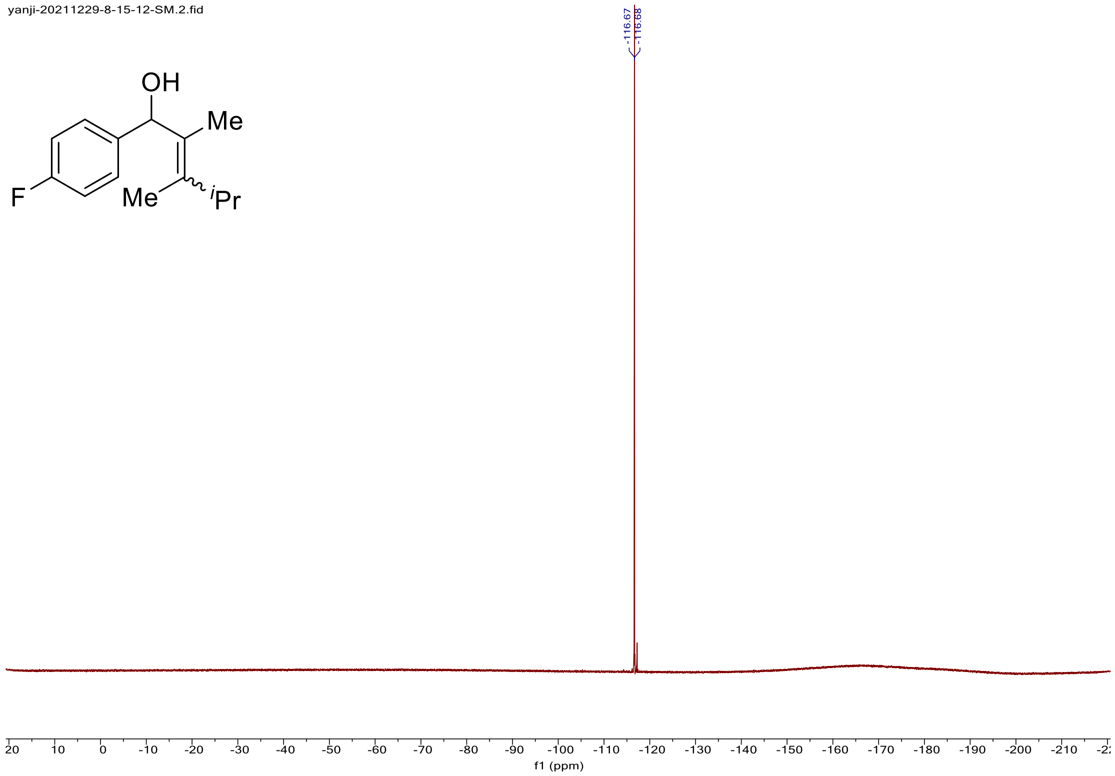
yanji-20211229-8-15-12-SM.2.fid
OH
Me
F
Me
iPr
-116.67
-116.68
f1 (ppm)

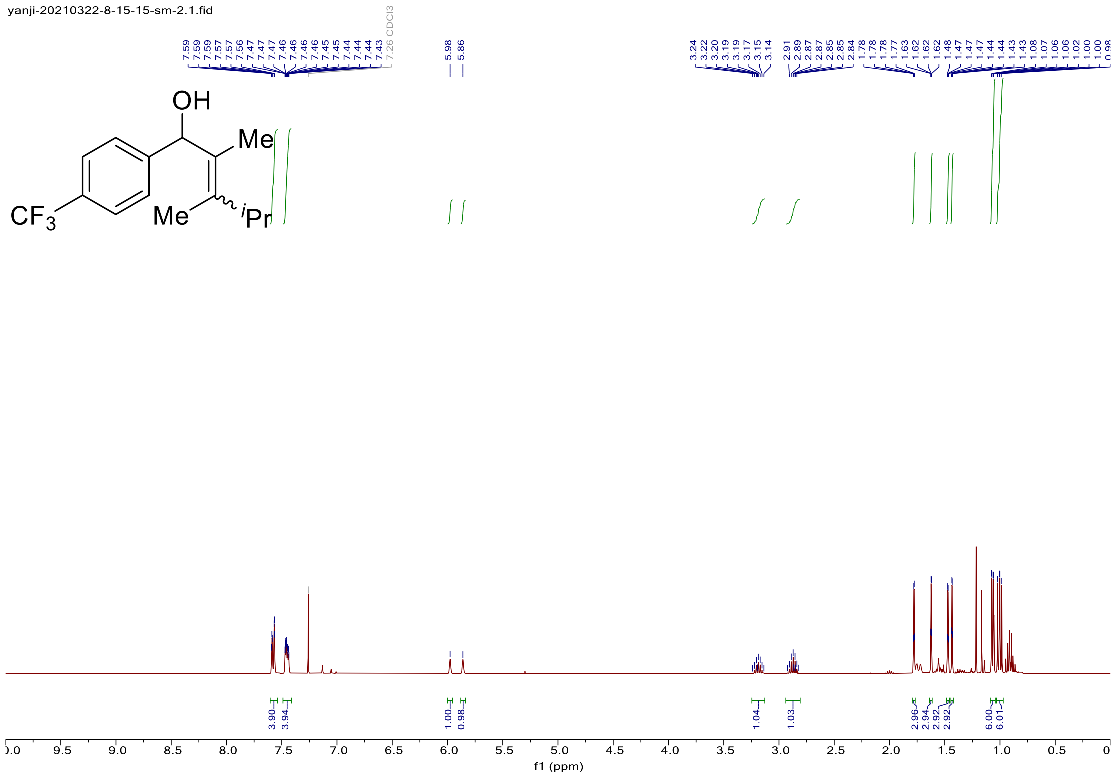
yanji-20210322-8-15-15-sm-2.1.fid
OH
Me
CF3
Me
iPr
7.26 CDCl3
f1 (ppm)

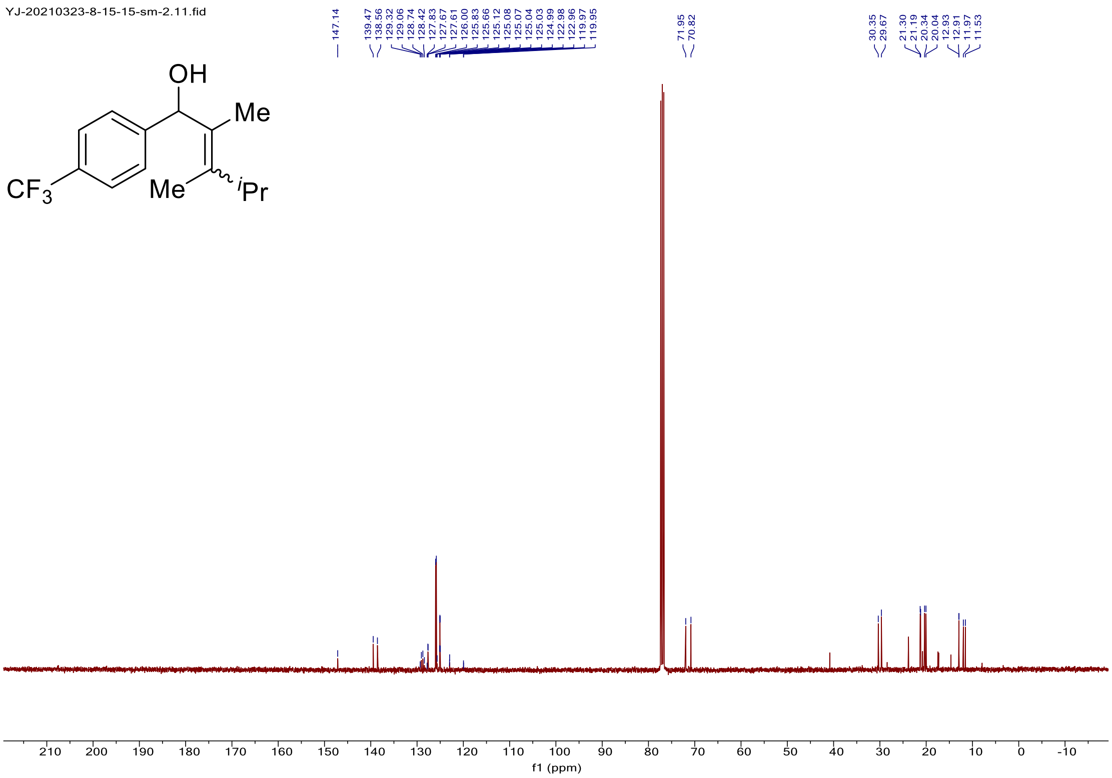
YJ-20210323-8-15-15-sm-2.11.fid
147.14
139.47
138.56
129.32
129.06
128.74
128.42
127.83
127.67
127.61
126.00
125.83
125.66
125.12
125.08
125.07
125.04
125.03
124.99
122.98
122.86
119.97
119.95
71.95
70.82
30.35
29.67
21.30
21.19
20.34
20.04
12.93
12.91
11.97
11.53
OH
Me
CF3
Me
iPr
f1 (ppm)

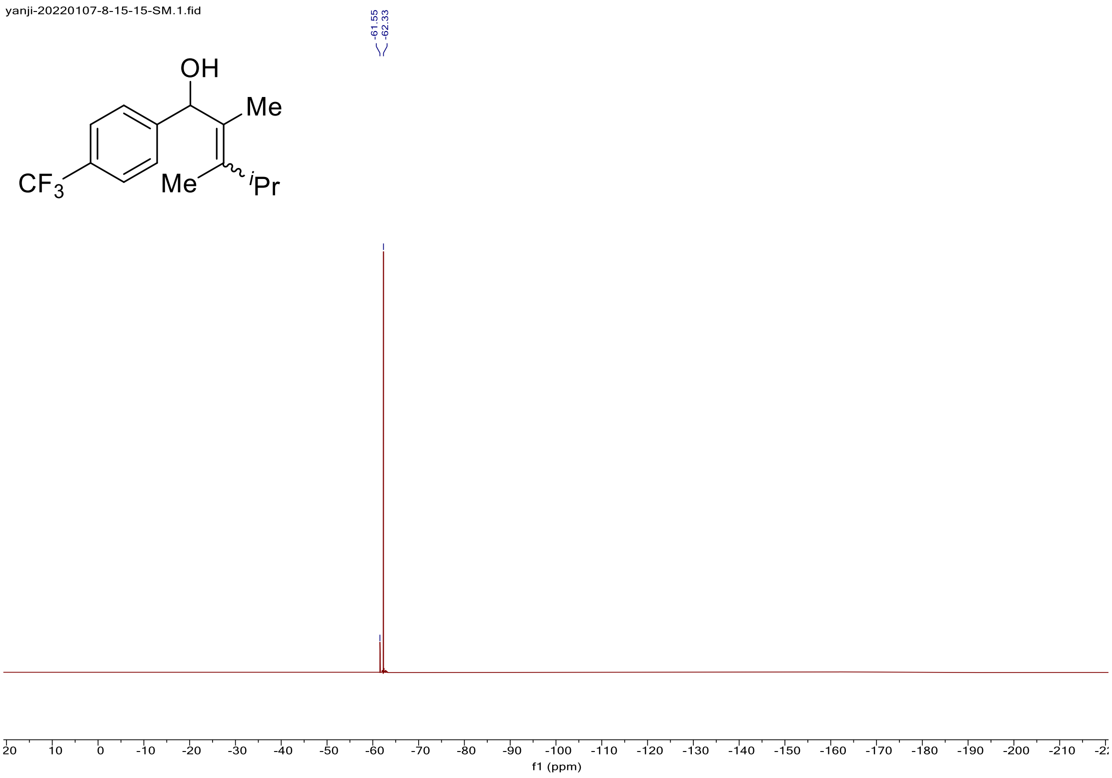
yanji-20220107-8-15-15-SM.1.fid
-61.55
-62.33
OH
Me
CF3
Me
iPr
f1 (ppm)

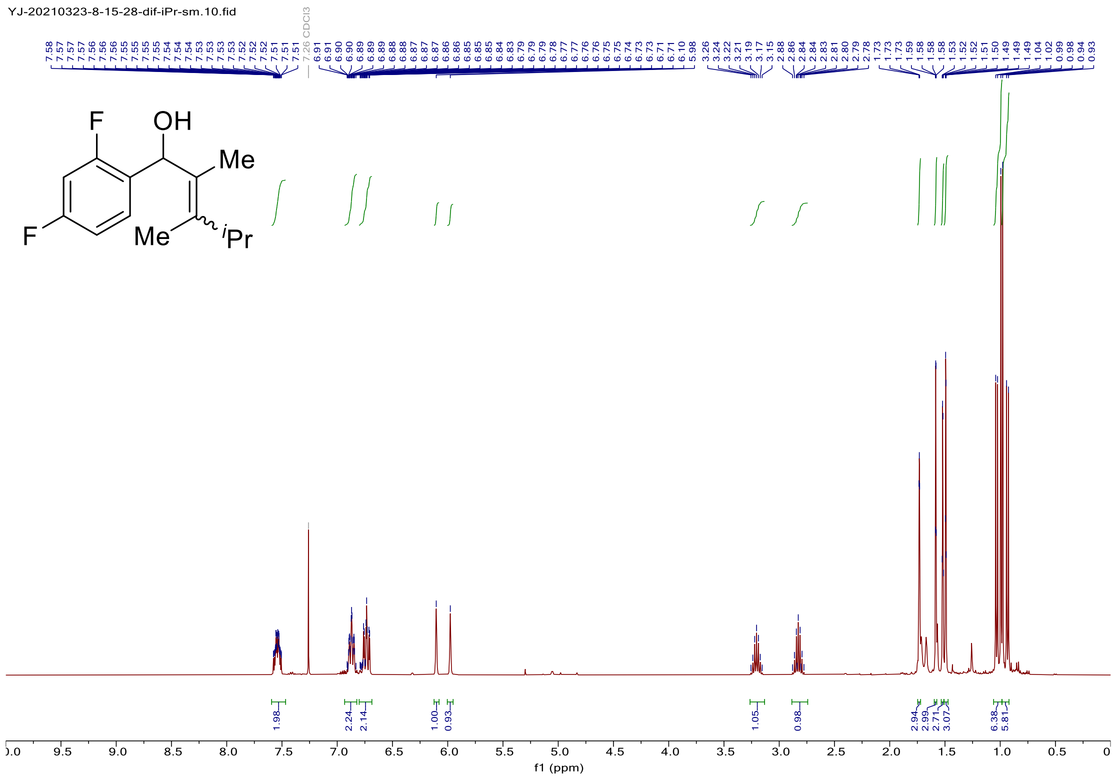
YJ-20210323-8-15-28-dif-iPr-sm.10.fid
F
OH
Me
F
Me
iPr
f1 (ppm)

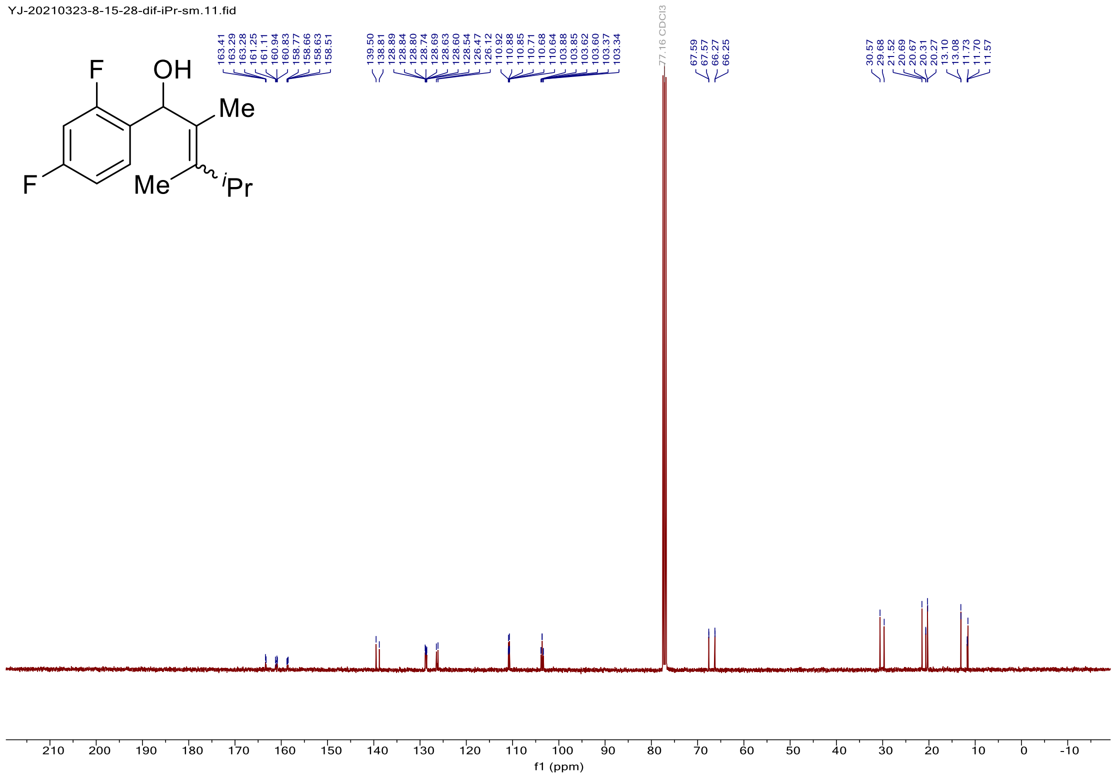
YJ-20210323-8-15-28-dif-iPr-sm.11.fid
F
OH
Me
F
Me
iPr
f1 (ppm)

yanji-20211124-8-15-28-SM.2.fid

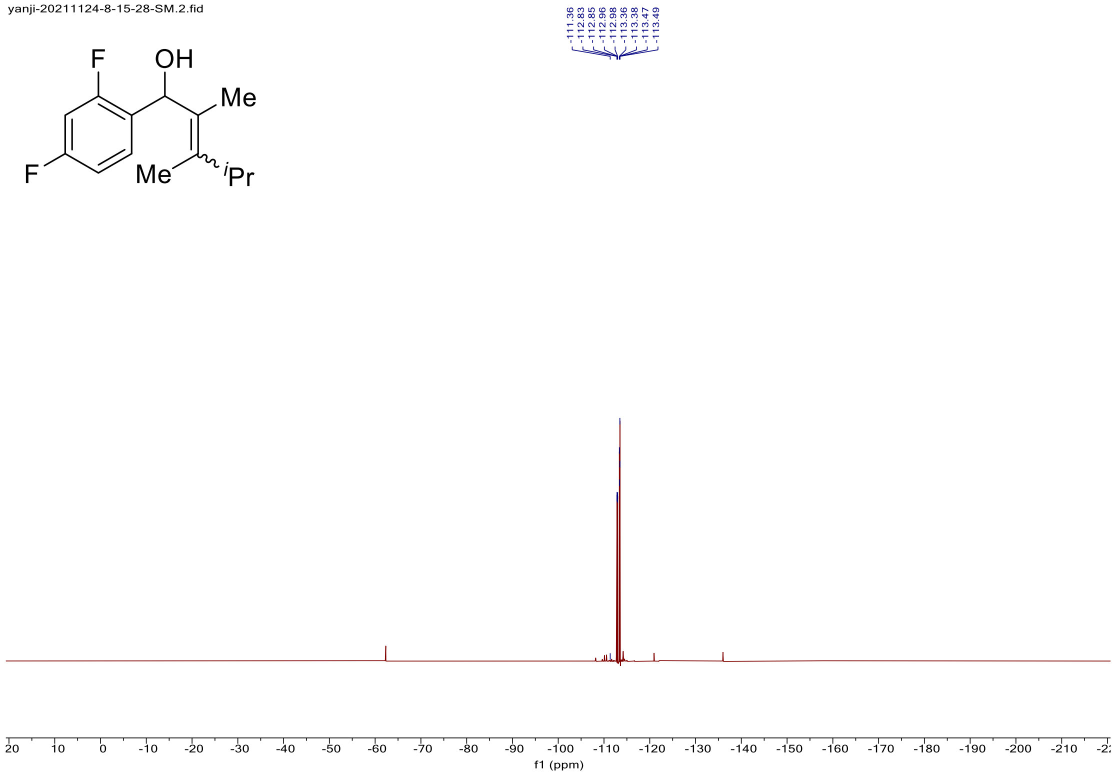

yanji-20211124-8-15-18-SM.2.fid

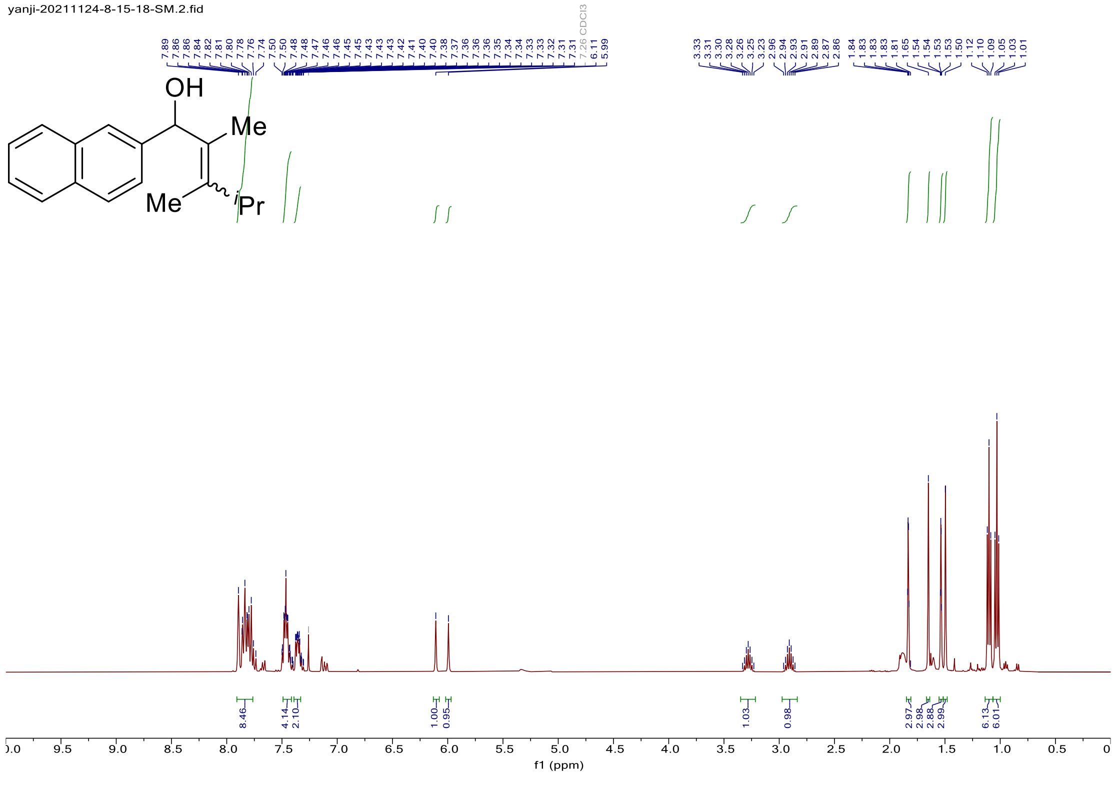

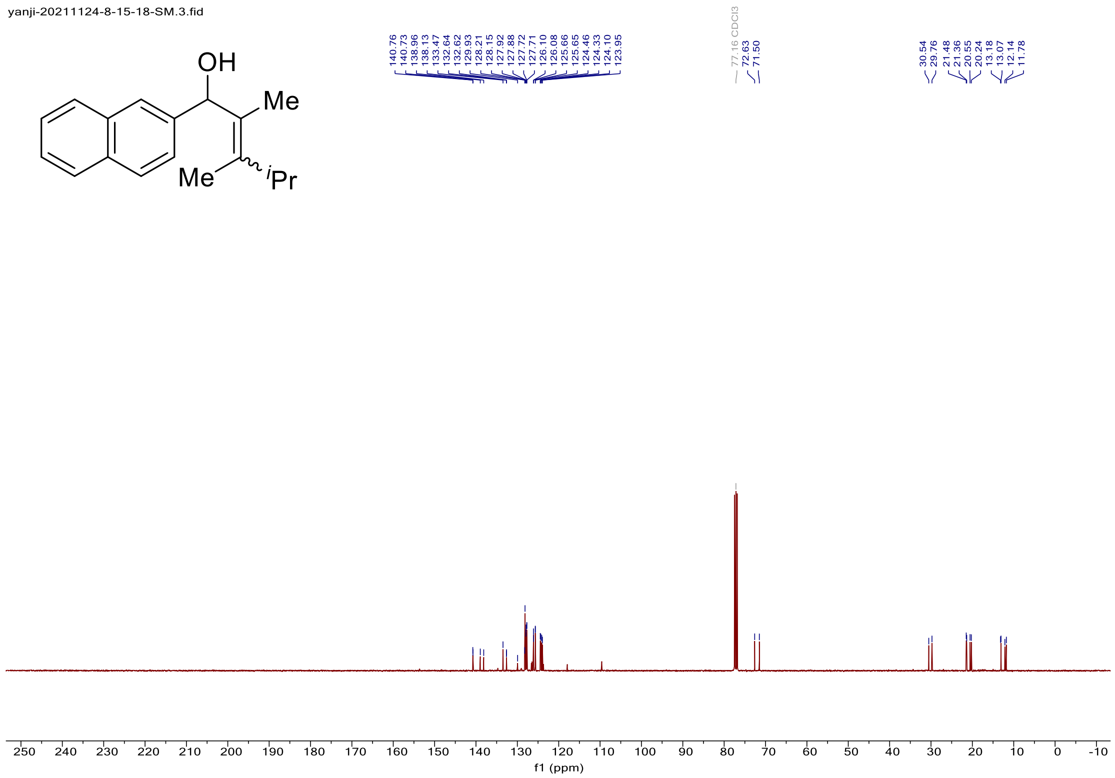
yanji-20211124-8-15-18-SM.3.fid
140.76
140.73
138.96
138.13
133.47
132.64
132.62
129.93
128.21
128.15
127.92
127.88
127.72
127.71
126.10
126.08
125.66
125.65
124.46
124.33
124.10
123.95
77.16 CDCl3
72.63
71.50
30.54
29.76
21.48
21.36
20.55
20.24
13.18
13.07
12.14
11.78
OH
Me
Me
iPr
250 240 230 220 210 200 190 180 170 160 150 140 130 120 110 100 90 80 70 60 50 40 30 20 10 0 -10
f1 (ppm)

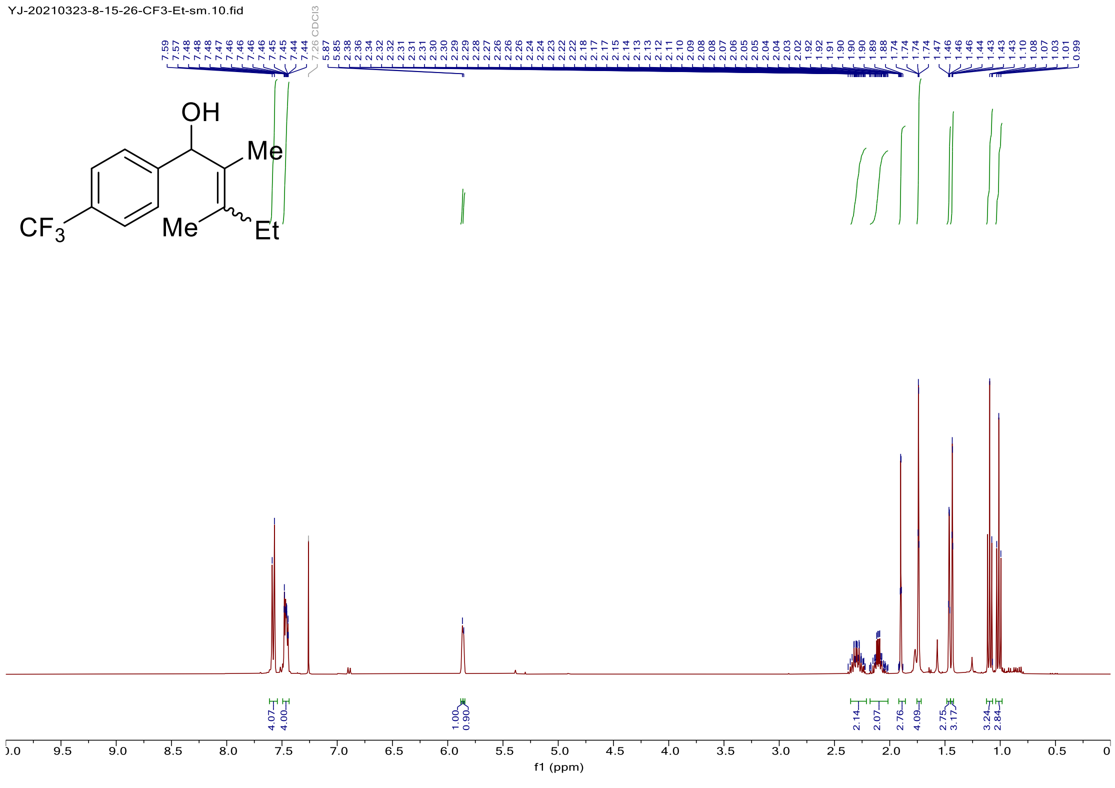
YJ-20210323-8-15-26-CF3-Et-sm.10.fid
7.59
7.57
7.48
7.48
7.47
7.46
7.46
7.46
7.45
7.45
7.44
7.44
7.26 CDCl3
5.87
5.85
2.36
2.34
2.32
2.32
2.31
2.31
2.30
2.30
2.29
2.29
2.28
2.27
2.26
2.26
2.24
2.24
2.23
2.22
2.22
2.18
2.17
2.17
2.15
2.14
2.13
2.13
2.12
2.11
2.10
2.09
2.08
2.08
2.07
2.06
2.05
2.05
2.04
2.04
2.03
2.02
2.02
1.92
1.91
1.90
1.90
1.90
1.89
1.88
1.74
1.74
1.74
1.74
1.47
1.46
1.46
1.44
1.43
1.43
1.10
1.08
1.07
1.03
1.01
0.99
OH
Me
CF3
Me
Et
4.07
4.00
1.00
0.90
2.14
2.07
2.76
4.09
2.75
3.17
3.24
2.84
9.5 9.0 8.5 8.0 7.5 7.0 6.5 6.0 5.5 5.0 4.5 4.0 3.5 3.0 2.5 2.0 1.5 1.0 0.5 0
f1 (ppm)

YJ-20210323-8-15-26-CF3-Et-sm.11.fid

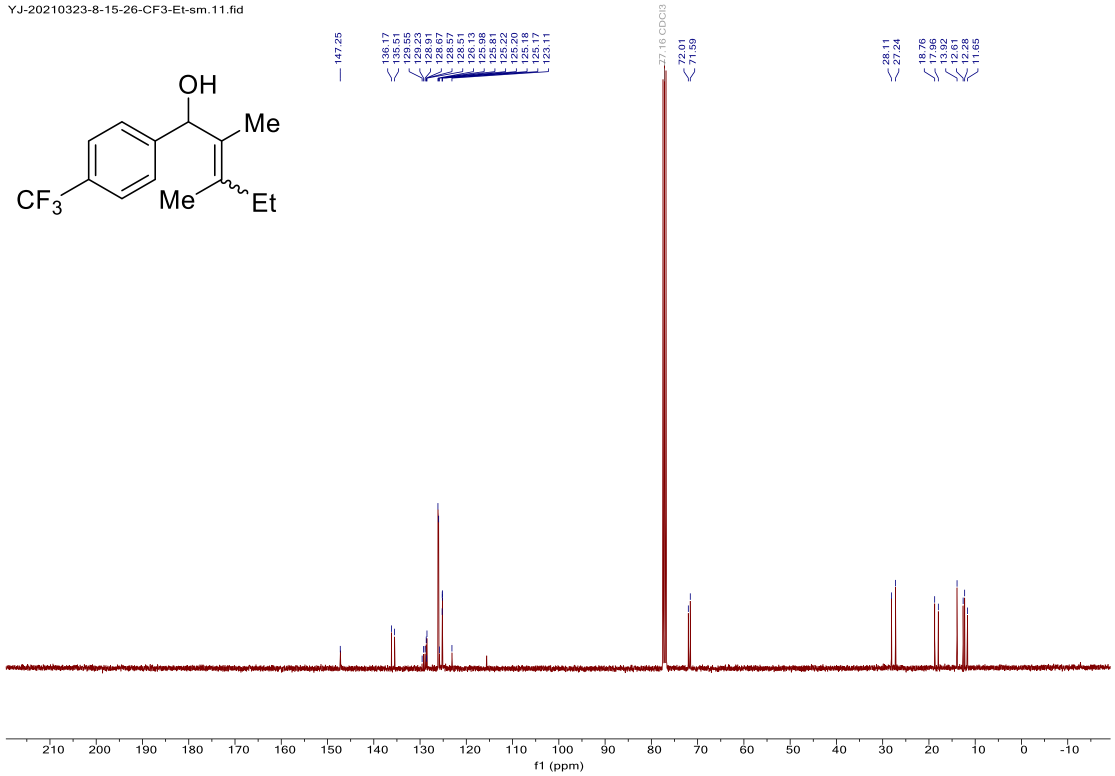

yanji-20220106-8-15-26-SM.2.fid

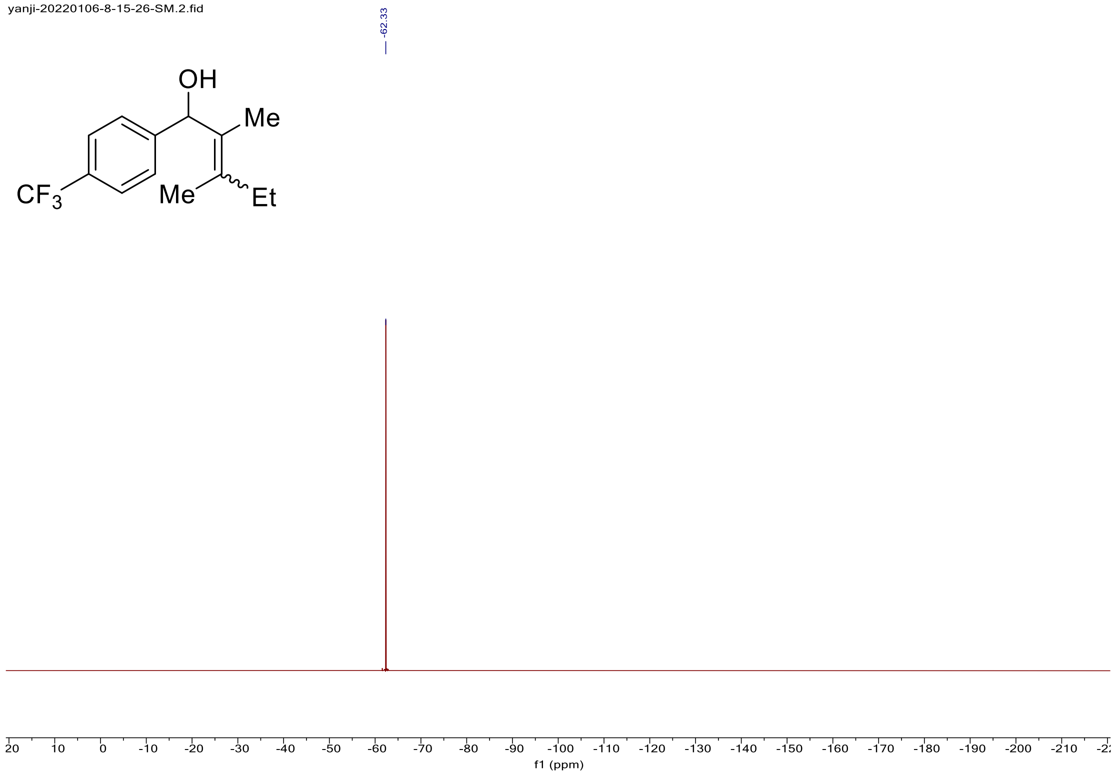

yanji-20220106-8-15-35-SM.1.fid

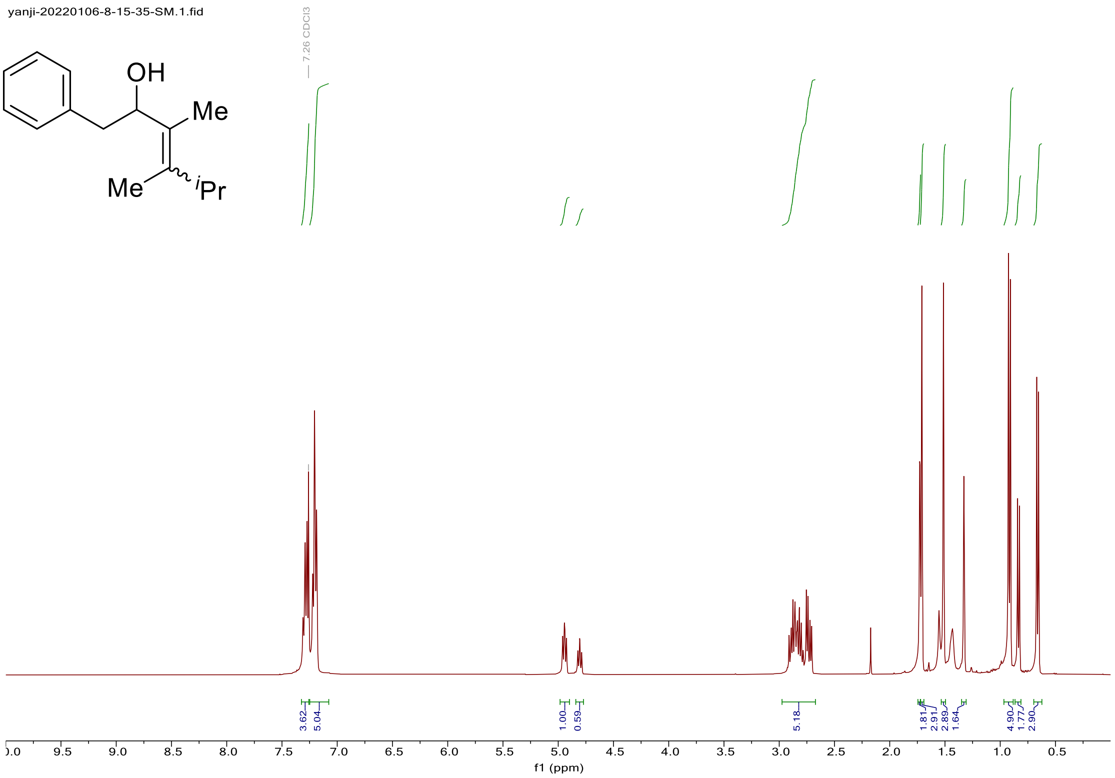


yanji-20220106-8-15-35-SM.2.fid

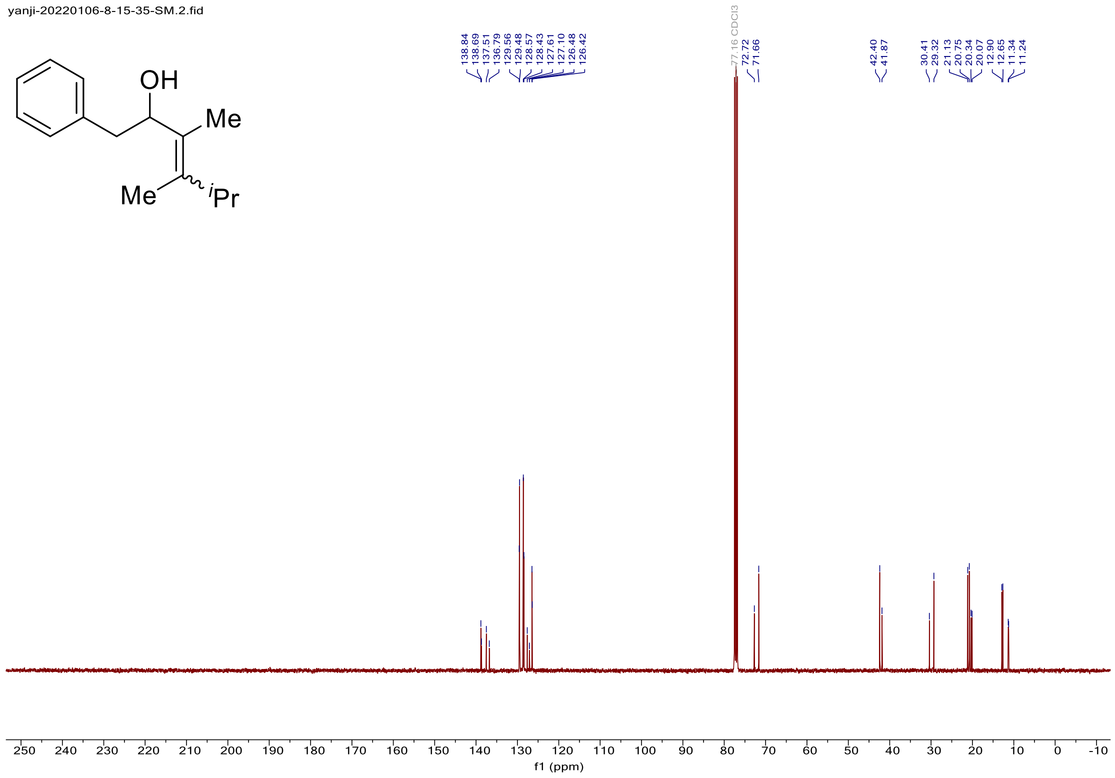

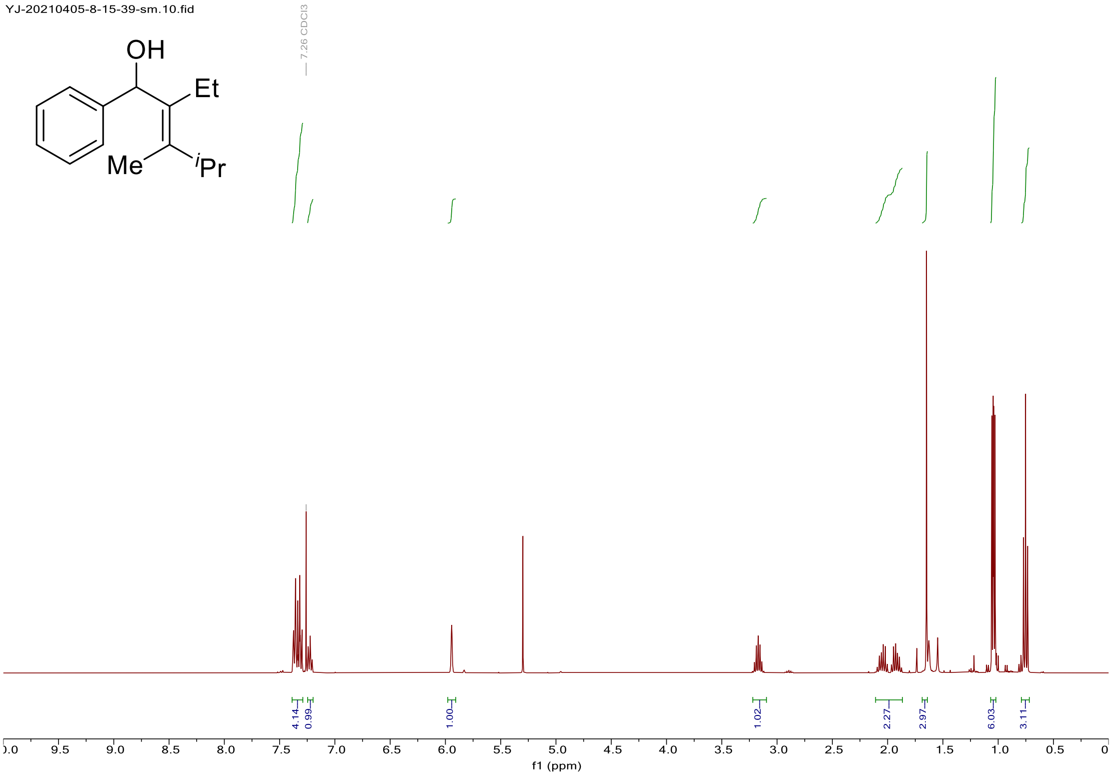

YJ-20210405-8-15-39-sm.10.fid
OH
Et
Me
iPr
7.26 CDCl3
4.14
0.99
1.00
1.02
2.27
2.97
6.03
3.11
f1 (ppm)


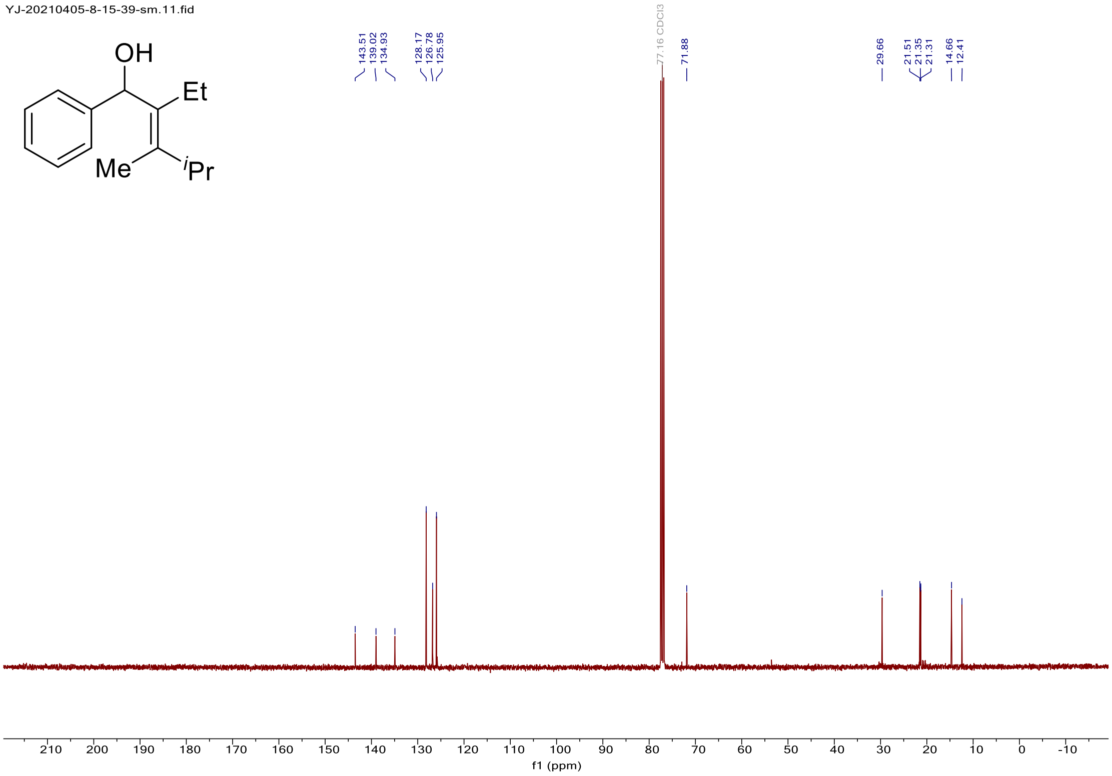

YJ-20210405-8-15-39-sm.11.fid
OH
Et
Me
iPr
143.51
139.02
134.93
128.17
126.78
125.95
77.16 CDCl3
71.88
29.66
21.51
21.35
21.31
14.66
12.41
f1 (ppm)

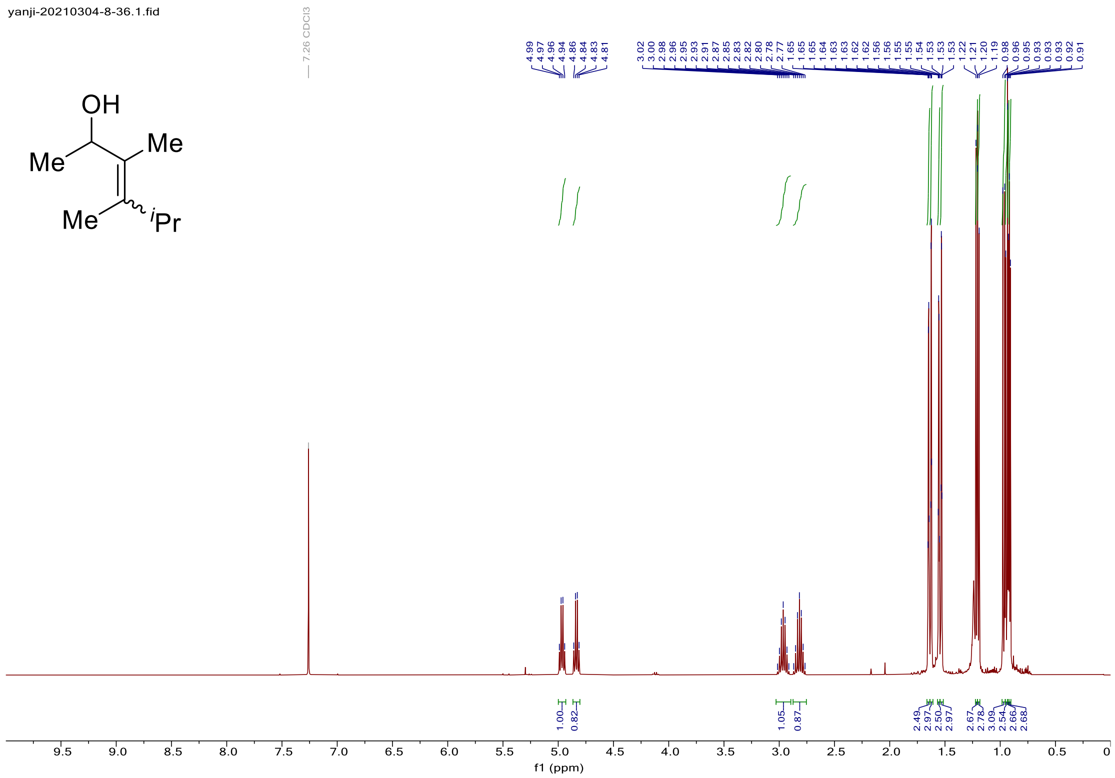
yanji-20210304-8-36.1.fid
OH
Me
Me
Me
iPr
7.26 CDCl3
4.99 4.97 4.96 4.94 4.86 4.84 4.83 4.81
3.02 3.00 2.98 2.96 2.95 2.93 2.91 2.87 2.85 2.83 2.82 2.80 2.78 2.77 1.65 1.65 1.65 1.64 1.63 1.63 1.62 1.62 1.56 1.56 1.55 1.55 1.54 1.53 1.53 1.53 1.22 1.21 1.20 1.19 0.98 0.96 0.95 0.93 0.93 0.93 0.92 0.91
1.00 0.82 1.05 0.87 2.49 2.97 2.50 2.97 2.67 2.78 3.09 2.54 2.66 2.68
9.5 9.0 8.5 8.0 7.5 7.0 6.5 6.0 5.5 5.0 4.5 4.0 3.5 3.0 2.5 2.0 1.5 1.0 0.5 0
f1 (ppm)

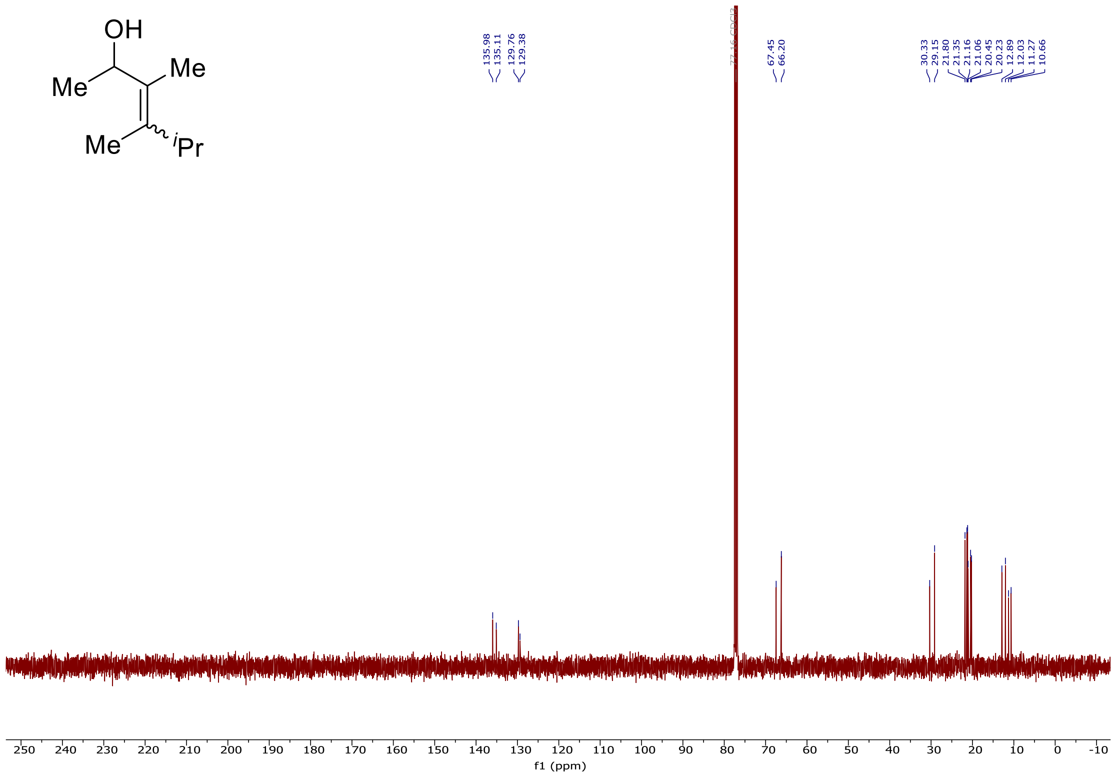
OH
Me
Me
Me
iPr
135.98 135.11 129.76 129.38
77.16 CDCl3
67.45 66.20
30.33 29.15 21.80 21.35 21.16 21.06 20.45 20.23 12.89 12.03 11.27 10.66
250 240 230 220 210 200 190 180 170 160 150 140 130 120 110 100 90 80 70 60 50 40 30 20 10 0 -10
f1 (ppm)

yanji-20210401-8-15-32-sm.1.fid

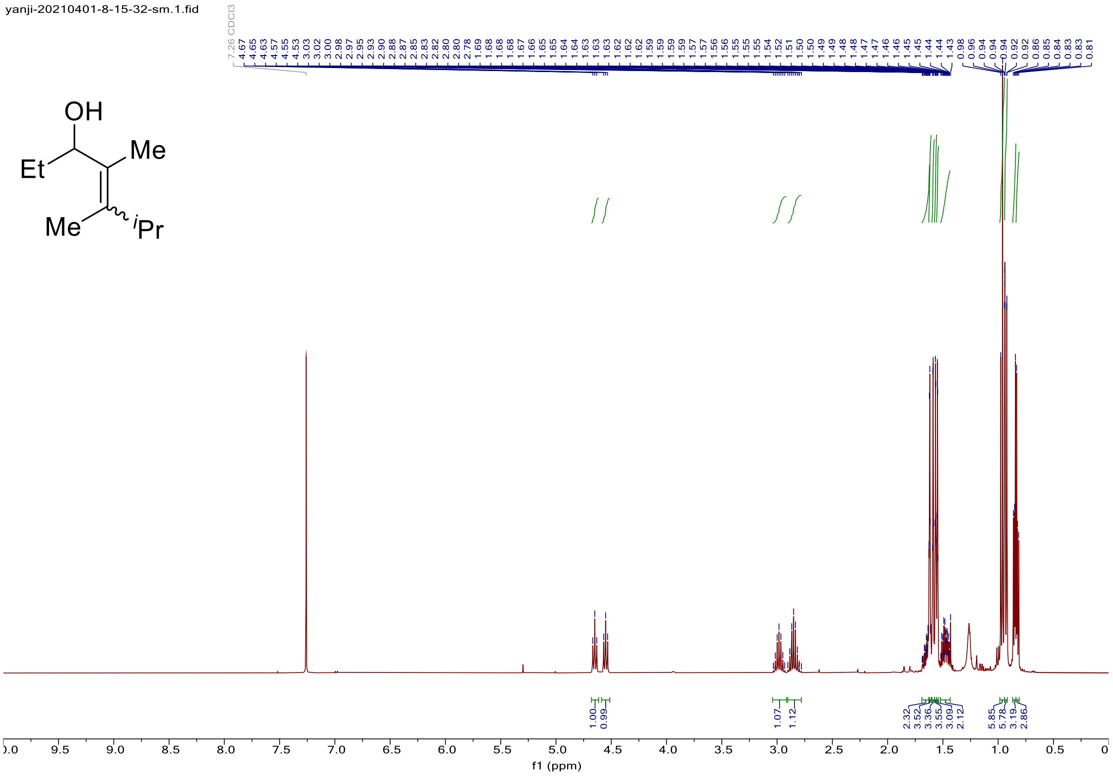

yanji-20210401-8-15-32-sm.2.fid

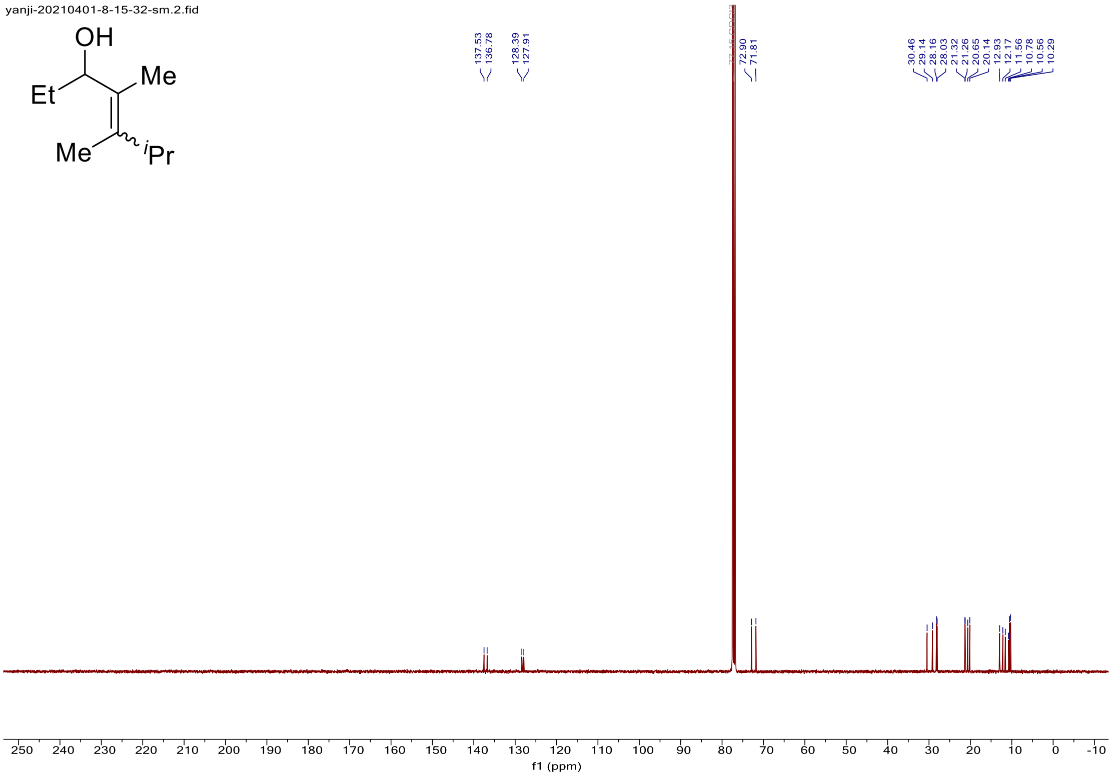

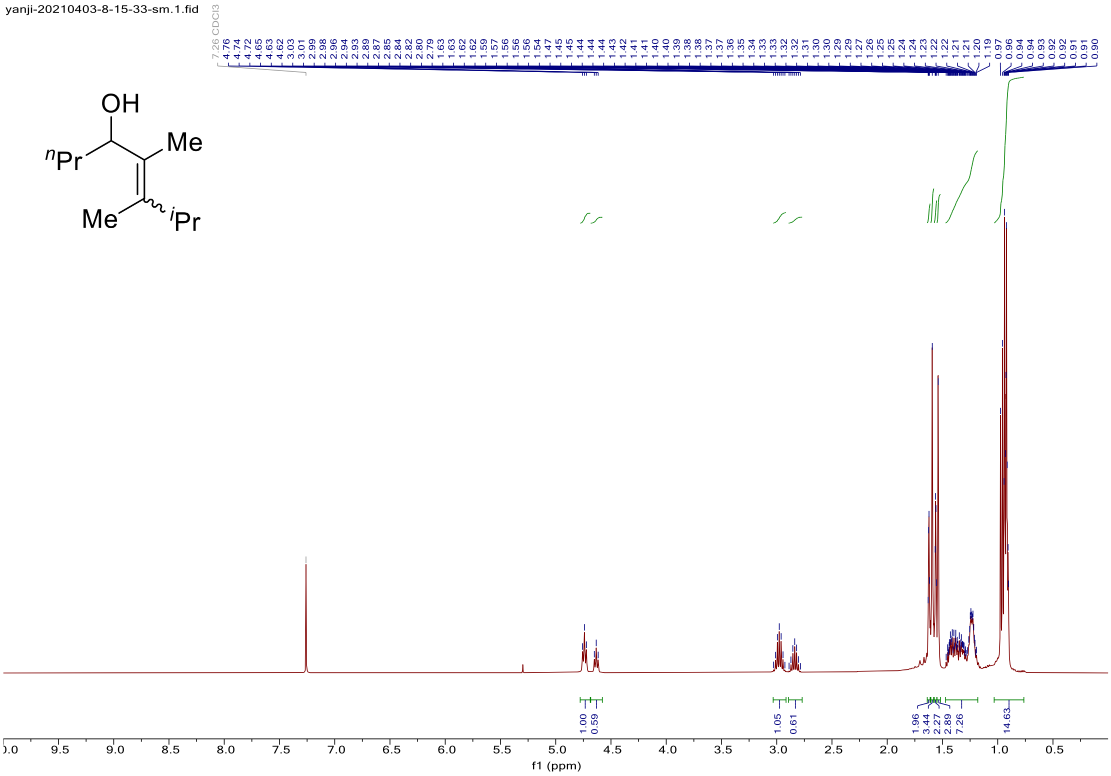
yanji-20210403-8-15-33-sm.1.fid
OH
ⁿPr
Me
Me
ⁱPr
7.26 CDCl3
4.76 4.74 4.72 4.65 4.63 4.62 3.03 3.01 2.99 2.98 2.96 2.94 2.93 2.89 2.87 2.85 2.84 2.82 2.80 2.79 1.63 1.63 1.62 1.59 1.57 1.56 1.56 1.56 1.54 1.47 1.45 1.45 1.44 1.44 1.44 1.43 1.42 1.41 1.41 1.40 1.40 1.39 1.38 1.38 1.37 1.37 1.36 1.35 1.34 1.33 1.33 1.32 1.32 1.31 1.30 1.29 1.29 1.27 1.26 1.25 1.25 1.24 1.24 1.23 1.22 1.22 1.21 1.21 1.20 1.19 0.97 0.96 0.94 0.94 0.93 0.92 0.92 0.91 0.91 0.90
1.00 0.59 1.05 0.61 1.96 3.44 2.27 2.89 7.26 14.63
f1 (ppm)

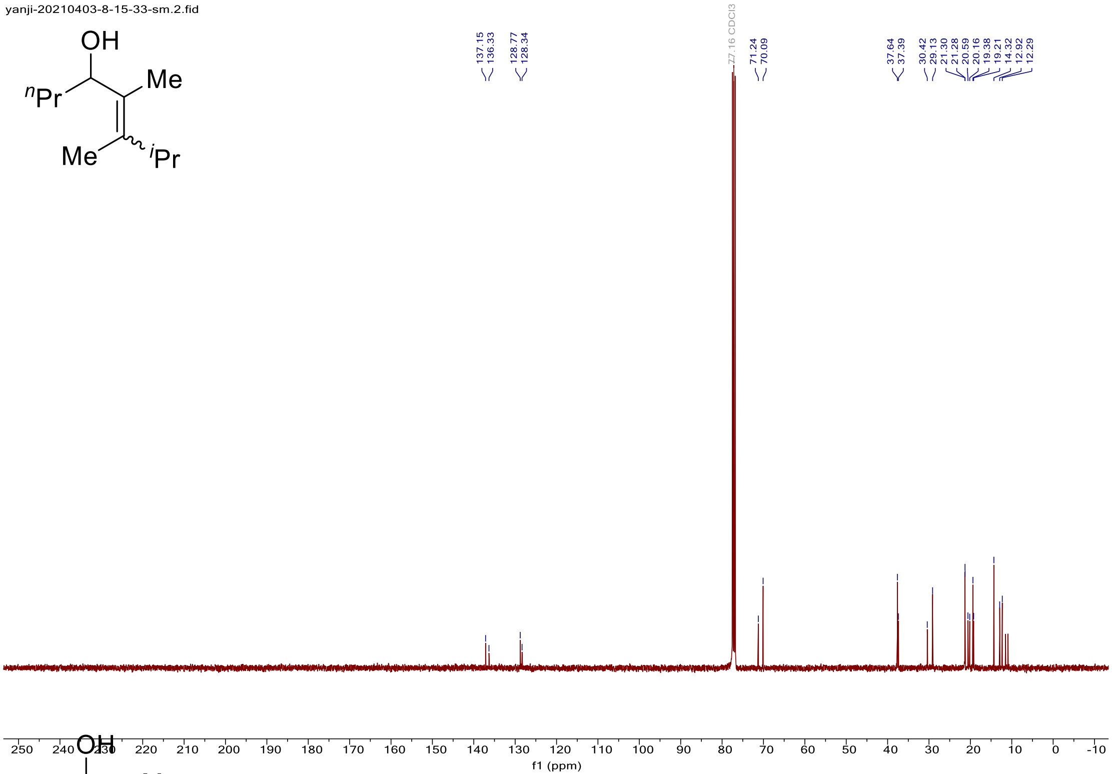
yanji-20210403-8-15-33-sm.2.fid
OH
ⁿPr
Me
Me
ⁱPr
137.15 136.33 128.77 128.34
77.16 CDCl3
71.24 70.09
37.64 37.39 30.42 29.13 21.30 21.28 20.59 20.16 19.38 19.21 14.32 12.92 12.29
f1 (ppm)

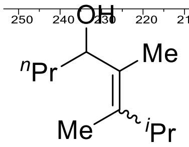
OH
ⁿPr
Me
Me
ⁱPr

YJ-20210404-8-15-34-sm.10.fid

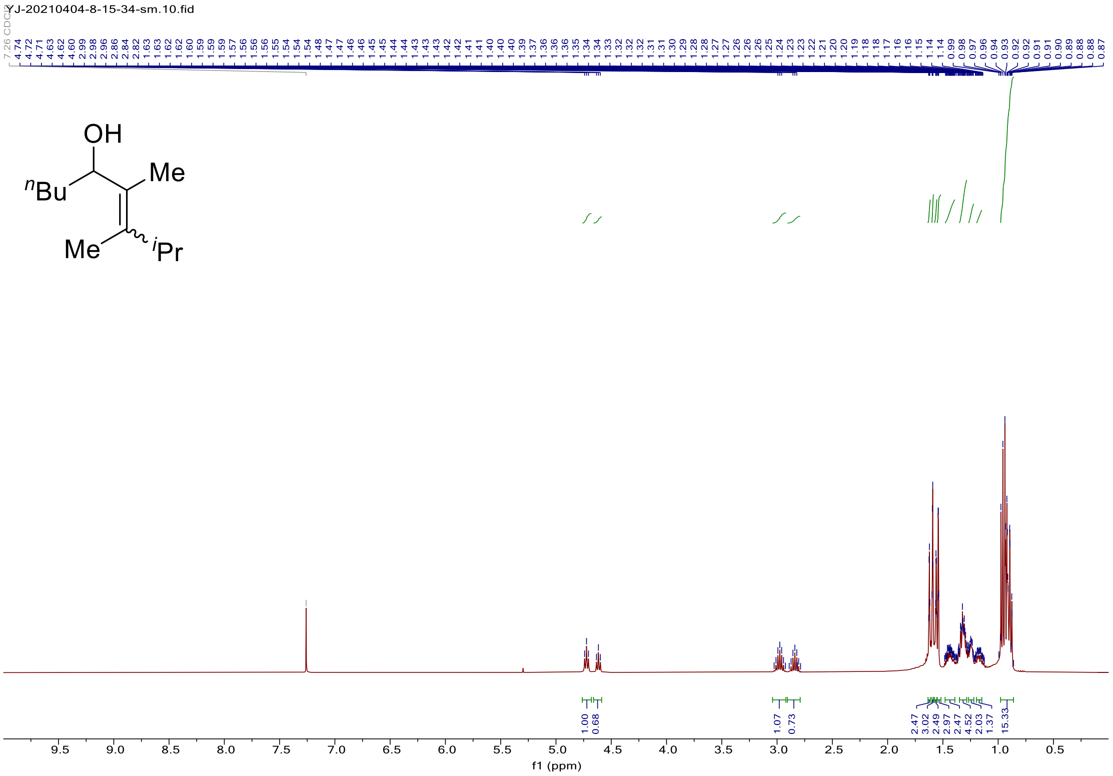

YJ-20210404-8-15-34-sm.11.fid

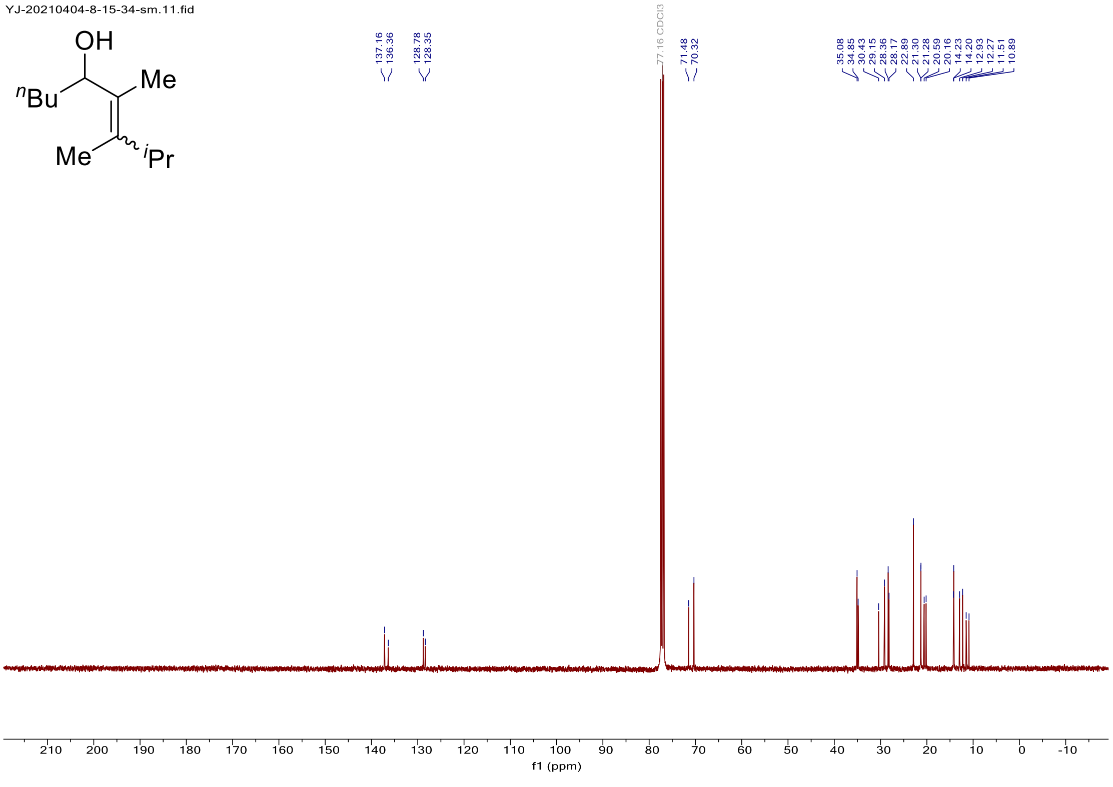

yanji-20211124-8-15-41-SM.1.fid

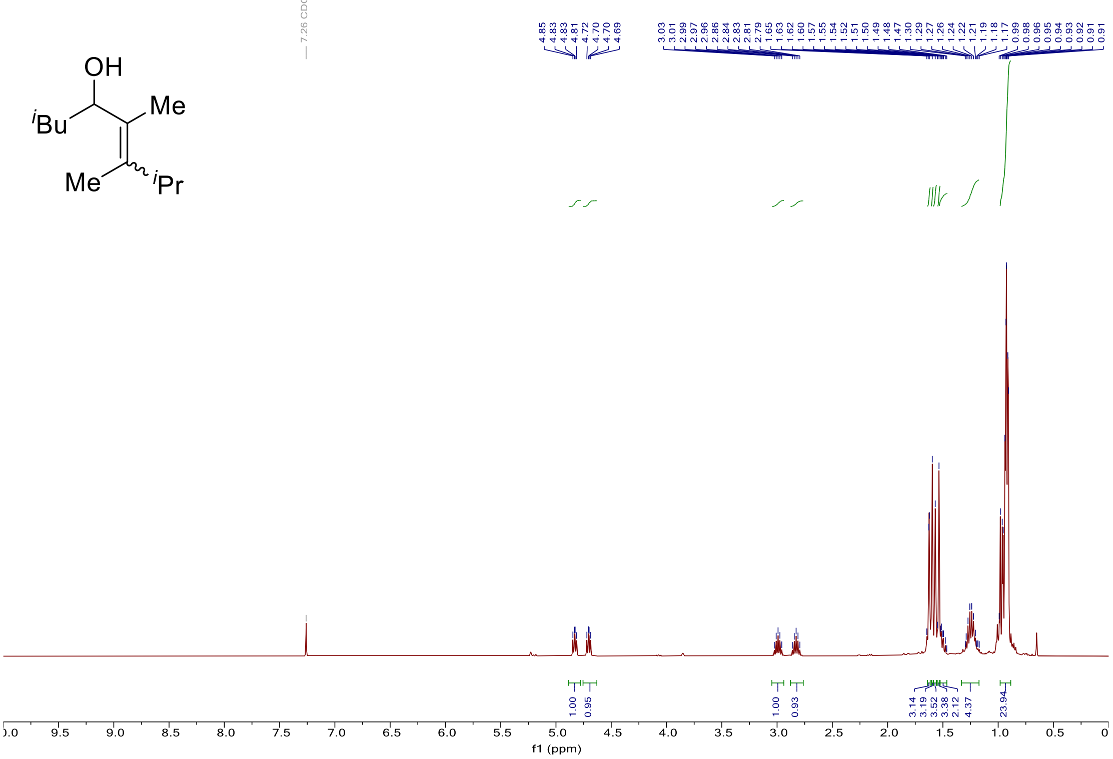


yanji-20211124-8-15-41-SM.2.fid

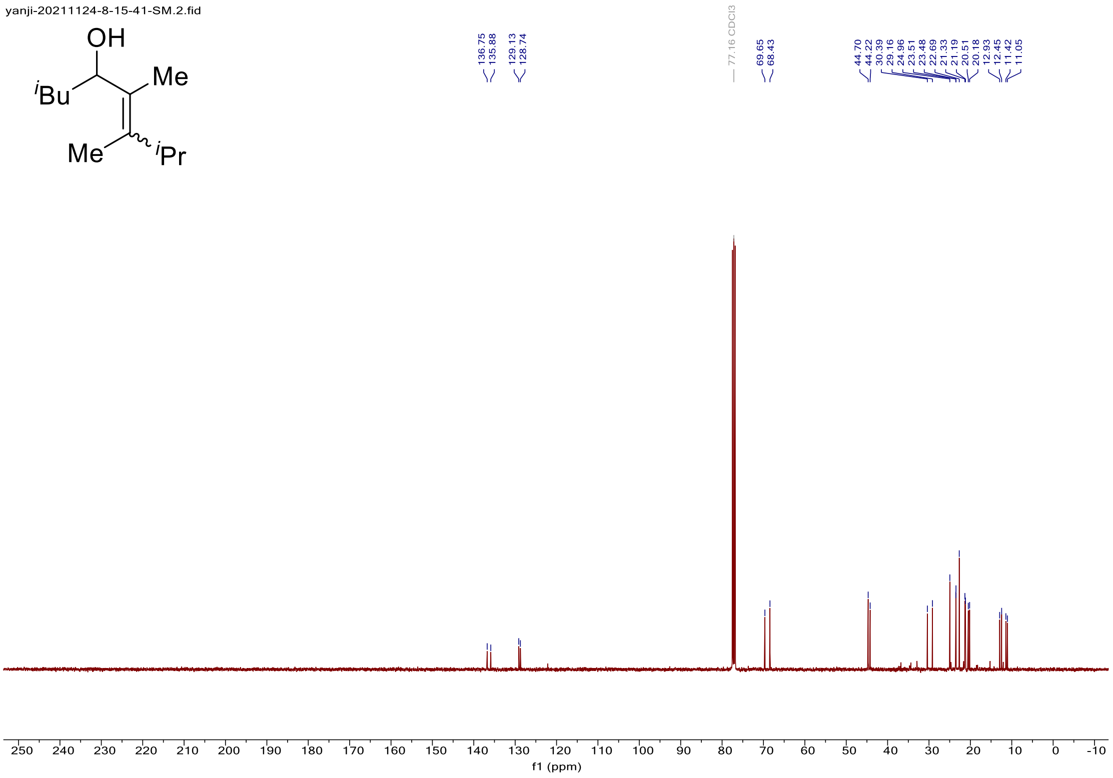

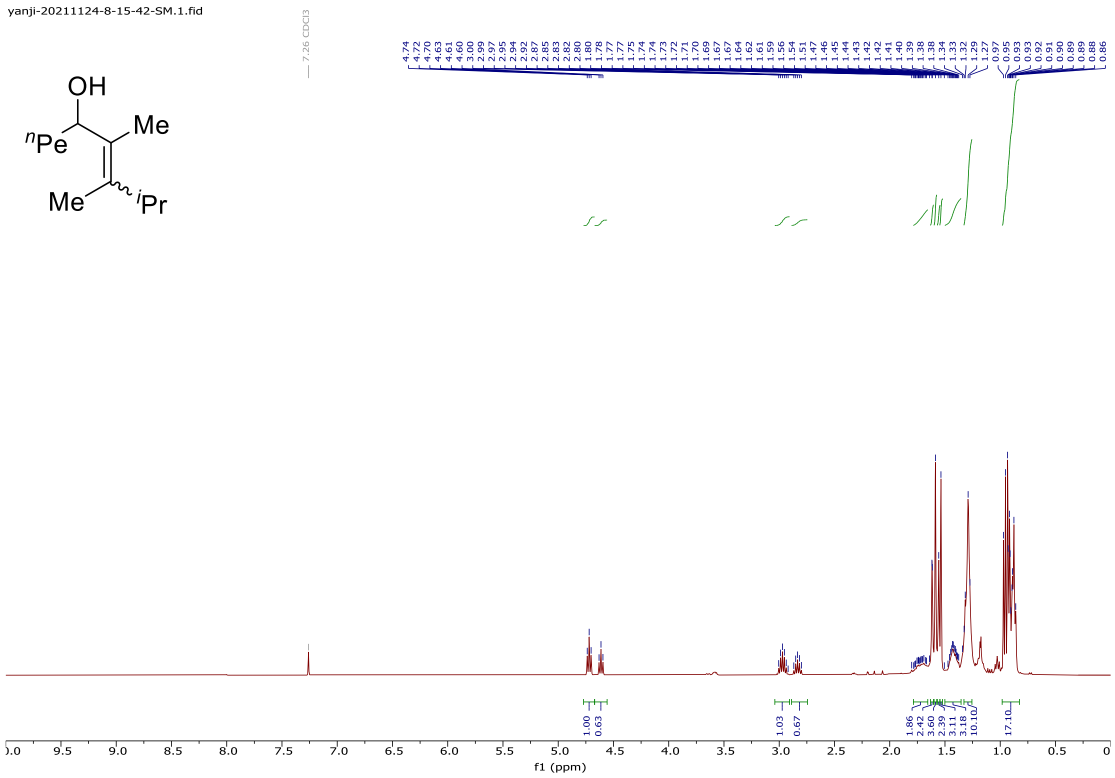
yanji-20211124-8-15-42-SM.1.fid
OH
nPe
Me
Me
iPr
7.26 CDCl3
4.74 4.72 4.70 4.63 4.61 4.60 3.00 2.99 2.97 2.95 2.94 2.92 2.87 2.85 2.83 2.82 2.80 1.80 1.78 1.77 1.77 1.75 1.74 1.74 1.73 1.72 1.71 1.70 1.69 1.67 1.67 1.64 1.62 1.61 1.59 1.56 1.54 1.51 1.47 1.46 1.45 1.44 1.43 1.42 1.42 1.41 1.40 1.39 1.38 1.38 1.34 1.33 1.32 1.29 1.27 0.97 0.95 0.93 0.93 0.92 0.91 0.90 0.89 0.88 0.86
1.00 0.63 1.03 0.67 1.86 2.42 3.60 2.39 3.11 3.18 10.10 17.10
f1 (ppm)

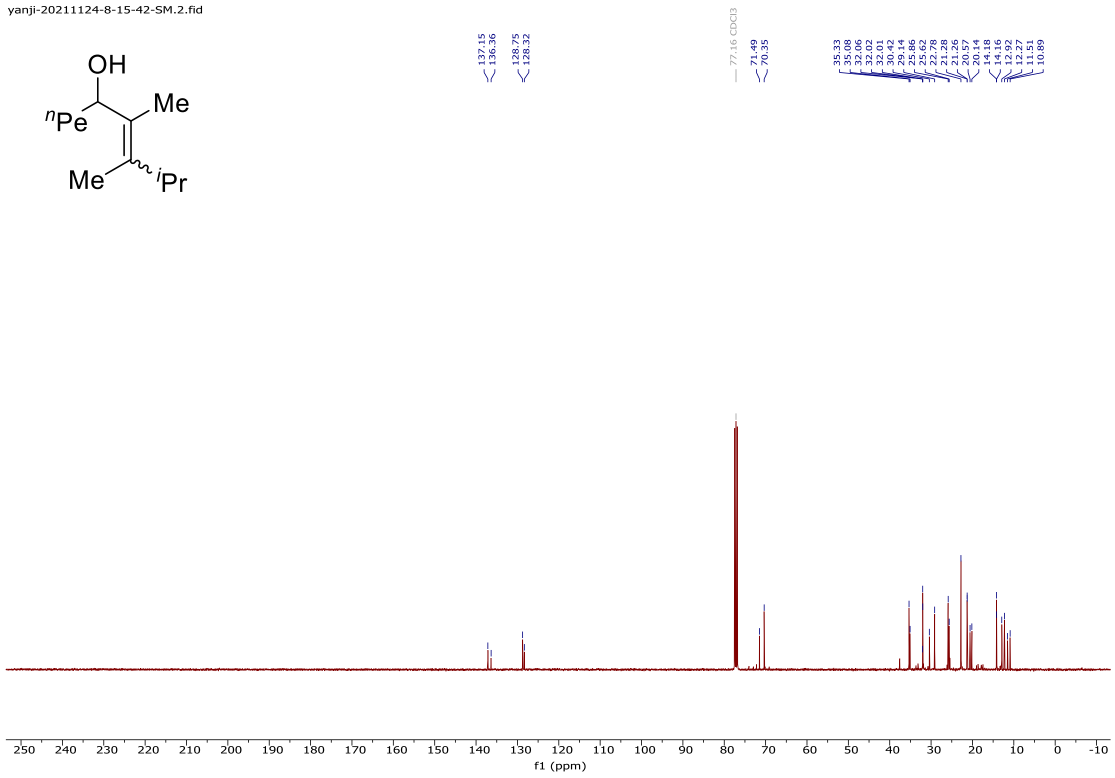
yanji-20211124-8-15-42-SM.2.fid
OH
nPe
Me
Me
iPr
137.15 136.36 128.75 128.32
77.16 CDCl3
71.49 70.35
35.33 35.08 32.06 32.02 32.01 30.42 29.14 25.86 25.62 22.78 21.28 21.26 20.57 20.14 14.18 14.16 12.92 12.27 11.51 10.89
f1 (ppm)

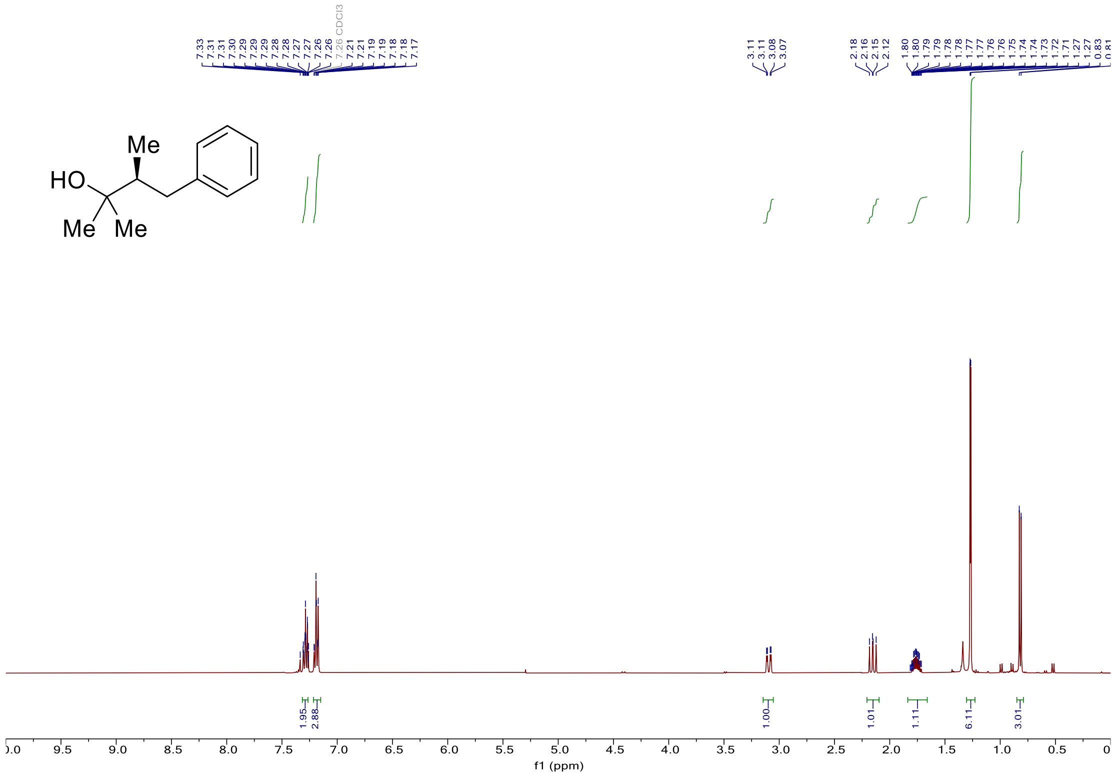
7.33 7.31 7.31 7.30 7.29 7.29 7.28 7.28 7.27 7.27 7.26 7.26 CDCl3 7.21 7.21 7.19 7.19 7.18 7.18 7.17
3.11 3.11 3.08 3.07
2.18 2.16 2.15 2.12
1.80 1.80 1.79 1.79 1.78 1.78 1.77 1.77 1.76 1.76 1.75 1.74 1.74 1.73 1.72 1.71 1.27 1.27 0.83
HO
Me
Me Me
1.95
2.88
1.00
1.01
1.11
6.11
3.01
f1 (ppm)

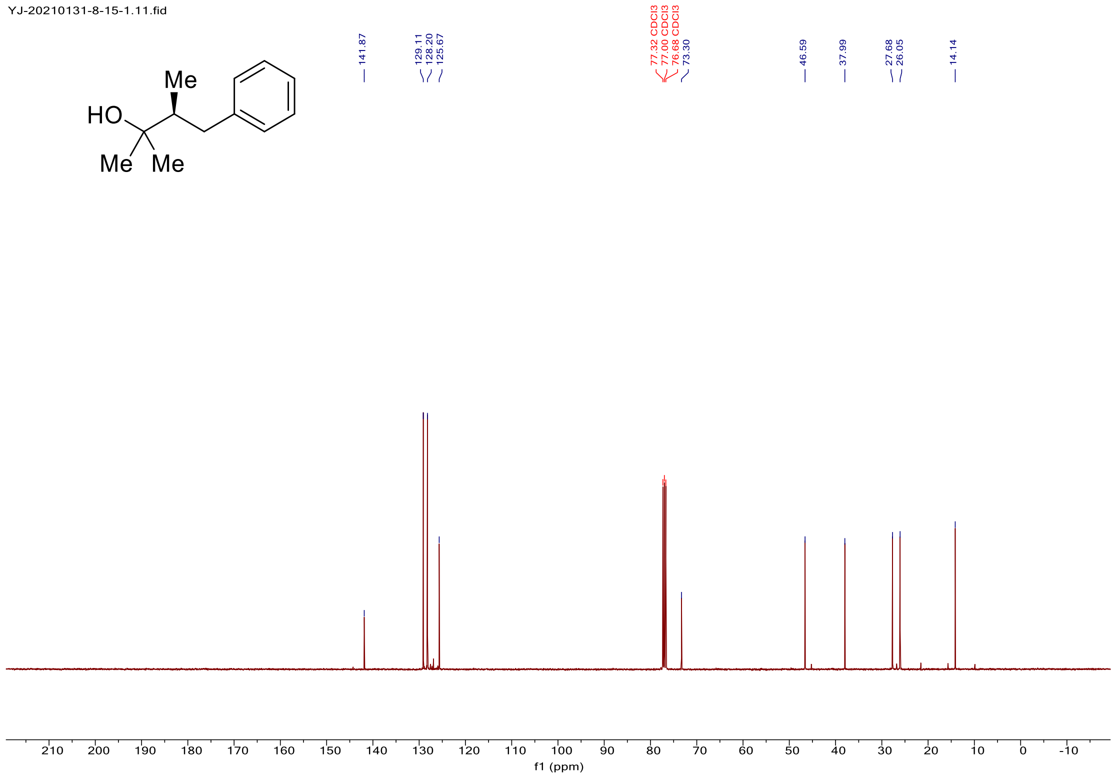
YJ-20210131-8-15-1.11.fid
141.87
129.11 128.20 125.67
77.32 CDCl3 77.00 CDCl3 76.68 CDCl3 73.30
46.59
37.99
27.68 26.05
14.14
HO
Me
Me Me
f1 (ppm)

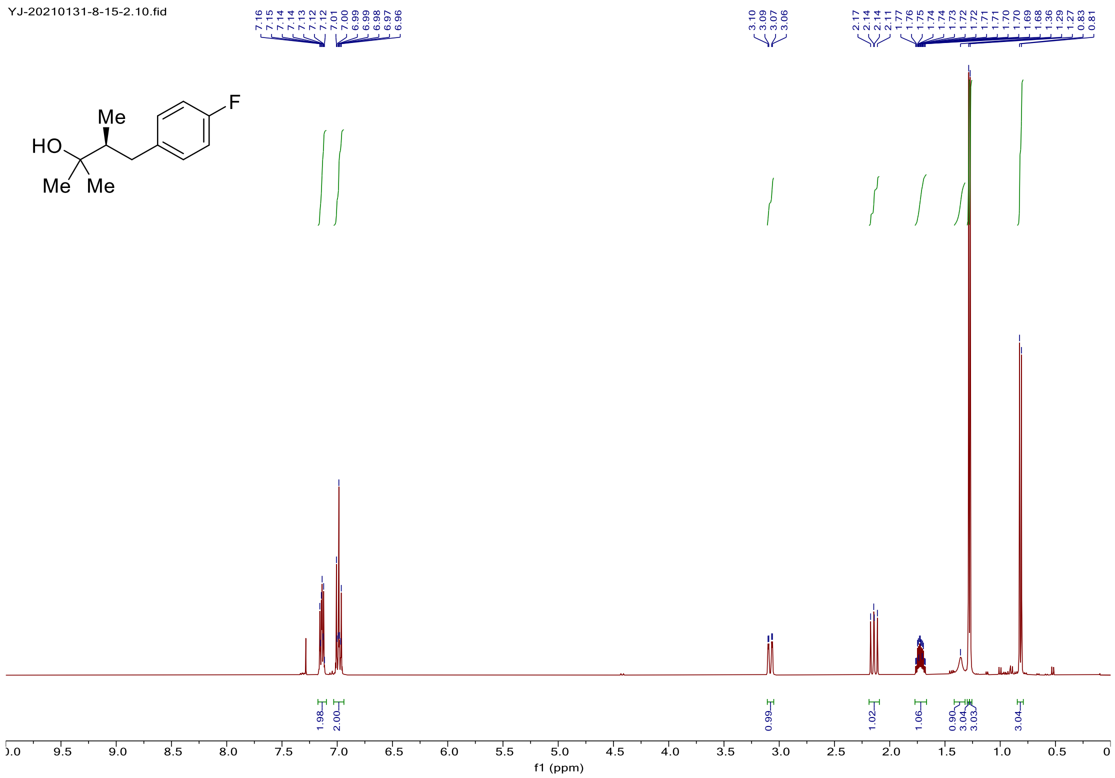
YJ-20210131-8-15-2.10.fid
7.16 7.15 7.14 7.14 7.13 7.12 7.12 7.01 7.00 6.99 6.99 6.98 6.97 6.96
3.10 3.09 3.07 3.06
2.17 2.14 2.14 2.11 1.77 1.76 1.75 1.74 1.74 1.73 1.72 1.72 1.71 1.71 1.70 1.70 1.69 1.68 1.36 1.29 1.27 0.83 0.81
HO
Me
Me Me
F
1.98 2.00 0.99 1.02 1.06 0.90 3.04 3.03 3.04
f1 (ppm)

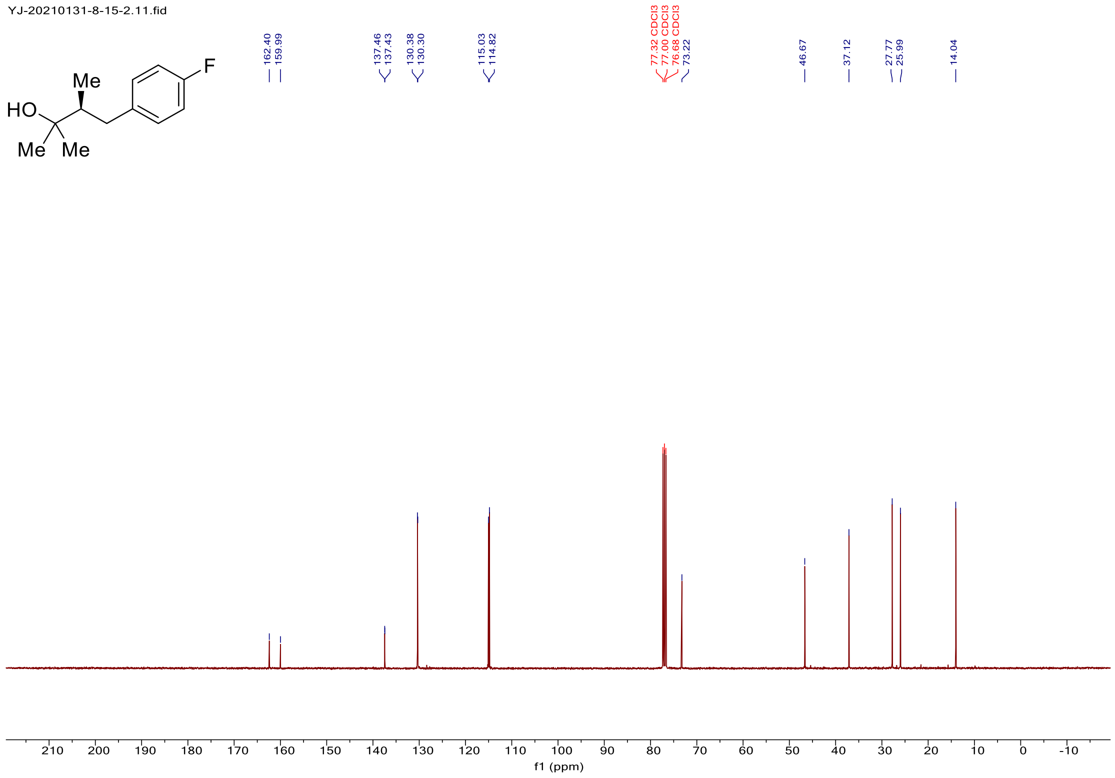
YJ-20210131-8-15-2.11.fid
162.40 159.99
137.46 137.43 130.38 130.30
115.03 114.82
77.32 CDCl3 77.00 CDCl3 76.68 CDCl3 73.22
46.67
37.12
27.77 25.99
14.04
HO
Me
Me Me
F
f1 (ppm)

yanji-20220106-8-15-2-F.1.fid

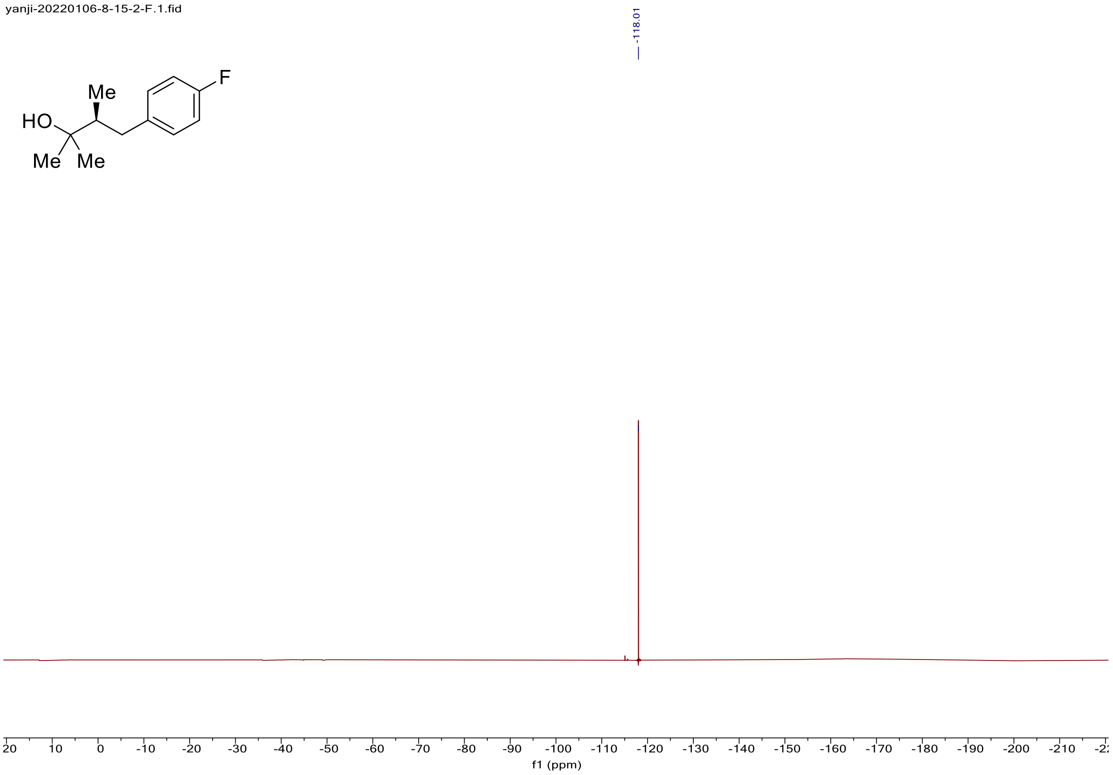

yanji-20210318-8-15-6.1.fid

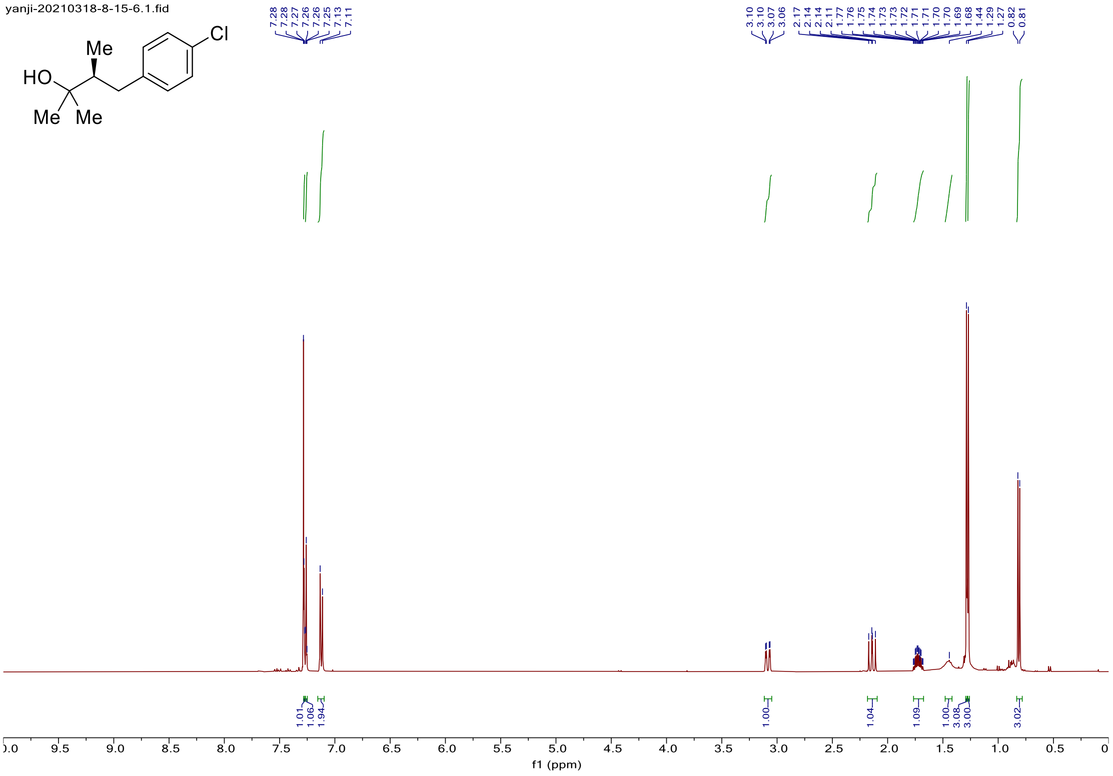

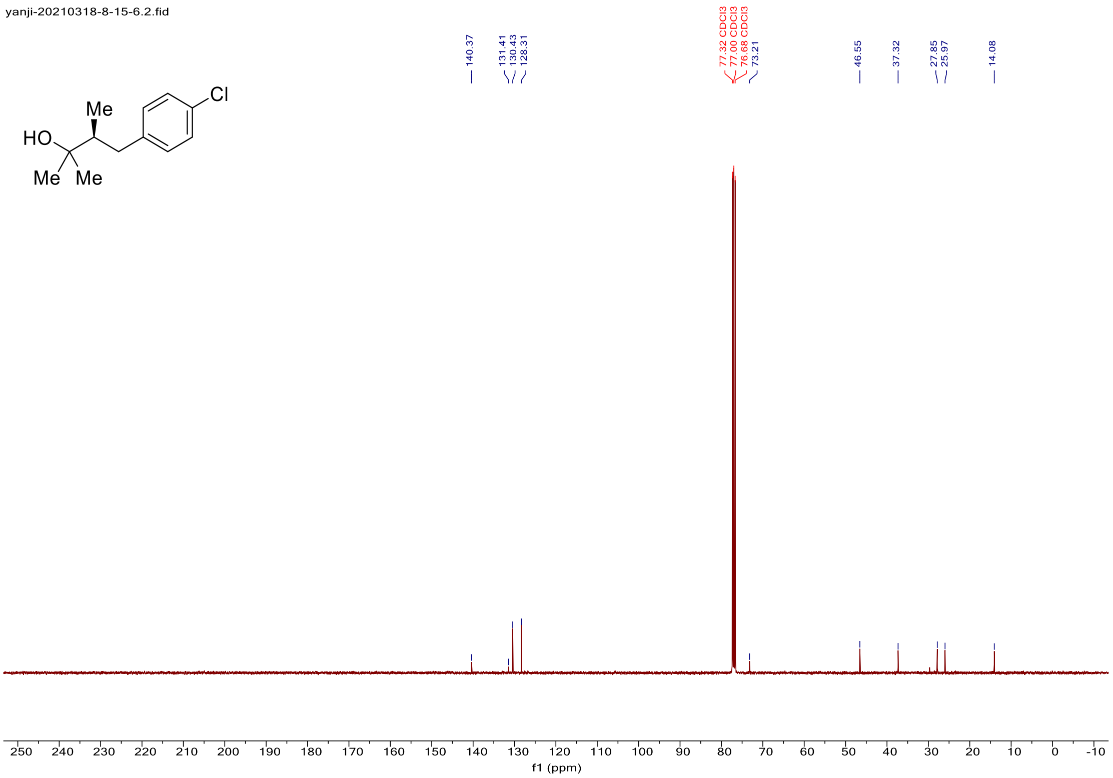
yanji-20210318-8-15-6.2.fid
140.37
131.41
130.43
128.31
77.32 CDCl3
77.00 CDCl3
76.68 CDCl3
73.21
46.55
37.32
27.85
25.97
14.08
HO
Me
Me
Me
Cl
f1 (ppm)

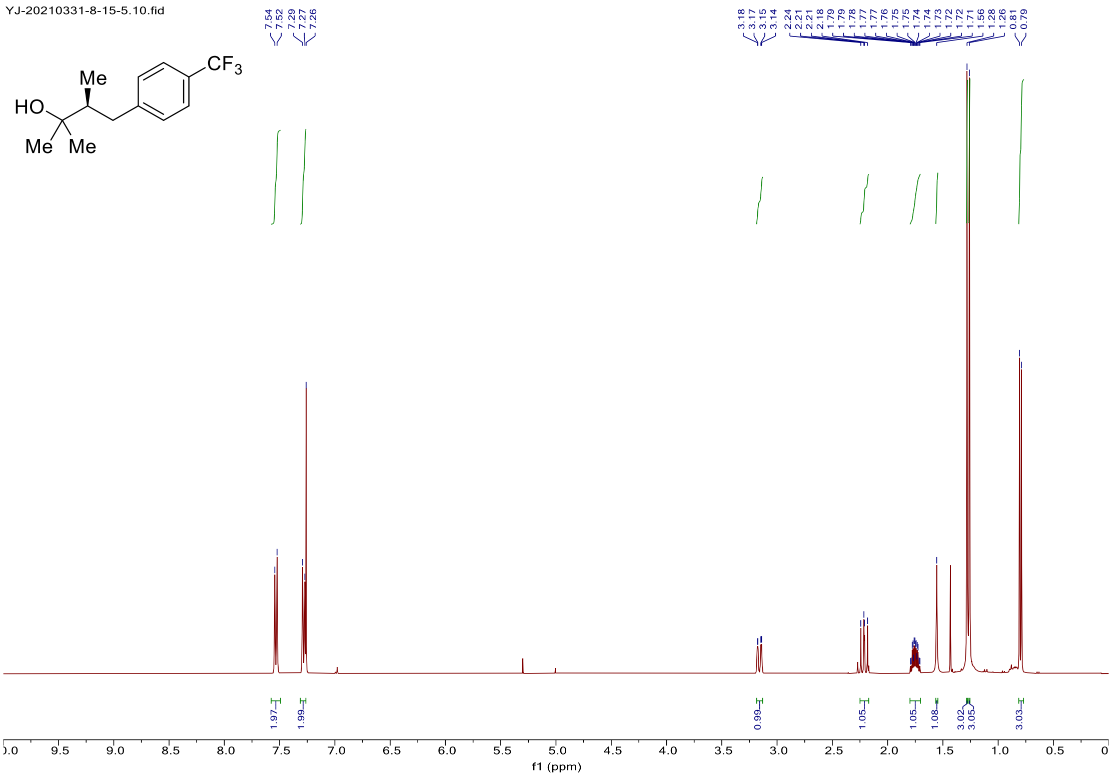
YJ-20210331-8-15-5.10.fid
7.54
7.52
7.29
7.27
7.26
3.18
3.17
3.15
3.14
2.24
2.21
2.21
2.18
1.79
1.78
1.77
1.77
1.76
1.75
1.75
1.74
1.73
1.72
1.72
1.71
1.56
1.28
1.26
0.81
0.79
HO
Me
Me
Me
CF3
1.97
1.99
0.99
1.05
1.05
1.08
3.02
3.05
3.03
f1 (ppm)

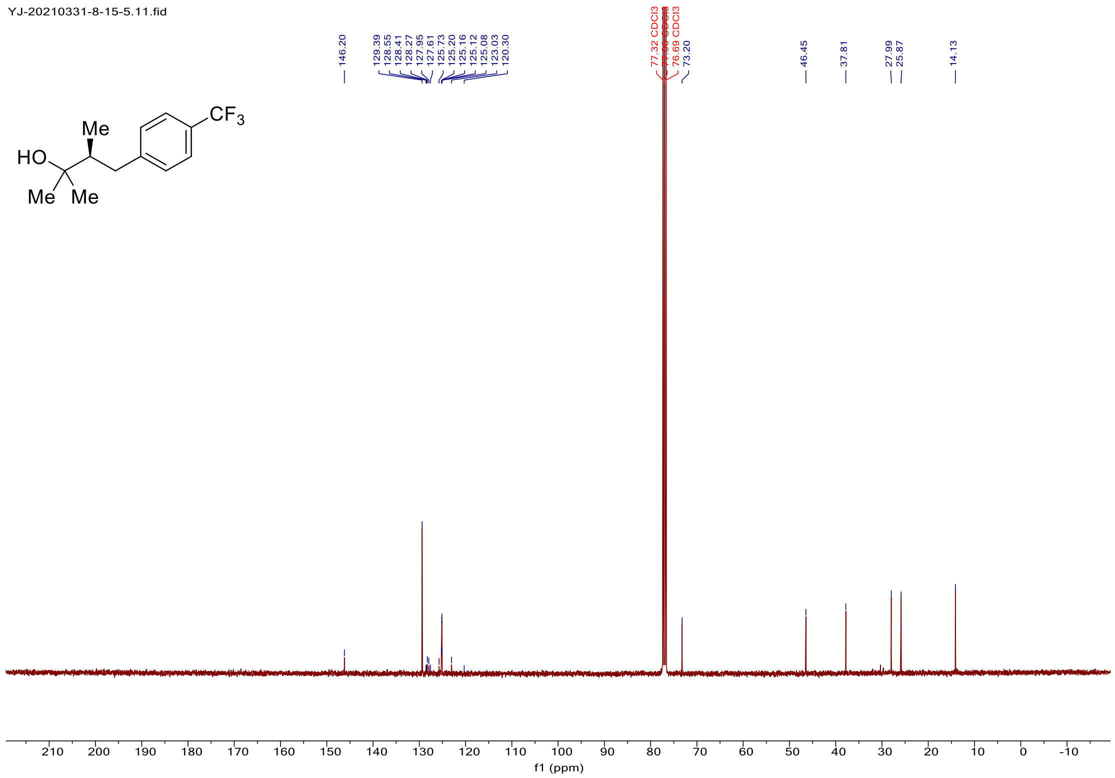
YJ-20210331-8-15-5.11.fid
146.20
129.39
128.55
128.41
128.27
127.95
127.61
125.73
125.20
125.16
125.12
125.08
123.03
120.30
77.32 CDCl3
76.69 CDCl3
73.20
46.45
37.81
27.99
25.87
14.13
CF3
Me
HO
Me Me
210 200 190 180 170 160 150 140 130 120 110 100 90 80 70 60 50 40 30 20 10 0 -10
f1 (ppm)

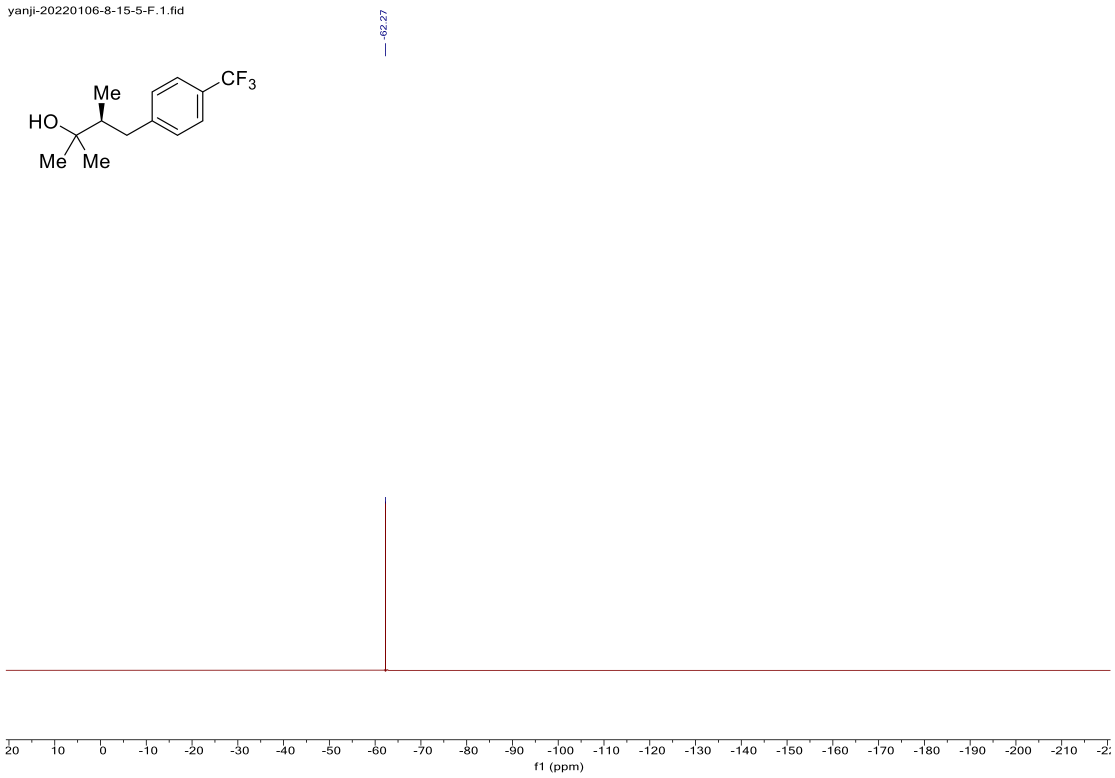
yanji-20220106-8-15-5-F.1.fid
-62.27
CF3
Me
HO
Me Me
20 10 0 -10 -20 -30 -40 -50 -60 -70 -80 -90 -100 -110 -120 -130 -140 -150 -160 -170 -180 -190 -200 -210
f1 (ppm)

YJ-20210319-8-15-3.10.fid

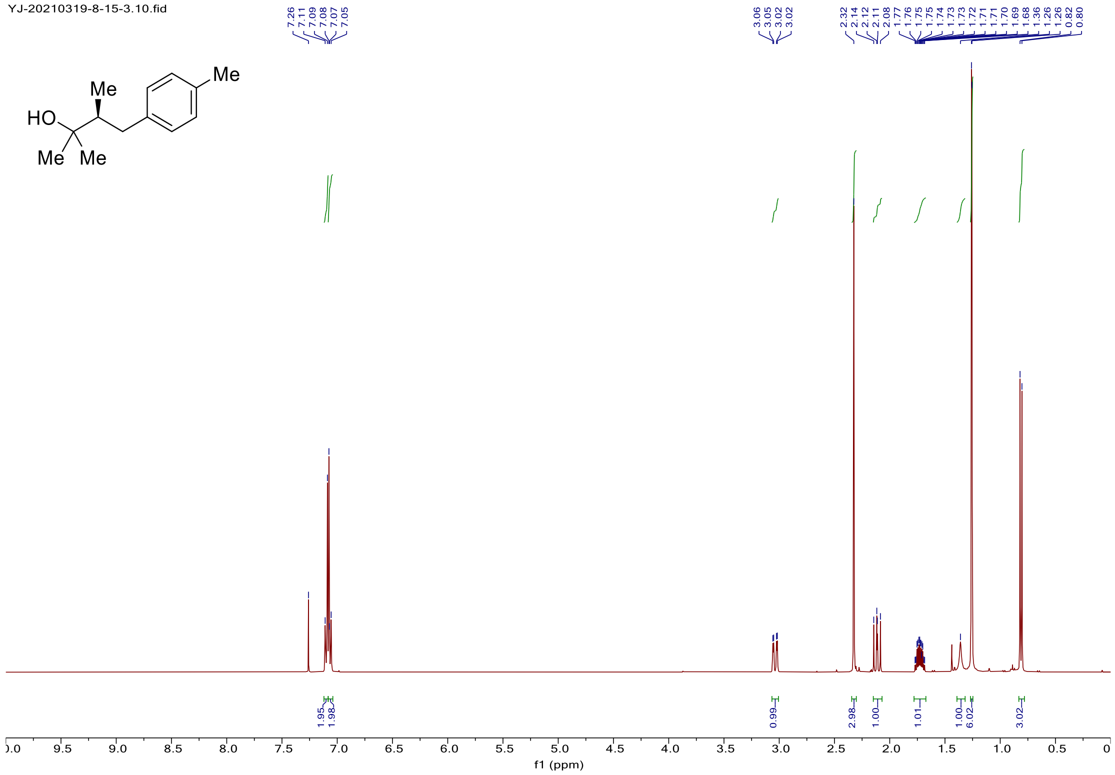


YJ-20210319-8-15-3.11.fid

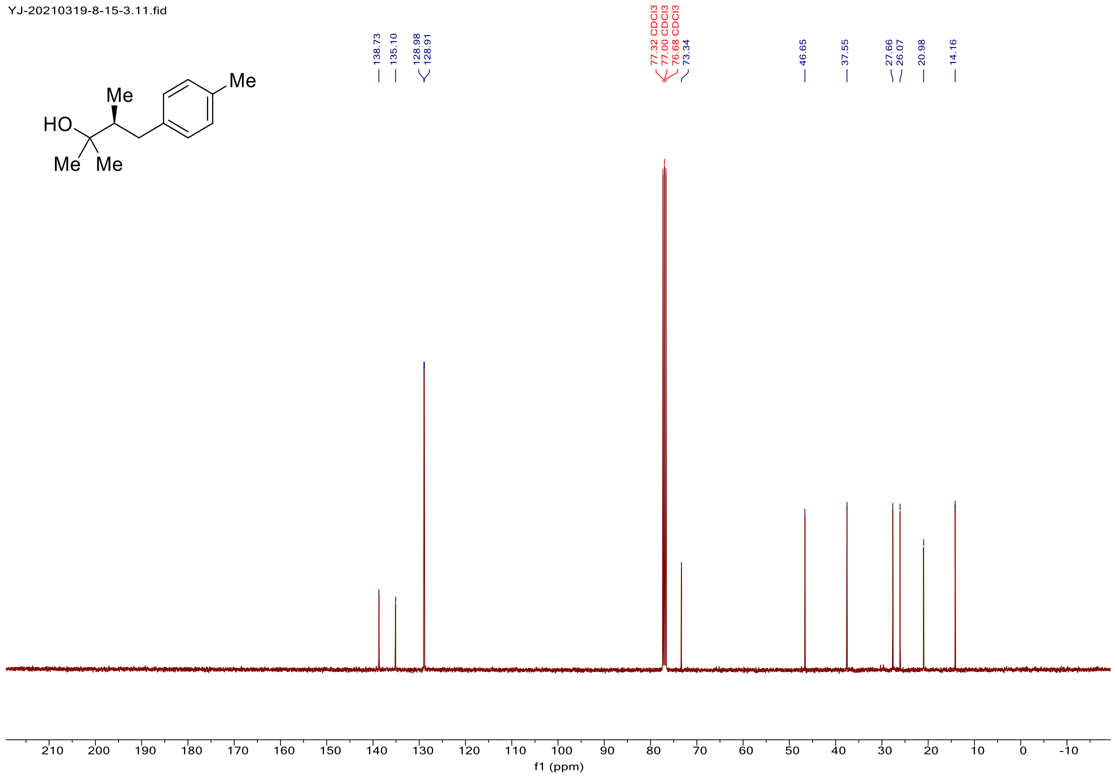

yanji-20210318-8-15-7.1.fid

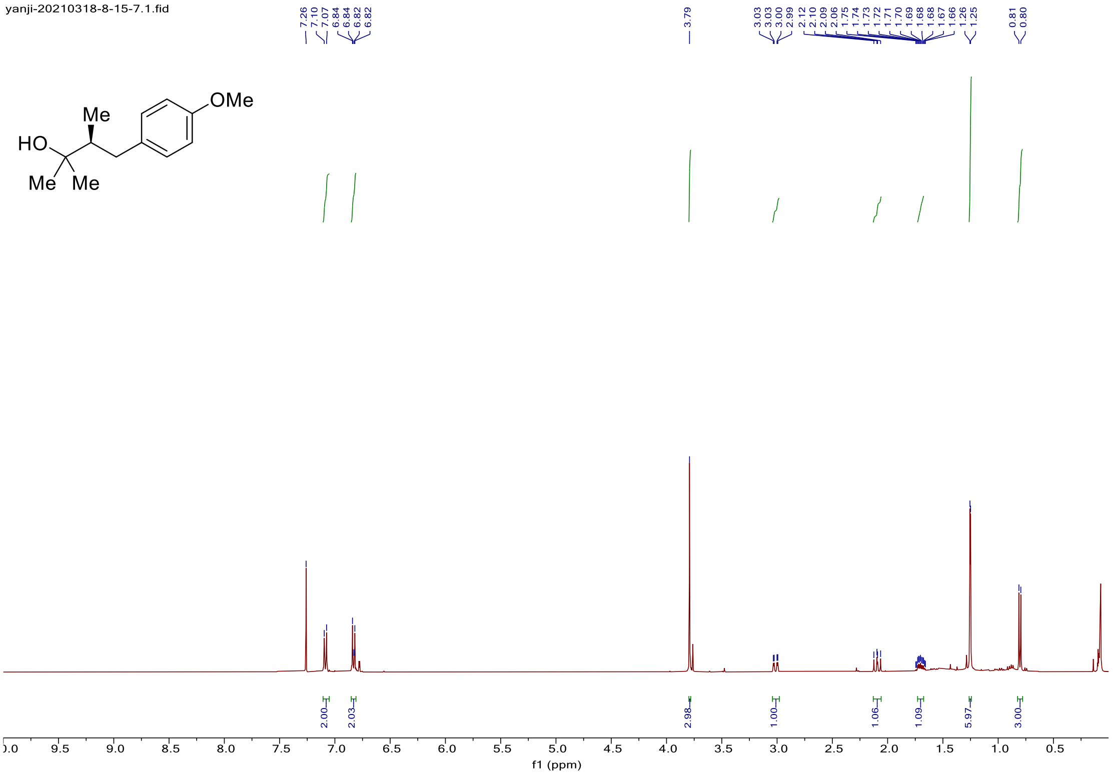

yanji-20210318-8-15-7.3.fid

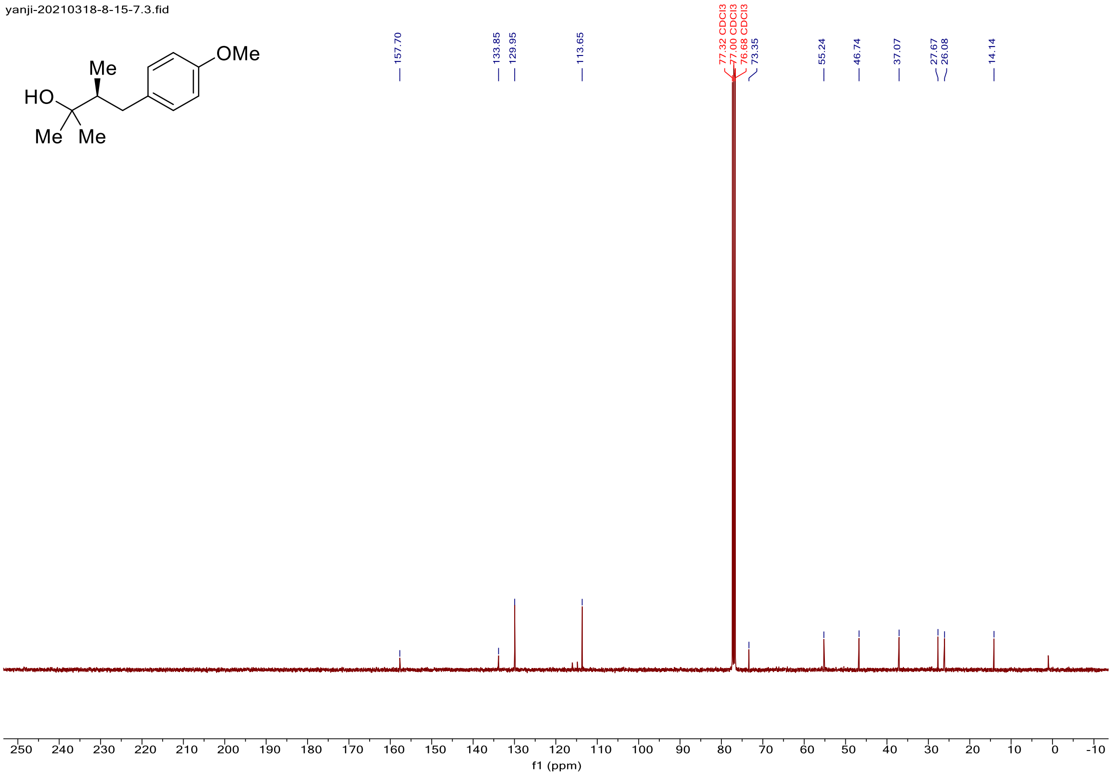

YJ-20210319-8-15-4.10.fid

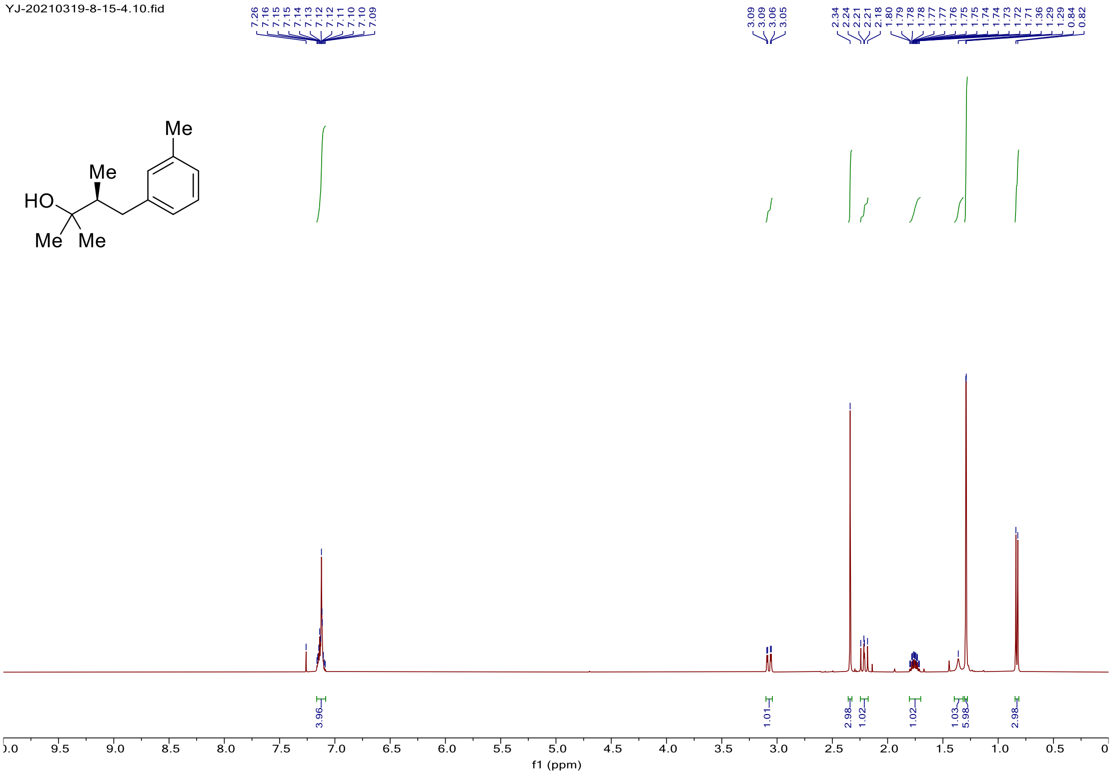

YJ-20210319-8-15-4.11.fid

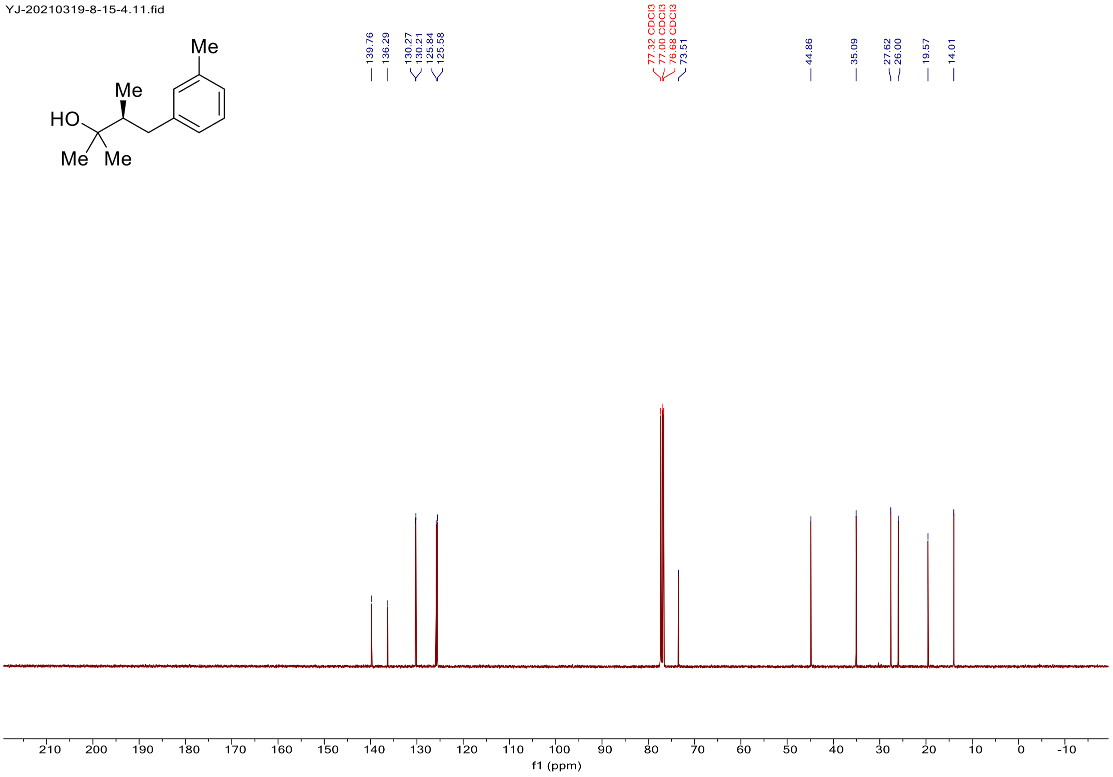

YJ-20210319-8-15-8.10.fid

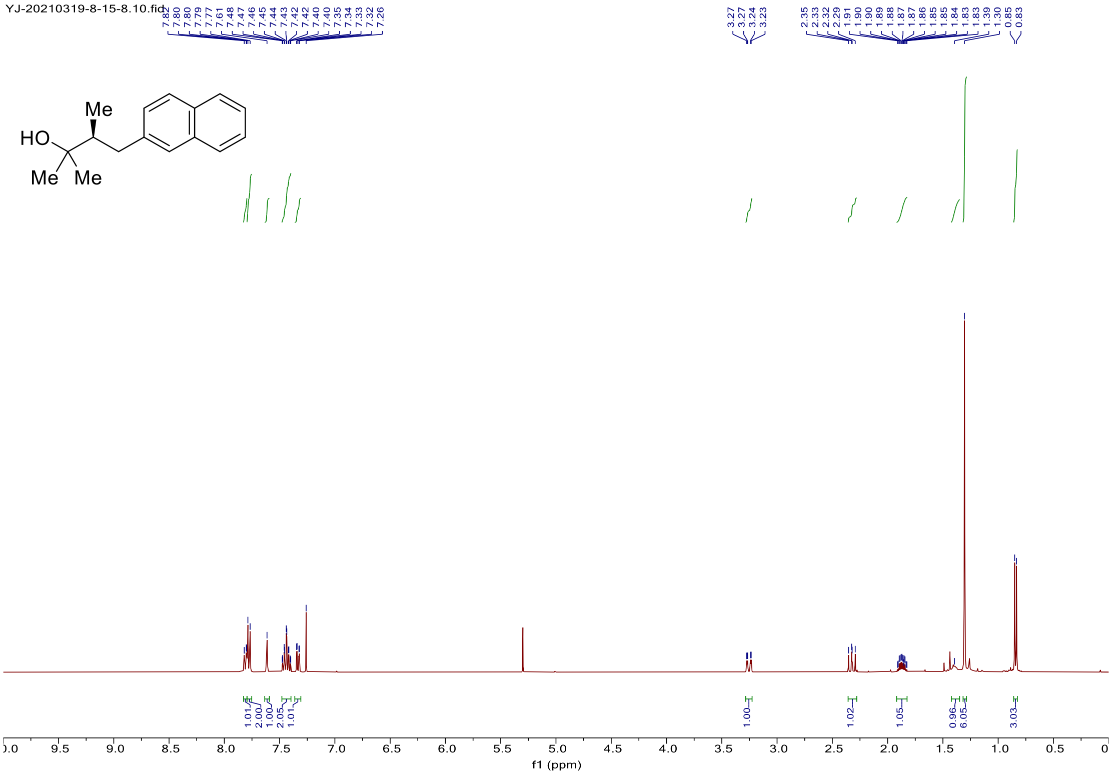

YJ-20210319-8-15-8.11.fid

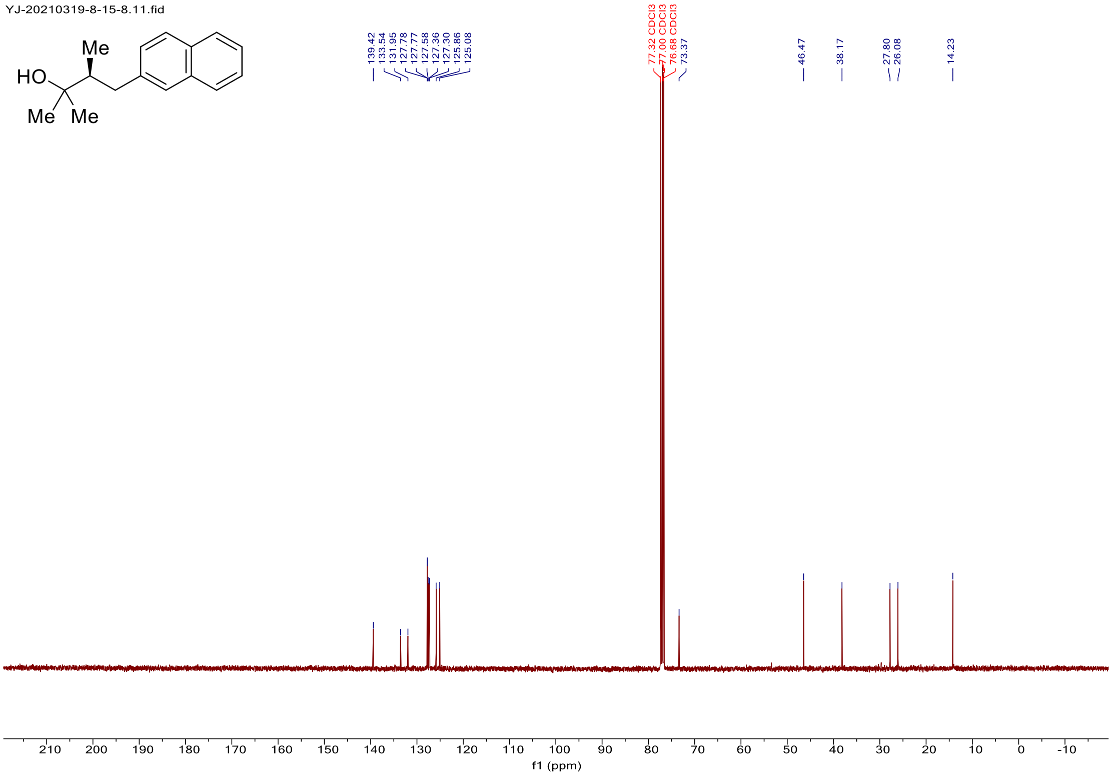

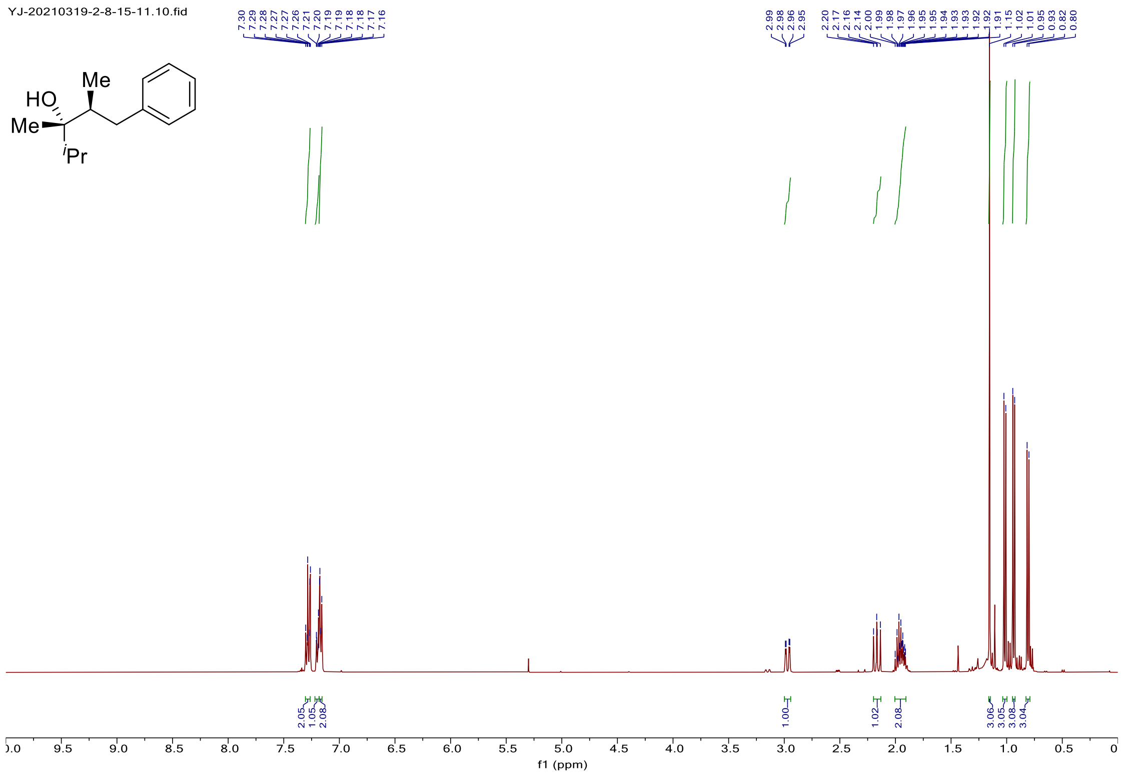
YJ-20210319-2-8-15-11.10.fid
7.30 7.29 7.28 7.27 7.27 7.26 7.21 7.20 7.19 7.19 7.19 7.18 7.17 7.16
2.99 2.98 2.96 2.95
2.20 2.17 2.16 2.14 2.00 1.99 1.98 1.97 1.96 1.95 1.95 1.94 1.93 1.93 1.92 1.92 1.91 1.15 1.02 1.01 0.95 0.93 0.82 0.80
HO
Me
Me
iPr
2.05 1.05 2.08 1.00 1.02 2.08 3.06 3.05 3.08 3.04
f1 (ppm)

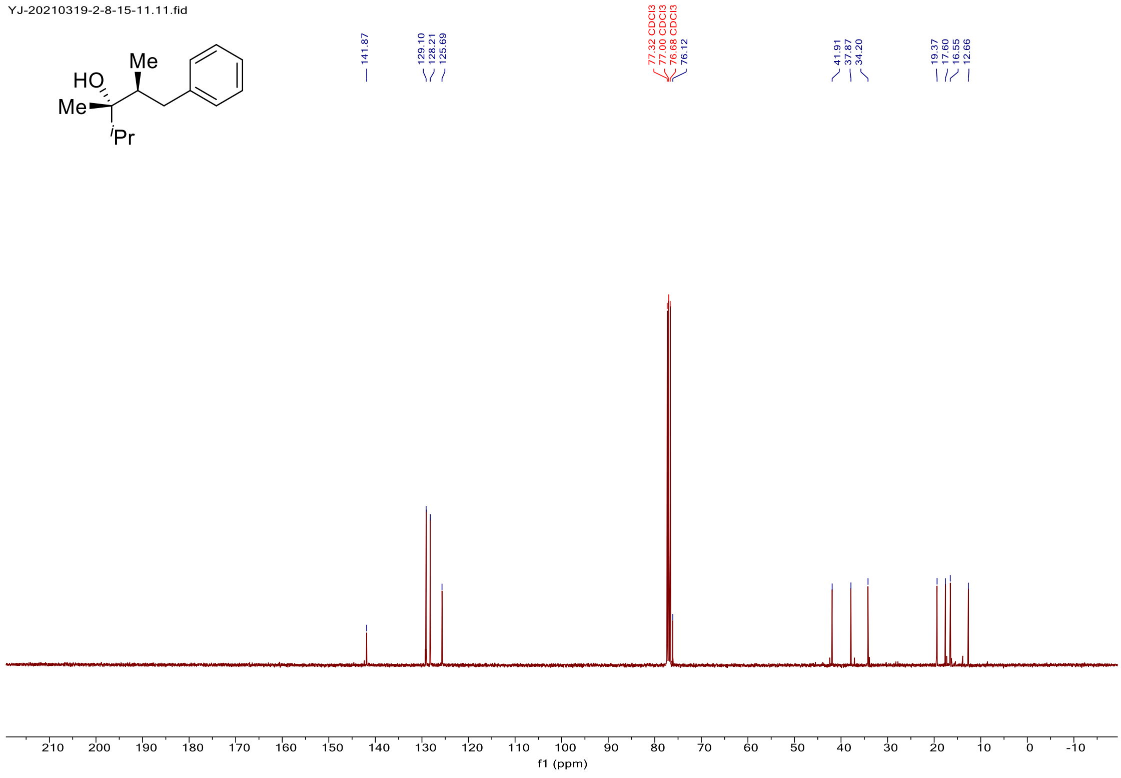
YJ-20210319-2-8-15-11.11.fid
141.87
129.10 128.21 125.69
77.32 CDCl3 77.00 CDCl3 76.68 CDCl3 76.12
41.91 37.87 34.20
19.37 17.60 16.55 12.66
HO
Me
Me
iPr
f1 (ppm)

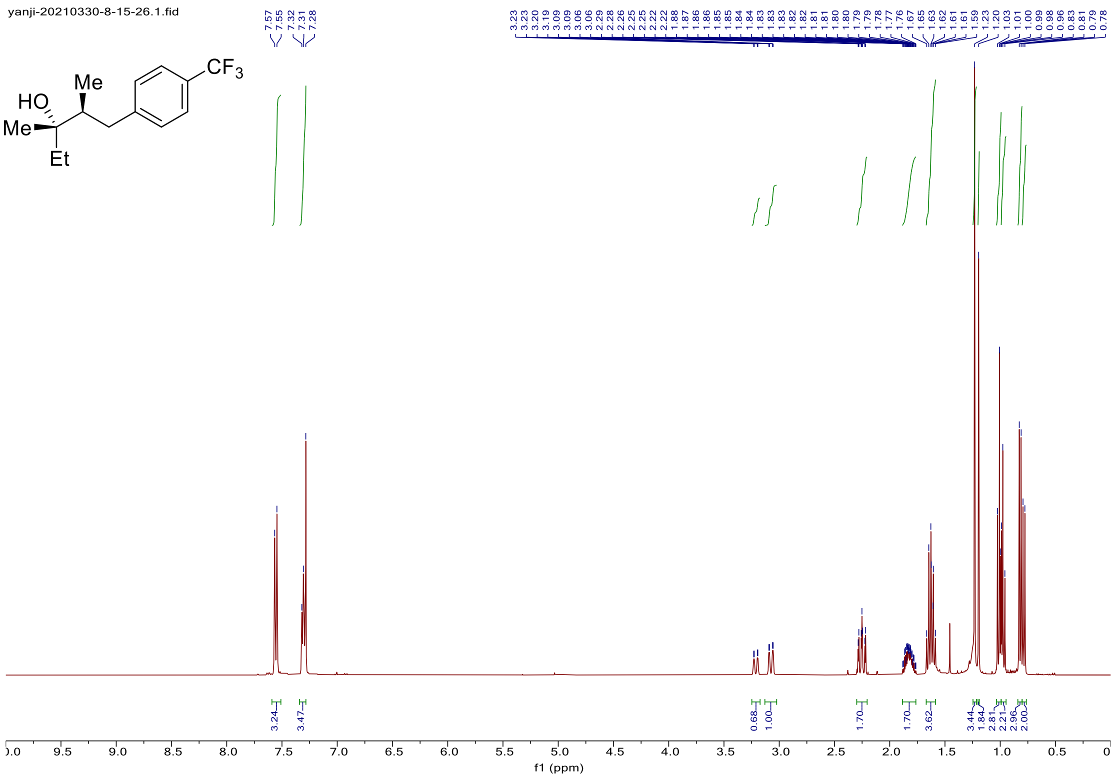
yanji-20210330-8-15-26.1.fid
HO
Me
Me
Et
CF3
f1 (ppm)

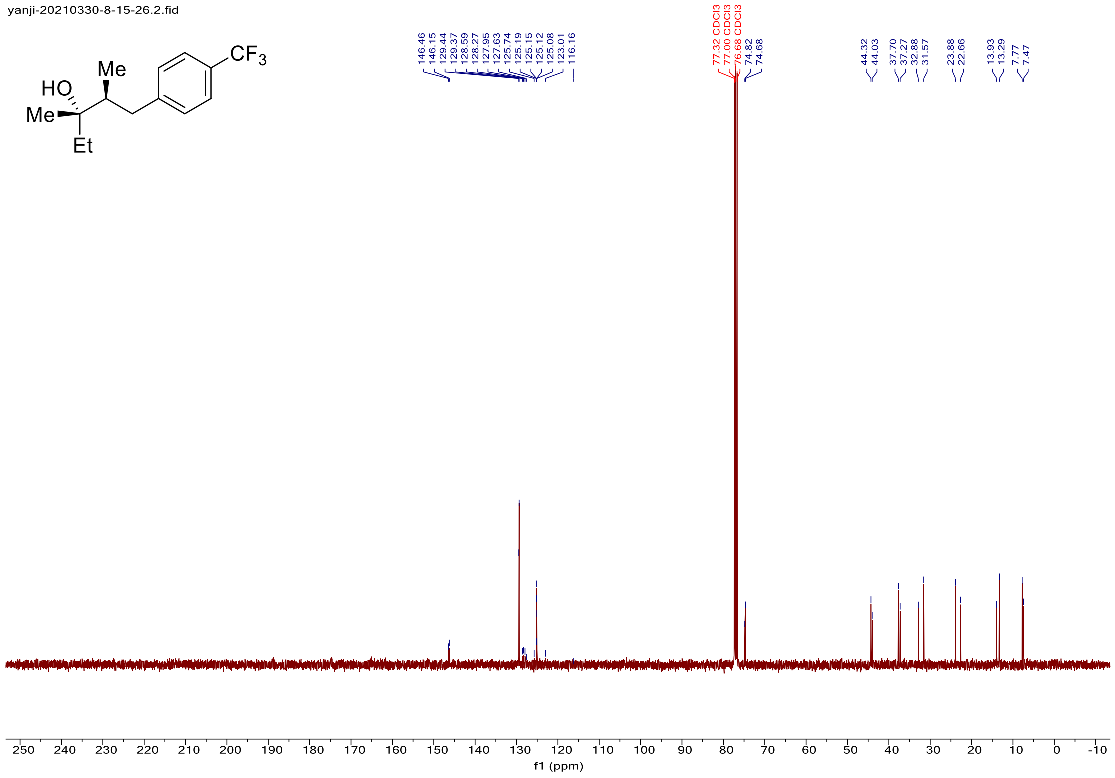
yanji-20210330-8-15-26.2.fid
HO
Me
Me
Et
CF3
77.32 CDCl3
77.00 CDCl3
76.68 CDCl3
f1 (ppm)

yanji-20220106-8-15-26-F.4.fid

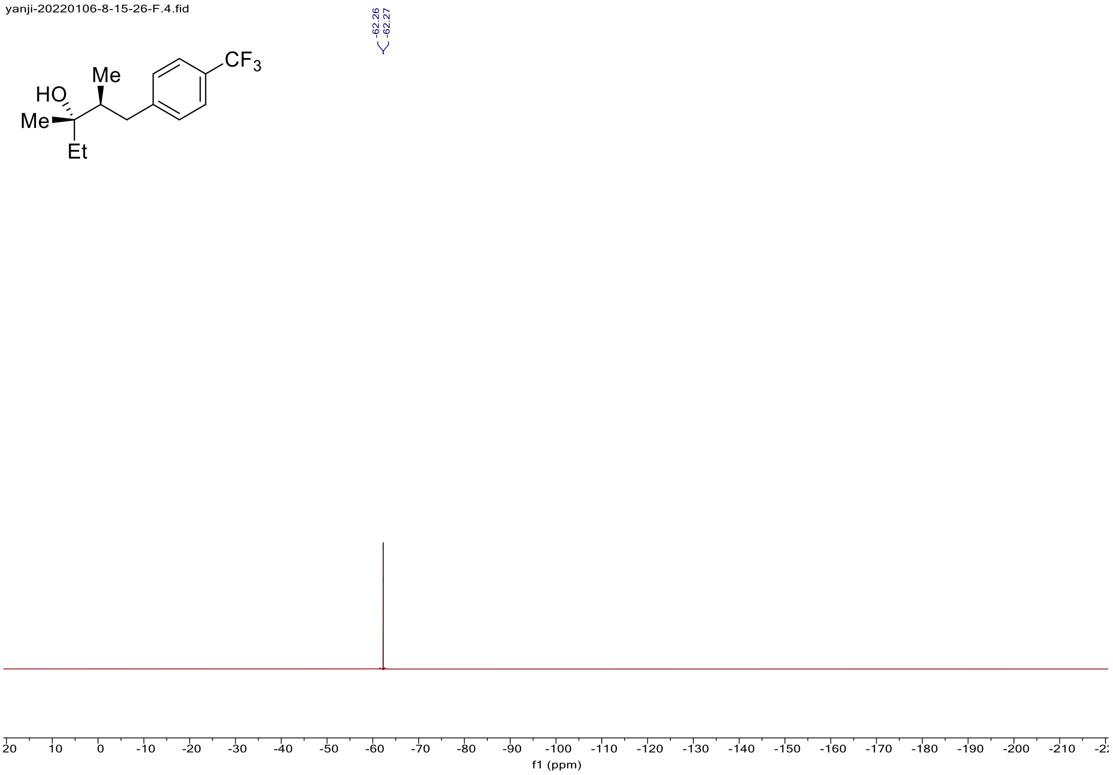


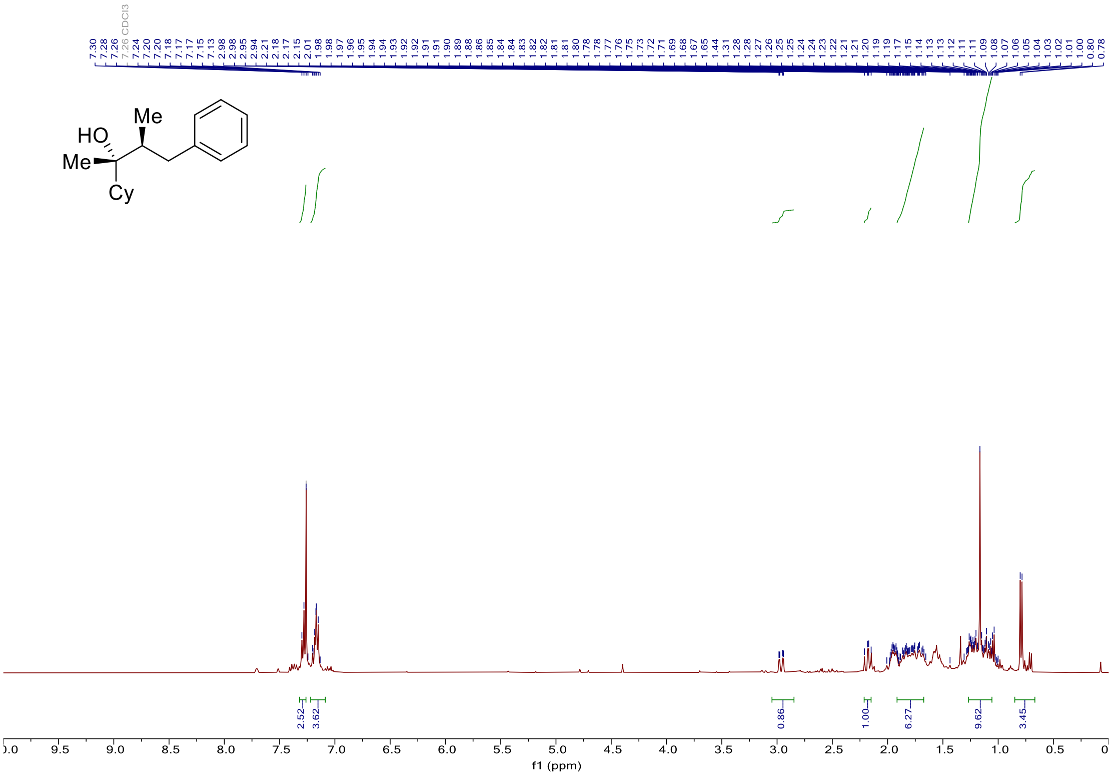

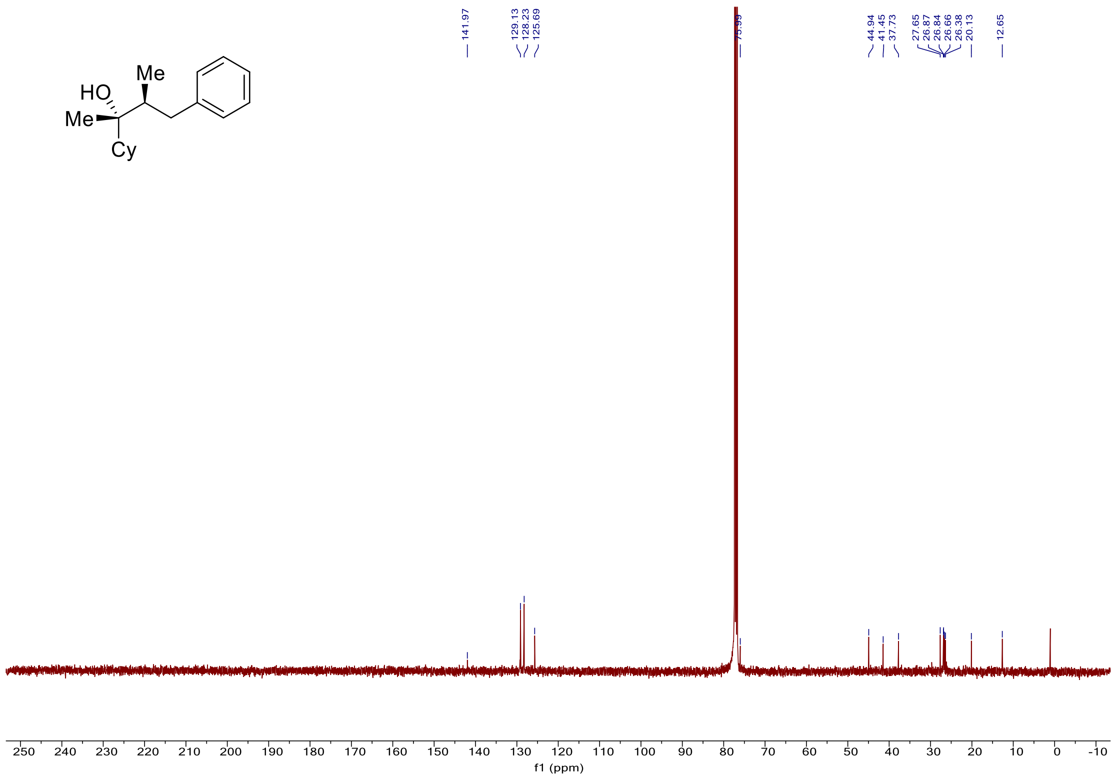
HO
Me
Me
Cy
141.97
129.13
128.23
125.69
44.94
41.45
37.73
27.65
26.87
26.84
26.66
26.38
20.13
12.65
f1 (ppm)

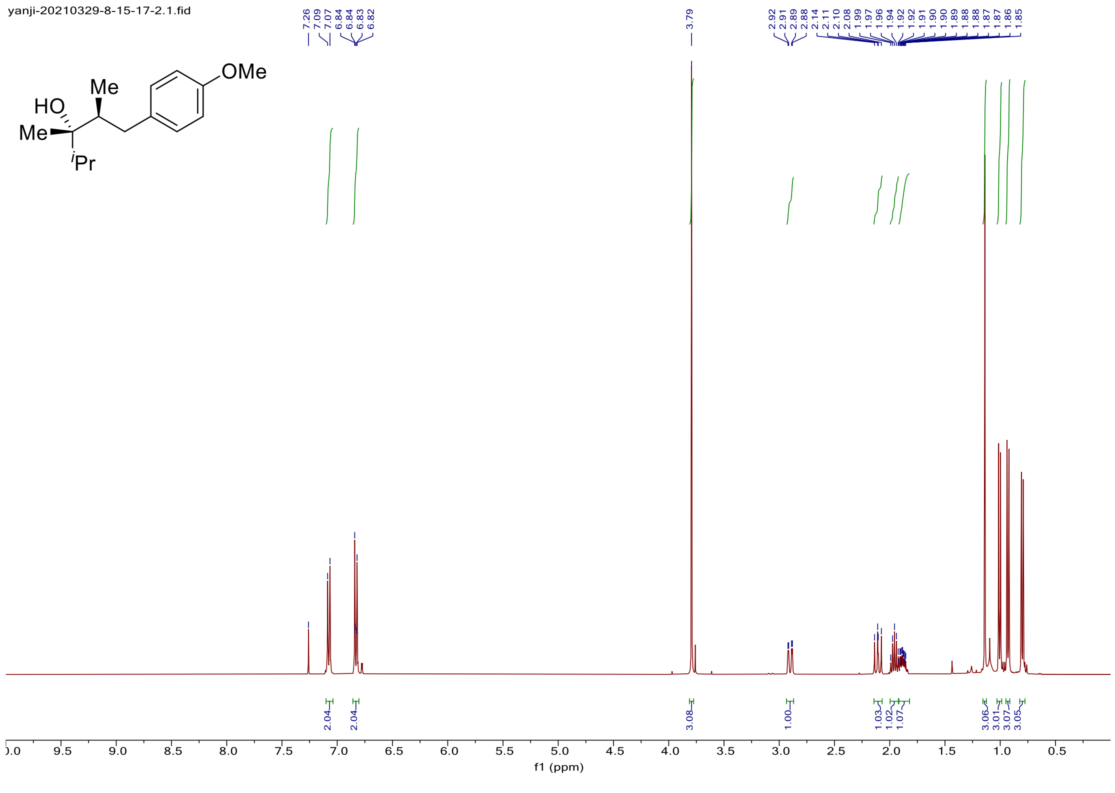
yanji-20210329-8-15-17-2.1.fid
HO
Me
Me
OMe
iPr
7.26
7.09
7.07
6.84
6.83
6.82
3.79
2.04
2.04
3.08
1.00
1.03
1.02
1.07
3.06
3.01
3.07
3.05
f1 (ppm)

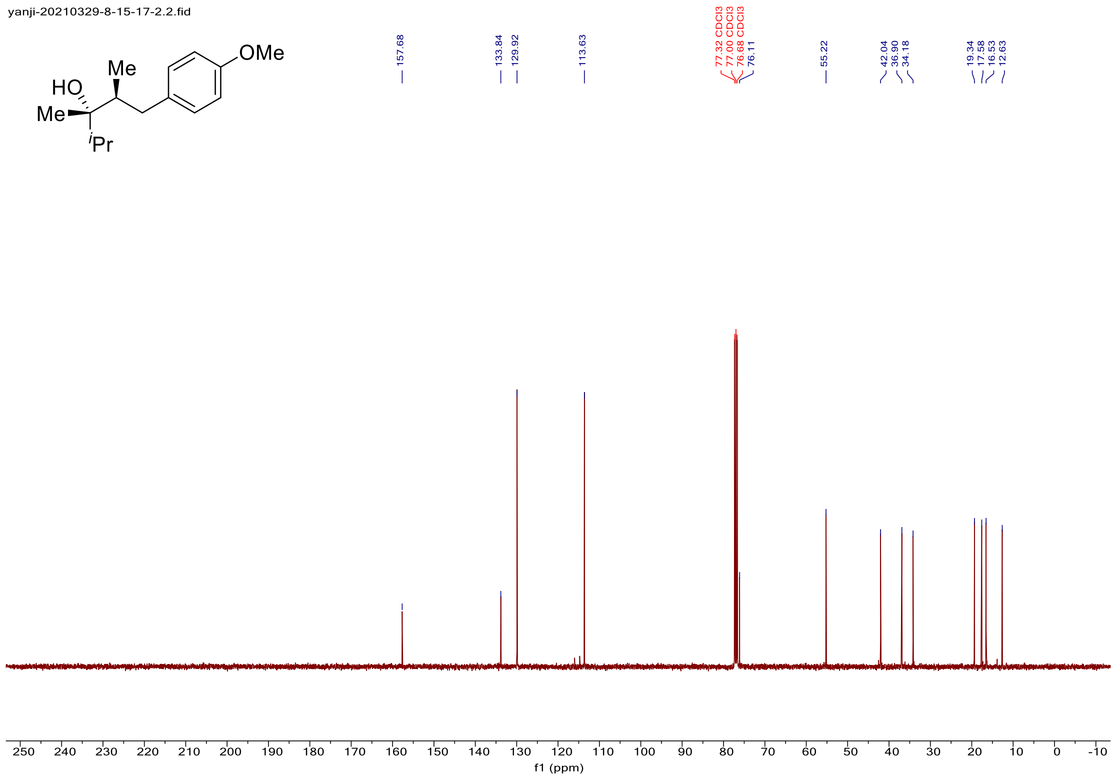

yanji-20210329-8-15-17-2.2.fid
HO
Me
Me
OMe
iPr
157.68
133.84
129.92
113.63
77.32 CDCl3
77.00 CDCl3
76.68 CDCl3
76.11
55.22
42.04
36.90
34.18
19.34
17.58
16.53
12.63
f1 (ppm)


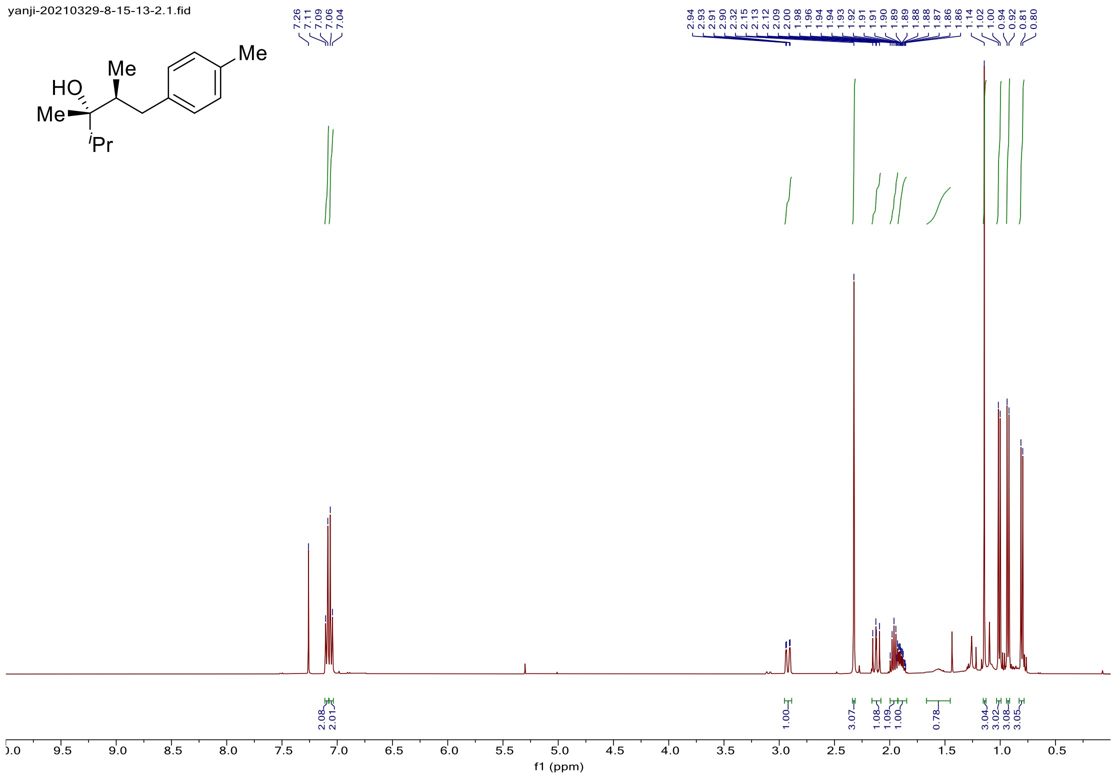

yanji-20210329-8-15-13-2.1.fid
HO
Me
Me
Me
iPr
7.26
7.11
7.09
7.06
7.04
2.94
2.93
2.91
2.90
2.32
2.15
2.13
2.12
2.09
2.00
1.98
1.96
1.94
1.94
1.93
1.92
1.91
1.90
1.89
1.88
1.88
1.87
1.86
1.14
1.02
1.00
0.94
0.92
0.81
0.80
2.08
2.01
1.00
3.07
1.08
1.09
1.00
0.78
3.04
3.02
3.08
3.05
f1 (ppm)

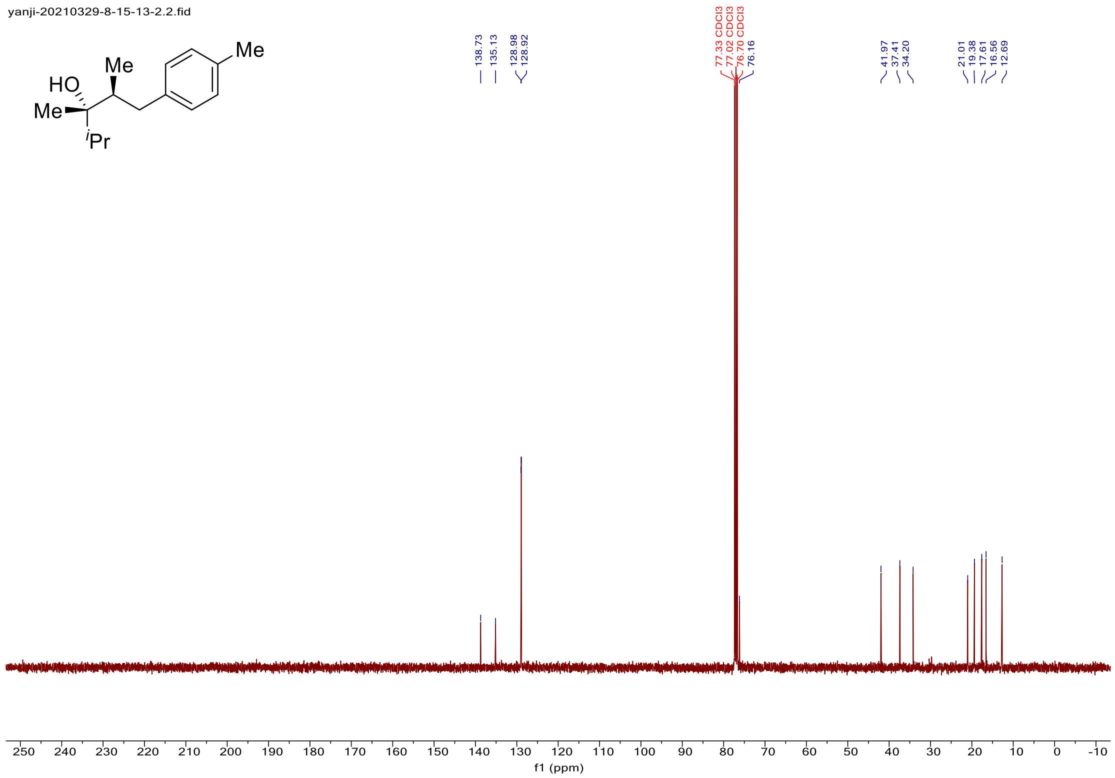
yanji-20210329-8-15-13-2.2.fid
HO
Me
Me
Me
iPr
138.73
135.13
128.98
128.92
77.33 CDCl3
77.02 CDCl3
76.70 CDCl3
76.16
41.97
37.41
34.20
21.01
19.38
17.61
16.56
12.69
f1 (ppm)

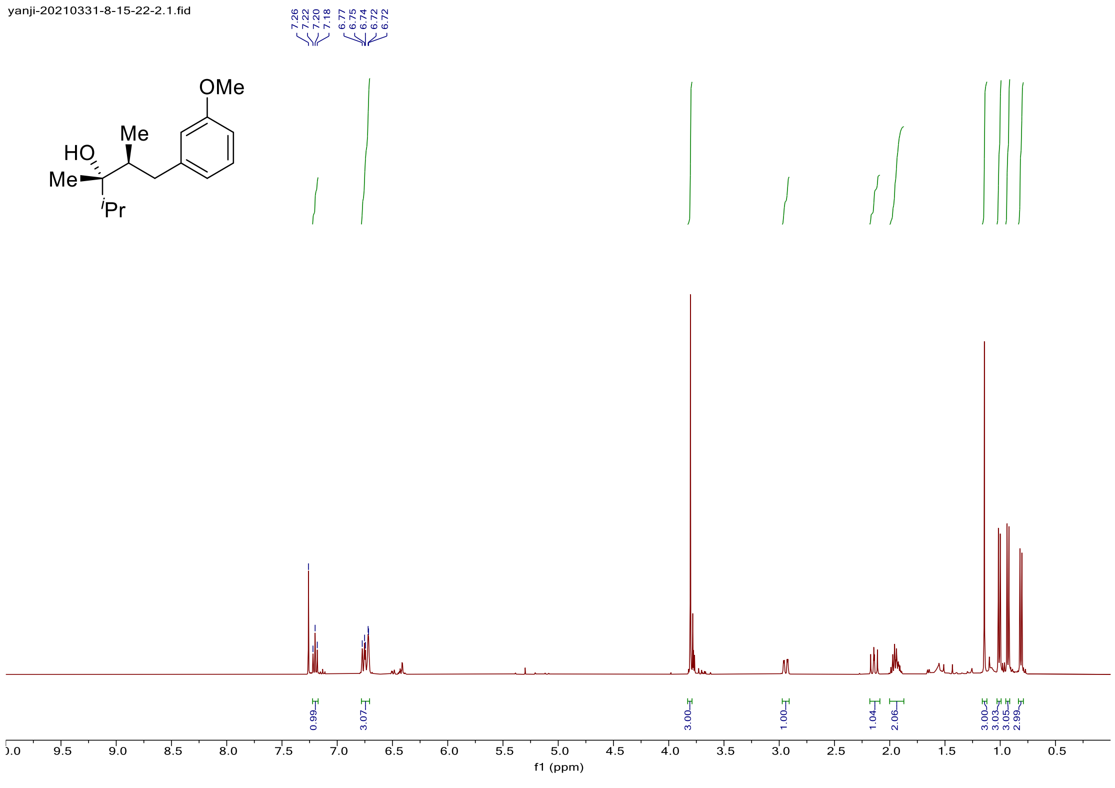
yanji-20210331-8-15-22-2.1.fid
OMe
Me
HO
Me
iPr
7.26
7.22
7.20
7.18
6.77
6.75
6.74
6.72
6.72
0.99
3.07
3.00
1.00
1.04
2.06
3.00
3.03
3.05
2.99
f1 (ppm)

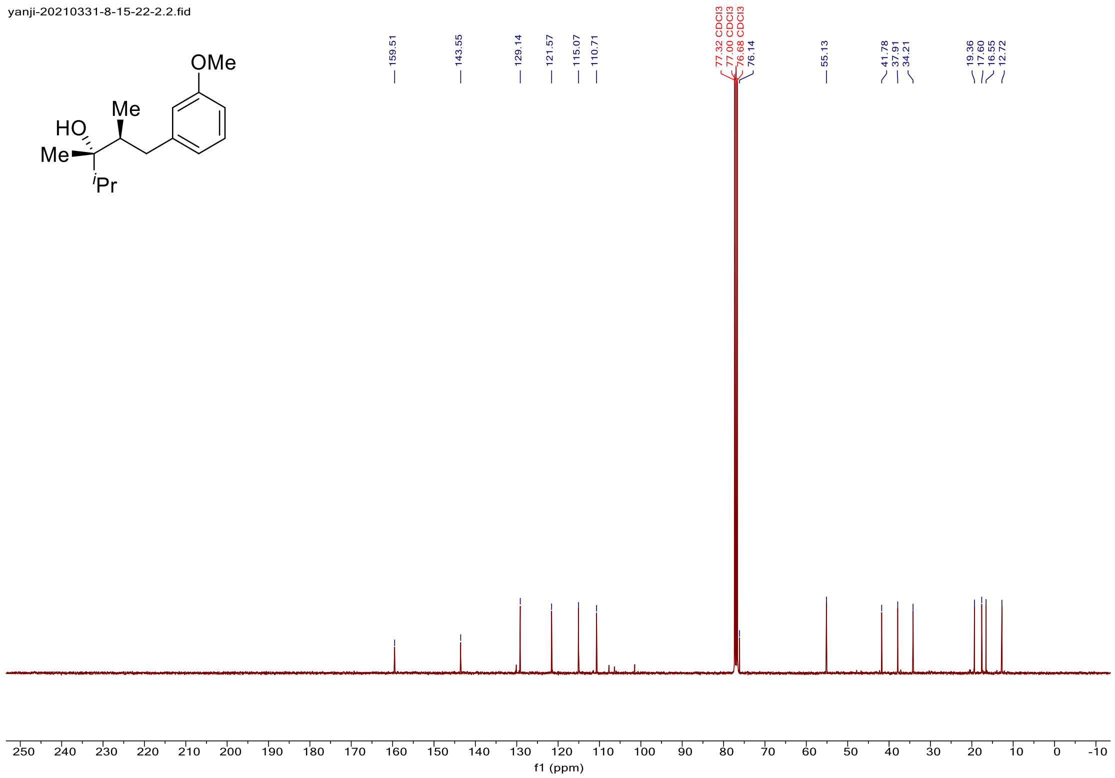
yanji-20210331-8-15-22-2.2.fid
OMe
Me
HO
Me
iPr
159.51
143.55
129.14
121.57
115.07
110.71
77.32 CDCl3
77.00 CDCl3
76.68 CDCl3
76.14
55.13
41.78
37.91
34.21
19.36
17.60
16.55
12.72
f1 (ppm)

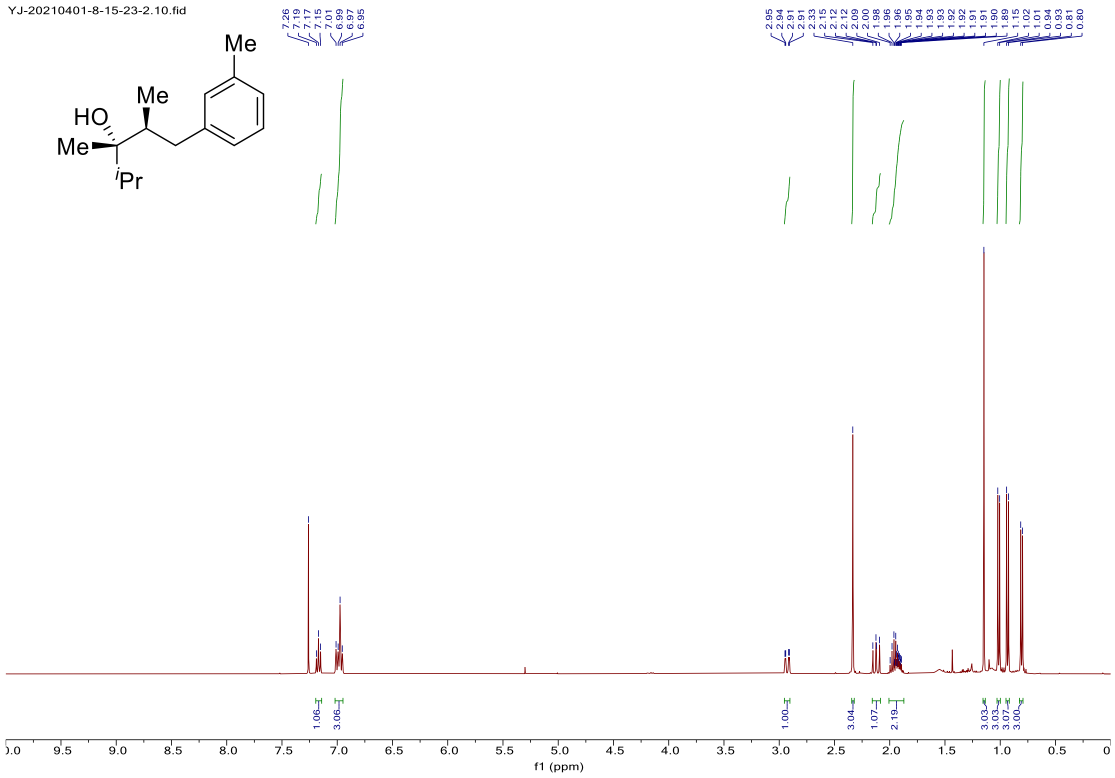
YJ-20210401-8-15-23-2.10.fid
Me
Me
HO
Me
iPr
7.26
7.19
7.17
7.15
7.01
6.99
6.97
6.95
2.95
2.94
2.91
2.91
2.33
2.15
2.12
2.12
2.09
2.00
1.98
1.96
1.95
1.94
1.93
1.93
1.92
1.92
1.91
1.91
1.90
1.89
1.15
1.02
1.01
0.94
0.93
0.81
0.80
1.06
3.06
1.00
3.04
1.07
2.19
3.03
3.03
3.07
3.00
f1 (ppm)

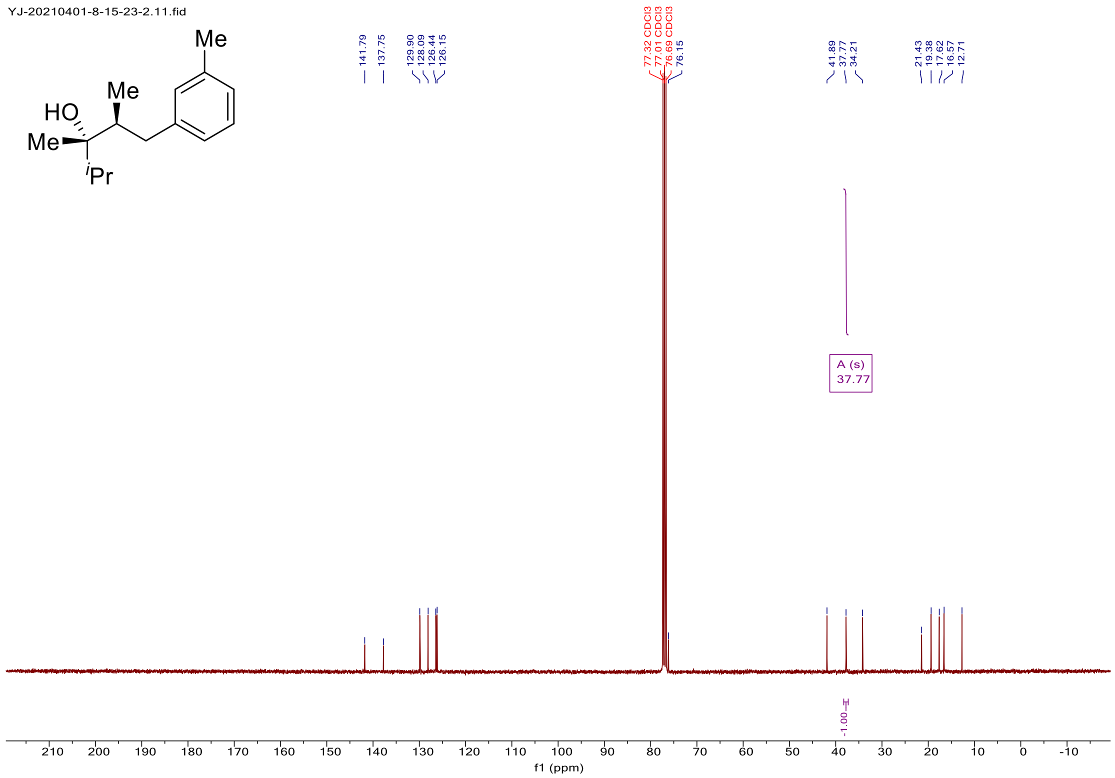

YJ-20210401-8-15-23-2.11.fid
Me
Me
HO
Me
iPr
141.79
137.75
129.90
128.09
126.44
126.15
77.32 CDCl3
77.01 CDCl3
76.69 CDCl3
76.15
41.89
37.77
34.21
21.43
19.38
17.62
16.57
12.71
A (s)
37.77
-1.00
210 200 190 180 170 160 150 140 130 120 110 100 90 80 70 60 50 40 30 20 10 0 -10
f1 (ppm)


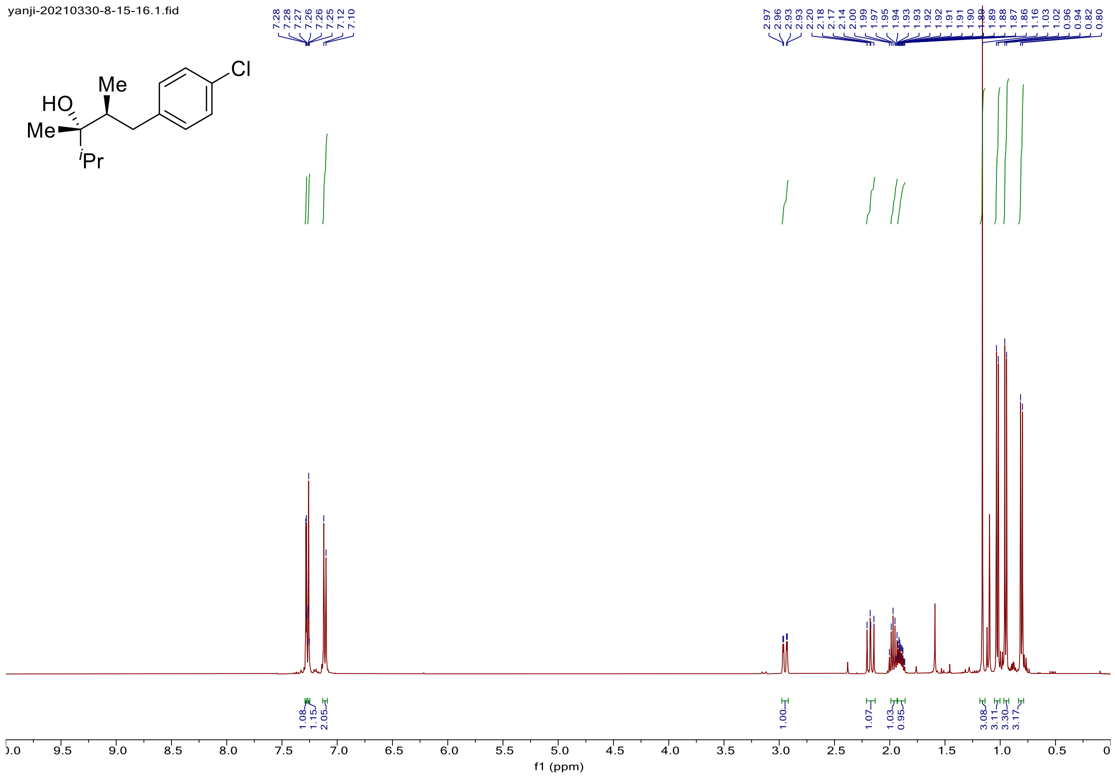

yanji-20210330-8-15-16.1.fid
Cl
Me
HO
Me
iPr
7.28
7.28
7.27
7.26
7.25
7.12
7.10
2.97
2.96
2.93
2.93
2.20
2.18
2.17
2.14
2.00
1.99
1.97
1.95
1.94
1.93
1.93
1.92
1.91
1.91
1.90
1.89
1.89
1.88
1.87
1.86
1.16
1.03
1.02
0.96
0.94
0.82
0.80
1.08
1.15
2.05
1.00
1.07
1.03
0.95
3.08
3.11
3.30
3.17
9.5 9.0 8.5 8.0 7.5 7.0 6.5 6.0 5.5 5.0 4.5 4.0 3.5 3.0 2.5 2.0 1.5 1.0 0.5 0
f1 (ppm)

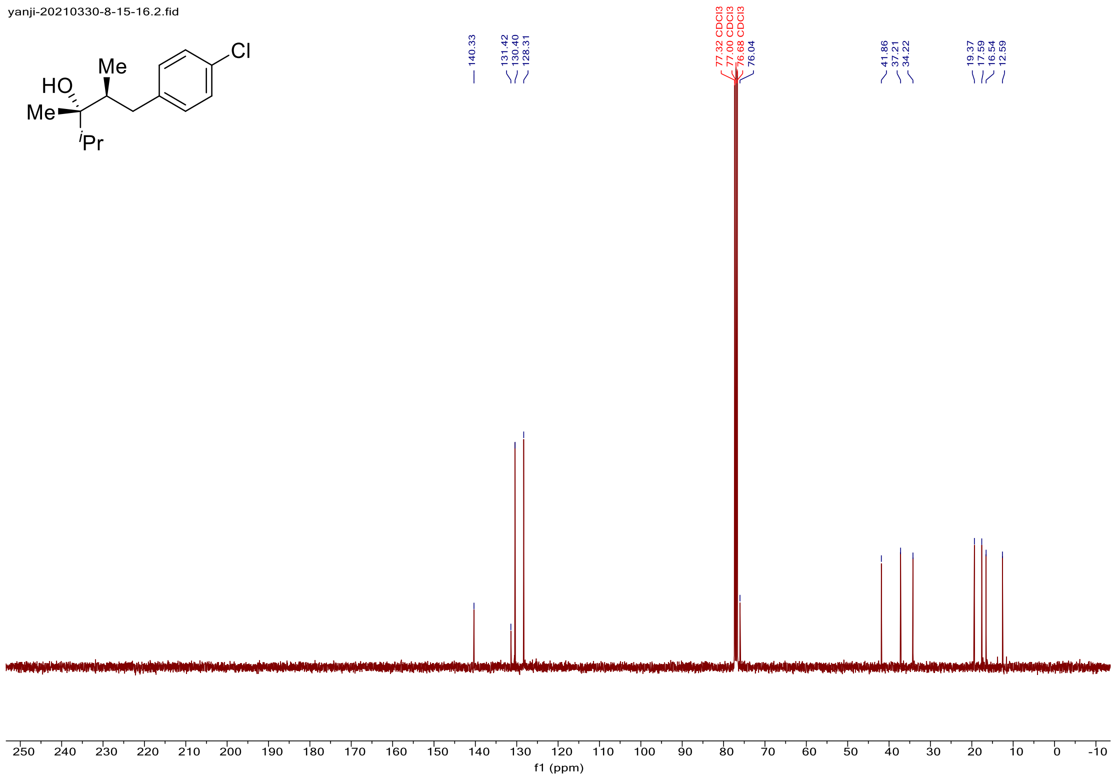
yanji-20210330-8-15-16.2.fid
HO
Me
Me
Cl
iPr
140.33
131.42
130.40
128.31
77.32 CDCl3
77.00 CDCl3
76.68 CDCl3
76.04
41.86
37.21
34.22
19.37
17.59
16.54
12.59
f1 (ppm)

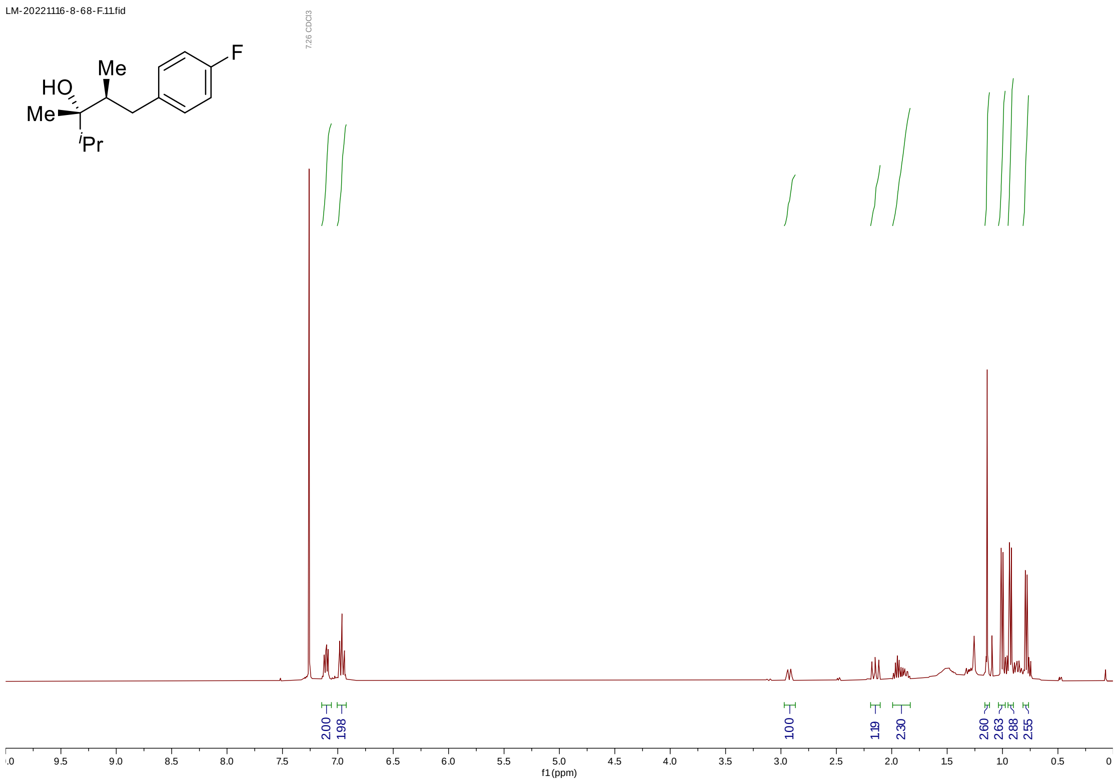
LM-20221116-8-68-F.11.fid
HO
Me
Me
F
iPr
7.26 CDCl3
2.00
1.98
1.00
1.19
2.30
2.60
2.63
2.88
2.55
f1 (ppm)

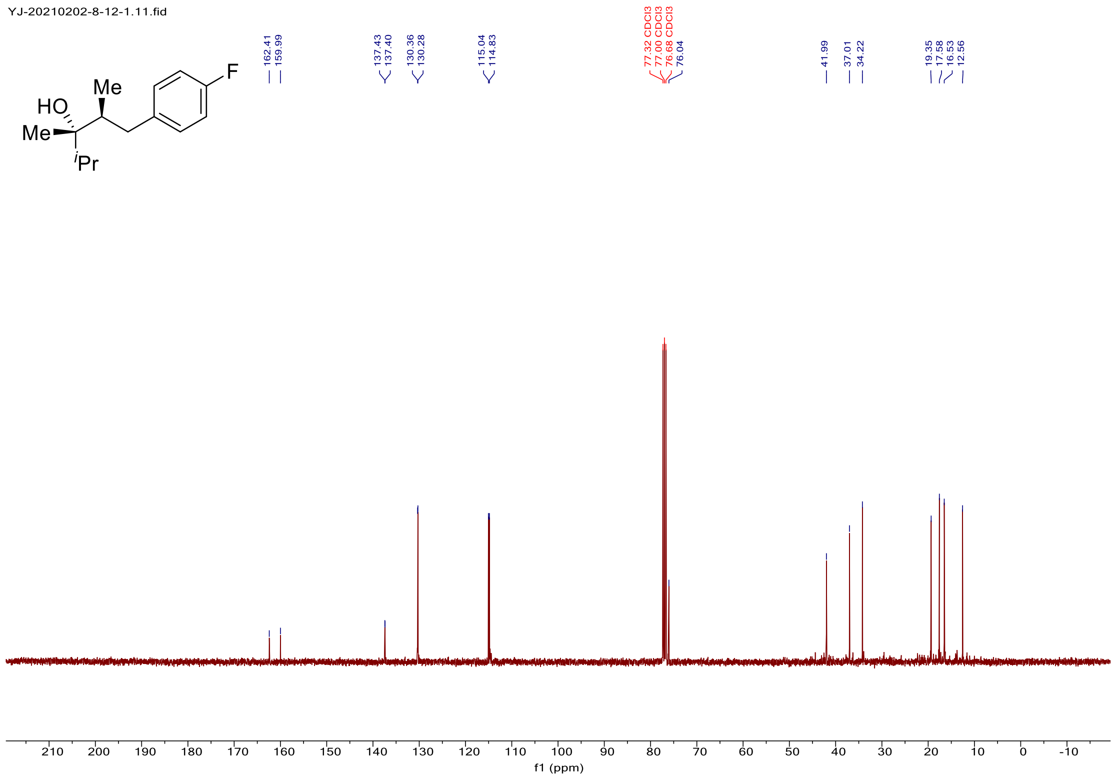
YJ-20210202-8-12-1.11.fid
162.41
159.99
137.43
137.40
130.36
130.28
115.04
114.83
77.32 CDCl3
77.00 CDCl3
76.68 CDCl3
76.04
41.99
37.01
34.22
19.35
17.58
16.53
12.56
f1 (ppm)

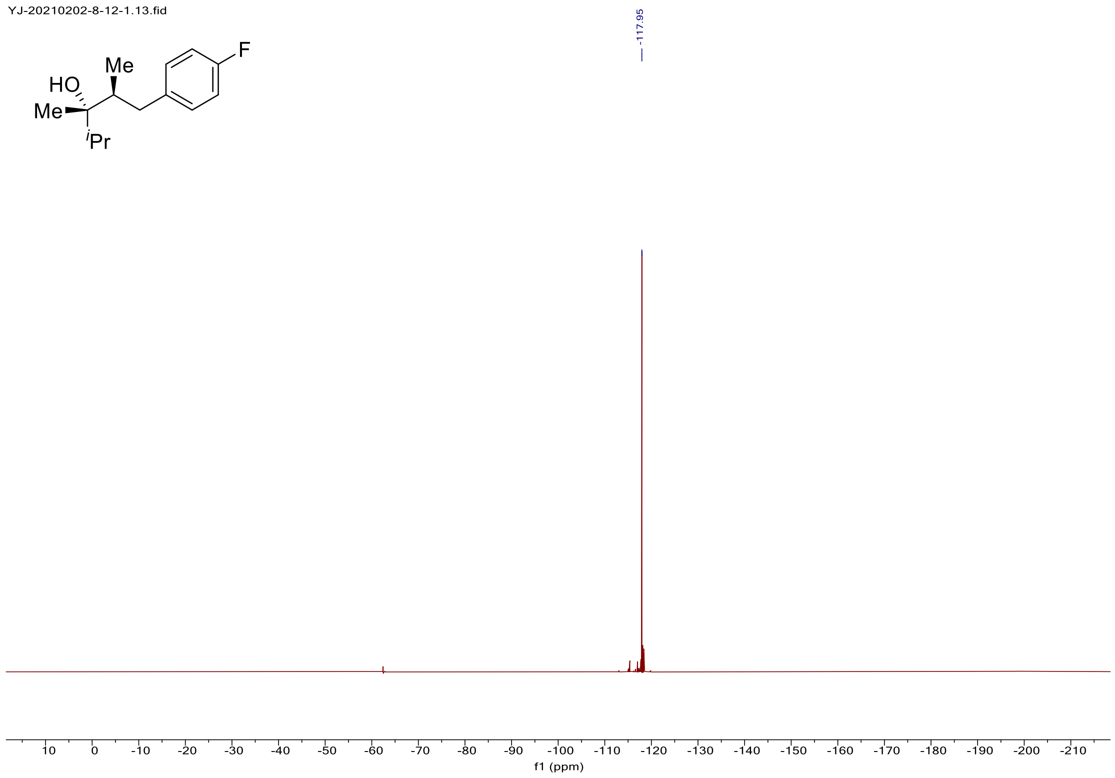
YJ-20210202-8-12-1.13.fid
-117.95
f1 (ppm)

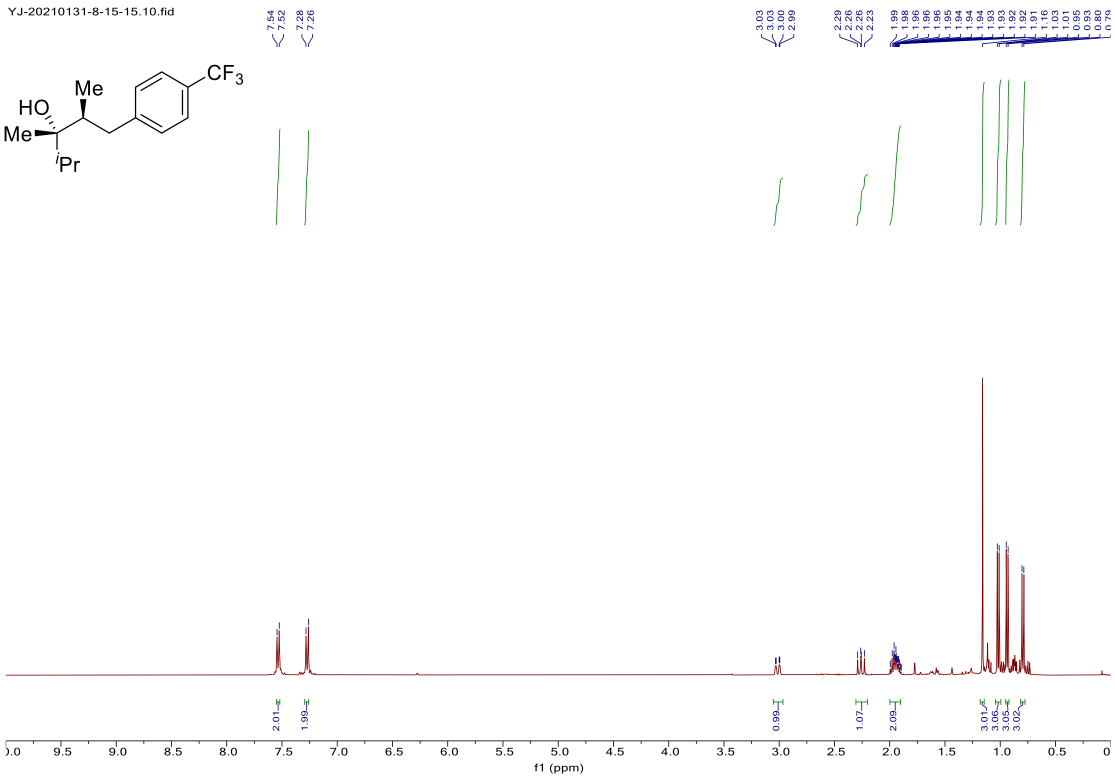
YJ-20210131-8-15-15.10.fid
HO
Me
Me
CF3
iPr
7.54
7.52
7.28
7.26
3.03
3.03
3.00
2.99
2.29
2.26
2.26
2.23
2.01
1.99
0.99
1.07
2.09
3.01
3.06
3.05
3.02
f1 (ppm)

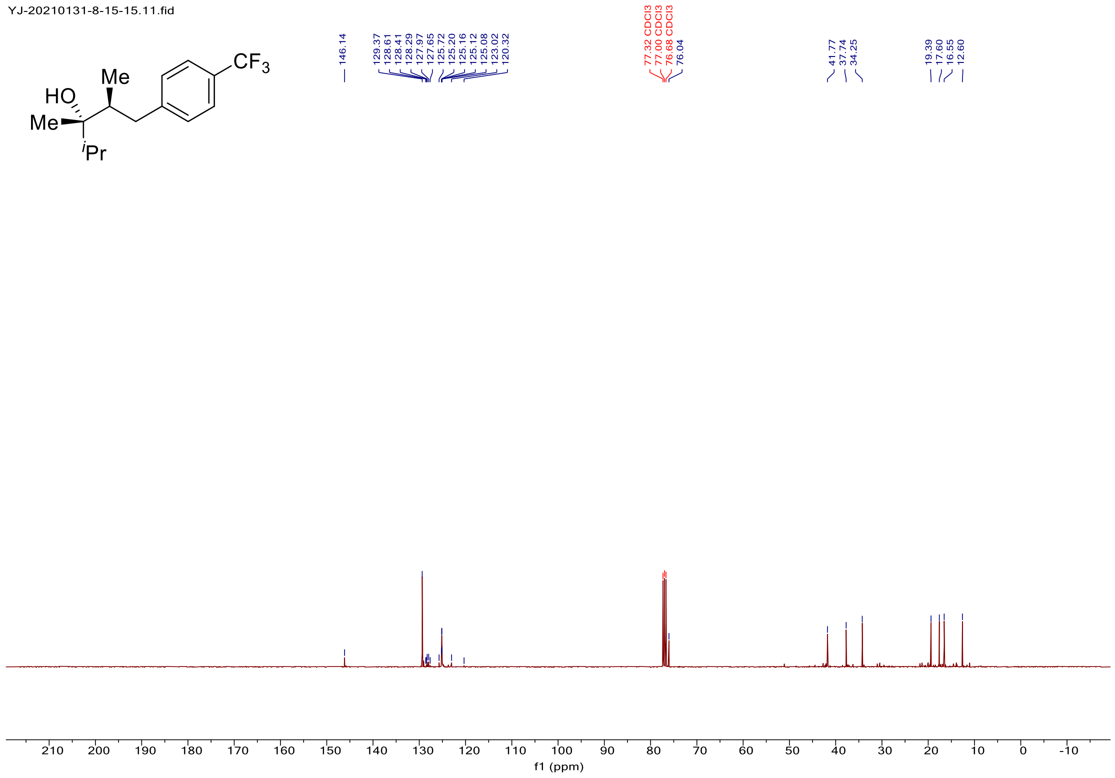
YJ-20210131-8-15-15.11.fid
HO
Me
Me
CF3
iPr
146.14
129.37
128.61
128.41
128.29
127.97
127.65
125.72
125.20
125.16
125.12
125.08
123.02
120.32
77.32 CDCl3
77.00 CDCl3
76.68 CDCl3
76.04
41.77
37.74
34.25
19.39
17.60
16.55
12.60
f1 (ppm)

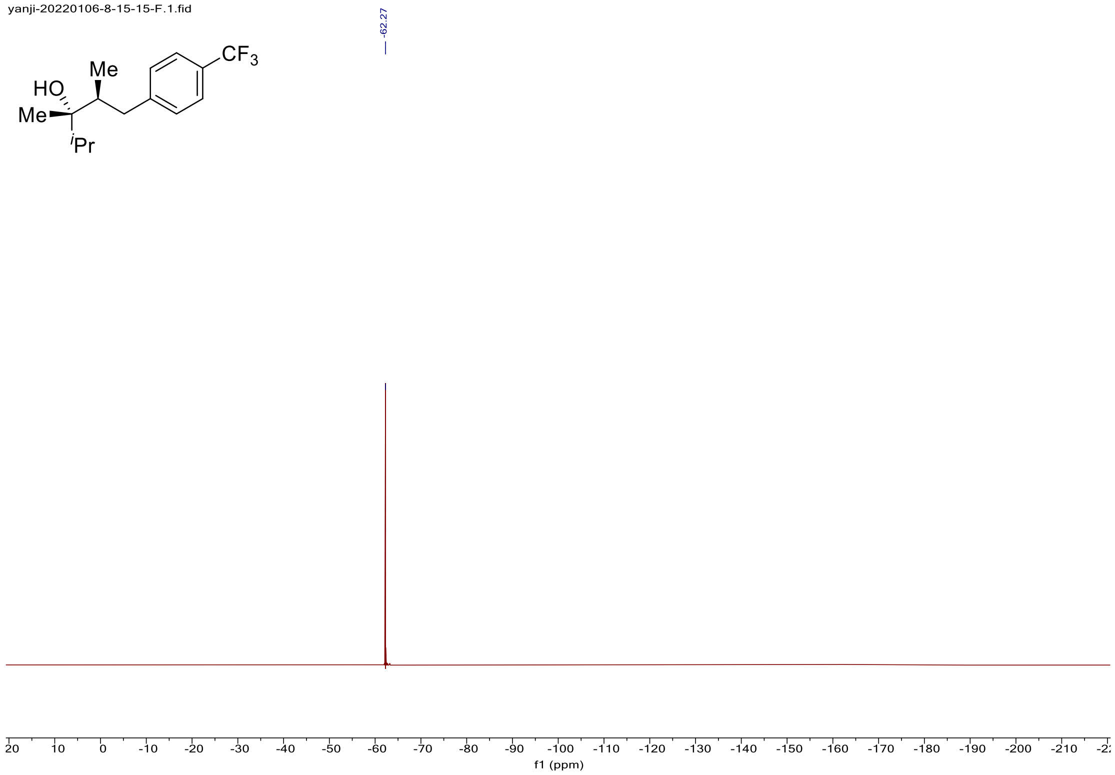
yanji-20220106-8-15-15-F.1.fid
-62.27
f1 (ppm)

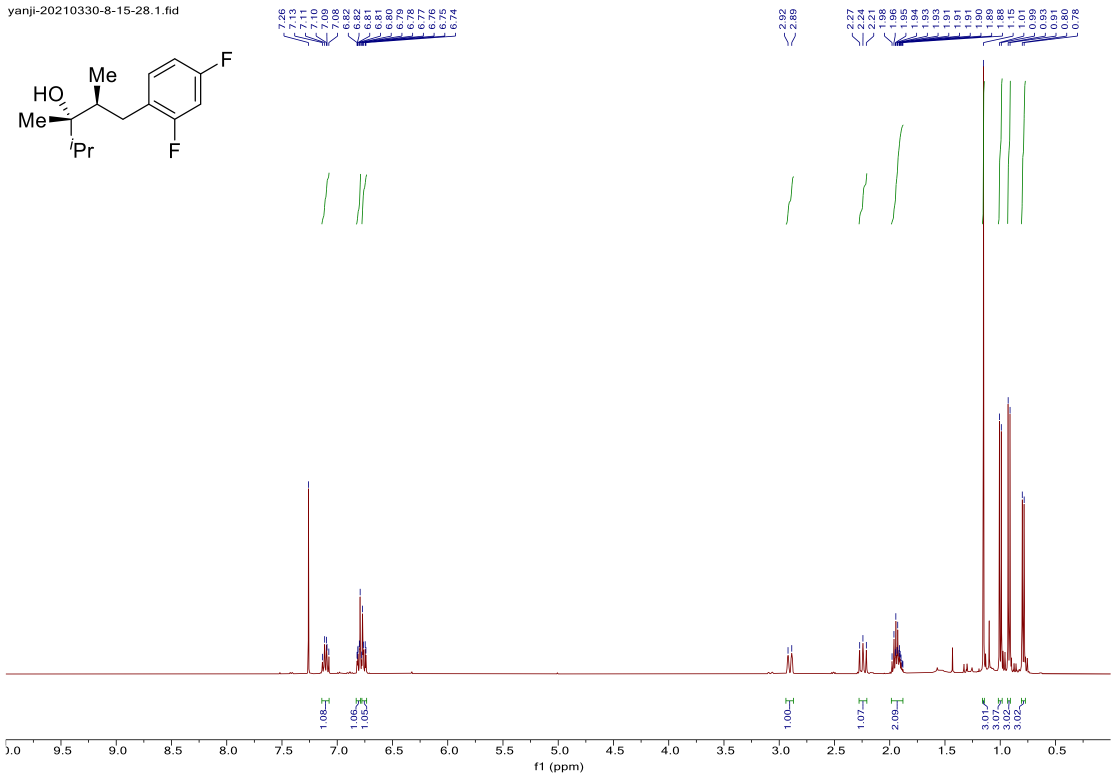
yanji-20210330-8-15-28.1.fid
f1 (ppm)

yanji-20210330-8-15-28.2.fid

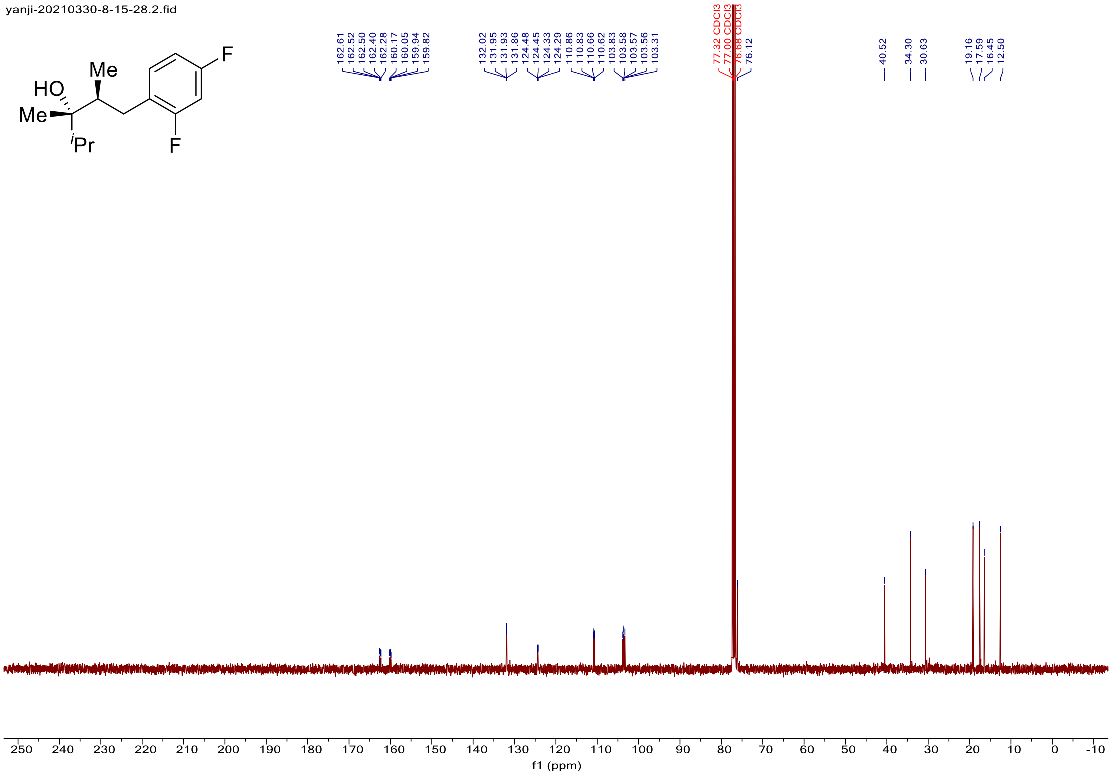

yanji-20220107-8-15-28-F.1.fid

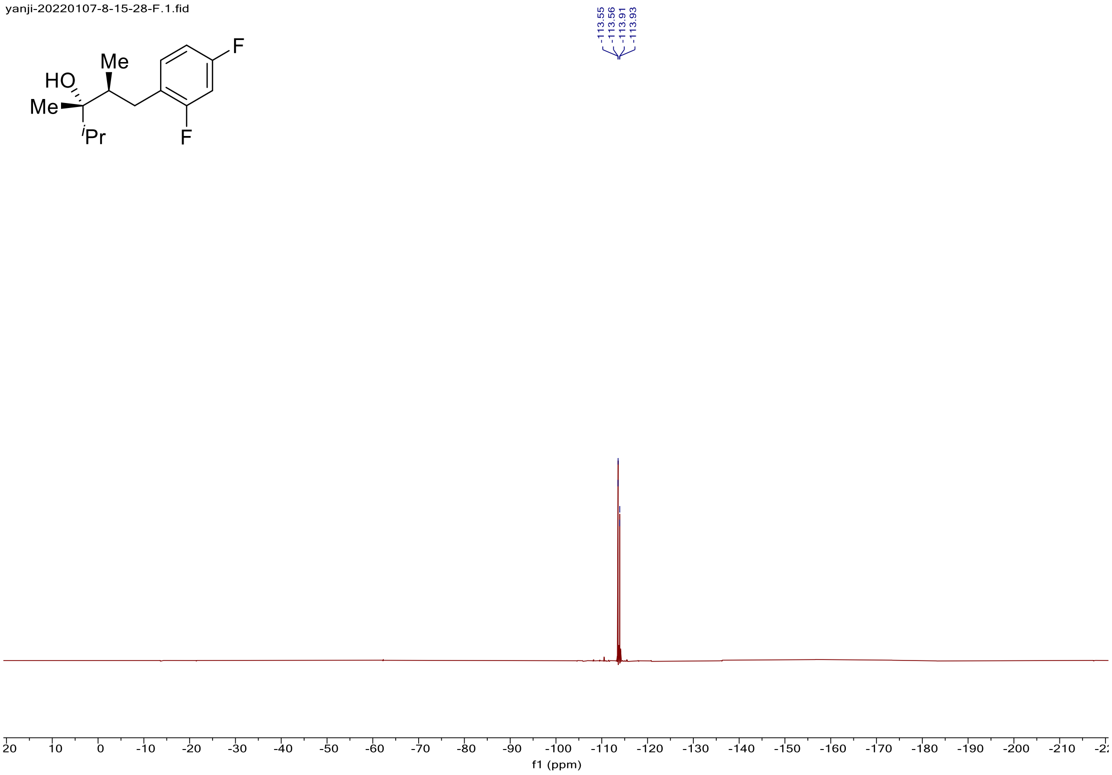

yanji-20210330-8-15-18.1.fid

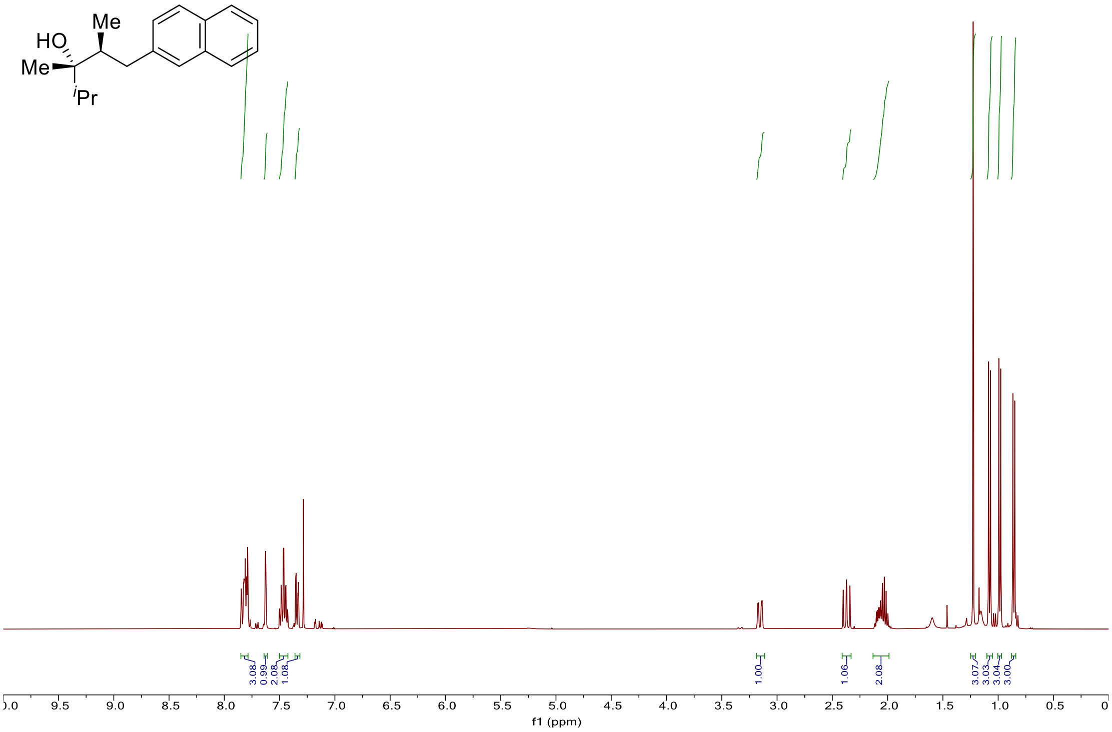


yanji-20210330-8-15-18.2.fid

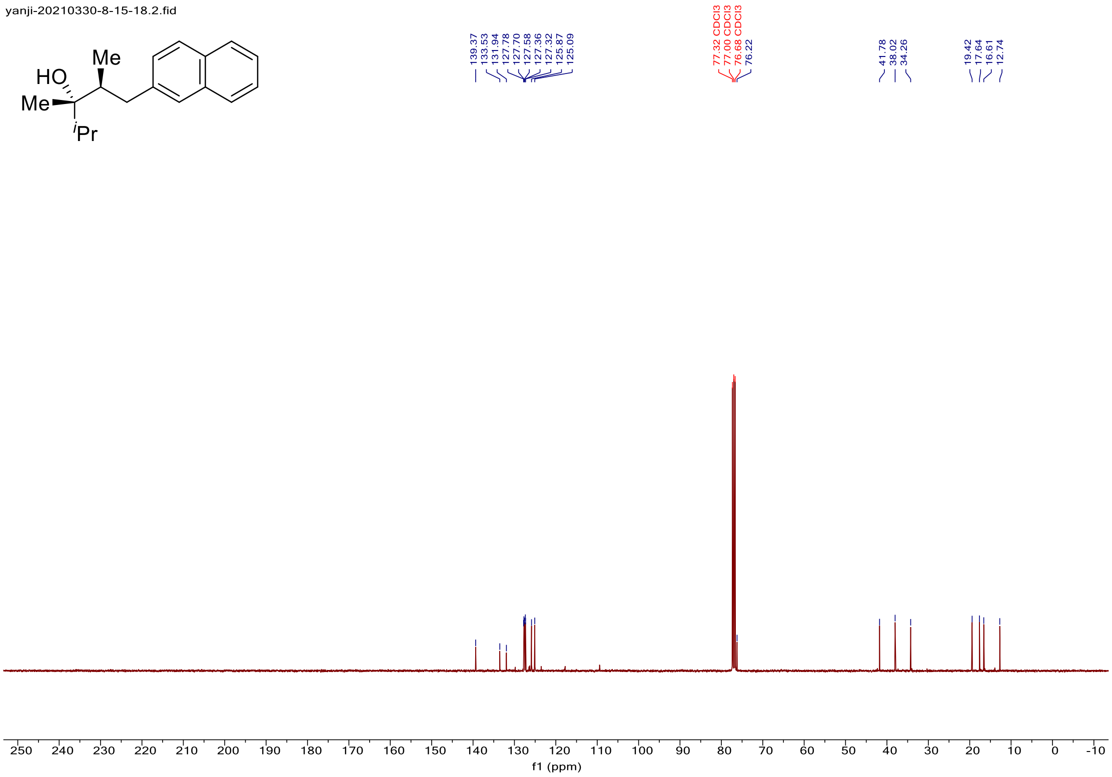

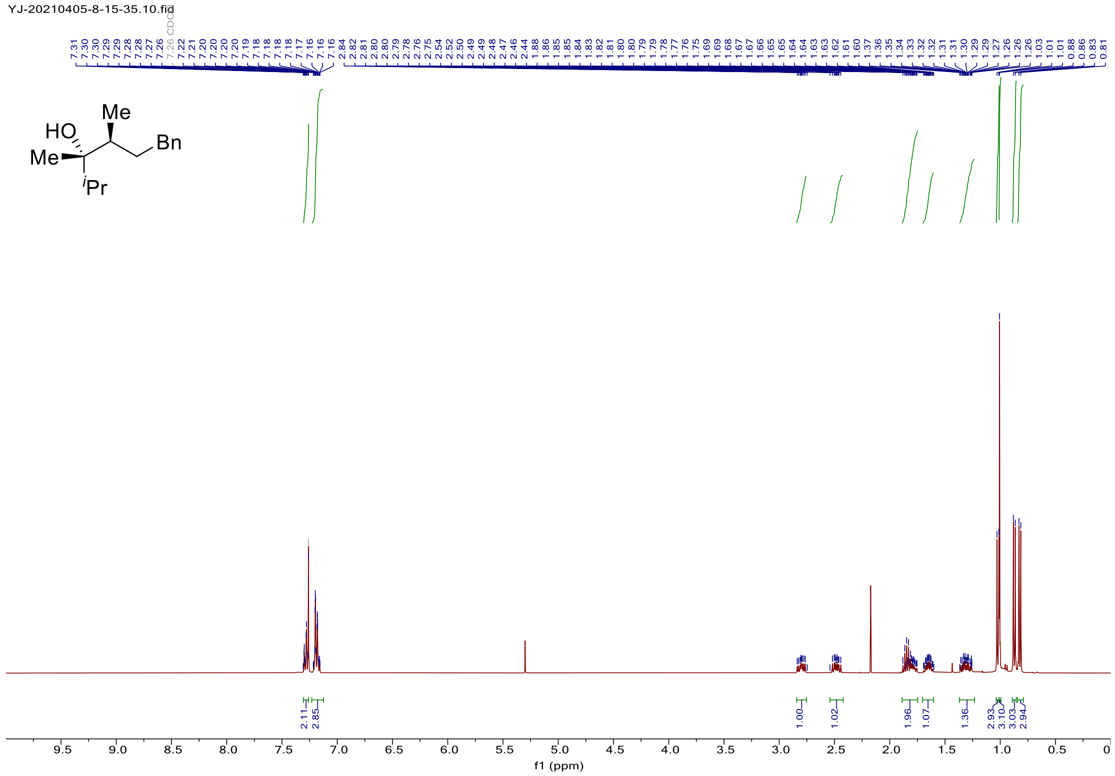
YJ-20210405-8-15-35.10.fid
7.31 7.30 7.29 7.29 7.28 7.28 7.27 7.26 CDCl3 7.22 7.21 7.20 7.20 7.20 7.20 7.19 7.18 7.18 7.18 7.18 7.17 7.16 7.16 7.16 2.84 2.82 2.81 2.80 2.80 2.79 2.78 2.76 2.75 2.54 2.52 2.50 2.49 2.49 2.48 2.47 2.46 2.44 1.88 1.86 1.85 1.85 1.84 1.83 1.82 1.81 1.80 1.80 1.79 1.79 1.78 1.77 1.76 1.75 1.69 1.69 1.68 1.67 1.67 1.66 1.65 1.65 1.64 1.63 1.63 1.62 1.61 1.60 1.37 1.36 1.35 1.34 1.33 1.32 1.32 1.31 1.30 1.29 1.29 1.27 1.26 1.26 1.26 1.03 1.01 1.01 0.88 0.86 0.83 0.81
HO
Me
Me
Bn
iPr
2.11
2.85
1.00
1.02
1.96
1.07
1.36
2.93
3.10
3.03
2.94
f1 (ppm)

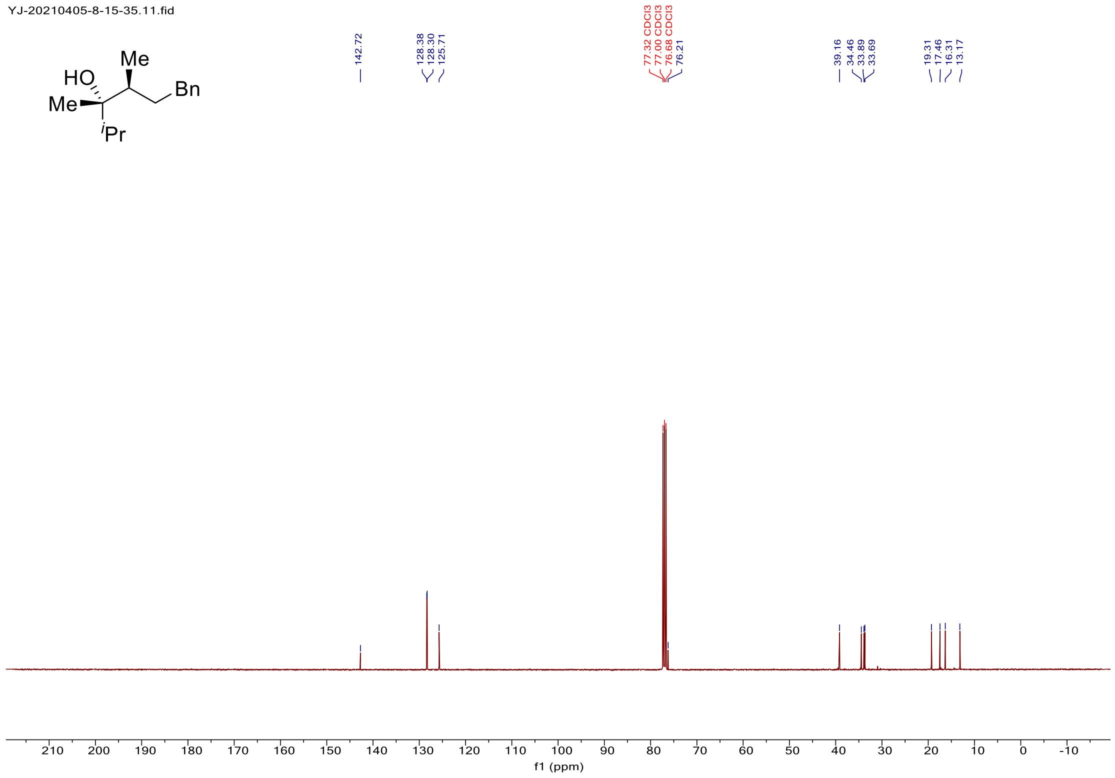
YJ-20210405-8-15-35.11.fid
142.72
128.38 128.30 125.71
77.32 CDCl3 77.00 CDCl3 76.68 CDCl3 76.21
39.16 34.46 33.89 33.69
19.31 17.46 16.31 13.17
HO
Me
Me
Bn
iPr
f1 (ppm)

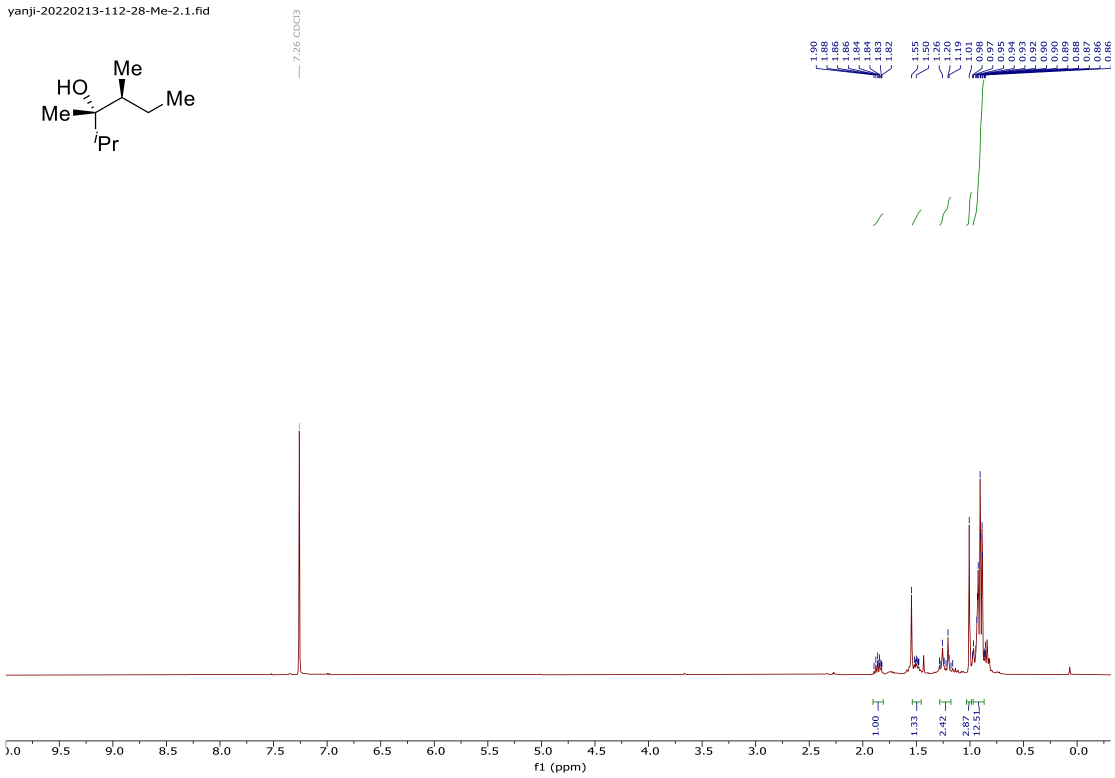
yanji-20220213-112-28-Me-2.1.fid
HO
Me
Me
Me
iPr
7.26 CDCl3
1.90 1.88 1.86 1.86 1.84 1.84 1.83 1.82 1.55 1.50 1.26 1.20 1.19 1.01 0.98 0.97 0.95 0.94 0.93 0.92 0.90 0.90 0.89 0.88 0.87 0.86 0.86
1.00 1.33 2.42 2.87 12.51
f1 (ppm)

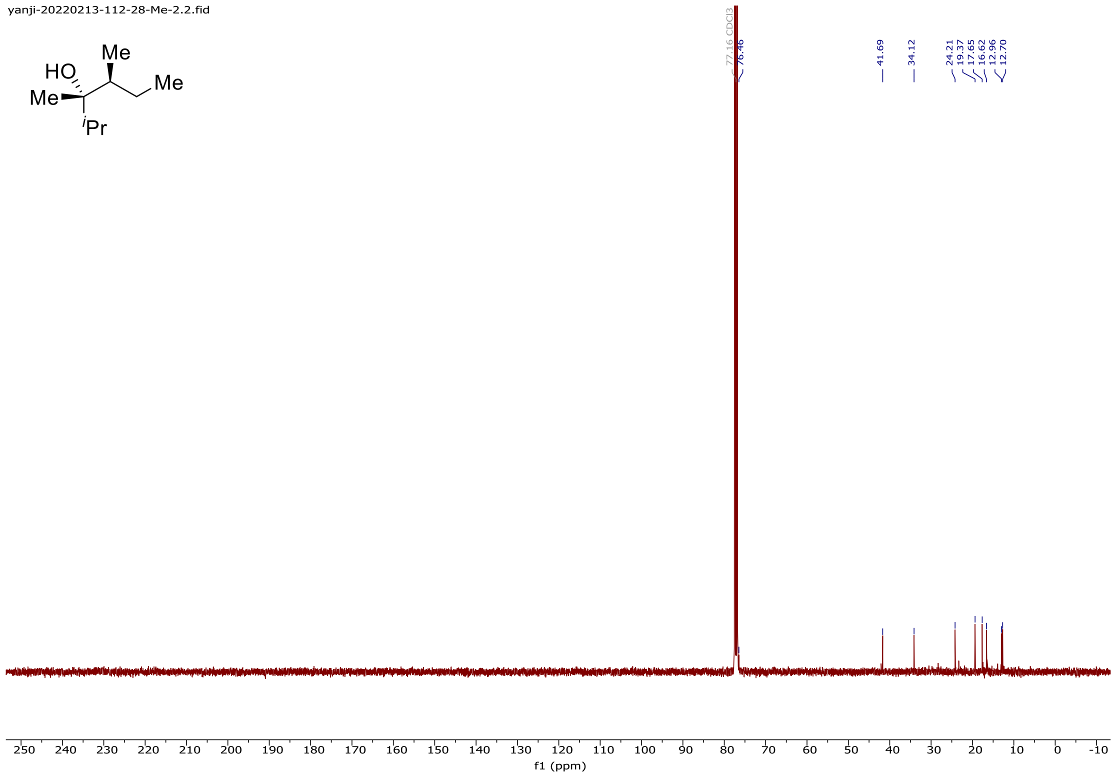
yanji-20220213-112-28-Me-2.2.fid
HO
Me
Me
Me
iPr
77.16 CDCl3
76.46
41.69
34.12
24.21 19.37 17.65 16.62 12.96 12.70
f1 (ppm)

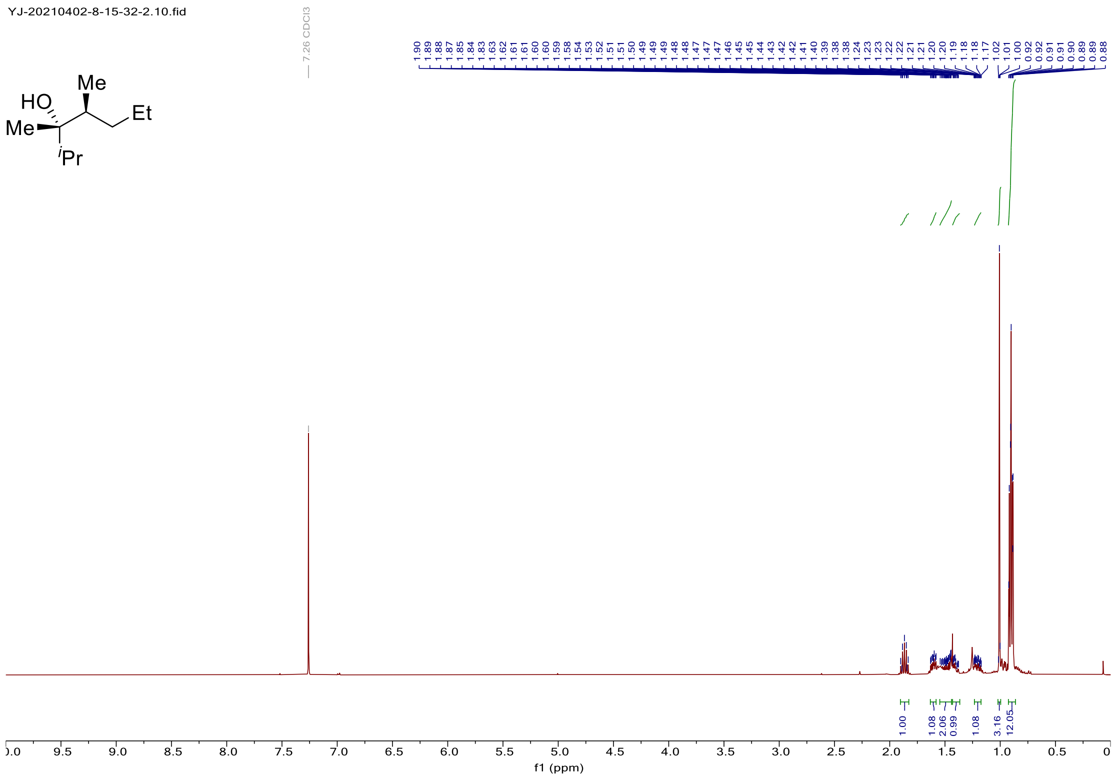
YJ-20210402-8-15-32-2.10.fid
Me
HO
Et
Me
iPr
7.26 CDCl3
1.90 1.89 1.88 1.87 1.85 1.84 1.83 1.63 1.62 1.61 1.61 1.60 1.60 1.59 1.58 1.54 1.53 1.52 1.51 1.51 1.50 1.49 1.49 1.49 1.48 1.48 1.47 1.47 1.46 1.45 1.45 1.44 1.43 1.42 1.42 1.41 1.40 1.39 1.38 1.38 1.24 1.23 1.23 1.22 1.22 1.21 1.21 1.20 1.20 1.19 1.18 1.18 1.17 1.02 1.01 1.00 0.92 0.92 0.91 0.91 0.90 0.89 0.89 0.88
1.00
1.08
2.06
0.99
1.08
3.16
12.05
f1 (ppm)

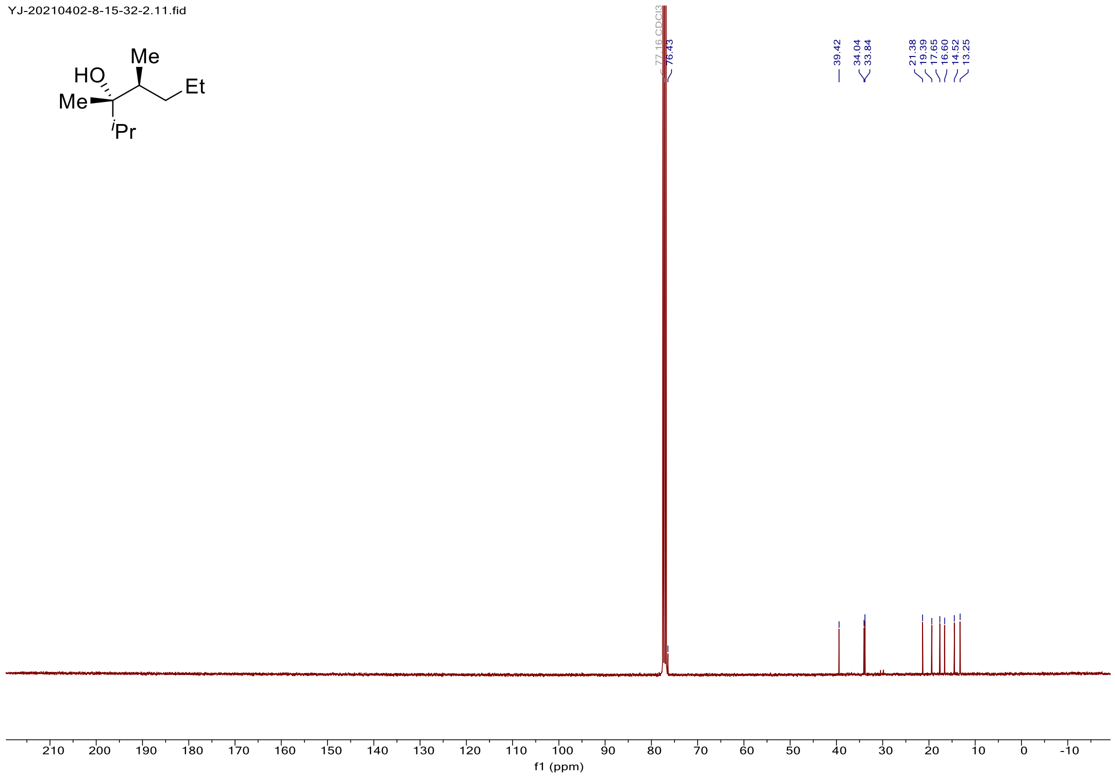
YJ-20210402-8-15-32-2.11.fid
Me
HO
Et
Me
iPr
77.16 CDCl3
76.43
39.42
34.04
33.84
21.38
19.39
17.65
16.60
14.52
13.25
f1 (ppm)

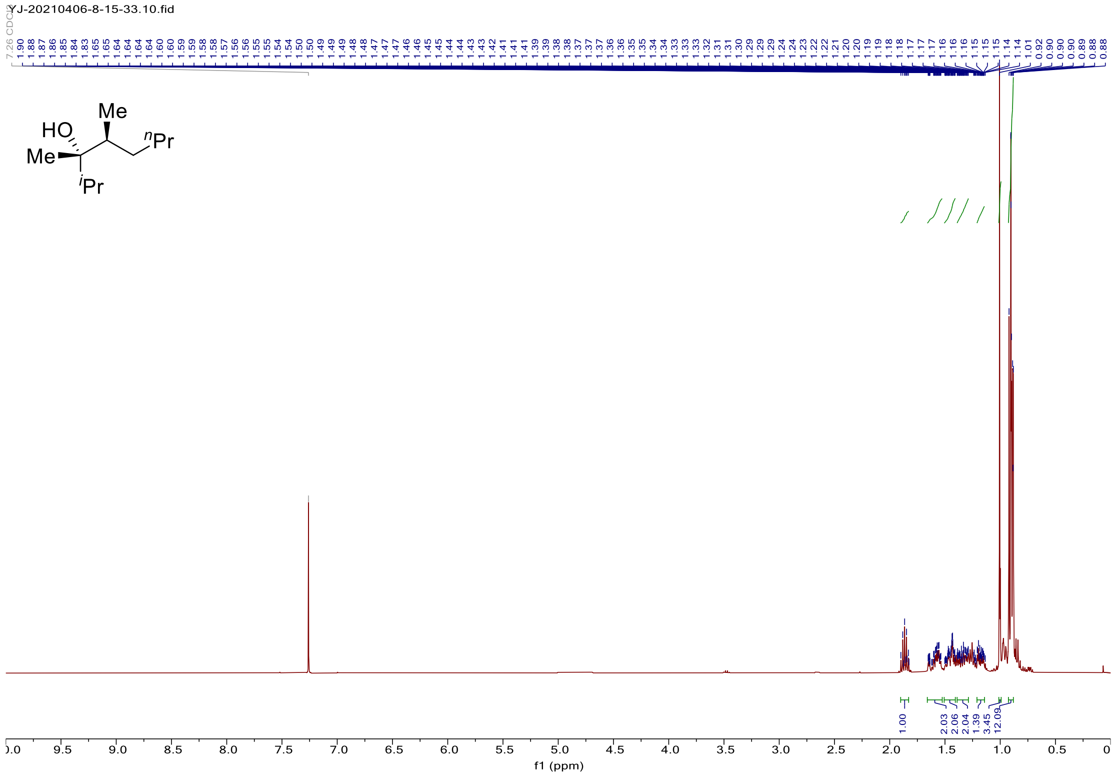
YJ-20210406-8-15-33.10.fid
7.26 CDCl3
1.90 1.88 1.87 1.86 1.85 1.84 1.83 1.65 1.65 1.64 1.64 1.64 1.60 1.60 1.59 1.59 1.58 1.58 1.57 1.56 1.56 1.55 1.55 1.54 1.54 1.50 1.50 1.49 1.49 1.49 1.48 1.48 1.47 1.47 1.46 1.46 1.45 1.45 1.44 1.43 1.43 1.42 1.42 1.41 1.41 1.39 1.39 1.38 1.38 1.37 1.37 1.37 1.36 1.36 1.35 1.35 1.34 1.34 1.33 1.33 1.33 1.32 1.31 1.31 1.30 1.29 1.29 1.29 1.24 1.24 1.23 1.22 1.22 1.21 1.21 1.20 1.19 1.19 1.19 1.18 1.18 1.17 1.17 1.17 1.16 1.16 1.16 1.15 1.15 1.15 1.14 1.14 1.01 0.92 0.90 0.90 0.90 0.89 0.88 0.88
HO
Me
Me
nPr
iPr
1.00
2.03
2.06
2.04
1.39
3.45
12.09
f1 (ppm)

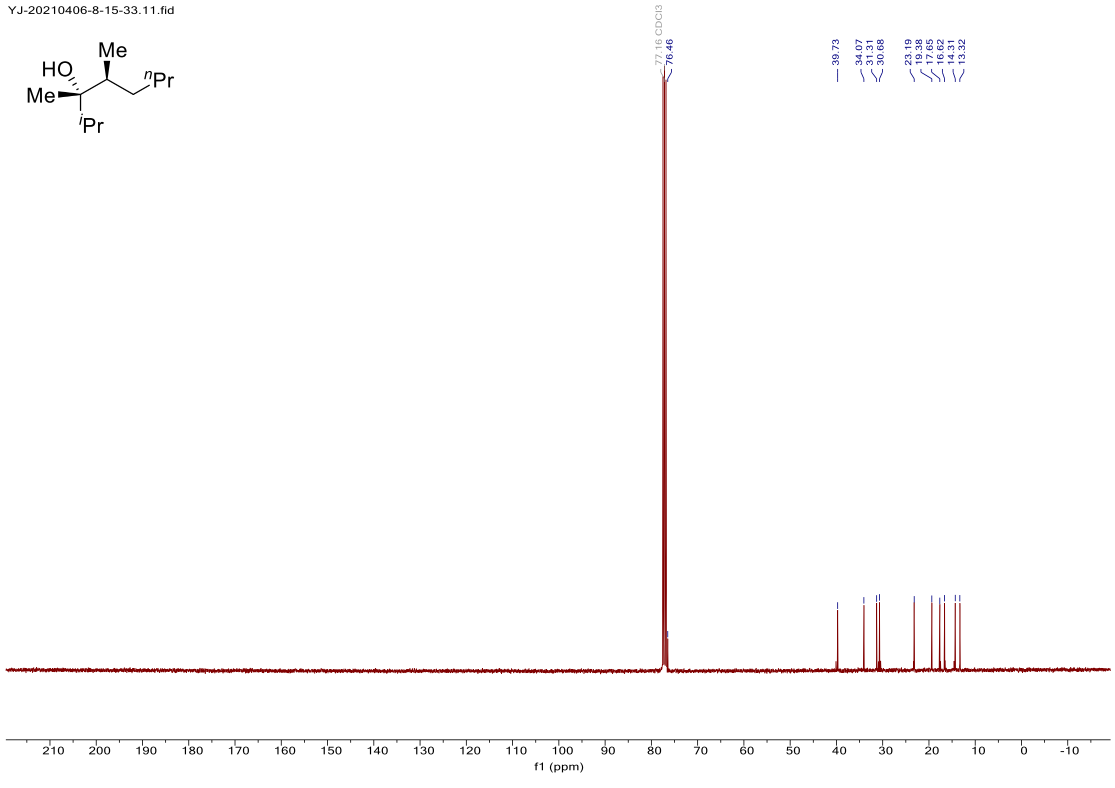
YJ-20210406-8-15-33.11.fid
77.16 CDCl3
76.46
39.73
34.07
31.31
30.68
23.19
19.38
17.65
16.62
14.31
13.32
HO
Me
Me
nPr
iPr
f1 (ppm)

YJ-20210406-8-15-34.10.fid

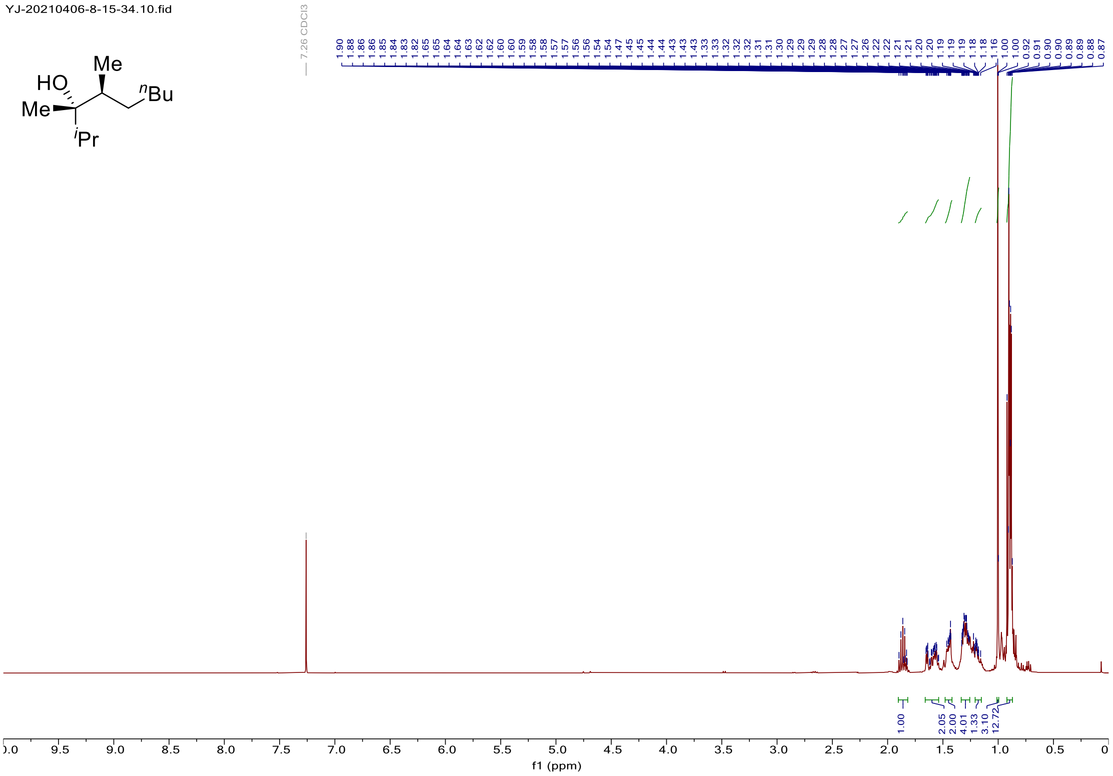

YJ-20210406-8-15-34.11.fid

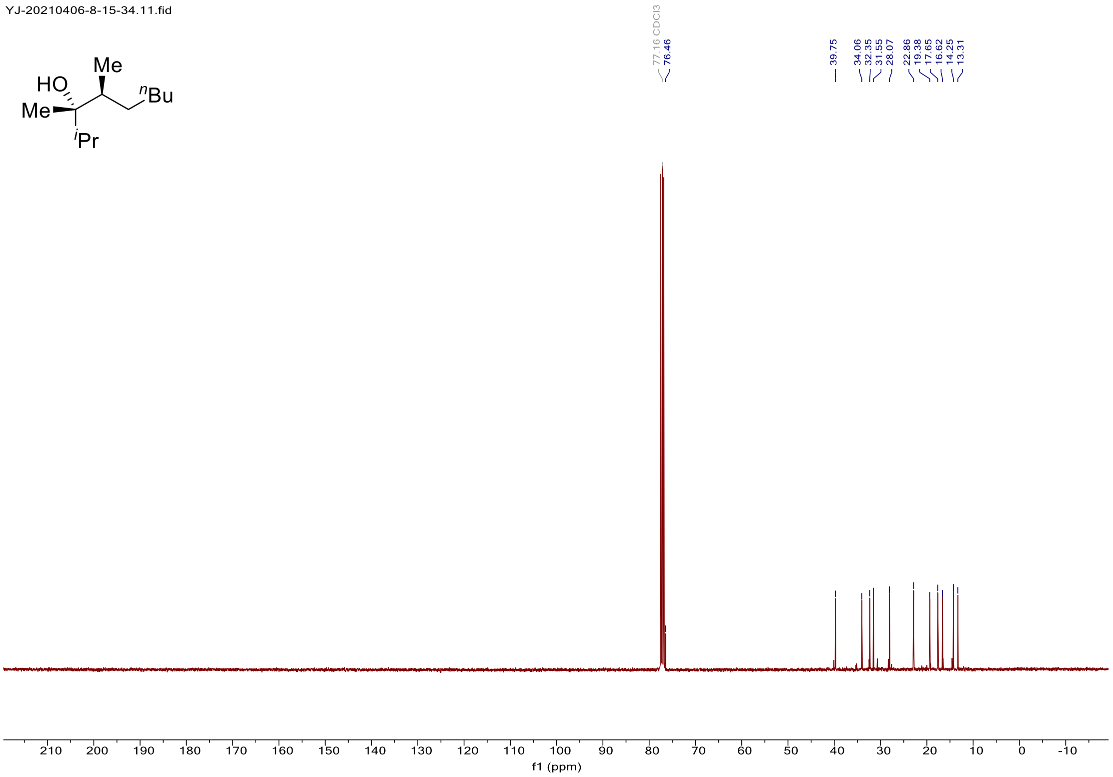

yanji-20210420-8-15-41.1.fid

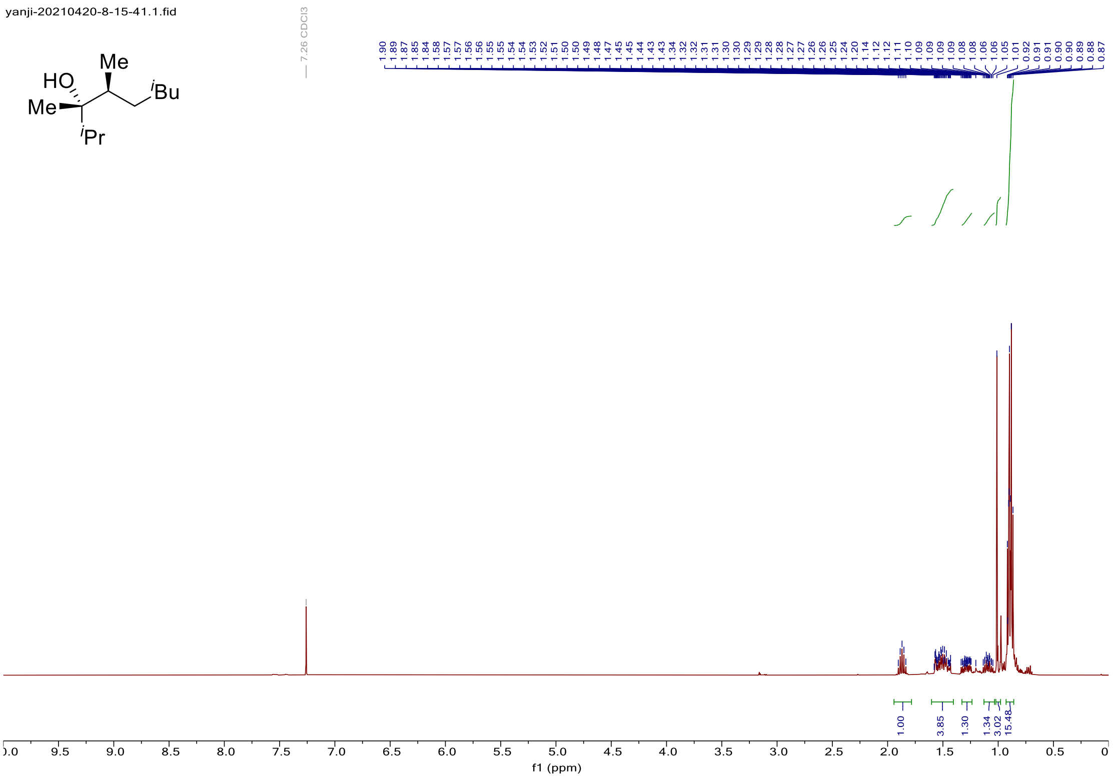


yanji-20210420-8-15-41.2.fid

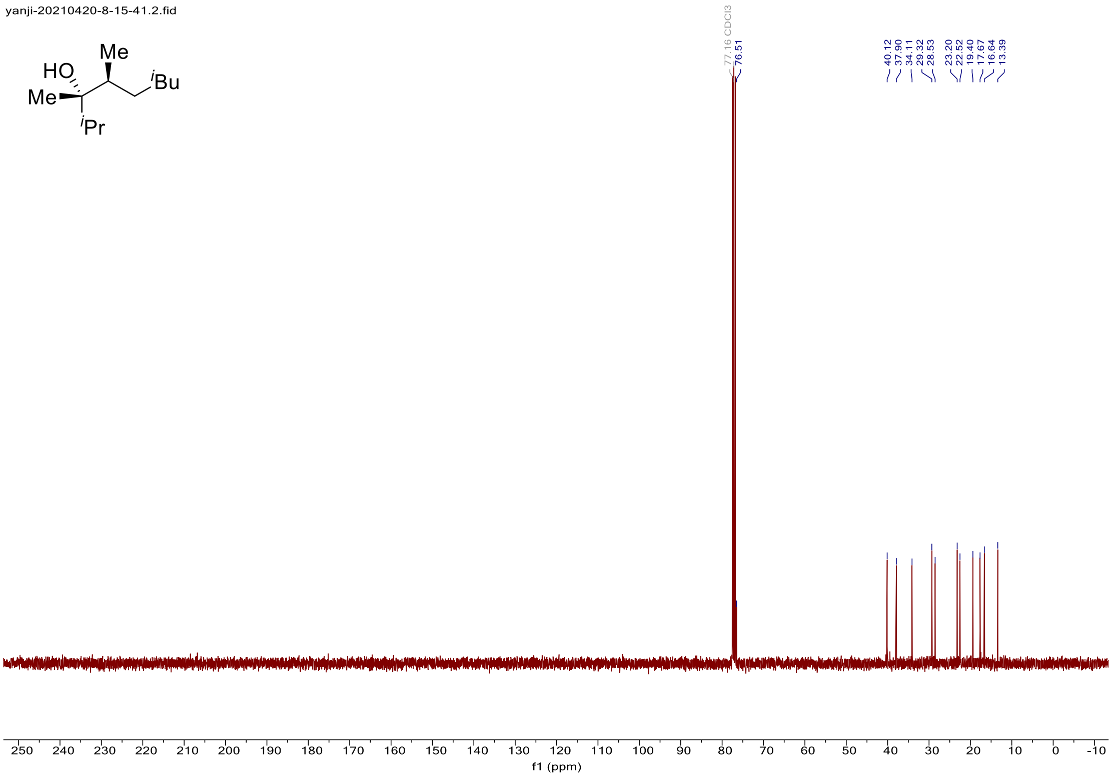

yanji-20220213-11-28-Pe-2.1.fid

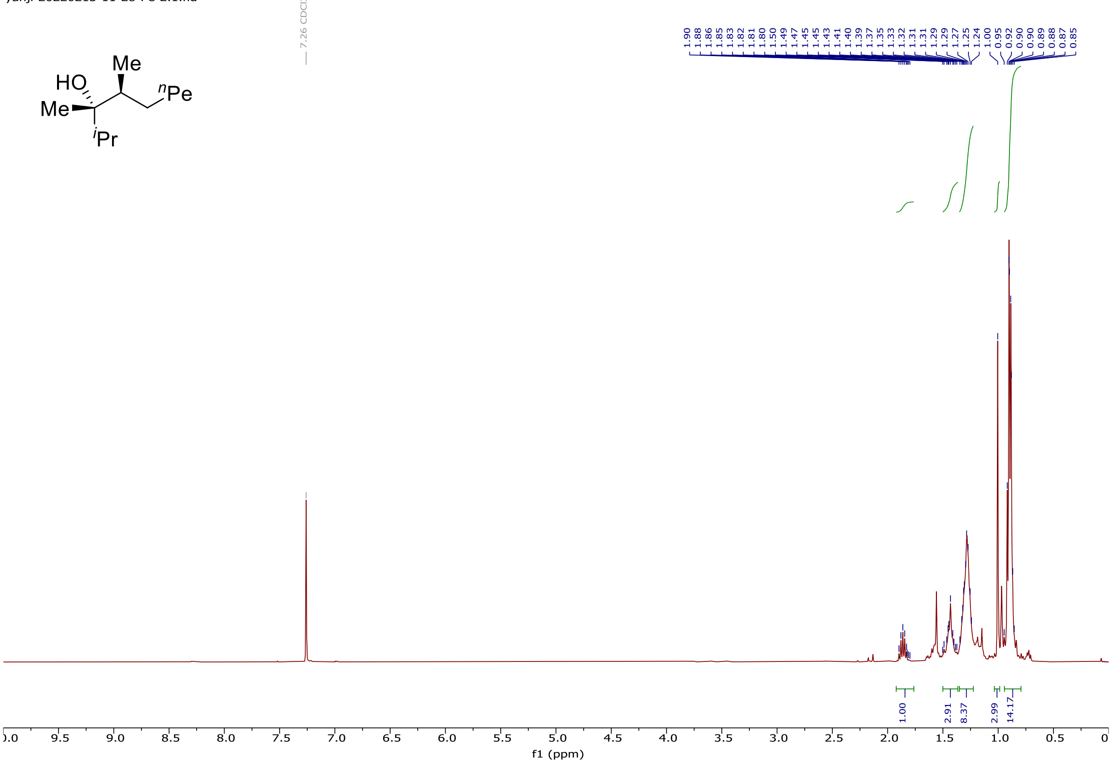


yanji-20220213-11-28-Pe-2.2.fid

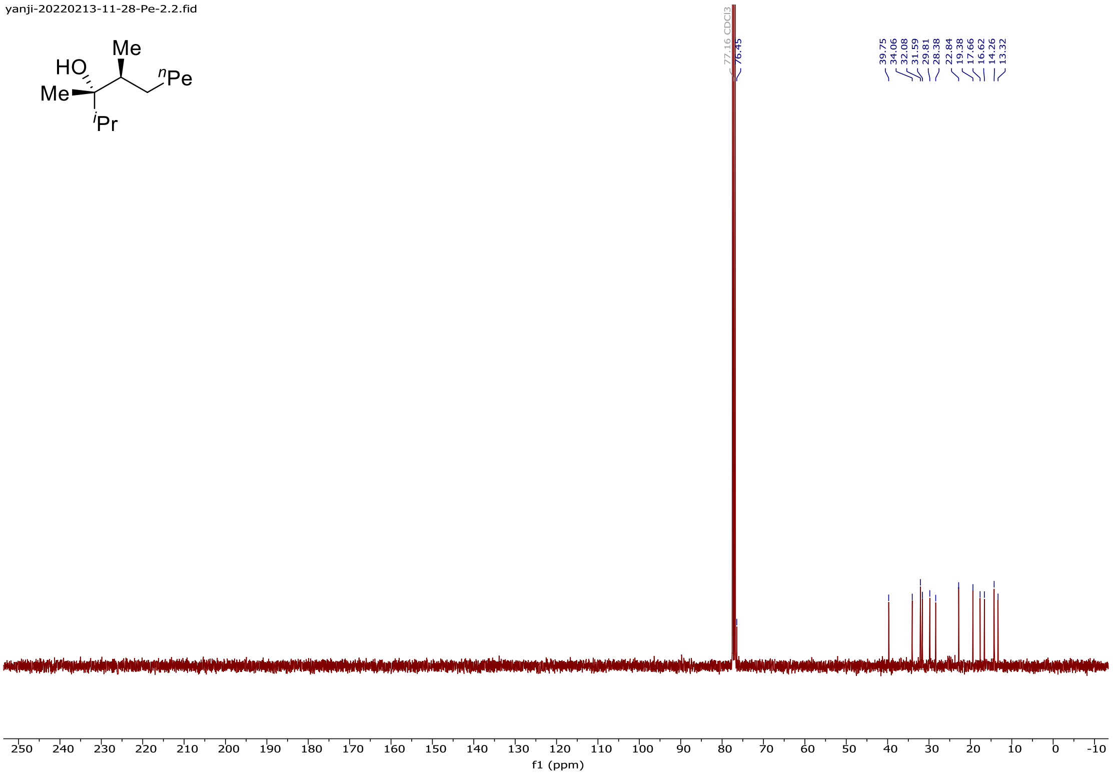

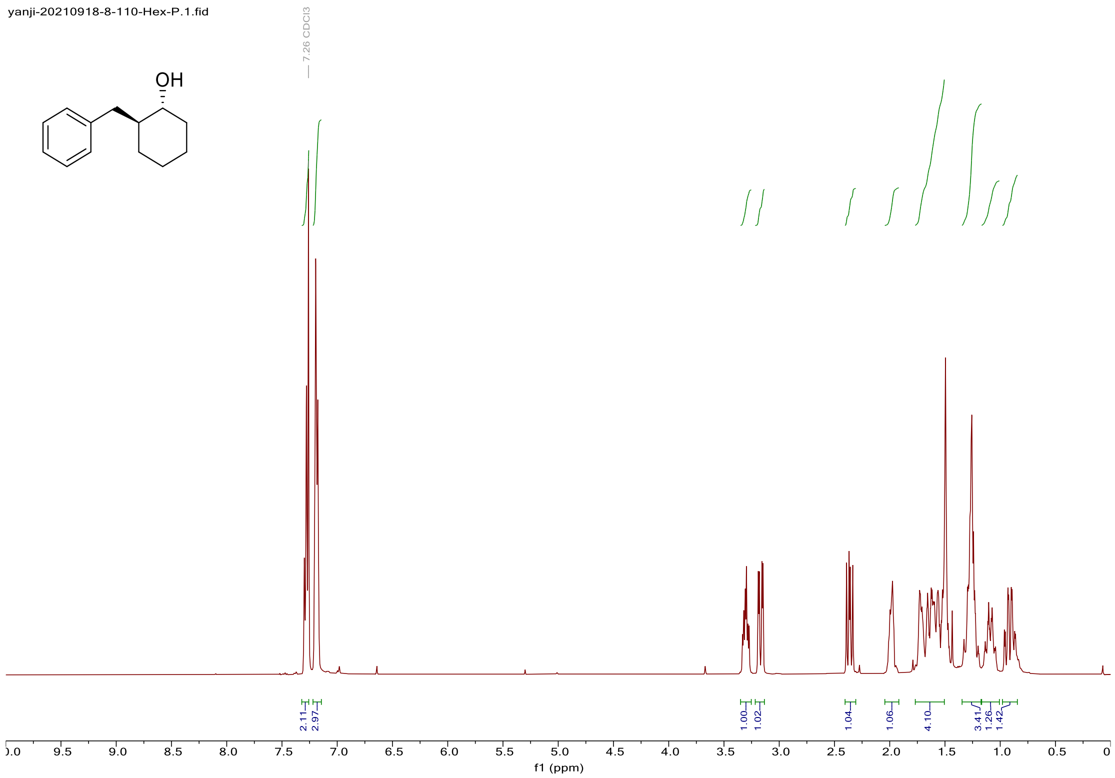
yanji-20210918-8-110-Hex-P.1.fid
OH
7.26 CDCl3
2.11
2.97
1.00
1.02
1.04
1.06
4.10
3.41
1.26
1.42
f1 (ppm)

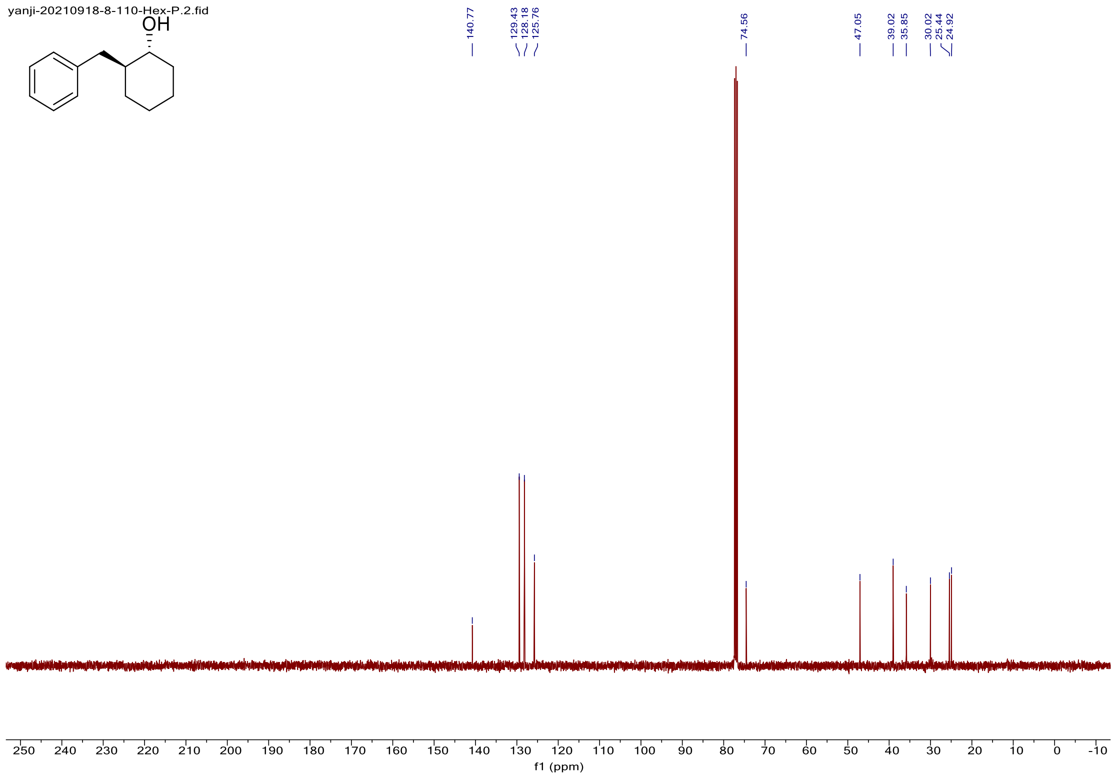
yanji-20210918-8-110-Hex-P.2.fid
OH
140.77
129.43
128.18
125.76
74.56
47.05
39.02
35.85
30.02
25.44
24.92
f1 (ppm)

yanji-20211122-10-9-Me-f2.1.fid

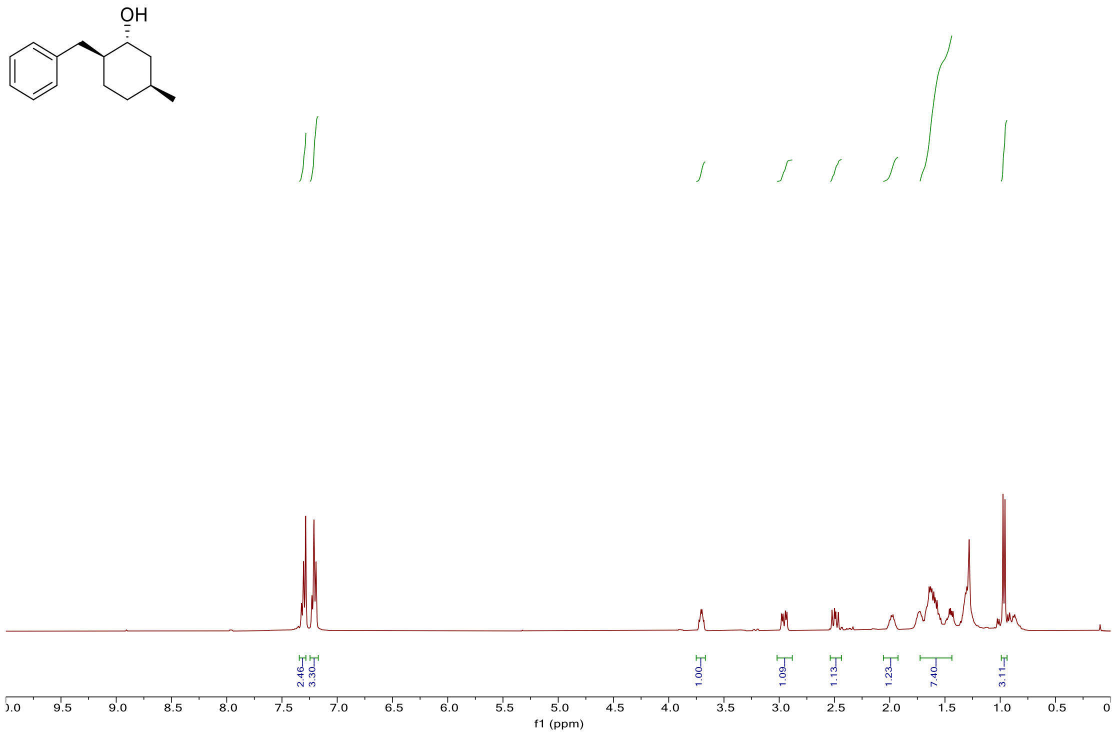


yanji-20211122-10-9-Me-f2.2.fid

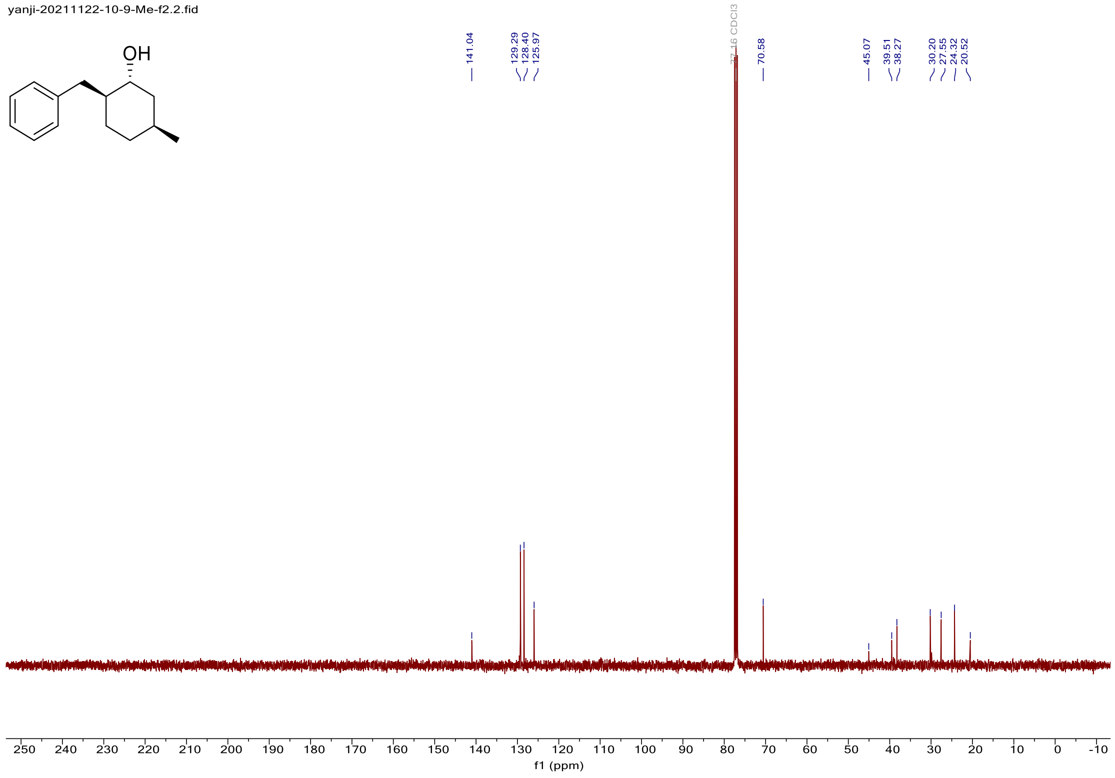

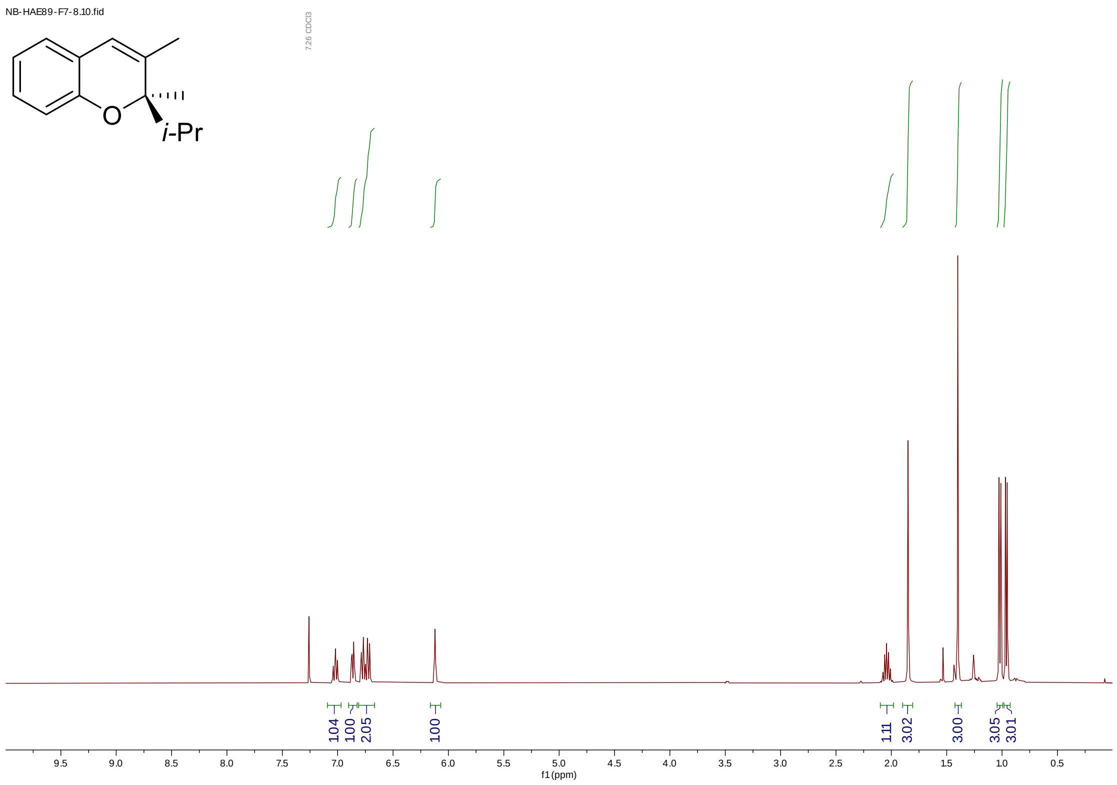
NB-HAE89-F7-8.10.fid
O
i-Pr
7.26 CDCl3
1.04
1.00
2.05
1.00
1.11
3.02
3.00
3.05
3.01
f1 (ppm)

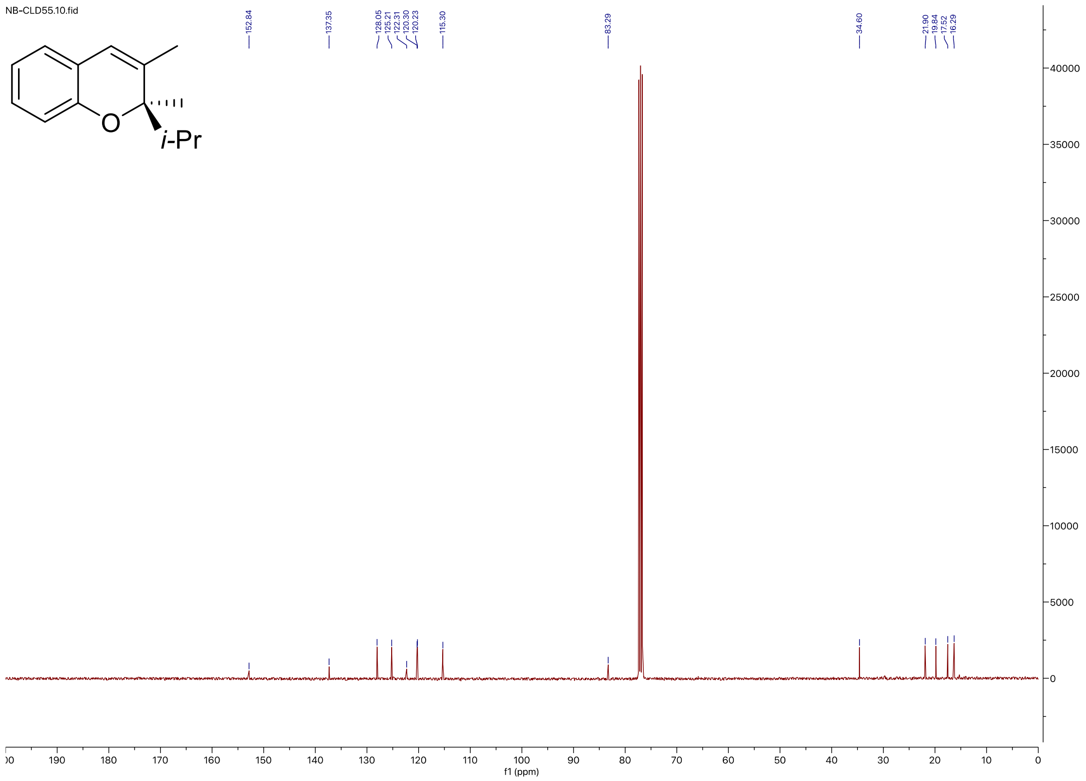
NB-CLD55.10.fid
O
i-Pr
152.84
137.35
128.05
125.21
122.31
120.30
120.23
115.30
83.29
34.60
21.90
19.84
17.52
16.29
f1 (ppm)

# 6. Chromatograms

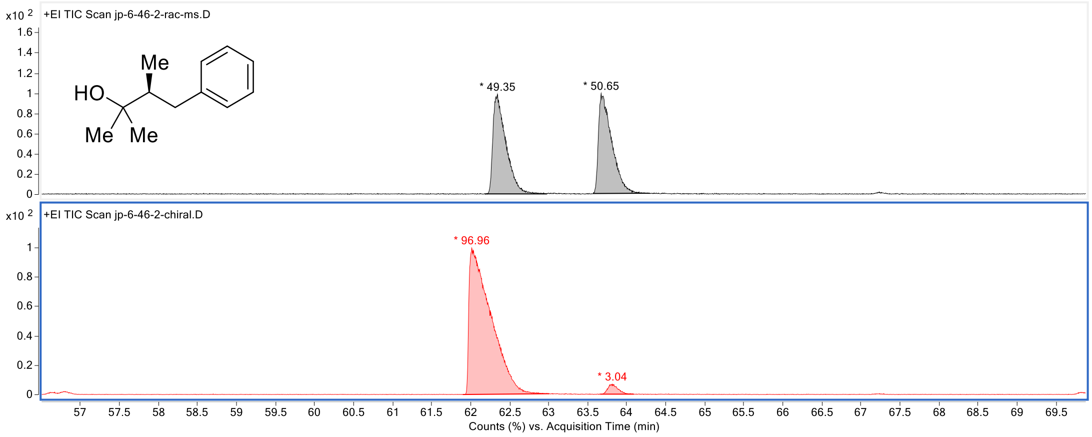


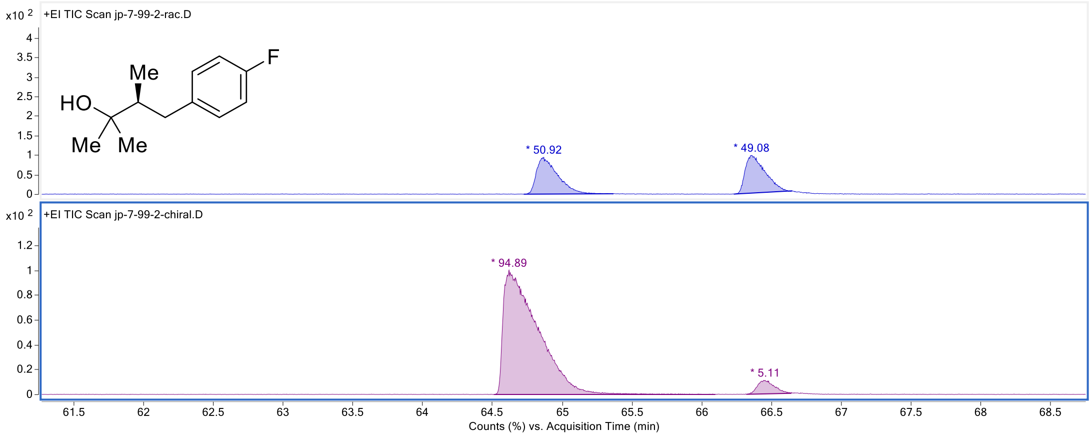


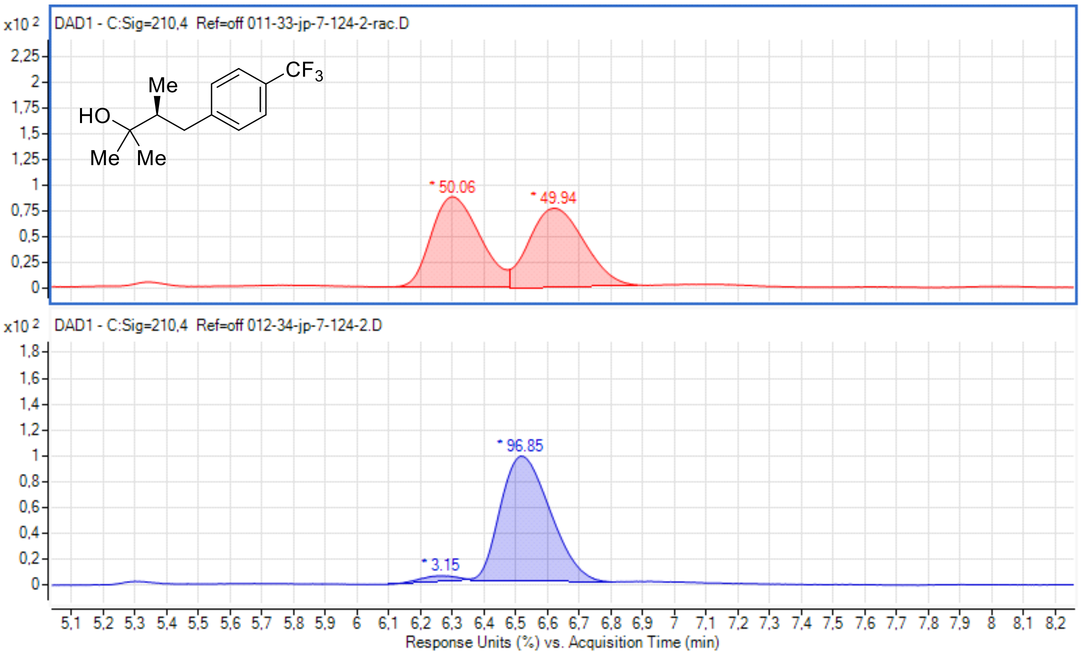

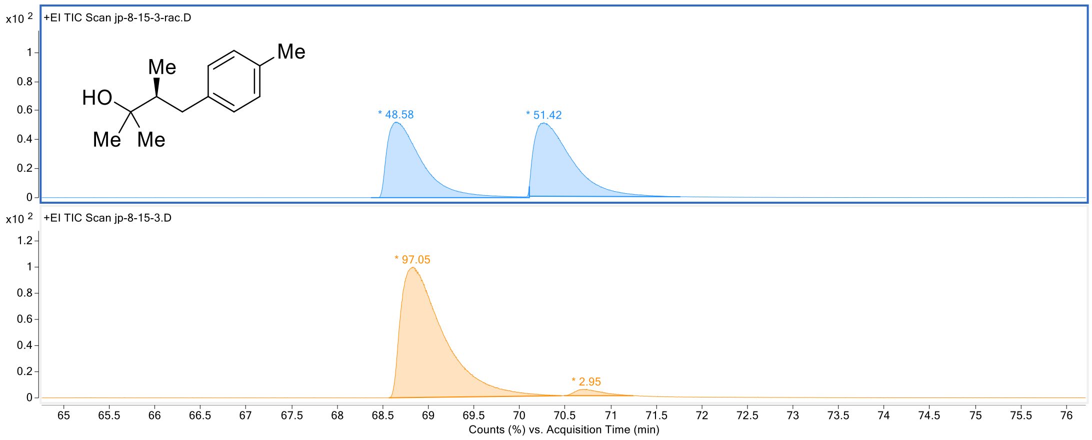
+EI TIC Scan jp-8-15-3-rac.D
HO
Me
Me Me
Me
* 48.58
* 51.42
+EI TIC Scan jp-8-15-3.D
* 97.05
* 2.95
Counts (%) vs. Acquisition Time (min)

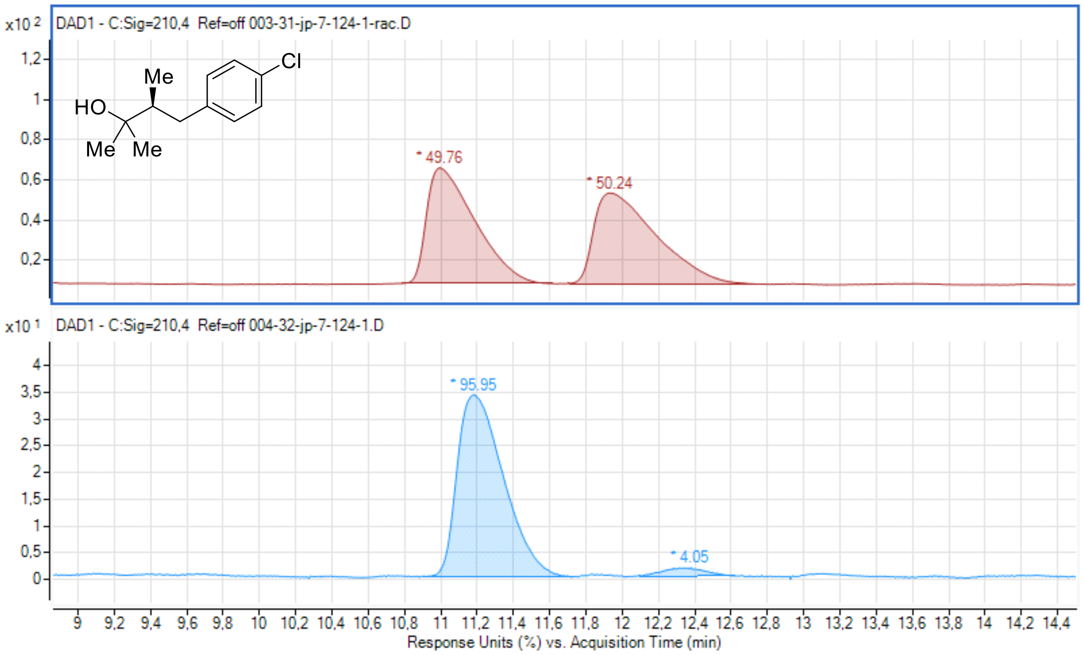
DAD1 - C:Sig=210,4 Ref=off 003-31-jp-7-124-1-rac.D
HO
Me
Me Me
Cl
* 49.76
* 50.24
DAD1 - C:Sig=210,4 Ref=off 004-32-jp-7-124-1.D
* 95.95
* 4.05
Response Units (%) vs. Acquisition Time (min)

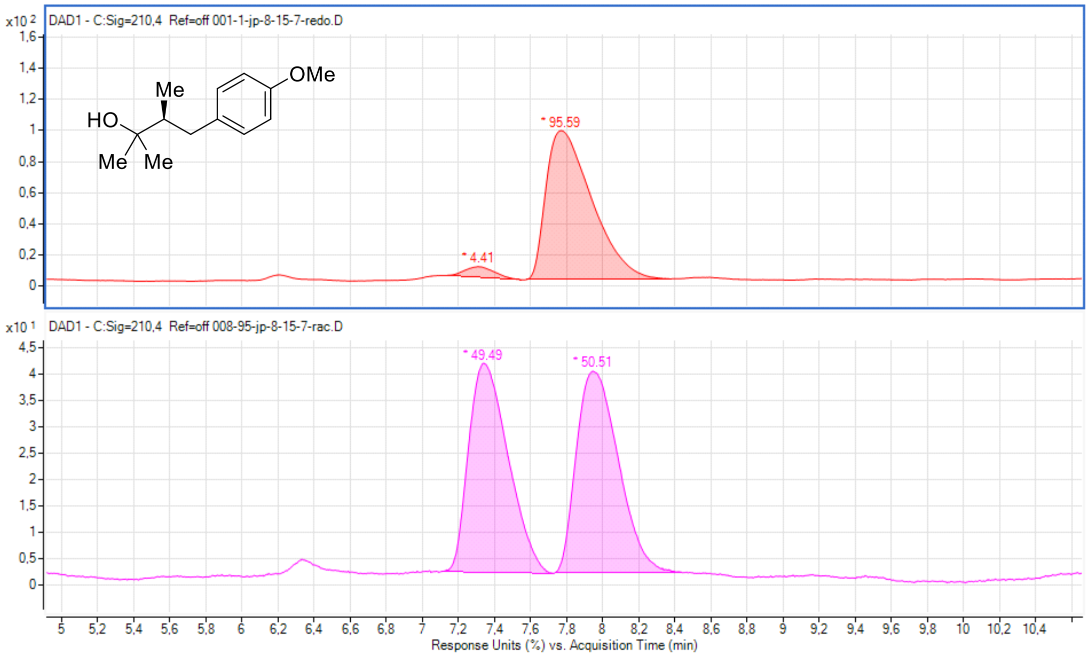
DAD1 - C:Sig=210,4 Ref=off 001-1-jp-8-15-7-redo.D
HO
Me
Me Me
OMe
* 95.59
* 4.41
DAD1 - C:Sig=210,4 Ref=off 008-95-jp-8-15-7-rac.D
* 49.49
* 50.51
Response Units (%) vs. Acquisition Time (min)

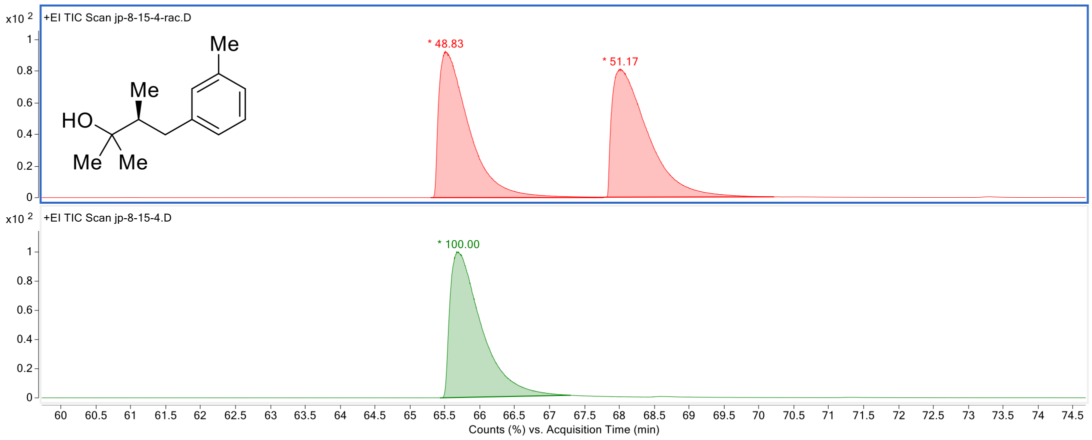
+EI TIC Scan jp-8-15-4-rac.D
* 48.83
* 51.17
+EI TIC Scan jp-8-15-4.D
* 100.00
Counts (%) vs. Acquisition Time (min)

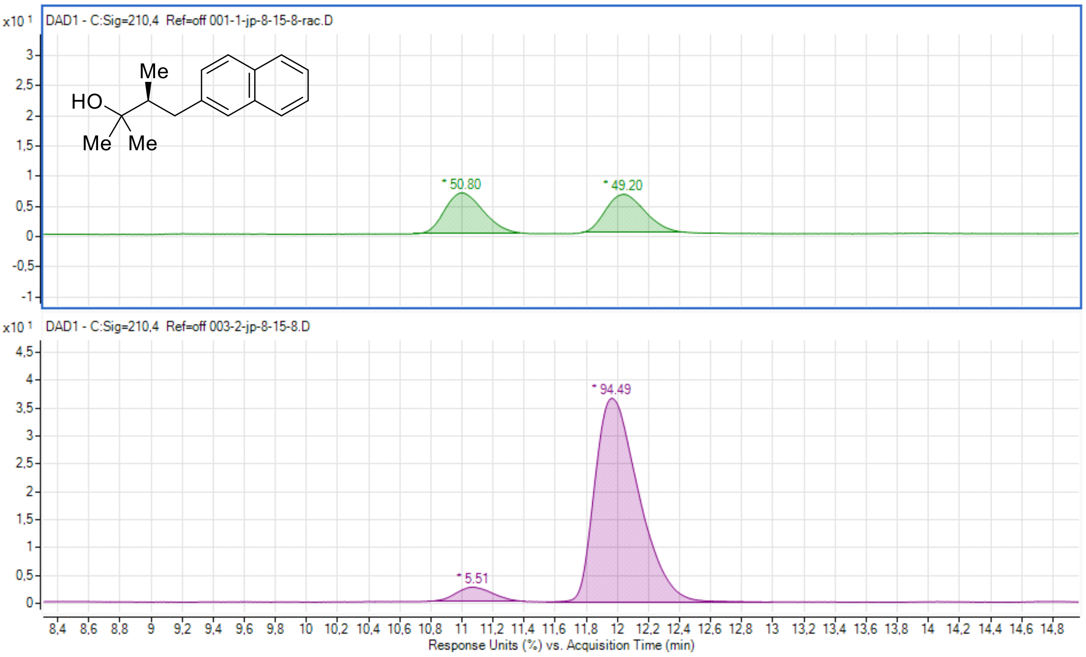
DAD1 - C:Sig=210,4 Ref=off 001-1-jp-8-15-8-rac.D
* 50.80
* 49.20
DAD1 - C:Sig=210,4 Ref=off 003-2-jp-8-15-8.D
* 94.49
* 5.51
Response Units (%) vs. Acquisition Time (min)

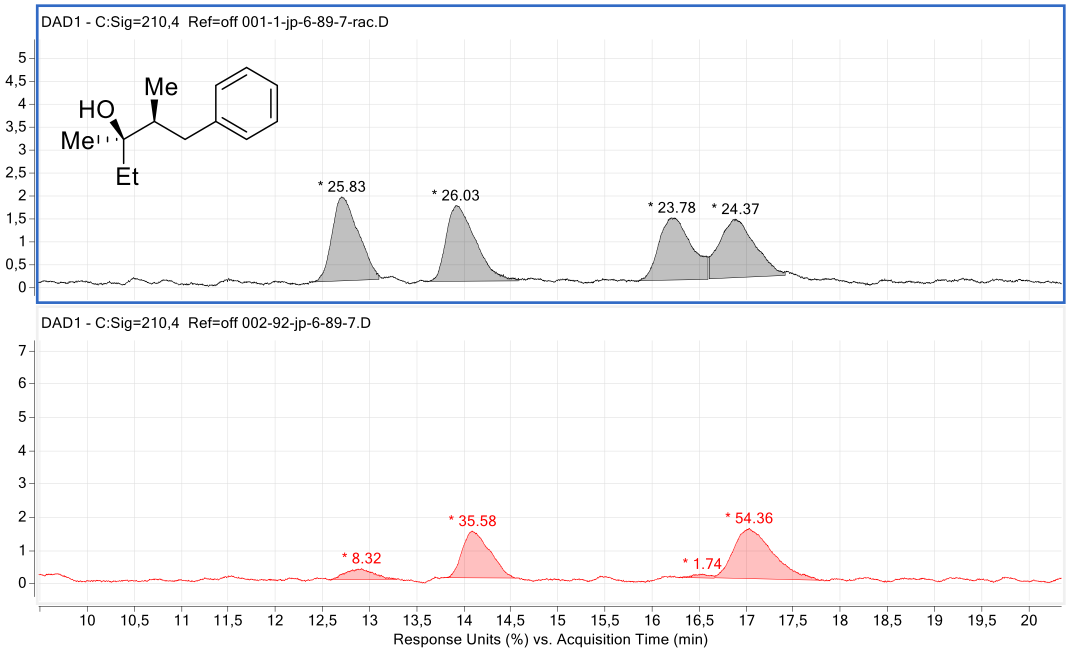
DAD1 - C:Sig=210,4 Ref=off 001-1-jp-6-89-7-rac.D
* 25.83
* 26.03
* 23.78
* 24.37
DAD1 - C:Sig=210,4 Ref=off 002-92-jp-6-89-7.D
* 8.32
* 35.58
* 1.74
* 54.36
Response Units (%) vs. Acquisition Time (min)

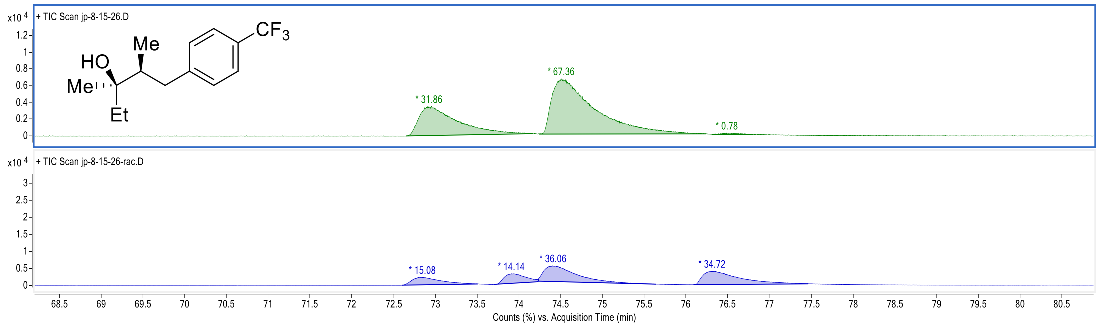
+ TIC Scan jp-8-15-26.D
HO
Me
Me
Et
CF3
* 31.86
* 67.36
* 0.78
+ TIC Scan jp-8-15-26-rac.D
* 15.08
* 14.14
* 36.06
* 34.72
Counts (%) vs. Acquisition Time (min)

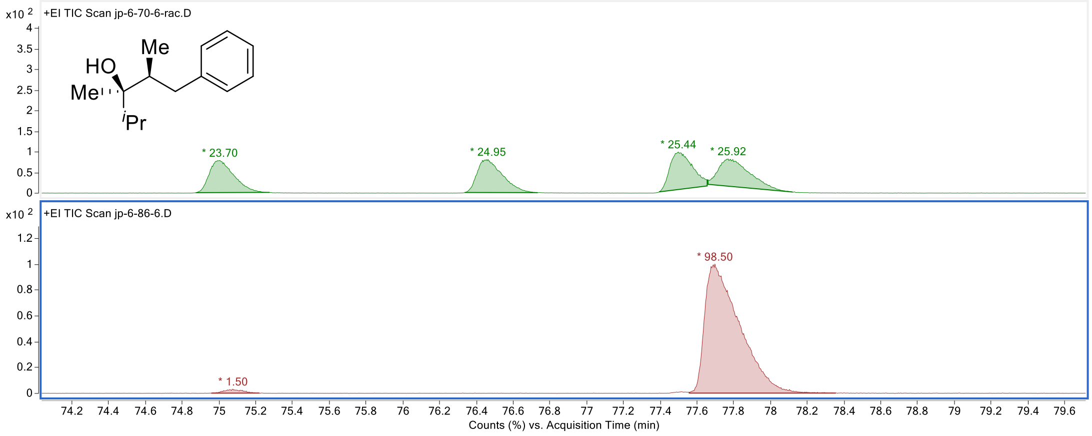
+EI TIC Scan jp-6-70-6-rac.D
HO
Me
Me
iPr
* 23.70
* 24.95
* 25.44
* 25.92
+EI TIC Scan jp-6-86-6.D
* 98.50
* 1.50
Counts (%) vs. Acquisition Time (min)

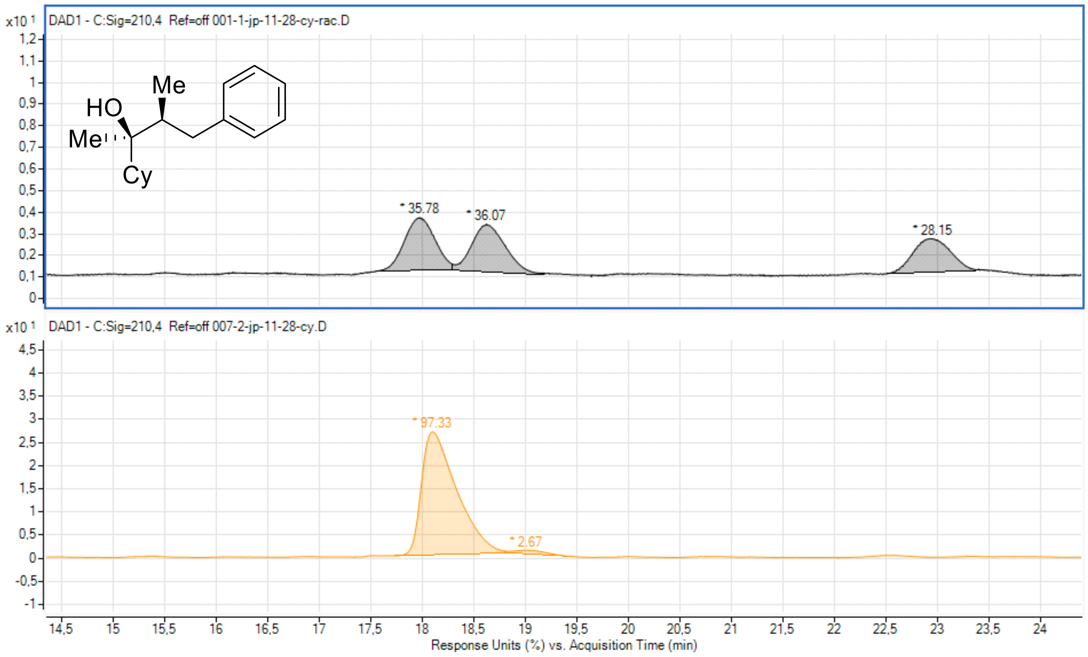
DAD1 - C:Sig=210,4 Ref=off 001-1-jp-11-28-cy-rac.D
HO
Me
Me
Cy
* 35.78
* 36.07
* 28.15
DAD1 - C:Sig=210,4 Ref=off 007-2-jp-11-28-cy.D
* 97.33
* 2.67
Response Units (%) vs. Acquisition Time (min)

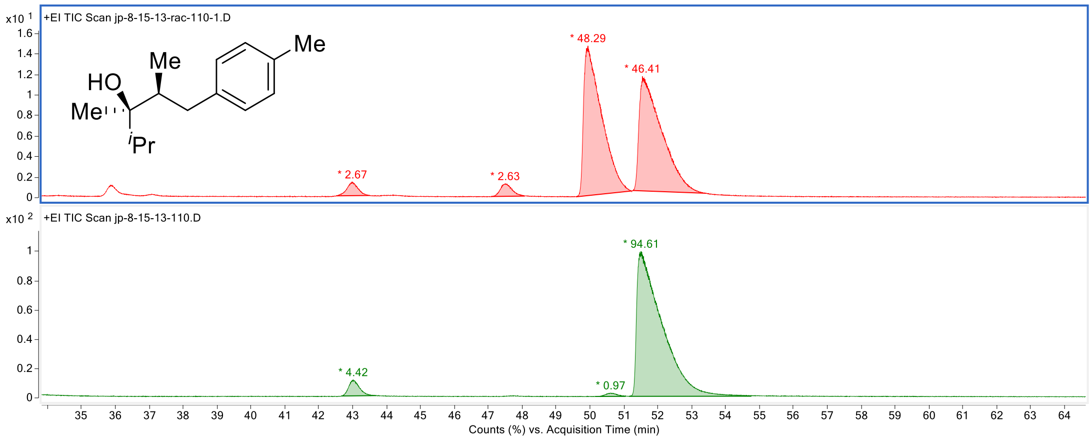
+EI TIC Scan jp-8-15-13-rac-110-1.D
HO
Me
Me
Me
iPr
* 48.29
* 46.41
* 2.67
* 2.63
+EI TIC Scan jp-8-15-13-110.D
* 94.61
* 4.42
* 0.97
Counts (%) vs. Acquisition Time (min)

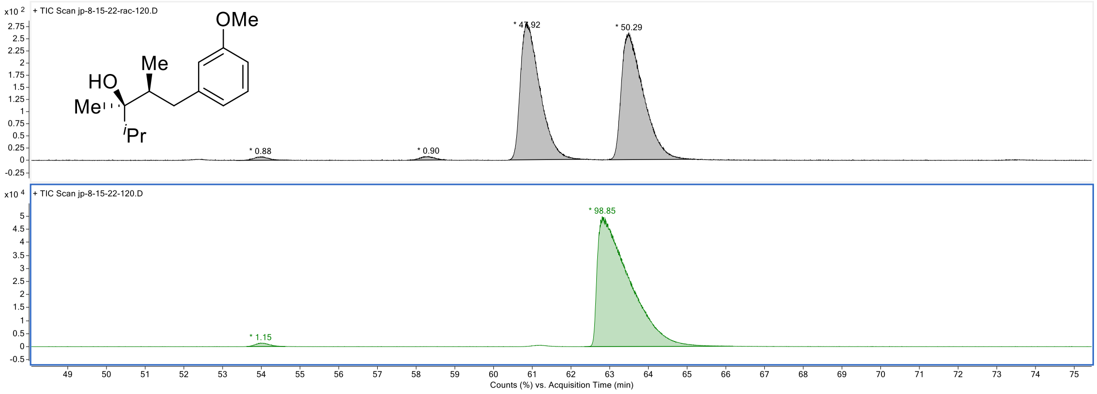
+ TIC Scan jp-8-15-22-rac-120.D
OMe
Me
HO
Me
iPr
* 0.88
* 0.90
* 50.29
+ TIC Scan jp-8-15-22-120.D
* 98.85
* 1.15
Counts (%) vs. Acquisition Time (min)

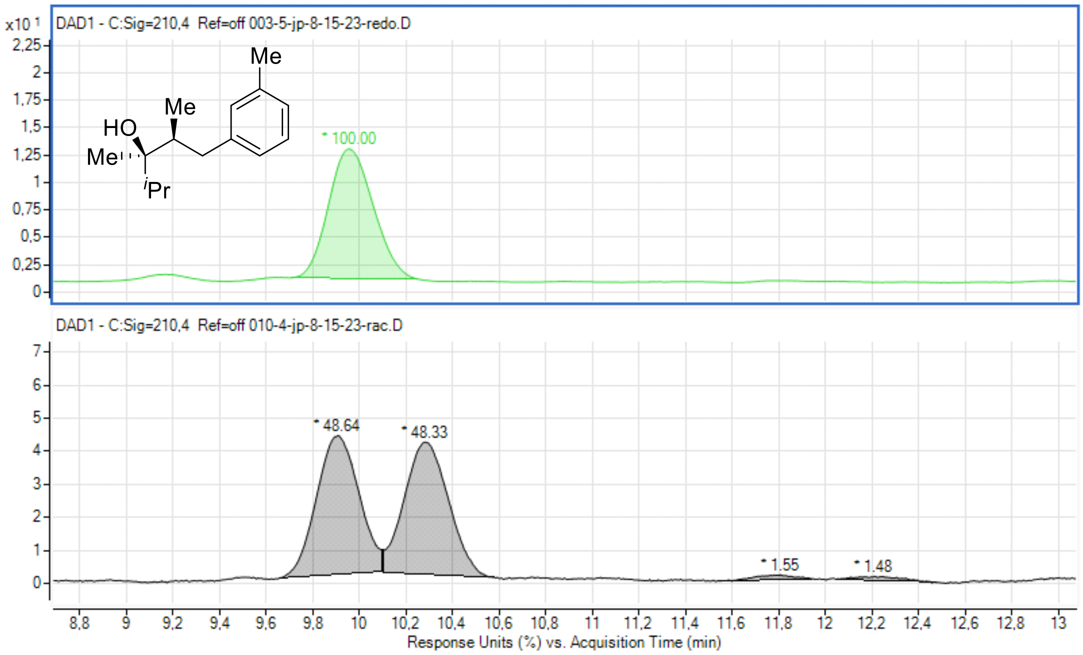
DAD1 - C:Sig=210,4 Ref=off 003-5-jp-8-15-23-redo.D
Me
Me
HO
Me
iPr
* 100.00
DAD1 - C:Sig=210,4 Ref=off 010-4-jp-8-15-23-rac.D
* 48.64
* 48.33
* 1.55
* 1.48
Response Units (%) vs. Acquisition Time (min)

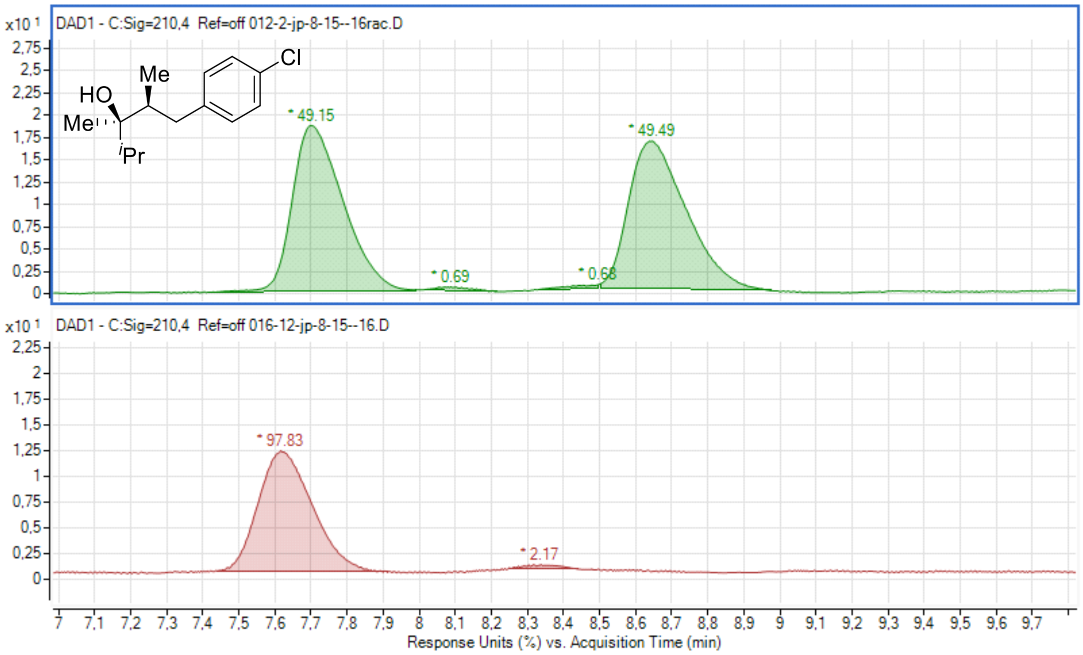
DAD1 - C:Sig=210,4 Ref=off 012-2-jp-8-15--16rac.D
49.15
49.49
0.69
0.68
DAD1 - C:Sig=210,4 Ref=off 016-12-jp-8-15--16.D
97.83
2.17
Response Units (%) vs. Acquisition Time (min)

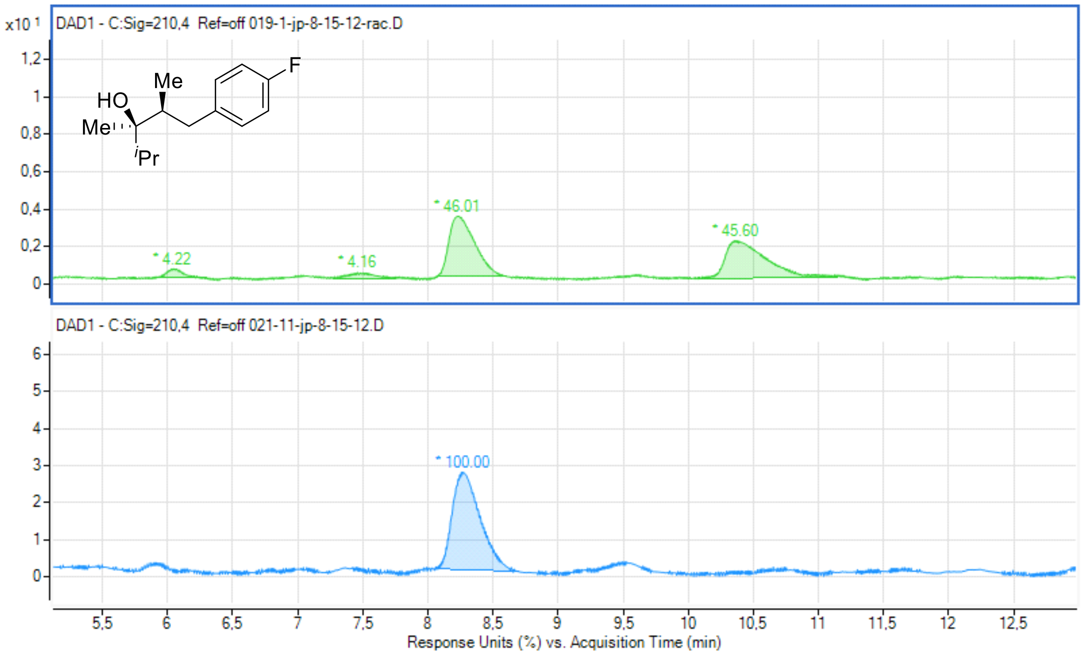
DAD1 - C:Sig=210,4 Ref=off 019-1-jp-8-15-12-rac.D
4.22
4.16
46.01
45.60
DAD1 - C:Sig=210,4 Ref=off 021-11-jp-8-15-12.D
100.00
Response Units (%) vs. Acquisition Time (min)

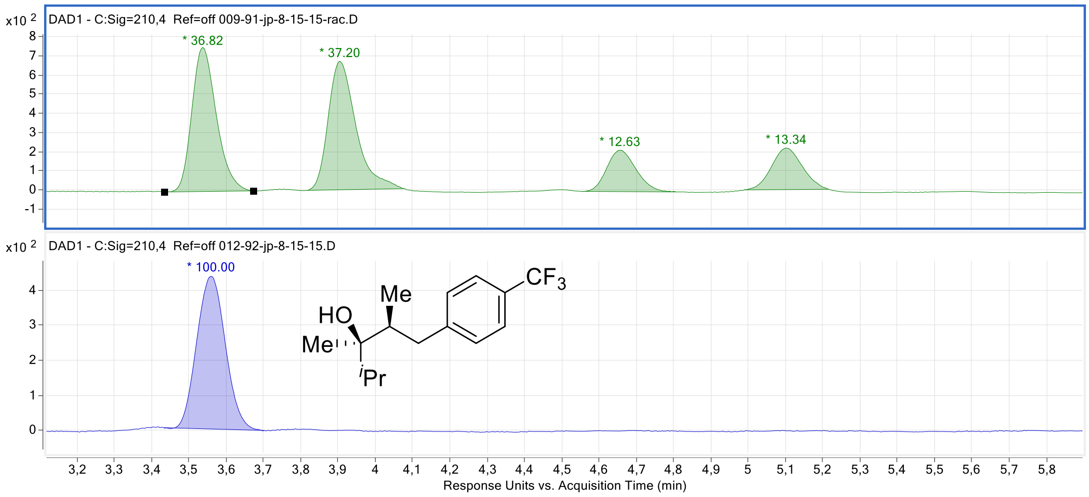
DAD1 - C:Sig=210,4 Ref=off 009-91-jp-8-15-15-rac.D
36.82
37.20
12.63
13.34
DAD1 - C:Sig=210,4 Ref=off 012-92-jp-8-15-15.D
100.00
Response Units vs. Acquisition Time (min)

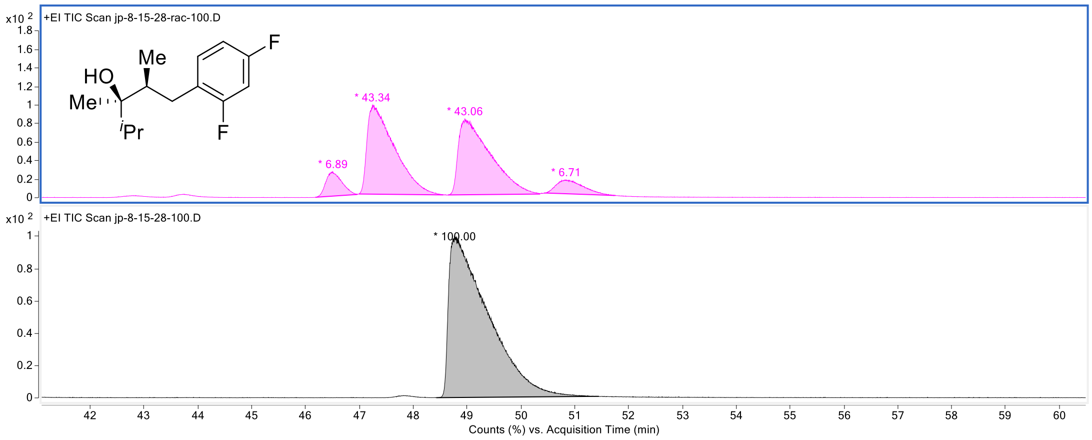
+EI TIC Scan jp-8-15-28-rac-100.D
Me
HO
Me
F
F
iPr
* 6.89
* 43.34
* 43.06
* 6.71
+EI TIC Scan jp-8-15-28-100.D
* 100.00
Counts (%) vs. Acquisition Time (min)

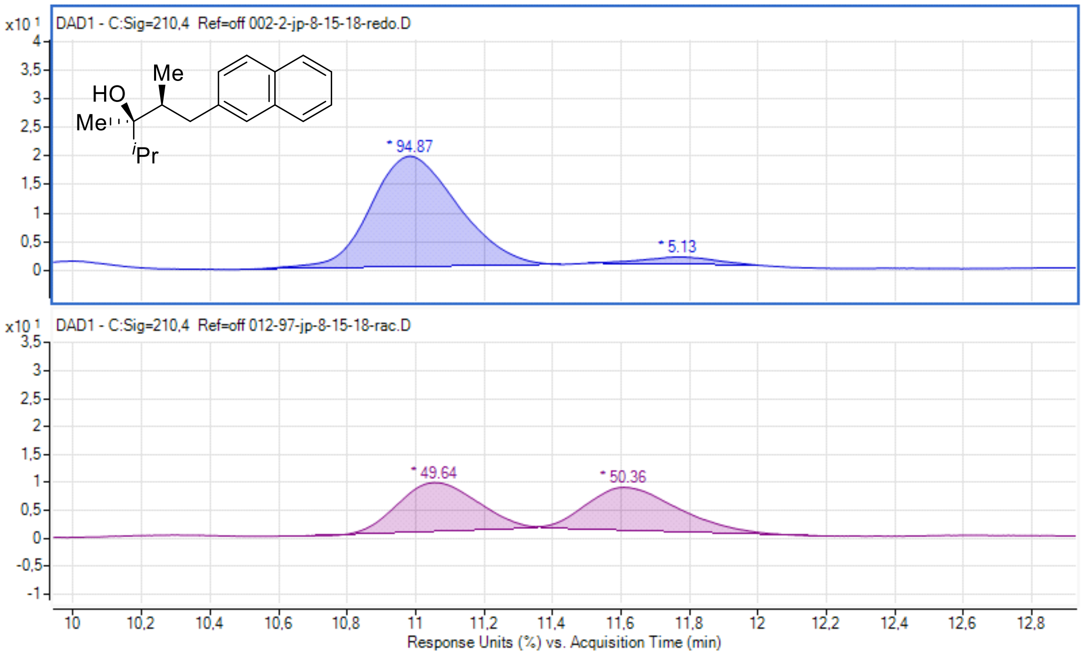
DAD1 - C:Sig=210,4 Ref=off 002-2-jp-8-15-18-redo.D
Me
HO
Me
iPr
* 94.87
* 5.13
DAD1 - C:Sig=210,4 Ref=off 012-97-jp-8-15-18-rac.D
* 49.64
* 50.36
Response Units (%) vs. Acquisition Time (min)

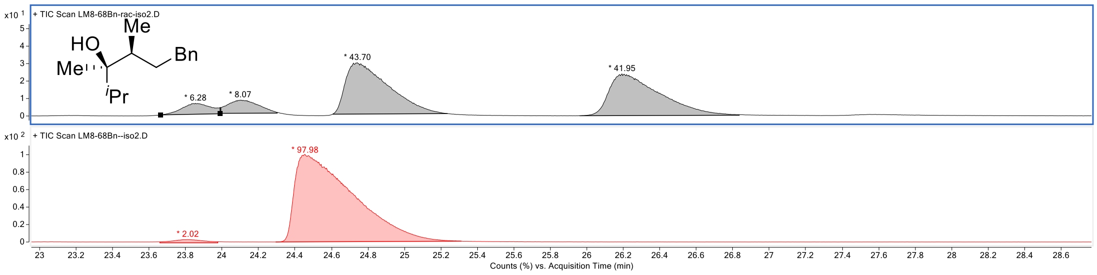
+ TIC Scan LM8-68Bn-rac-iso2.D
Me
HO
Me
Bn
iPr
* 6.28
* 8.07
* 43.70
* 41.95
+ TIC Scan LM8-68Bn--iso2.D
* 97.98
* 2.02
Counts (%) vs. Acquisition Time (min)

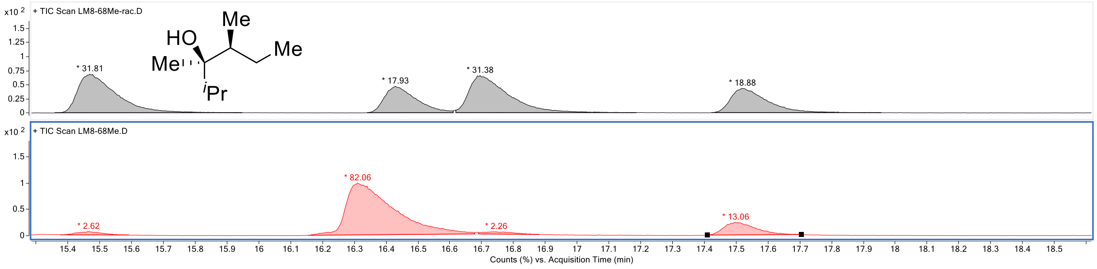
+ TIC Scan LM8-68Me-rac.D
Me
HO
Me
Me
iPr
* 31.81
* 17.93
* 31.38
* 18.88
+ TIC Scan LM8-68Me.D
* 82.06
* 2.62
* 2.26
* 13.06
Counts (%) vs. Acquisition Time (min)

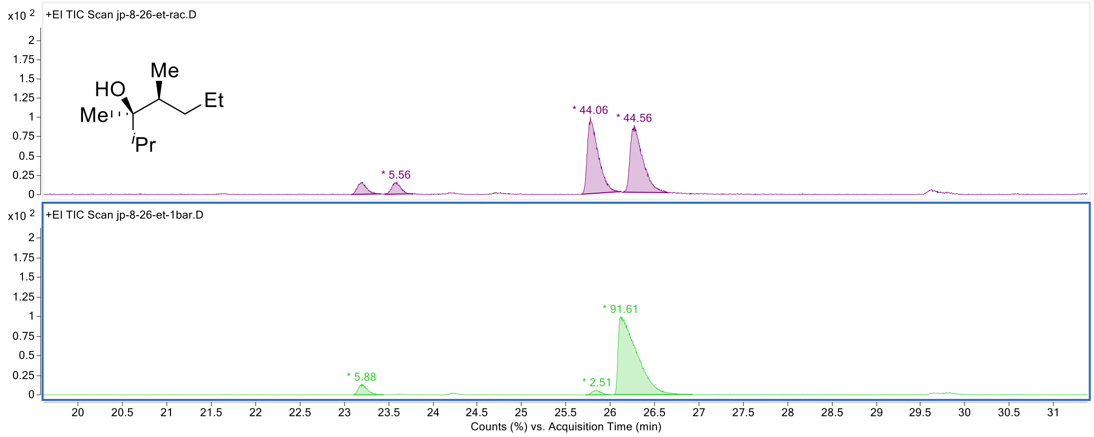
+EI TIC Scan jp-8-26-et-rac.D
x10 2
* 5.56
* 44.06
* 44.56
+EI TIC Scan jp-8-26-et-1bar.D
* 5.88
* 2.51
* 91.61
Counts (%) vs. Acquisition Time (min)
HO
Me
Et
Me
iPr

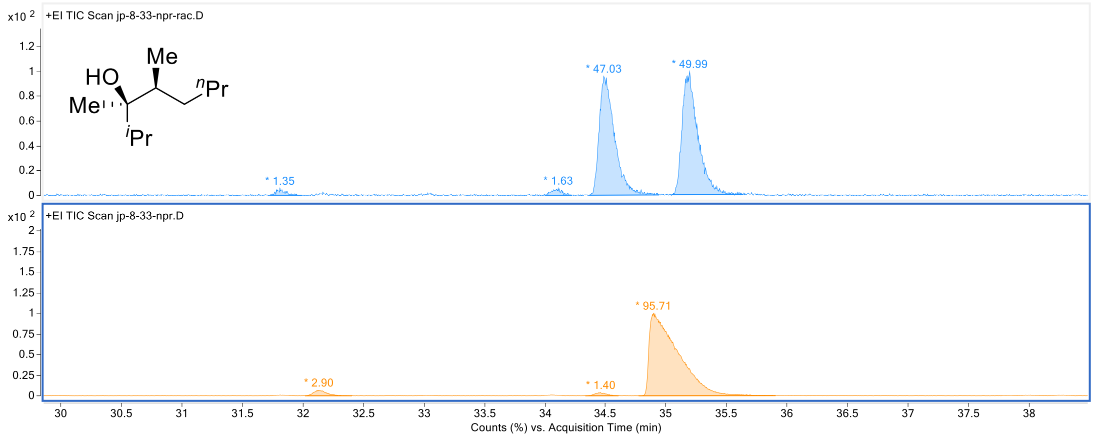
+EI TIC Scan jp-8-33-npr-rac.D
x10 2
* 1.35
* 1.63
* 47.03
* 49.99
+EI TIC Scan jp-8-33-npr.D
* 2.90
* 1.40
* 95.71
Counts (%) vs. Acquisition Time (min)
HO
Me
nPr
Me
iPr

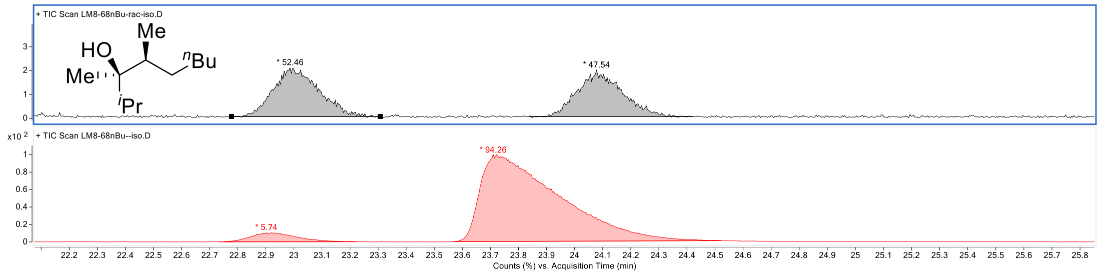
+ TIC Scan LM8-68nBu-rac-iso.D
* 52.46
* 47.54
x10 2
+ TIC Scan LM8-68nBu--iso.D
* 5.74
* 94.26
Counts (%) vs. Acquisition Time (min)
HO
Me
nBu
Me
iPr

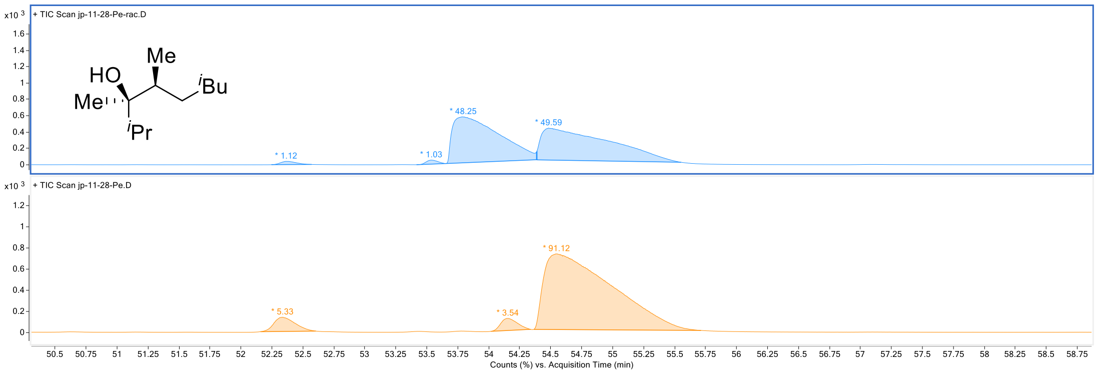
+ TIC Scan jp-11-28-Pe-rac.D
+ TIC Scan jp-11-28-Pe.D
Counts (%) vs. Acquisition Time (min)

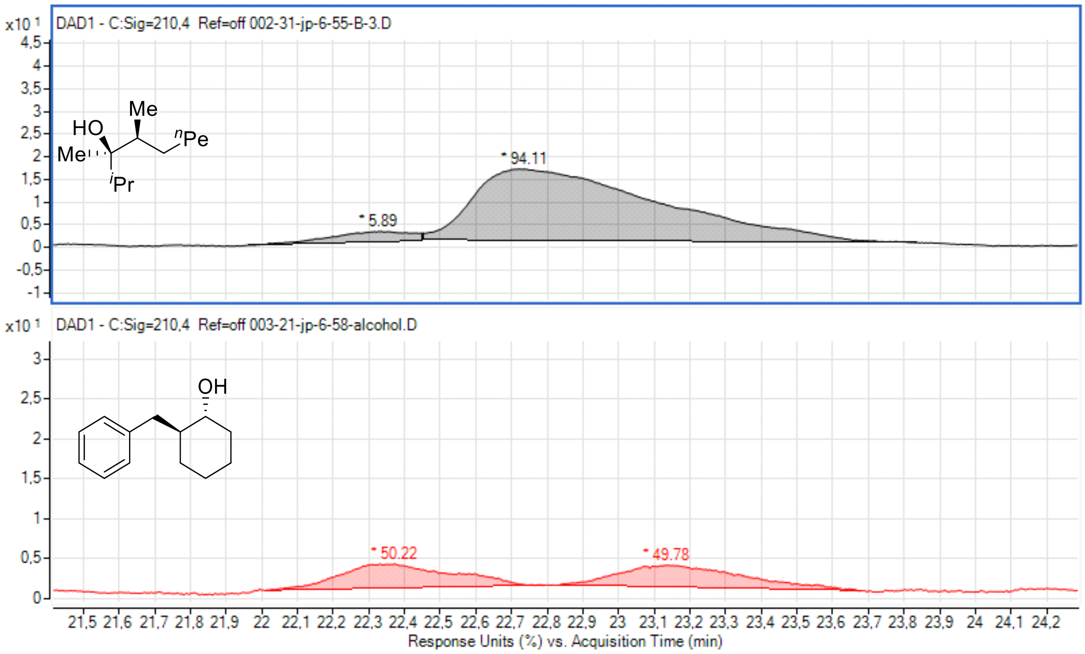
DAD1 - C:Sig=210,4 Ref=off 002-31-jp-6-55-B-3.D
DAD1 - C:Sig=210,4 Ref=off 003-21-jp-6-58-alcohol.D
Response Units (%) vs. Acquisition Time (min)

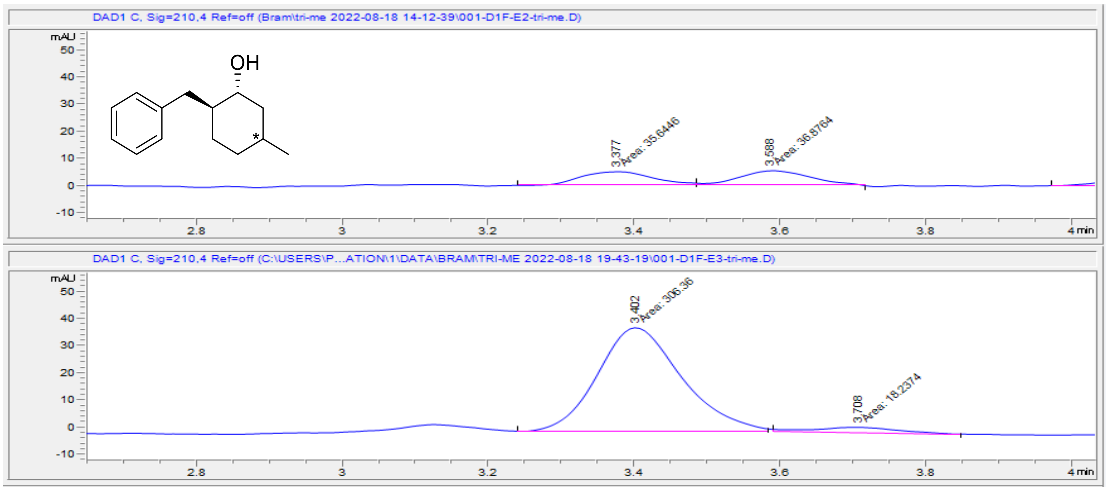
DAD1 C, Sig=210,4 Ref=off (Bram\tri-me 2022-08-18 14-12-39\001-D1F-E2-tri-me.D)
DAD1 C, Sig=210,4 Ref=off (C:\USERS\P...ATION\1\DATA\BRAM\TRI-ME 2022-08-18 19-43-19\001-D1F-E3-tri-me.D)

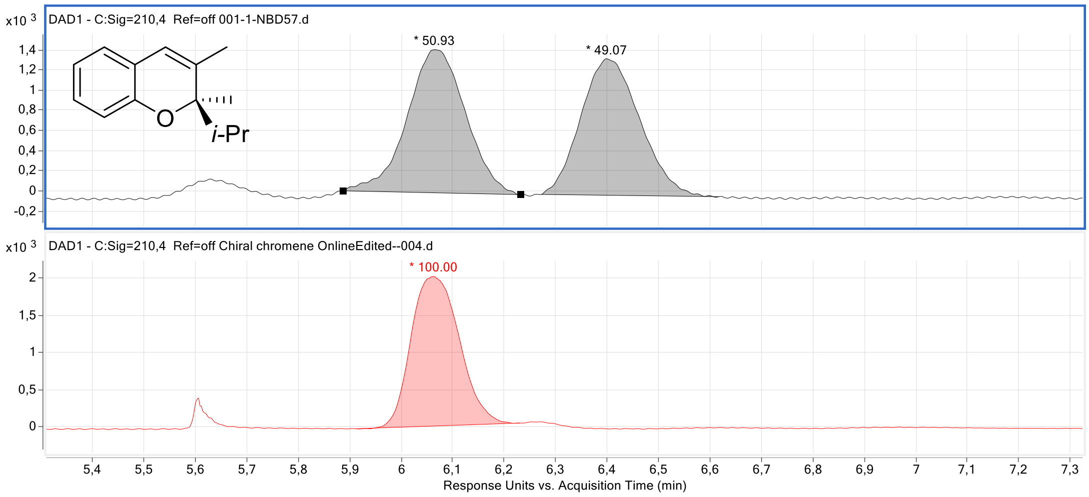


## 7. Computational Details

### XYZ coordinates of structures calculated

All calculations were performed with Gaussian 09, Revision E.01 [5] using the B3LYP/6-311+g(d) [6, 7] functional and basis set. [8] The energies shown, with the XYZ coordinates, were obtained from the optimized structures in the gas phase. All of the calculations were performed on resources provided by the Swedish National Infrastructure for Computing (SNIC) through the project SNIC 2022/22-585 and at the Center for High Performance Computing, South Africa.

**cycA_opt1 (Energy = --542.236158 Hartrees, 4.58 Kcal mol$^{-1}$)**

| | | | |
|---|---|---|---|
| C | 3.45610200 | 0.90964200 | -0.19447400 |
| C | 2.38668800 | 1.66235900 | 0.28680100 |
| C | 1.15710400 | 1.05482900 | 0.53633700 |
| C | 0.97690900 | -0.31395400 | 0.31422700 |
| C | 2.05723400 | -1.06374000 | -0.16412800 |
| C | 3.28462300 | -0.45491800 | -0.42086600 |
| H | 4.41249600 | 1.38255200 | -0.39413700 |

| | | | |
|---|---|---|---|
| H | 2.50525500 | 2.72720900 | 0.46230300 |
| H | 0.32839000 | 1.65776000 | 0.89217800 |
| H | 1.92616800 | -2.12325900 | -0.34583800 |
| H | 4.10952400 | -1.05071700 | -0.79962000 |
| C | -0.33911600 | -1.00270800 | 0.66011700 |
| H | -0.25161400 | -1.32077300 | 1.71001800 |
| C | -1.59405200 | -0.12520900 | 0.57740200 |
| C | -2.17839400 | 0.17510400 | 1.94031900 |
| H | -1.42071400 | 0.62268100 | 2.59467400 |
| H | -3.02622100 | 0.85695900 | 1.91800800 |
| H | -2.50571900 | -0.74320100 | 2.44408000 |
| O | -0.43123900 | -2.19795600 | -0.13585500 |
| H | -1.30598100 | -2.58055500 | 0.00108600 |
| C | -2.10154800 | 0.31433200 | -0.58979100 |
| C | -1.46205200 | 0.04874600 | -1.93262600 |
| H | -2.14829200 | -0.51185100 | -2.57917300 |
| H | -1.26445400 | 0.99779400 | -2.44545800 |
| H | -0.53337800 | -0.51022000 | -1.87408000 |
| C | -3.35991200 | 1.14212000 | -0.71054500 |
| H | -4.03817700 | 0.69565800 | -1.44653800 |
| H | -3.91553100 | 1.25849100 | 0.21815000 |
| H | -3.12336400 | 2.14551300 | -1.08546000 |

**cycB_opt1 (Energy = -542.243451 Hartrees, 0 Kcal mol$^{-1}$)**

| | | | |
|---|---|---|---|
| C | -4.15359200 | 0.11606300 | -0.00512200 |
| C | -3.31372200 | 1.16539700 | 0.36341600 |
| C | -1.93453800 | 0.97759600 | 0.42395400 |
| C | -1.35924500 | -0.26522100 | 0.11405300 |
| C | -2.22475400 | -1.31825800 | -0.22999100 |

| | | | |
|---|---|---|---|
| C | -3.60147300 | -1.13072700 | -0.29864300 |
| H | -5.22756700 | 0.26472100 | -0.05282700 |
| H | -3.73380800 | 2.13460000 | 0.61426400 |
| H | -1.30219000 | 1.79737900 | 0.74298500 |
| H | -1.80512500 | -2.29440500 | -0.45646100 |
| H | -4.24499400 | -1.95925100 | -0.57783800 |
| C | 0.09045300 | -0.53641000 | 0.18359300 |
| C | 1.11556200 | 0.26290300 | -0.15685700 |
| C | 0.94569800 | 1.63692000 | -0.76059300 |
| H | 1.00915000 | 2.43123000 | -0.00745500 |
| H | 1.73289200 | 1.82181800 | -1.49181600 |
| H | -0.01864900 | 1.73849900 | -1.25885100 |
| C | 2.56995600 | -0.18126500 | 0.04504900 |
| C | 2.74383200 | -1.64943500 | 0.44840300 |
| H | 2.29693900 | -2.31470600 | -0.29224700 |
| H | 3.81114700 | -1.88587000 | 0.50793700 |
| H | 2.31256800 | -1.86648600 | 1.42856800 |
| C | 3.24824600 | 0.72601800 | 1.09072600 |
| H | 4.29837900 | 0.44092400 | 1.22135300 |
| H | 3.22196000 | 1.77175600 | 0.78057400 |
| H | 2.75803800 | 0.64060000 | 2.06405700 |
| H | 0.31905800 | -1.53907900 | 0.53160500 |
| O | 3.20731300 | 0.01684700 | -1.23998800 |
| H | 4.15515900 | -0.13799500 | -1.14187300 |

**cycC_cont_opt1 (Energy = -542.235357 Hartrees, 5.08 Kcal mol$^{-1}$)**

| | | | |
|---|---|---|---|
| C | 0.14905400 | 1.21460000 | -0.40411600 |
| C | 1.42963300 | 0.82549700 | -0.26754400 |
| C | 2.51778400 | 1.81790300 | -0.62685400 |
| H | 3.06888400 | 2.16183100 | 0.25431000 |

| | | | |
|---|---|---|---|
| H | 3.25791900 | 1.38885700 | -1.31083600 |
| H | 2.09663600 | 2.69729200 | -1.11583900 |
| C | 1.93251200 | -0.52747000 | 0.23881100 |
| C | 3.06232900 | -0.34979100 | 1.27210200 |
| H | 3.34049300 | -1.32747200 | 1.67970900 |
| H | 3.96445100 | 0.08821600 | 0.84077200 |
| H | 2.72754900 | 0.27778600 | 2.10077500 |
| O | 0.83920000 | -1.18726400 | 0.88822300 |
| H | 1.13999300 | -2.05033600 | 1.19866000 |
| C | 2.42932800 | -1.39316500 | -0.93731800 |
| H | 2.79918700 | -2.35708600 | -0.56946500 |
| H | 1.61897600 | -1.58411500 | -1.64276100 |
| H | 3.24967100 | -0.91674600 | -1.47914900 |
| C | -1.15813300 | 0.54751300 | -0.20350100 |
| C | -2.16867500 | 1.25829600 | 0.46215200 |
| C | -1.48289000 | -0.70723300 | -0.73640200 |
| C | -3.44209900 | 0.72011800 | 0.63019400 |
| H | -1.94665600 | 2.24286200 | 0.86447900 |
| C | -2.75902000 | -1.24137200 | -0.58524300 |
| H | -0.73081400 | -1.26646000 | -1.27766200 |
| C | -3.74344000 | -0.53558000 | 0.10635100 |
| H | -4.20001000 | 1.28523500 | 1.16406200 |
| H | -2.98732100 | -2.21356300 | -1.01186200 |
| H | -4.73716200 | -0.95569300 | 0.22600600 |
| H | 0.02631700 | 2.25160400 | -0.71877000 |

**Aprime_opt1 (Energy = -350.45983Hartrees, 2.53 Kcal mol$^{-1}$)**

| | | | |
|---|---|---|---|
| C | -1.98145700 | 0.03193400 | 1.16951700 |
| C | -1.34763500 | 0.27435400 | -0.20667500 |
| C | 0.14155800 | 0.59972900 | -0.12050200 |

| | | | |
|---|---|---|---|
| C | 0.37941800 | 2.09193500 | -0.20257600 |
| H | -0.28566000 | 2.62886800 | 0.48474300 |
| H | 1.39594600 | 2.39218600 | 0.04300600 |
| H | 0.15187700 | 2.47082400 | -1.20684800 |
| C | 1.11410200 | -0.31572900 | 0.03796300 |
| C | 0.87927400 | -1.80312900 | 0.17148000 |
| H | 1.37479300 | -2.34022200 | -0.64620600 |
| H | 1.33296400 | -2.17173200 | 1.10024500 |
| H | -0.16789900 | -2.08508000 | 0.15105900 |
| C | 2.58680600 | 0.02311200 | 0.10269400 |
| H | 3.14327000 | -0.58088800 | -0.62326100 |
| H | 2.82123100 | 1.06691900 | -0.09714700 |
| H | 2.99882100 | -0.22893600 | 1.08780500 |
| H | -1.82153500 | 0.89139300 | 1.82705100 |
| H | -3.06426000 | -0.11695400 | 1.08315500 |
| H | -1.55027500 | -0.84815500 | 1.65022300 |
| O | -1.57479200 | -0.83491000 | -1.09251500 |
| H | -2.52716600 | -0.96738600 | -1.17856600 |
| H | -1.83617100 | 1.15519900 | -0.64654500 |

**Bprime_opt1 (Energy = -350.463863 Hartrees, 0 Kcal mol$^{-1}$)**

| | | | |
|---|---|---|---|
| C | 2.95050500 | -0.46544400 | -0.06344000 |
| C | 1.46510700 | -0.68637300 | -0.02050700 |
| C | 0.49962700 | 0.23846100 | 0.03940900 |
| C | 0.77546500 | 1.72510400 | 0.09537500 |
| H | 0.72981700 | 2.18401100 | -0.89936000 |
| H | 0.03552400 | 2.22556200 | 0.72035900 |
| H | 1.76110900 | 1.94434700 | 0.50584100 |
| C | -0.98526500 | -0.14249000 | 0.01707800 |
| C | -1.26022800 | -1.64418300 | 0.14950500 |

| | | | |
|---|---|---|---|
| H | -0.82009400 | -2.04298600 | 1.06489900 |
| H | -2.34088400 | -1.81467800 | 0.19562800 |
| H | -0.88239600 | -2.21303200 | -0.70351500 |
| C | -1.64969000 | 0.39396200 | -1.26570300 |
| H | -2.71937900 | 0.15510100 | -1.27341600 |
| H | -1.54964600 | 1.47807000 | -1.33758300 |
| H | -1.20324000 | -0.05454900 | -2.15715300 |
| H | 1.17974600 | -1.73421200 | -0.04995300 |
| O | -1.56278800 | 0.52875500 | 1.16472300 |
| H | -2.52072000 | 0.41057700 | 1.14124400 |
| H | 3.44237600 | -0.95904300 | 0.78289900 |
| H | 3.37989900 | -0.90949900 | -0.96924000 |
| H | 3.23705500 | 0.58606300 | -0.04873000 |

**Cprime_cont_opt1 (Energy = -350.460203 Hartrees) 2.30 Kcal mol$^{-1}$)**

| | | | |
|---|---|---|---|
| C | 1.66394700 | 0.56871700 | -0.03540000 |
| C | 0.33518100 | 0.74775000 | -0.02859200 |
| C | -0.21060700 | 2.15943000 | 0.04286600 |
| H | -0.76962700 | 2.43570700 | -0.85683200 |
| H | -0.89404600 | 2.29517600 | 0.88947700 |
| H | 0.59908000 | 2.88122700 | 0.16182200 |
| C | -0.71860600 | -0.36680900 | -0.01386600 |
| C | -1.99834400 | 0.03232400 | -0.76901300 |
| H | -2.69181600 | -0.81557500 | -0.78709300 |
| H | -2.52288000 | 0.86637200 | -0.29898700 |
| H | -1.76870600 | 0.30008800 | -1.80258500 |
| C | -1.05740600 | -0.73517700 | 1.44361200 |
| H | -1.83434000 | -1.50806800 | 1.47923400 |
| H | -0.16902400 | -1.11343500 | 1.95231900 |

| | | | |
|---|---|---|---|
| H | -1.42930800 | 0.12890100 | 2.00079500 |
| O | -0.16372400 | -1.51979900 | -0.67824700 |
| H | -0.82802600 | -2.22059900 | -0.67142600 |
| C | 2.50521500 | -0.67575800 | -0.04702700 |
| H | 1.93014900 | -1.58630000 | 0.08536700 |
| H | 3.04828200 | -0.76074700 | -0.99632200 |
| H | 3.26797300 | -0.61227100 | 0.73802800 |
| H | 2.25581300 | 1.48504900 | -0.02330000 |

## 8. References


1. Yang, J.; Massaro, L.; Krajangsri, S.; Singh, T.; Su, H.; Silvi, E.; Ponra, S.; Eriksson, L.; Ahlquist, M. S. G.; Andersson, P. G. *J. Am. Chem. Soc.* **2021**, *143*, 21594– 21603
2. a) Fronza, G.; Fogliato, G.; Fuganti, C.; Lanati, S.; Rallo, R.; Servi, S. *Tetrahedron Lett.* **1995**, *36*, 121. b) Fogliato, G.; Fronza, G.; Fuganti, C.; Lanati, S.; Rallo, R.; Rigoni, R.; Servi, S. *Tetrahedron* **1995**, *51*, 10231.
3. Peters, B. B. C.; Jongcharoenkamol, J.; Krajangsri, S.; Andersson, P. G. *Org. Lett.* **2021**, *23*, 242–246
4. Chérest, M.; Felkin, H.; Prudent, N. *Tetrahedron Lett.* **1968**, *9*, 2199.ù
5. M. J. Frisch, G. W. Trucks, H. B. Schlegel, G. E. Scuseria, M. A. Robb, J. R. Cheeseman, G. Scalmani, V. Barone, B. Mennucci, G. A. Petersson, H. Nakatsuji, M. Caricato, X. Li, H. P. Hratchian, A. F. Izmaylov, J. Bloino, G. Zheng, J. L. Sonnenberg, M. Hada, M. Ehara, K. Toyota, R. Fukuda, J. Hasegawa, M. Ishida, T. Nakajima, Y. Honda, O. Kitao, H. Nakai, T. Vreven, J. A. Montgomery, Jr., J. E. Peralta, F. Ogliaro, M. Bearpark, J. J. Heyd, E. Brothers, K. N. Kudin, V. N. Staroverov, T. Keith, R. Kobayashi, J. Normand, K. Raghavachari, A. Rendell, J. C. Burant, S. S. Iyengar, J. Tomasi, M. Cossi, N. Rega, J. M. Millam, M. Klene, J. E. Knox, J. B. Cross, V. Bakken, C. Adamo, J. Jaramillo, R. Gomperts, R. E. Stratmann, O. Yazyev, A. J. Austin, R. Cammi, C. Pomelli, J. W. Ochterski, R. L. Martin, K. Morokuma, V. G. Zakrzewski, G. A. Voth, P. Salvador, J. J. Dannenberg, S. Dapprich, A. D. Daniels, O. Farkas, J. B. Foresman, J. V. Ortiz, J. Cioslowski, and D. J. Fox, Gaussian, Inc., Wallingford CT, 2013., 2011.
6. (a) Becke, A. D. J. Chem. Phys. **1993**, *98*, 5648–5652. (b) Lee, C.; Yang, W.; Parr, R. G. Phys. Rev. B. **1988**, *37*, 785–789.
7. Grimme, S.; Antony, J.; Ehrlich, S.; Krieg, H. J. Chem. Phys. 2010, 132, 154104.
8. Hay, P. J.; Wadt, W. R. J. Chem. Phys. **1985**, *82*, 270–283.
